# Supplementary material for: PPARγ, a key modulator of metabolic reprogramming, stemness and chemoresistance associated with retrodifferentiation in human hepatocellular carcinomas
Source: Cell Death Dis. 2025 Nov 17;16(1):831. doi: 10.1038/s41419-025-07799-3 (PMC12624014; doi:10.1038/s41419-025-07799-3)
Supplement: Supplementary file 1 — Supplemental material [file 41419_2025_7799_MOESM1_ESM.pdf]

Supplementary materials & methods

PPARγ, a key modulator of metabolic reprogramming, stemness and chemoresistance associated with retrodifferentiation in human hepatocellular carcinomas

Yoann Daniel[1], Claudine Rauch[1], Lucille Moutaux[1], Lise Desquilles[1], Tifenn Le Charpentier[1], Karim Fekir[1,2], Luis Cano[1], Daniel Catheline[1], Servane Pierre[1], Agnès Burel[3], Camille Savary[1], Catherine Ribault[1], Claude Bendavid[1,4], Bruno Clément[1], Caroline Aninat[1], Vincent Rioux[1], Orlando Musso[1], Bernard Fromenty[1], Florian Cabillic[1,4#*] and Anne Corlu[1,#*]

[1] Inserm, Univ Rennes, INRAE, NuMeCan Institute (Nutrition, metabolisms and cancer), Rennes, France

[2] Division of trauma and orthopaedic surgery, University of Cambridge, Addenbrooke's hospital, Hills road, Cambridge CB2 0QQ, UK

[3] Univ Rennes, CNRS, Inserm, Biosit UAR 3480 US_S 018, MRIC-TEM platform, Rennes, France

[4] CHU of Rennes, Pontchaillou site, Rennes, France

[#] Florian Cabillic and Anne Corlu contributed equally to this work.

Data acquisition, integration and processing

Transcriptomes across several time points of mouse liver development (GSE90047 [1] n = 21; GSE13149 [2], n = 25) were quantile-normalized. Orthologs were obtained through the *gorth* function (*gProfiler* R package [3]). Batch effect was assessed and corrected with the ComBat algorithm [4] (*sva* R package [5]).

Two transcriptomic datasets representing stepwise carcinogenic process GSE6764 [6] and GSE12443 [7] were retrieved and processed. For each dataset, probe sets corresponding to the PPARG and PPARA genes were extracted. When multiple probes mapped to the same gene, their expression values were averaged per sample.

GSE12443: To compare gene expression levels across different conditions – cirrhotic nodule (CN, n=48), low-grade dysplastic nodules (LGDN, n=6), high-grade dysplastic nodules (HGDN, n=24), early hepatocellular carcinoma (n=20) – a non-parametric Wilcoxon test was performed.

GSE6764 : Condition labels – normal liver tissue (n=10), cirrhotic liver tissue (n=13) low-grade dysplastic liver tissue (n=10), high-grade dysplastic liver tissue (n=7), early hepatocellular carcinoma (n=18) and advanced hepatocellular carcinoma (n=17), were harmonized to reduce the number of comparisons before performing the same statistical analysis.

Data visualization. For each dataset, boxplots were generated to visualise the expression levels of PPARA and PPARG across the various conditions. All analysis and visualisations were conducted in R.

Mitocarta gene set analysis

The reads per million normalized gene expression data (n=374 HCCs) and clinical data were retrieved from the TCGA database. Genes from the Mitocarta_up_REST and Mitocarta_dn_REST signatures were considered as up-regulated or down-regulated, respectively (REST : DEG between HepaRG -progenitors, -confluent and -differentiated cells versus HepaRG-SP and -Sphere. HCCs were classified as periportal, perivenous, ECM or STEM according to a hierarchical ascendant classification based on the Euclidean distance. PCA was computed using the *factoextra* and *FactoMineR* R packages and a biplot is plotted with confidence ellipses."

Survival analysis

Data from the Roessler (GSE14520, n = 247 HCCs) and ICGC (n = 232 HCCs) datasets were median-normalized using the DESeq2 R package. The reads per million normalized gene expression data (n=374 HCCs) from the TCGA database were also retrieved. The data relative to *PPARA*, *PPARG*, AFP levels and overall survival time and status were extracted and filtered for missing value. For each gene, patients were classified in "high expression" and "low expression" using the *surv_cutpoint* function for the *survival* R package. Survival curves were fitted using the cutpoints of 2 genes at time as covariates producing 4 groups of patients. The differences in prognosis were evaluated according to Kaplan-Meier method. At the end, AFP levels were evaluated for each group to strengthen their prognostic values.

Gene set enrichment analysis

Gene set enrichment analysis (GSEA) was used to compare the 6115 genes differentially expressed (DEG) between immature and differentiating HepaRG cells with the Mitocarta 3.0 Gene Set (1136 genes) [8]. Analysis was performed using the Java-tool developed at the Broad Institute (Cambridge, MA). 311 down-regulated genes in HepaRG-CSC were integrated into Enrichr website (https://maayanlab.cloud/Enrichr/).

siRNA transfection

The validated siRNA (LQ-003436-00-0002, On-TARGETplus Human PPARG 5468 siRNA – Set of 4, 2 nmol, Dharmacon) was used for knocking down the expression of *PPARG*. The ON-TARGETplus Non-targeting siRNA (D-001810-01-20, Dharmacon) was used as control. Transfection was performed using lipofectamine® RNAiMAX (Invitrogen, 13778-075). Briefly, one day after cell seeding, cells were incubated overnight with the liposome-DNA mix (1.5 µl RNAiMAX/25 pmoles DNA/10^5 cells) in Opti-MEM™ I Reduced Serum Medium

(Gibco, 31985-070). Expression of *PPARG* was analyzed 48 and 72 hours after transfection by RT-qPCR. Knock down of *PPARG* in HepaRG sphere was performed by electroporation. Treatments with drugs were performed 48h after transfection for 48 hours. For N-Acetyl Cysteine (NAC, Merck, A9165) experiments, NAC was added to the medium at time of transfection and then every day.

Drug treatments

In absence of pre-treatment, adherent cells or spheres were cultured for 3 days in the presence of cisplatin (Mylan), sorafenib (Interchim, CJ592), dichloroacetate (Sigma-Aldrich, 347795-10G), clofibrate (Sigma Aldrich, C6643-5G), rosiglitazone (Sigma-Aldrich, R2408), SC-79 (4µg/ml, Tebu Bio, T2274) or LY294002 (10µM, Tebu Bio, B-0294) at the indicated concentration. For NAC and/or T0070907 (Tebu Bio, T6689) treatment, 24h after cell seeding, cells were pre-incubated for 24h with or without NAC (1mM) and/or T0070907 (10µM). Then, cells were cultured for 48h with or without NAC (1mM) and/or T0070907 (10µM) in the presence of cisplatin (Mylan), sorafenib (Interchim, CJ592) or dichloroacetate (Sigma-Aldrich, 347795-10G). NAC was added every day. To study the impact of co-treatment on HepaRG- and BC2-spheres, cells were pre-treated 24h with clofibrate at 100 or 500µM before 72h of co-treatment with cisplatin, sorafenib or dichloroacetate.

Cytotoxicity and lipid peroxidation assays

Cytotoxicity assays were performed using CellTiter-Glo® Luminescent Cell Viability Assay (Promega, G7571) on cells. Lipid peroxides that result in the formation of malondialdehyde (MDA) were measured as Thiobarbituric Acid Reactive Substances with the TBARS kit (Bio-Techne, KGE013) on HepaRG supernatants. Luminescence was measured by POLARstar Omega plate reader (BMG Labtech).

Measurement of reactive oxygen species

Cells were incubated with 5 µM Mitosox (ThermoFisher Scientific, M36008) or 2 µM H2DCFDA (Molecular Probes, D399) in warm in Hank's balanced salt solution (HBSS) for 30 min at 37°C and 5% CO2 in the dark. The medium was removed and the cells were washed with warm HBSS and the fluorescence intensity was measured by the POLARstar Omega plate reader (BMG Labtech). Two measurements were performed for each well with the two required wavelength pairs for Mitosox (ex520/em590) and DFCDA (ex485/em520). For probe control, specific wells containing cells were treated with 60 mM H2O2 (Sigma-Aldrich, H1009) for 1 hour before probe incubation to validate probe activation.

Real-time PCR

Total RNA extraction and purification were performed using NucleoSpin RNA® Kit (Macherey-Nagel, 740955). Retrotranscription of RNA to cDNA was realized using High Capacity cDNA Reverse Transcription Kit (Applied Biosystems, 4368814). Total DNA extraction and purification were performed by DNeasy® Blood & Tissue Kit (Qiagen, 69504). Real-time qPCR was performed with Thermocycler StepOnePlus™ device (Applied Biosystems) using SYBR Green PCR Master Mix (Applied Biosystems, 4309155). Gene expression was normalized by housekeeping gene Tata Binding Protein for (*TBP*) cDNA and Ribosomal Protein S6 (*RPS6*) for DNA. The primer sequences are listed in the following table.

Primer	Gene name	Forward	Reverse
TBP	TATA-Box Binding Protein	5’-ACT-CCA-CTG-TAT-CCC-TCC-CC-3’	5’-CAG-CAA-ACC-GCT-TGG-GAT-TA-3’
ALB	Albumin	5’-TGCTTGAATGTGCTGATGACAGG-3’	5’-AAGGCAAGTCAGCAGGCATCTCATC -3’
CD44	CD44 Molecule	5’-GGC-TTT-CAA-TAG-CAC-CTT-GC-3’	5’-CAC-GTG-CCC-TTC-TAT-GAA-CC-3’
FAT/CD36	Fatty Acid Translocase	5’-GCC-AGG-TAT-TGC-AGT-TCT-TTT-C-3’	5’-TGT-CTG-GGT-TTT-CAA-CTG-GAG-3’
PPARG	Peroxisome Proliferator Activated Receptor Gamma	5’-AAG-GCC-ATT-TTC-TCA-AAC-GA-3’	5’-AGG-AGT-GGG-AGT-GGT-CTT-CC-3’

PPARG2	Peroxisome Proliferator Activated Receptor Gamma 2	5’-CCT ATT GAC CCA GAA AGC GA-3’	5’-TTA CGG AGA GAT CCA CGG AG-3’
PPARA	Peroxisome Proliferator Activated Receptor Alpha	5’-GTT-CTG-GAA-GCT-TTG-GCT-TTA-C-3’	5’-GAA-AGC-GTG-TCC-GTG-ATG-A-3’
PPARD	Peroxisome Proliferator Activated Receptor Delta	5’-AGC-ATC-CTC-ACC-GGC-AAA-G-3’	5’-CCA-CAA-TGT-CTC-GAT-GGC-AGG-CG-3’
PLIN2	Perilipin 2	5’-GCT-CCA-TTC-TAG-TGT-TCA-CCT-G-3’	5’-CTC-CTT-TTC-CAC-TCT-ACC-CAT-G-3’
PDK4	Pyruvate Dehydrogenase Kinase 4	5’-CTCGCGCTAGAGCCCG-3’	5’-GCATTTTCTGAACCAAAGTCCAG-3’
ANGPTL4	Angiopoietin Like 4	5’-GACCAAGGGGCATGGAGCTT-3’	5’-CAGGGGACCTACACACAACAG-3’
KLF4	Kruppel Like Factor 4	5’-GAC-GCT-GCT-GAG-TGG-AAG-AG-3’	5’-AGA-CAA-TCA-GCA-AGG-CGA-GT-3’
CDH1	Cadherin 1	5’-AGT-GGG-CAG-AGA-TGG-TGT-GA-3’	5’-TAG-GTG-GAG-TCC-CAG-GCG-TA-3’
RPS6	Ribosomal Protein S6	5’-TGA-TGT-CCG-CCA-GTA-TGT-TG-3’	5’-TCT-TGG-TAC-GCT-GCT-TCT-TC-3’

Protein extraction and western blot analysis

Cells were lysed in ice-cold RIPA buffer containing phosphatase and protease inhibitors (ThermoFisher Scientific, A32959). Protein concentration was determined using the Pierce BCA Protein Assay Kit (Thermo Fisher Scientific, 23227) and equal amounts of protein (20µg for HepaRG cells and 60µg for BC2 cells) were diluted with NuPAGE LDS Sample Buffer (Thermo Fisher Scientific, NP0007). Samples were then separated by SDS-PAGE using iD PAGE Gel 4–12% (Eurogentec, ID-PA4121-012). Proteins were transferred with iBlot Gel Transfer Stacks Nitrocellulose (Life Technologies, IB301001). Membranes were blocked in 3% BSA in TBST (10 mM Tris-HCl, 100 mM NaCl, 0.02% Tween 20) for 1h at room temperature and incubated overnight at 4 C with antibodies against PPARγ (Santa Cruz Biotechnology Cat# sc-7196, RRID:AB_654710), PPARα (Invitrogen Cat# MA5-37652, RRID:AB_2897578), E-Cadherin (Santa Cruz Biotechnology Cat# Sc8426, RRID:AB_626780), CD36 (Thermofisher Cat# PA5-27236, RRID:AB_2544712), PLIN2 (Biotechne Cat# NB110-40877, RRID:AB_787904), PDK4 (Proteintech Cat# 12949-1-AP, RRID:AB_2161499) or HSC70 (Santa Cruz Biotechnology Cat# sc-7298, RRID:AB_627761). Membranes were washed with TBST, incubated for 1h at room temperature with appropriate HRP secondary antibodies

(Agilent Cat# P0447, RRID:AB_2617137, and Cat# P0448, RRID:AB_2617138, respective dilutions 1:5000 and 1:10,000), washed with TBST, and then visualized by enhanced chemiluminescence (Pierce ECL Western Blotting Substrate; ThermoFisher Scientific) using Fusion FX imaging system (Vilber Lourmat). Protein content was quantified by densitometry with ImageJ software (National Institutes of Health, Bethesda, MD).

Mitochondrial mass by flow cytometry

Cells were incubated with 100 nM of MitoTracker® Green FM (ThermoFisher Scientific, M7514) for 30 min in HBSS (Gibco, 14175095). Cells were then washed in HBSS, detached with 0.05% tryspin (Gibco, 25300-054), resuspended in HBSS and ran through the LSR Fortessa X-20 flow cytometer.

Dosage of pyruvic and lactic acids

Supernatants of culture were collected and put into a tube containing 2 ml of perchloric acid. The medium had been renewed 24 hours before this sampling. Pyruvic acid and lactic acid determinations were performed by photometry using a COBAS c111 (ROCHE) in the biochemistry/toxicology laboratory of the University Hospital of Rennes.

Assessment of neutral lipids with Nile Red

Cells were washed with PBS, fixed and stained with PBS containing 4% formaldehyde and 10 µg/mL Hoechst 33342 dye for 30 min and washed 3 times with PBS. Cells were then incubated with PBS containing 0.1 µg/ml Nile Red (ThermoFisher Scientific, N1142) for 30 min and washed once. Fluorescence intensity was measured by the POLARstar Omega plate reader (BMG Labtech). Neutral lipids were then normalized per number of nuclei and expressed relative to control cells.

Lipid extraction, lipid species separation and fatty acid analysis

Total lipids were extracted twice from cells with hexane/isopropanol (3/2 v/v), after acidification with HCl 3M, as previously described [9]. Lipid species were then separated by thin-layer chromatography (TLC) using silica gel H plates (0.5 mm thickness) and a mixture of hexane:diethylether:acetic acid (85:15:1 v/v/v) for development, after addition of internal standards (diheptadecanoylphosphatidycholine, heptadecanoic acid, triheptadecanoylglycerol and cholesteryl heptadecanoate) [10]. Phospholipids (PL), free fatty acids (FFA), triglycerides (TG) and cholesterol esters (CE) were scraped off the plates and extracted with 2 mL of methanol. Total lipids and lipid species were converted to Fatty Acid Methyl Esters (FAMEs), by successive saponification (1 mL of 0.5 M NaOH in methanol at 70°C for 30 min) and methylation (1 mL of BF_3 14% in methanol at 70°C for 30 min) [11]. Gas chromatography-mass spectrometry (GC-MS) analysis of FAMEs was subsequently performed using an Agilent Technologies 7890A GC system (Agilent, Les Ulis, France) with a bonded silica capillary column (BPX 70, 60 m × 0.25 mm; SGE, Melbourne, Australia) containing a polar stationary phase of 70% cyanopropyl polysilphenylene-siloxane (0.25 μm film thickness). Helium was used as carrier gas (average velocity 24 cm/s). The column temperature program started at 150°C and gradually increased at 4°C/min to 250°C, and held at 250°C for 10 min. Mass spectra were recorded with an Agilent Technologies 5975C inert MSD with triple axis detector [12]. The mass spectrometer was operated under electron impact ionization conditions (electron energy 70 eV, source temperature 230°C). Data were obtained in the full scan mode with a mass range of m/z 50–550 atomic mass units (amu). Peak integration was performed with MassHunter Workstation Software Qualitative Analysis Version B.07.00 for Windows (Agilent Technologies). Identification of the FAMEs was based upon retention times (Rt) obtained for methyl ester of authentic standards, when available. The National Institute of Standards and

Technology database (NIST version 2.2) was used to identify unknown fatty acids. All identified fatty acids with a signal/noise above 10 were considered in the analysis. Results were expressed as the mass of identified fatty acids/cell number.

Electron microscopy

Cells were fixed with 2.5% glutaraldehyde (Sigma-Aldrich, G7526) and treated by 1% osmium (Electron Microscope Sciences, 19150) in 0.2M sodium cacodylate buffer pH=7.2 (Electron Microscope Sciences, 11650). The dehydration was carried out by successive alcohol baths (increasing alcohol content: 50%, 70%, 80%, 90%, 100%) followed by twice impregnation with an Epon / DMP30 mixture (Sigma-Aldrich, 45348) and placed at 37°C. The next day, capsules were inserted into the same resin and the plates were placed at 37°C. Two days later, the capsules were filled with an Epon/DMP30 mixture and placed in an oven at 60°C to polymerize for 24 hours. The capsules were detached from the plates and cut with an ultra-microtome (UC7, Leica). Sections were stained with uranyl acetate and the images acquired with a Gatan Orius SC1000 wide angle camera on a JEOL JEM-1400 electron microscope. For each cell type, the TEM experiments were carried out at least twice. At least two capsules were cut by experiment and for each section examined by TEM, 4 different fields were investigated in order to overview the cells layer.

Confocal microscopy

HepaRG cell line was cultured in compartmented Lab-Tek™. After culture, cells were fixed with 4% paraformaldehyde (ThermoFisher Scientific, 28908) and permealibilized by PBS with 1% BSA (Sigma-Aldrich, A2153) and 0.5% saponin (Sigma-Aldrich, 47036) for 1h at room temperature. First incubation was done with antibody against mitochondrial import receptor subunit (TOM22) at 1/5000 (Abcam Cat# ab57523, RRID:AB_945897) in PBS with 1% BSA

and 0.5% saponin overnight at 4°C. Next day, compartmented slats were washed with PBS and secondary anti-mouse antibody coupled with green FITC (Jackson ImmunoResearch Labs Cat# 115-095-003, RRID:AB_2338589) for 1 hour at room temperature, followed by PBS washing and Hoescht 33342 labelling for 15 min. The cells were observed with a LEICA DMI 6000 CS confocal microscope. The acquisition of images was performed with the LAS AF software. Image analysis was performed with ImageJ software, based on the work of Koopman et al [13].

Immunohistochemistry

Tissue Microarray (TMA) comprises 58 HCC patients and 5 histologically normal livers, all in triplicate as previously described [14]. Briefly, TMA construction was done with a MiniCore3 tissue arrayer (Alphelys, France). Five-µm microtome sections were processed for immunohistochemistry with a Discovery XT from Ventana Medical Systems (Roche) slide staining system. PPARG antibodies used were purchased from Invitrogen (Cat# MA5-14889, RRID:AB_10985650). Stained slides were converted into high-resolution digital data with a NanoZoomer digital slide scanner (Hamamatsu, France). Digital slides were viewed using NDP.view software (Hamamatsu).

References

1. Yang L, Wang W-H, Qiu W-L, Guo Z, Bi E, Xu C-R. A single-cell transcriptomic analysis reveals precise pathways and regulatory mechanisms underlying hepatoblast differentiation. Hepatology 2017;66:1387–1401.

2. Li T, Huang J, Jiang Y, Zeng Y, He F, Zhang MQ, et al. Multi-stage analysis of gene expression and transcription regulation in C57/B6 mouse liver development. Genomics 2009;93:235–242.

3. Kolberg L, Raudvere U, Kuzmin I, Vilo J, Peterson H. gprofiler2 -- an R package for gene list functional enrichment analysis and namespace conversion toolset g:Profiler. F1000Res 2020;9:ELIXIR-709.

4. Johnson WE, Li C, Rabinovic A. Adjusting batch effects in microarray expression data using empirical Bayes methods. Biostatistics 2007;8:118–127.

5. Liu Q, Markatou M. Infect Dis Transl Med 2016;2(1):3-9.

6. Wurmbach E, Chen YB, Khitrov G, Zhang W, Roayaie S, Schwartz M et al. Genome-wide molecular profiles of HCV-induced dysplasia and hepatocellular carcinoma. Hepatology. 2007 Apr;45(4):938-47.

7. Kaposi-Novak P, Libbrecht L, Woo HG, Lee YH, Sears NC, Coulouarn C et al. Central role of c-Myc during malignant conversion in human hepatocarcinogenesis. Cancer Res. 2009 Apr 1;69(7):2775-82.

8. Calvo SE, Clauser KR, Mootha VK. MitoCarta2.0: an updated inventory of mammalian mitochondrial proteins. Nucleic Acids Res 2016;44:D1251–D1257.

9. Drouin G, Catheline D, Guillocheau E, Gueret P, Baudry C, Le Ruyet P, et al. Comparative effects of dietary n-3 docosapentaenoic acid (DPA), DHA and EPA on plasma lipid parameters, oxidative status and fatty acid tissue composition. The Journal of Nutritional Biochemistry 2019;63:186–196.

10. Garcia C, Guillocheau E, Richard L, Drouin G, Catheline D, Legrand P, et al. Conversion of dietary trans-vaccenic acid to trans11,cis13-conjugated linoleic acid in the rat lactating mammary gland by Fatty Acid Desaturase 3-catalyzed methyl-end Δ13-desaturation. Biochem Biophys Res Commun 2018;505:385–391.

11. Guillocheau E, Garcia C, Drouin G, Richard L, Catheline D, Legrand P, et al. Retroconversion of dietary trans-vaccenic (trans-C18:1 n-7) acid to trans-palmitoleic acid (trans-C16:1 n-7): proof of concept and quantification in both cultured rat hepatocytes and pregnant rats. J Nutr Biochem 2019;63:19–26.

12. Rioux V, Pédrono F, Blanchard H, Duby C, Boulier-Monthéan N, Bernard L, et al. Trans-vaccenate is Δ13-desaturated by FADS3 in rodents. J Lipid Res 2013;54:3438–3452.

13. Koopman WJ, Verkaart S, Visch HJ, van der Westhuizen FH, Murphy MP, van den Heuvel LW, et al. Inhibition of complex I of the electron transport chain causes $O2^{-\cdot}$-mediated mitochondrial outgrowth. Am J Physiol 2005;288:C1440–C1450.

14. Mebarki S, Désert R, Sulpice L, Sicard M, Desille M, Canal F, et al. De novo HAPLN1 expression hallmarks Wnt-induced stem cell and fibrogenic networks leading to aggressive human hepatocellular carcinomas. Oncotarget 2016;7:39026–39043.

Supplementary figure legends

PPARγ, a key modulator of metabolic reprogramming, stemness and chemoresistance associated with retrodifferentiation in human hepatocellular carcinomas

Yoann Daniel[1], Claudine Rauch[1], Lucille Moutaux[1], Lise Desquilles[1], Tifenn Le Charpentier[1], Karim Fekir[1,2], Luis Cano[1], Daniel Catheline[1], Servane Pierre[1], Agnès Burel[3], Camille Savary[1], Catherine Ribault[1], Claude Bendavid[1,4], Bruno Clément[1], Caroline Aninat[1], Vincent Rioux[1], Orlando Musso[1], Bernard Fromenty[1], Florian Cabillic[1,4#*] and Anne Corlu[1,#]

[1] Inserm, Univ Rennes, INRAE, NuMeCan Institute (Nutrition, metabolisms and cancer), Rennes, France

[2] Division of trauma and orthopaedic surgery, University of Cambridge, Addenbrooke's hospital, Hills road, Cambridge CB2 0QQ, UK

[3] Univ Rennes, CNRS, Inserm, Biosit UAR 3480 US_S 018, MRIC-TEM platform, Rennes, France

[4] CHU of Rennes, Pontchaillou site, Rennes, France

[#] Florian Cabillic and Anne Corlu contributed equally to this work.

Supplementary Fig. 1

A Phase-contrast microscopy of HepaRG cells during differentiation/retrodifferentiation. Bar=50µm. **B** Non-supervised gene set enrichment analysis (GSEA) plot from KEGG gene set database performed using the differentially expressed genes (DEG) between immature (Sphere

and SP) and differentiating (progenitors, committed/confluent and differentiated) HepaRG cell groups. **C** Basal and maximal respiration assessed with Seahorse® analyzer. HepaRG were treated 24h with a glutamine antagonist (DON) or cultured in medium without glutamine (n=3). * p<0.05, ** p<0.01, *** p<0.001 in comparison with the sphere condition; $ p<0.05, $$ p<0.01, $$$ p<0.001 in comparison with the progenitor condition. **D** mRNA expression of *JUN, FOS, CDKN1A, CDKN1B, CDK4, CCND1, CDK1* and *CCNB1*. Results are expressed as relative to HepaRG progenitors, arbitrary set to 1 (n=4). * p<0.05, ** p<0.01, *** p<0.001.

Supplementary Fig. 2

A Phase-contrast microscopy of BC2, Huh7, HepG2 and Huh6 in sphere conformation (upper panel) and proliferative or differentiated stages (lower panel); Bar=50µm. **B** mRNA expression of *PPARGC1A/*PGC1a in HepaRG, BC2, Huh-7, HepG2 and Huh6 cells at proliferative/differentiated stages or in sphere conformation. Results are expressed as relative to differentiated cells for HepaRG and BC2 cell lines or relative to proliferative cells for Huh7, HepG2 and Huh6 cell lines, arbitrary set to 1 (n = 3). * p<0.05, ** p<0.01, *** p<0.001. **C** mRNA expression of *PPARG2* throughout the differentiation process of HepaRG and BC2 cells: Sph=spheres, SP=side population, Prog=progenitors, Conf=committed/confluent, Diff=differentiated, Pro=proliferative. Results are expressed relative to HepaRG-progenitors or BC2-proliferative cells (n≥3). Ct=Cycle threshold. *p<0.05, **p<0.01, ***p<0.001. **D** mRNA expression *PPARG* in pre-neoplastic lesions (cirrhosis, low grade (LG) dysplastic nodules, high grade (HG) dysplastic nodules), early HCC and advanced HCC. Transcriptomic datasets obtained from Gene Expression Omnibus (GEO) database GSE12443 (Kaposi-Novac’s cohort) (Panels A, C) and GSE6764 Wurmbach’s cohort) (Panels B, D). *p<0.05, **p<0.01, ***p<0.001.

Supplementary Fig. 3

A Overall survivals according *PPARA/PPARG* expression in the ICGC cohort. **B** Overall survivals according *PPARA/PPARG* expression in the Roessler cohort. **C** Correlation between *PPARA/PPARG* expression and AFP levels in the Roessler cohort.

Supplementary Fig. 4

A mRNA expression of *PPARG*, *PPARA* and *PDK4* in HepaRG progenitors after treatment with increasing concentrations of rosiglitazone (50 and 100µM) or clofibrate (50, 100, 300, 500 and 1000µM) during 24 or 48h. Results are expressed as relative to the untreated cells, arbitrary set to 1 (n=3). *p<0.05, *** p<0.001. **B** Left panel: PPARγ expression assessed by western blot 24, 48 or 72h after transfection of HepaRG progenitors by 4 different si*PPARG*; Right panel: PPARγ expression assessed by western blot 72h after transfection of HepaRG progenitors by si*PPARG*-4 (n=3). HSC70 protein was used as normalization protein. **C** Upper left panel: production of peroxides (reactive oxygen species) by HepaRG-spheres after DCA treatment during 12h, 24h, 48h and 72h, assessed using H2DCFDA (n=3); Upper right panel: production of peroxides by HepaRG-progenitors after siRNA transfection during 12h, 24h, 48h and 72h, assessed using H2DCFDA. *p<0.05, ** p<0.01, *** p<0.001. Lower left panel: Phase-contrast microscopy of differentiated HepaRG cells; Lower right panel: production of peroxide and superoxide anions (reactive oxygen species) by differentiated HepaRG cells after DCA treatment during 12h, 24h, 48h and 72h, assessed using H2DCFDA or Mitosox®, respectively (n=3). ** p<0.01.

Supplementary Fig. 5

A Heatmap of metabolic redox regulatory gene among the 6115 differentially expressed genes (DEG, p≤0.05, FC>1.5) identified between the HepaRG-CSCs (sphere and side population) and

the HepaRG differentiating cells i.e., progenitors, committed/confluent and differentiated cells. **B** Immunostaining of the mitochondrial protein TOM22 in HepaRG progenitors 72h after treatment with 50µM rosiglitazone or transfection by si*PPARG*. Image acquisition was performed by confocal microscopy. Bar=10µm. Mitochondrial network branching and length were analyzed using ImageJ software (n=3). ***$p<0.001$. **C** Immunostaining of the mitochondrial protein TOM22 in HepaRG progenitors 48h after treatment with 100µM clofibrate. Image acquisition was performed by confocal microscopy. Bar=10µm. Mitochondrial network branching and length were analyzed using ImageJ software (n=3). * $p<0.05$.

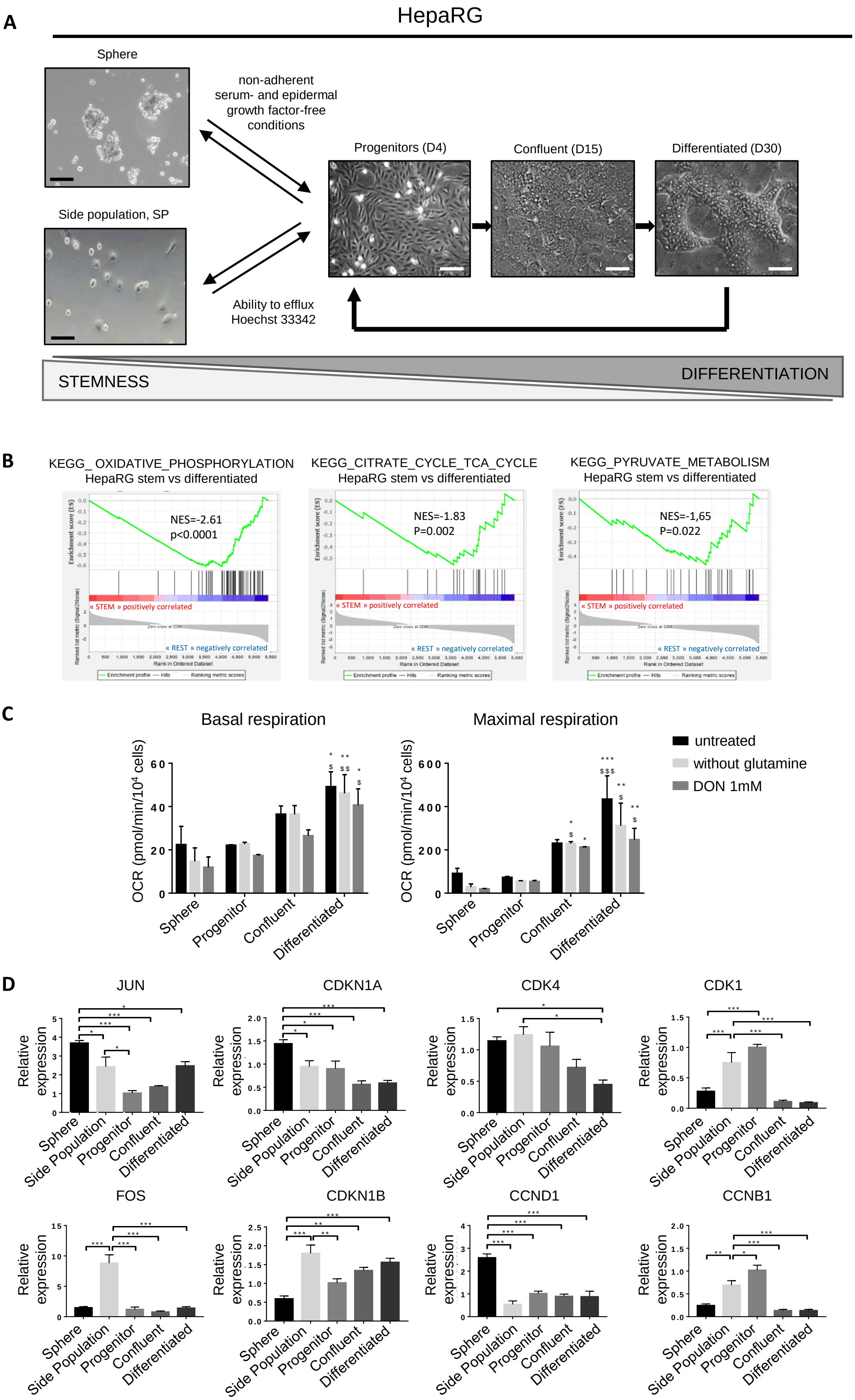

Supplementary Figure 1

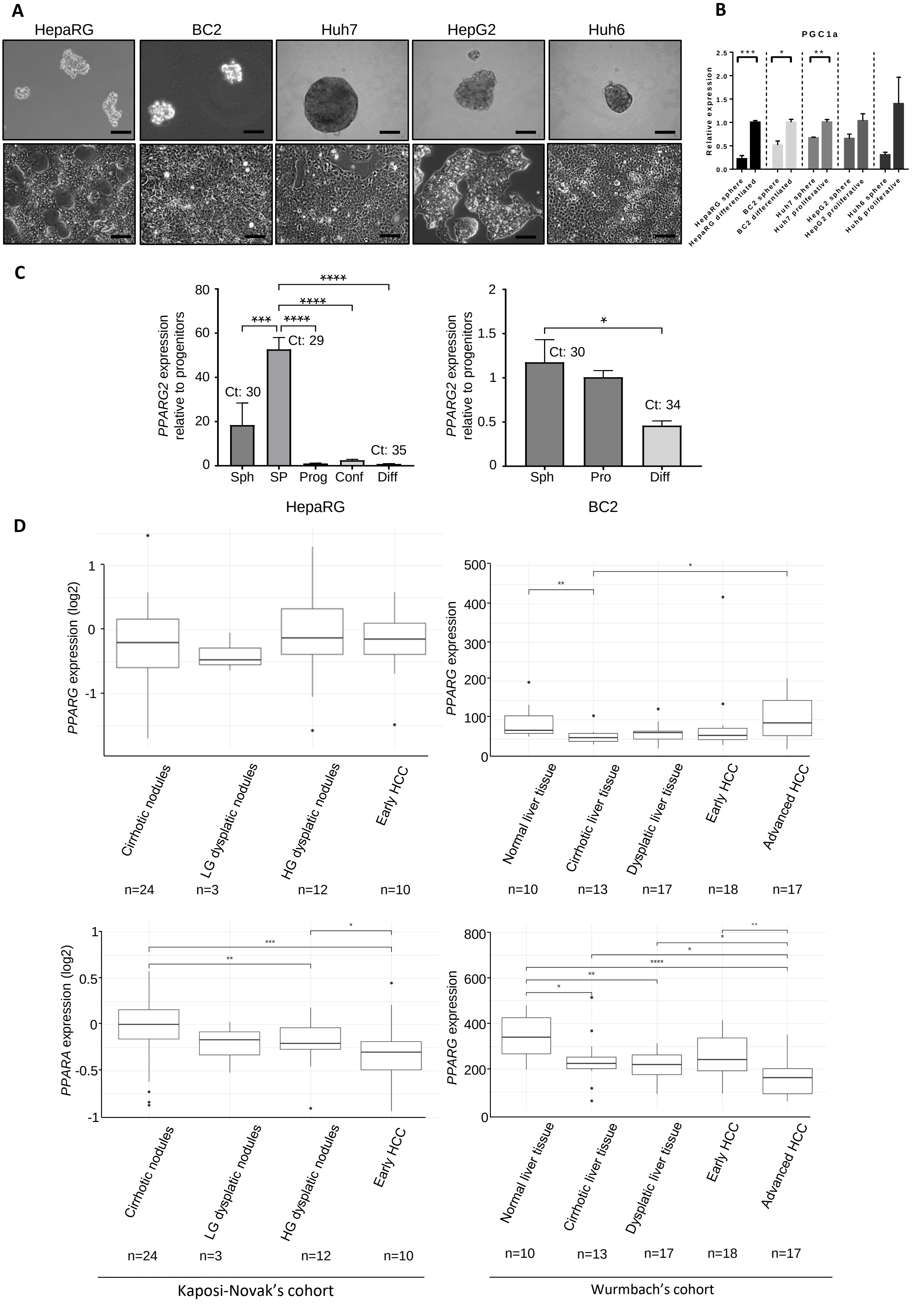

Supplementary Figure 2

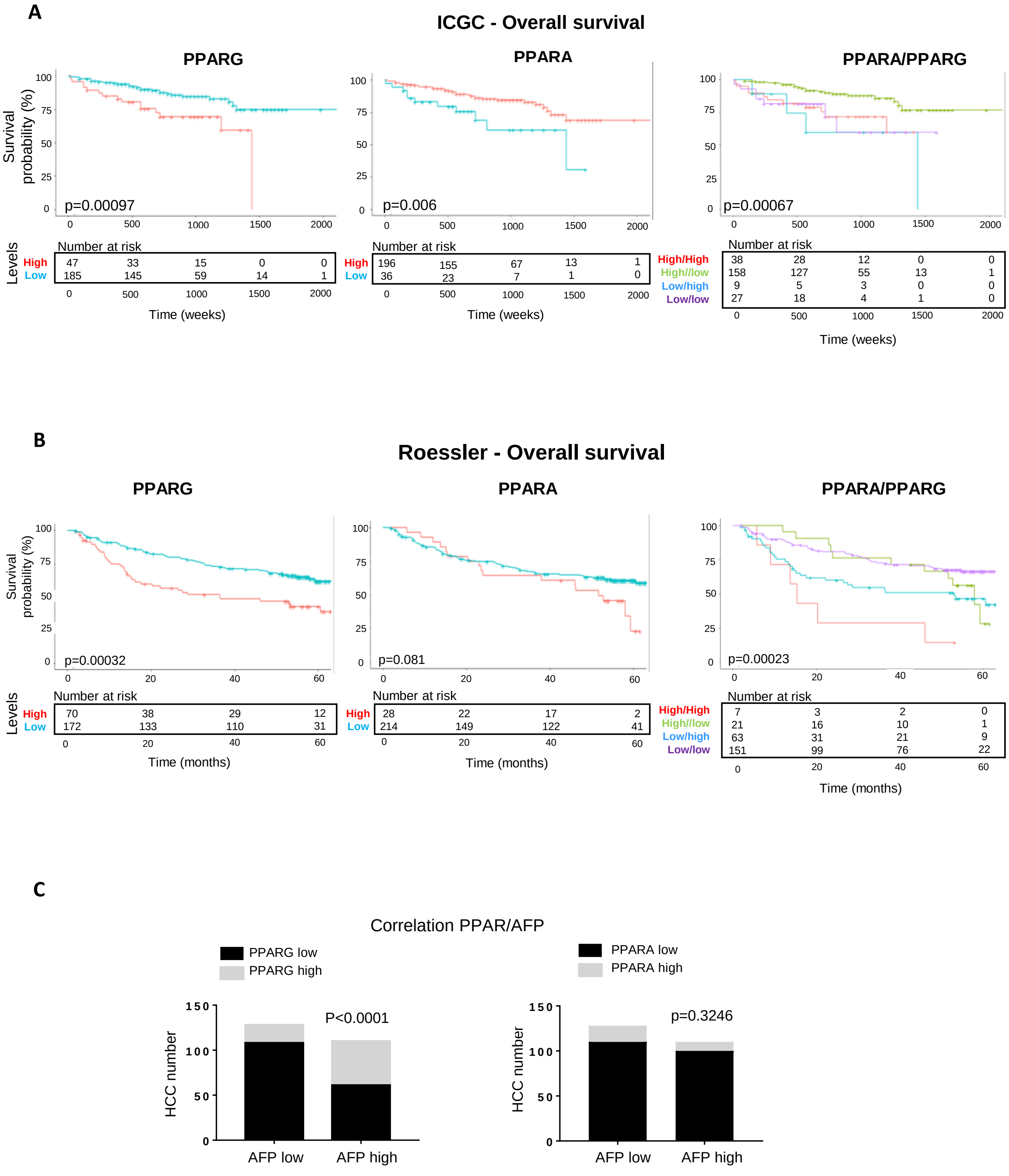

Supplementary Figure 3

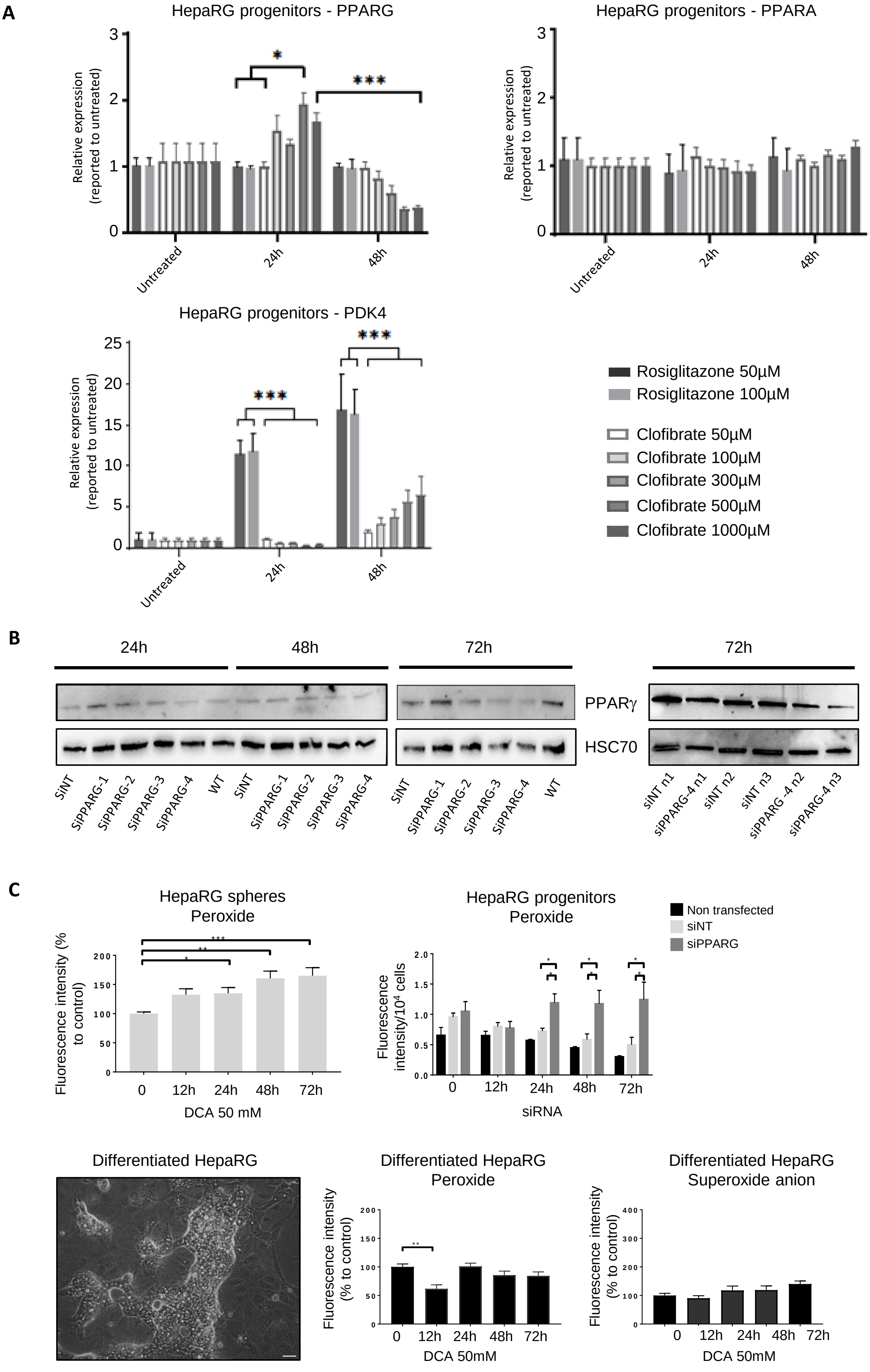

Supplementary Figure 4

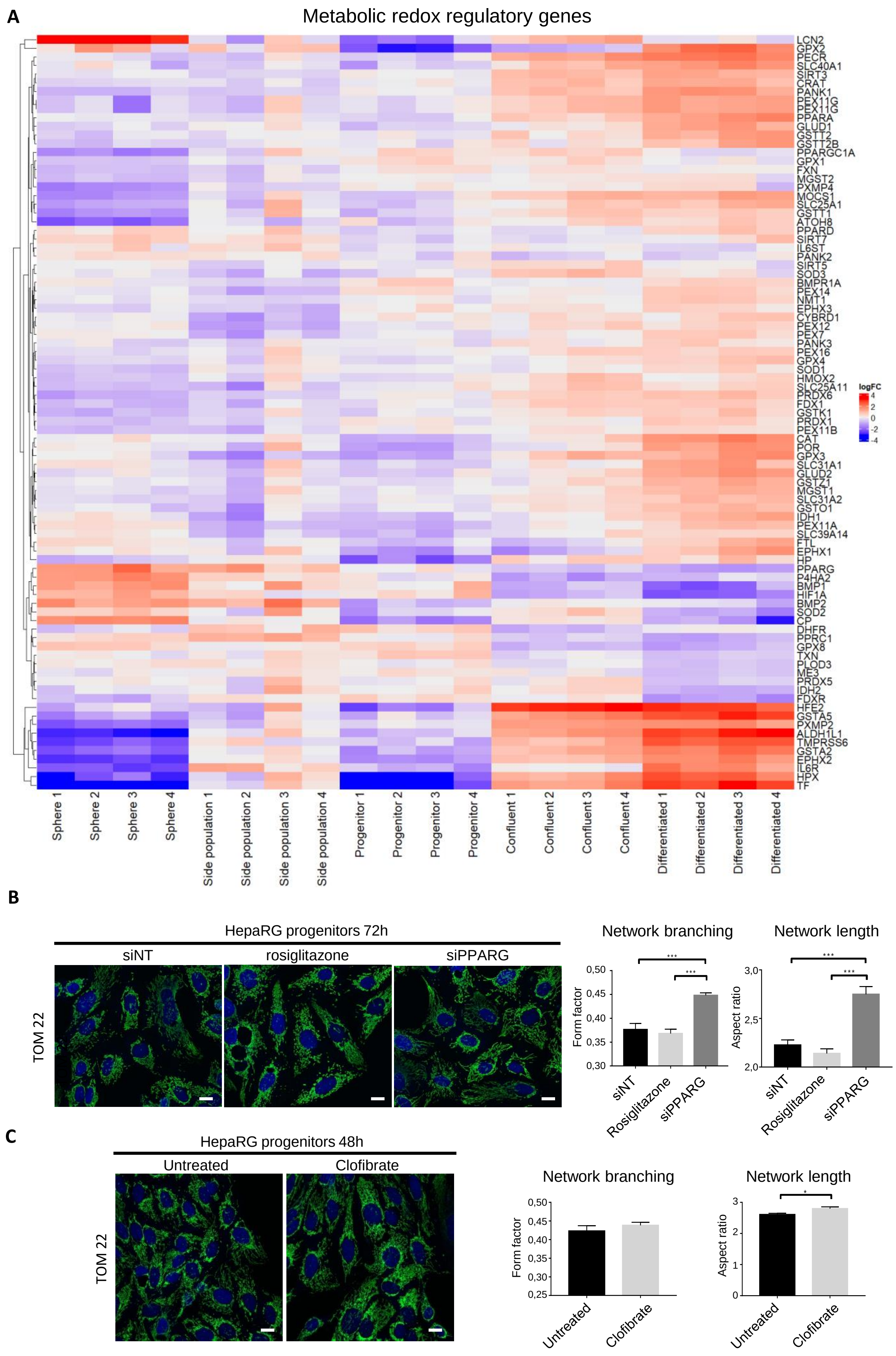

Supplementary Figure 5

Supplementary Table 1: List of 808 differentially expressed genes (DEG) significantly deregulated between progenitor HepaRG and differentiated HepaRG using t-test (fold change>2; p<0.05)

Gene	ExpressedIn
A1CF	Differentiated
AADAC	Differentiated
AASS	Differentiated
ABAT	Differentiated
ABCA1	Differentiated
ABCA6	Differentiated
ABCB1	Differentiated
ABCB4	Differentiated
ABCG5	Differentiated
ABCG8	Differentiated
ABHD15	Differentiated
ABI3	Differentiated
ABR	Progenitor
ACAA1	Differentiated
ACACB	Differentiated
ACAD11	Differentiated
ACKR3	Progenitor
ACMSD	Differentiated
ACOT12	Differentiated
ACOX2	Differentiated
ACSM2B	Differentiated
ACSM3	Differentiated
ACSM5	Differentiated
ACTG2	Progenitor
ACTL8	Progenitor
ACTN1	Progenitor
ADAM12	Progenitor
ADAMTS9	Progenitor
ADAP2	Differentiated
ADCY3	Progenitor
ADH1A	Differentiated
ADH1B	Differentiated
ADH1C	Differentiated
ADH4	Differentiated
ADH6	Differentiated
ADHFE1	Differentiated
AFAP1L2	Progenitor
AFM	Differentiated
AGMO	Differentiated
AGR2	Progenitor
AGT	Differentiated
AGXT	Differentiated
AGXT2	Differentiated
AHSG	Differentiated
ALB	Differentiated
ALDH1L1	Differentiated
ALDH2	Differentiated

ALDH5A1	Differentiated
ALDH6A1	Differentiated
ALDOB	Differentiated
ALOX5	Progenitor
ALPP	Progenitor
AMBP	Differentiated
AMDHD1	Differentiated
AMPH	Progenitor
AMTN	Progenitor
ANG	Differentiated
ANGPTL3	Differentiated
ANK1	Progenitor
ANKRD33B	Progenitor
ANKS4B	Differentiated
ANLN	Progenitor
ANXA10	Differentiated
ANXA3	Progenitor
ANXA8L1	Progenitor
ANXA9	Differentiated
AOX1	Differentiated
APBA1	Differentiated
APCDD1L	Progenitor
APOA1	Differentiated
APOA2	Differentiated
APOA4	Differentiated
APOA5	Differentiated
APOB	Differentiated
APOBEC3B	Progenitor
APOBEC3C	Progenitor
APOBEC3F	Progenitor
APOC1	Differentiated
APOC3	Differentiated
APOH	Differentiated
APOM	Differentiated
AQP11	Differentiated
AQP3	Differentiated
AQP7	Differentiated
AQP7P1	Differentiated
AQP7P3	Differentiated
AQP9	Differentiated
ARG1	Differentiated
ARHGDIB	Progenitor
ARL14	Progenitor
ARMC9	Progenitor
ARNT2	Progenitor
ARRDC3	Differentiated
ASAP1	Progenitor
ASB13	Differentiated
ASF1B	Progenitor
ASGR1	Differentiated

ASGR2	Differentiated
ASPM	Progenitor
ATP6V0E2	Differentiated
AUNIP	Progenitor
AURKA	Progenitor
AZGP1	Differentiated
BAAT	Differentiated
BBOX1	Differentiated
BCL2L15	Differentiated
BCO2	Differentiated
BDH1	Differentiated
BFSP1	Progenitor
BHMT2	Differentiated
BIRC3	Progenitor
BIRC5	Progenitor
BLM	Progenitor
BMPER	Progenitor
BNC2	Progenitor
BTD	Differentiated
BTG1	Differentiated
BTG3	Progenitor
BTNL8	Differentiated
BUB1	Progenitor
BUB1B	Progenitor
C11orf52	Differentiated
C11orf54	Differentiated
C11orf97	Differentiated
C1orf115	Differentiated
C1orf226	Differentiated
C1QTNF4	Differentiated
C2orf72	Differentiated
C3P1	Differentiated
C4BPA	Differentiated
C4orf46	Progenitor
C5	Differentiated
C6	Differentiated
C8A	Differentiated
C8B	Differentiated
C8G	Differentiated
CA9	Progenitor
CACFD1	Differentiated
CALB2	Progenitor
CALCR	Progenitor
CALML4	Differentiated
CAPN5	Differentiated
CARM1	Differentiated
CAT	Differentiated
CAV1	Progenitor
CCBE1	Progenitor
CCDC170	Differentiated

CCDC178	Progenitor
CCDC80	Progenitor
CCK	Progenitor
CCL2	Progenitor
CCNA2	Progenitor
CCNB1	Progenitor
CD109	Progenitor
CD70	Progenitor
CD83	Progenitor
CDA	Progenitor
CDC20	Progenitor
CDC45	Progenitor
CDCA2	Progenitor
CDCA5	Progenitor
CDCA7	Progenitor
CDCA8	Progenitor
CDH1	Differentiated
CDH11	Progenitor
CDH16	Progenitor
CDK1	Progenitor
CDO1	Differentiated
CDR2L	Progenitor
CDT1	Progenitor
CEBPA	Differentiated
CEMIP	Progenitor
CENPA	Progenitor
CENPF	Progenitor
CENPM	Progenitor
CENPU	Progenitor
CENPW	Progenitor
CEP55	Progenitor
CFAP70	Differentiated
CFB	Differentiated
CFH	Differentiated
CFHR1	Differentiated
CFHR2	Differentiated
CFHR4	Differentiated
CHAF1B	Progenitor
CHI3L2	Progenitor
CHN2	Differentiated
CHST3	Progenitor
CIB2	Progenitor
CIDEB	Differentiated
CIT	Progenitor
CKAP2L	Progenitor
CKS2	Progenitor
CLDN14	Differentiated
CLDN3	Differentiated
CLRN3	Differentiated
CLU	Differentiated

CNN1	Progenitor
CNN2	Progenitor
COBL	Differentiated
COL1A1	Progenitor
COL4A1	Progenitor
COL4A2	Progenitor
COL4A6	Progenitor
COL5A1	Progenitor
COL8A1	Progenitor
COX6A2	Differentiated
CPB2	Differentiated
CPS1	Differentiated
CREB3L3	Differentiated
CRYL1	Differentiated
CTH	Differentiated
CTSC	Progenitor
CXCL1	Progenitor
CXCL13	Differentiated
CXCL14	Progenitor
CYBA	Differentiated
CYP1A2	Differentiated
CYP1B1	Progenitor
CYP2A13	Differentiated
CYP2A7	Differentiated
CYP2B6	Differentiated
CYP2C18	Differentiated
CYP2C19	Differentiated
CYP2C8	Differentiated
CYP2C9	Differentiated
CYP2E1	Differentiated
CYP3A4	Differentiated
CYP3A5	Differentiated
CYP3A7	Differentiated
CYP4A11	Differentiated
CYP4B1	Differentiated
CYP4F12	Differentiated
CYP4F2	Differentiated
CYP4F3	Differentiated
CYP4F8	Differentiated
CYP7A1	Differentiated
CYP8B1	Differentiated
CYTL1	Progenitor
DAO	Differentiated
DBN1	Progenitor
DCAF11	Differentiated
DCN	Progenitor
DCXR	Differentiated
DDIAS	Progenitor
DEFB1	Differentiated
DEFB103B	Progenitor

DEPDC1	Progenitor
DGAT2	Differentiated
DGKA	Progenitor
DHRS3	Differentiated
DHTKD1	Differentiated
DIAPH3	Progenitor
DIRAS1	Progenitor
DIRAS3	Differentiated
DKK1	Progenitor
DLGAP5	Progenitor
DMGDH	Differentiated
DPYS	Differentiated
DPYSL3	Progenitor
DSE	Progenitor
DTL	Progenitor
E2F1	Progenitor
E2F2	Progenitor
E2F7	Progenitor
EBI3	Progenitor
ECHDC2	Differentiated
ECM1	Progenitor
EDIL3	Progenitor
EDN1	Progenitor
EDN3	Progenitor
EFEMP1	Progenitor
EHHADH	Differentiated
ELF4	Progenitor
EMP3	Progenitor
ENC1	Progenitor
ENTPD8	Differentiated
EPB41L1	Progenitor
EPB41L5	Differentiated
EPHX1	Differentiated
EPHX2	Differentiated
EPYC	Progenitor
ERBB3	Differentiated
ERP27	Differentiated
ERRFI1	Differentiated
ESPN	Differentiated
ETNK2	Differentiated
ETNPPL	Differentiated
EVA1C	Progenitor
EXO1	Progenitor
EYA1	Differentiated
F10	Differentiated
F11	Differentiated
F12	Differentiated
F13B	Differentiated
F2	Differentiated
F2R	Progenitor

F3	Progenitor
F5	Differentiated
F7	Differentiated
F9	Differentiated
FABP1	Differentiated
FAM111B	Progenitor
FAM151A	Differentiated
FAM167A	Progenitor
FAM198B	Progenitor
FAM20A	Differentiated
FAM81A	Progenitor
FANCA	Progenitor
FANCI	Progenitor
FAP	Progenitor
FAXDC2	Differentiated
FBLIM1	Progenitor
FCGRT	Differentiated
FEN1	Progenitor
FETUB	Differentiated
FGA	Differentiated
FGB	Differentiated
FGG	Differentiated
FGL1	Differentiated
FHL2	Progenitor
FILIP1L	Progenitor
FLNA	Progenitor
FMO5	Differentiated
FOLH1	Differentiated
FOLH1B	Differentiated
FOXL1	Progenitor
FOXM1	Progenitor
FPR1	Progenitor
FRMD5	Progenitor
FRMD6	Progenitor
FRY	Differentiated
FST	Progenitor
FSTL3	Progenitor
FXYD3	Progenitor
FXYD5	Progenitor
FYN	Differentiated
FZD2	Progenitor
FZD4	Differentiated
G6PC	Differentiated
GALNT5	Progenitor
GATM	Differentiated
GBA3	Differentiated
GBP7	Differentiated
GC	Differentiated
GCDH	Differentiated
GCHFR	Differentiated

GCKR	Differentiated
GINS1	Progenitor
GINS2	Progenitor
GINS4	Progenitor
GJB1	Differentiated
GLDC	Differentiated
GLIPR1	Progenitor
GLS2	Differentiated
GLTPD2	Differentiated
GLYAT	Differentiated
GLYATL1	Differentiated
GLYCTK	Differentiated
GNG4	Progenitor
GNMT	Differentiated
GOLT1A	Differentiated
GPD1	Differentiated
GPR84	Differentiated
GPR87	Progenitor
GPRC5A	Progenitor
GPRC5B	Progenitor
GPT	Differentiated
GPX2	Differentiated
GPX3	Differentiated
GRAMD1C	Differentiated
GRIN2C	Progenitor
GRIN2D	Differentiated
GRTP1	Differentiated
GSTA2	Differentiated
GSTA5	Differentiated
GTSE1	Progenitor
H19	Progenitor
HAAO	Differentiated
HABP2	Differentiated
HAGH	Differentiated
HAO1	Differentiated
HAO2	Differentiated
HAPLN1	Progenitor
HELLS	Progenitor
HGD	Differentiated
HHEX	Differentiated
HIF1A	Progenitor
HIST1H1B	Progenitor
HIST1H2AC	Differentiated
HIST1H2AI	Progenitor
HIST1H2AL	Progenitor
HIST1H3B	Progenitor
HJURP	Progenitor
HLF	Differentiated
HMGCL	Differentiated
HMGCS2	Differentiated

HMMR	Progenitor
HNF1A-AS1	Differentiated
HOMER2	Differentiated
HOMER3	Progenitor
HOXB9	Progenitor
HPD	Differentiated
HPGD	Differentiated
HPN	Differentiated
HPR	Differentiated
HPX	Differentiated
HRG	Differentiated
HSD11B1	Differentiated
HSD17B11	Differentiated
HSD17B3	Differentiated
HSD17B6	Differentiated
HSPB3	Progenitor
HSPG2	Progenitor
ICAM1	Progenitor
ID2	Differentiated
IDNK	Differentiated
IGF1	Differentiated
IGF2-AS	Differentiated
IGFBP3	Progenitor
IGFBP6	Progenitor
IGFBP7	Progenitor
IGFL2	Progenitor
IGFL3	Progenitor
IL22RA1	Differentiated
IL2RG	Differentiated
IL6R	Differentiated
IL7R	Progenitor
INPP5D	Progenitor
IQGAP3	Progenitor
ITGA7	Differentiated
ITGB4	Progenitor
ITIH1	Differentiated
ITIH2	Differentiated
ITIH4	Differentiated
ITPR3	Progenitor
IYD	Differentiated
KBTBD13	Differentiated
KCNK5	Differentiated
KCNN4	Progenitor
KDELC2	Progenitor
KHK	Differentiated
KIF11	Progenitor
KIF20A	Progenitor
KIF22	Progenitor
KIF23	Progenitor
KIF2C	Progenitor

KIF4A	Progenitor
KLB	Differentiated
KLHDC9	Differentiated
KLHL30	Progenitor
KLKB1	Differentiated
KNG1	Differentiated
KRT34	Progenitor
KRT4	Progenitor
KRT5	Progenitor
KRT6A	Progenitor
LAD1	Progenitor
LARP6	Progenitor
LDHD	Differentiated
LEAP2	Differentiated
LEF1	Progenitor
LGALS1	Progenitor
LIF	Progenitor
LIMK2	Progenitor
LIMS2	Progenitor
LINC00312	Progenitor
LINC00626	Differentiated
LIPC	Differentiated
LLGL2	Differentiated
LMCD1	Progenitor
LMNB1	Progenitor
LMNB2	Progenitor
LOC388282	Progenitor
LRRC31	Differentiated
LUM	Progenitor
LZTS1	Progenitor
MAD2L1	Progenitor
MAP7D2	Progenitor
MAT1A	Differentiated
MCM2	Progenitor
MCM4	Progenitor
MCM5	Progenitor
MDFI	Progenitor
MDGA1	Differentiated
MDK	Progenitor
MELK	Progenitor
MERTK	Differentiated
METTL7B	Differentiated
MEX3D	Differentiated
MFSD2A	Differentiated
MIR503HG	Progenitor
MLKL	Progenitor
MLXIPL	Differentiated
MMD	Progenitor
MMP7	Progenitor
MND1	Progenitor

MOGAT1	Differentiated
MRC2	Progenitor
MSC	Progenitor
MST1	Differentiated
MTFR2	Progenitor
MTHFD1L	Progenitor
MTTP	Differentiated
MUC1	Progenitor
MYADM	Progenitor
MYBL1	Progenitor
MYBPHL	Progenitor
MYCL	Differentiated
NCF2	Progenitor
NCKAP5	Differentiated
NDC80	Progenitor
NDRG1	Differentiated
NEFL	Progenitor
NES	Progenitor
NFE2	Progenitor
NFE2L3	Progenitor
NGEF	Differentiated
NMB	Progenitor
NMU	Progenitor
NOV	Progenitor
NR0B2	Differentiated
NR1H4	Differentiated
NR1I2	Differentiated
NR1I3	Differentiated
NR5A2	Differentiated
NSG1	Progenitor
NUDT7	Differentiated
NUF2	Progenitor
NUSAP1	Progenitor
OCEL1	Differentiated
OLFML2A	Progenitor
OLFML3	Progenitor
ONECUT1	Differentiated
ORC6	Progenitor
ORM1	Differentiated
ORM2	Differentiated
OSBPL10	Progenitor
OTC	Differentiated
OXER1	Differentiated
P3H2	Progenitor
PAGE4	Differentiated
PAH	Differentiated
PAPPA	Progenitor
PAQR9	Differentiated
PBK	Progenitor
PCCA	Differentiated

PCDH7	Progenitor
PCK1	Differentiated
PCK2	Differentiated
PCOLCE	Progenitor
PDE4DIP	Differentiated
PDE5A	Progenitor
PDE6A	Progenitor
PDZD3	Differentiated
PECR	Differentiated
PEG10	Differentiated
PFKFB1	Differentiated
PHF19	Progenitor
PID1	Differentiated
PIF1	Progenitor
PIK3C2G	Differentiated
PIPOX	Differentiated
PITPNM3	Progenitor
PKLR	Differentiated
PKMYT1	Progenitor
PLA2G12B	Differentiated
PLA2G1B	Differentiated
PLAC1	Progenitor
PLAC8	Progenitor
PLAUR	Progenitor
PLEK2	Progenitor
PLEKHG2	Progenitor
PLEKHG4	Progenitor
PLEKHO1	Progenitor
PLG	Differentiated
PLGLB1	Differentiated
PLIN5	Differentiated
PLK2	Progenitor
PLP2	Progenitor
PMEPA1	Progenitor
PML	Progenitor
PON1	Differentiated
PON3	Differentiated
POSTN	Progenitor
PPBP	Progenitor
PPP1R16A	Differentiated
PPP1R18	Progenitor
PPP1R1A	Differentiated
PPP1R3B	Differentiated
PPP1R3C	Differentiated
PRAP1	Differentiated
PRC1	Progenitor
PRKAG2	Differentiated
PROC	Differentiated
PRODH2	Differentiated
PROX1	Differentiated

PRSS23	Progenitor
PSG3	Progenitor
PSG5	Progenitor
PSG8	Progenitor
PTTG1	Progenitor
PTTG2	Progenitor
PTX3	Progenitor
RAB17	Differentiated
RAB3C	Differentiated
RAB7B	Progenitor
RAC2	Progenitor
RACGAP1	Progenitor
RAD51	Progenitor
RAD54L	Progenitor
RASGEF1B	Differentiated
RASSF2	Progenitor
RBP4	Differentiated
RBP5	Differentiated
RDH12	Differentiated
RDH16	Differentiated
RDH5	Differentiated
REEP6	Differentiated
RGN	Differentiated
RHOBTB1	Progenitor
RHOU	Differentiated
RIBC2	Progenitor
RIN1	Progenitor
RMI2	Progenitor
RNF125	Differentiated
RNF152	Differentiated
RNF43	Differentiated
ROR2	Progenitor
RORA	Differentiated
RORC	Differentiated
ROS1	Progenitor
RRAD	Progenitor
RRM2	Progenitor
RUNDC3B	Differentiated
RUNX1T1	Progenitor
RUNX2	Progenitor
S100A11	Progenitor
S100A2	Progenitor
S100A3	Progenitor
S100A4	Progenitor
SAA3P	Differentiated
SAA4	Differentiated
SAMSN1	Progenitor
SCGB1D1	Differentiated
SCGB1D2	Differentiated
SCGN	Differentiated

SCP2	Differentiated
SDS	Differentiated
SELENBP1	Differentiated
SEPHS2	Differentiated
SERPINA1	Differentiated
SERPINA10	Differentiated
SERPINA2	Differentiated
SERPINA3	Differentiated
SERPINA4	Differentiated
SERPINA6	Differentiated
SERPINA7	Differentiated
SERPINC1	Differentiated
SERPIND1	Differentiated
SERPINE1	Progenitor
SERPINF1	Differentiated
SERPINF2	Differentiated
SERPING1	Differentiated
SFN	Progenitor
SHCBP1	Progenitor
SHMT1	Differentiated
SKAP1	Differentiated
SLAMF7	Progenitor
SLC10A1	Differentiated
SLC15A1	Differentiated
SLC16A4	Differentiated
SLC17A3	Differentiated
SLC17A4	Differentiated
SLC22A1	Differentiated
SLC22A18	Differentiated
SLC22A7	Differentiated
SLC22A9	Differentiated
SLC23A1	Differentiated
SLC25A33	Differentiated
SLC25A42	Differentiated
SLC26A2	Progenitor
SLC27A2	Differentiated
SLC27A5	Differentiated
SLC28A1	Differentiated
SLC29A1	Progenitor
SLC2A14	Differentiated
SLC2A2	Differentiated
SLC30A10	Differentiated
SLC37A2	Progenitor
SLC37A4	Differentiated
SLC38A3	Differentiated
SLC39A5	Differentiated
SLC40A1	Differentiated
SLC46A1	Differentiated
SLC47A1	Differentiated
SLC7A1	Progenitor

SLC7A7	Progenitor
SLCO1B1	Differentiated
SLCO2B1	Differentiated
SLIT1	Differentiated
SMLR1	Differentiated
SMOC1	Differentiated
SMPDL3A	Differentiated
SMPX	Differentiated
SNTB1	Differentiated
SORBS1	Differentiated
SORBS2	Differentiated
SORD	Differentiated
SORL1	Differentiated
SOWAHA	Differentiated
SPAG5	Progenitor
SPANXA1	Progenitor
SPANXB1	Progenitor
SPANXC	Progenitor
SPARC	Progenitor
SPARCL1	Differentiated
SPC24	Progenitor
SPC25	Progenitor
SPINK1	Progenitor
SPOCD1	Progenitor
SPP2	Differentiated
SRD5A2	Differentiated
SRGN	Progenitor
SRPX	Progenitor
SRPX2	Progenitor
SRRM3	Progenitor
ST6GAL1	Differentiated
ST7-AS2	Differentiated
STARD10	Differentiated
STK39	Progenitor
STMN1	Progenitor
SULT1C2	Differentiated
SULT2A1	Differentiated
SUN3	Progenitor
SYNC	Progenitor
SYT13	Progenitor
SYT7	Differentiated
SYTL1	Progenitor
SYTL2	Progenitor
TACC3	Progenitor
TAT	Differentiated
TBC1D2	Progenitor
TCEAL5	Progenitor
TCEAL6	Progenitor
TDO2	Differentiated
TDRG1	Differentiated

TEF	Differentiated
TENM2	Progenitor
TF	Differentiated
TGFB2	Progenitor
THSD1	Progenitor
TK1	Progenitor
TM4SF1	Progenitor
TM4SF5	Differentiated
TM7SF2	Differentiated
TMEM132A	Progenitor
TMEM30B	Differentiated
TMPRSS2	Differentiated
TMPRSS6	Differentiated
TMSB15A	Progenitor
TNC	Progenitor
TNFSF10	Differentiated
TNFSF4	Differentiated
TNFSF9	Progenitor
TNNT1	Progenitor
TNS4	Progenitor
TOP2A	Progenitor
TP53I11	Progenitor
TPM1	Progenitor
TPM4	Progenitor
TPX2	Progenitor
TRIML2	Progenitor
TRIP13	Progenitor
TRIP6	Progenitor
TSKU	Differentiated
TSPAN33	Differentiated
TST	Differentiated
TTC39C	Differentiated
TTK	Progenitor
TTPA	Differentiated
TTR	Differentiated
TYMS	Progenitor
UBE2S	Progenitor
UGT2B10	Differentiated
UGT2B11	Differentiated
UGT2B15	Differentiated
UGT2B4	Differentiated
UGT2B7	Differentiated
UHRF1	Progenitor
UNC13D	Progenitor
UNC5CL	Differentiated
UPB1	Differentiated
VDR	Progenitor
VEGFC	Progenitor
VGLL3	Progenitor
VIL1	Differentiated

VNN1	Differentiated
VSNL1	Differentiated
VTN	Differentiated
VWCE	Progenitor
WDHD1	Progenitor
WDR62	Progenitor
WDR66	Progenitor
XDH	Differentiated
ZG16	Differentiated
ZP1	Progenitor
ZWINT	Progenitor

Supplementary Table 2: List of 6115 differentially expressed genes (DEG) significantly deregulated between HepaRG-CSCs and HepaRG differentiated cells using one-way ANOVA test (fold change>1.5; p<0.05)

Gene Symbol	GeneName	FC ([D4] vs [STEM])	FC ([D15] vs [STEM])	FC ([D30] vs [STEM])
A1BG	alpha-1-B glycoprotein	1.2786009	6.696783	3.082248
A1CF	APOBEC1 complementation factor	-4.9775176	1.9234512	3.5627084
A2M	alpha-2-macroglobulin	-3.180162	2.679835	-2.0026248
A4GALT	alpha 1,4-galactosyltransferase	1.7172765	2.330814	2.6973329
AADAC	arylacetamide deacetylase	-15.937277	-1.4010332	-1.0370533
AAED1	AhpC/TSA antioxidant enzyme domain containing 1	-1.6675323	-1.9920148	-3.1165292
AAK1	AP2 associated kinase 1	-1.1745824	-1.036342	-1.9163961
AAK1	AP2 associated kinase 1	3.2605274	1.584849	1.2995273
AAK1	AP2 associated kinase 1	1.7106649	2.26439	2.9384875
AAMDC	adipogenesis associated, Mth938 domain containing	2.0558	2.6811118	3.5350268
AARS	alanyl-tRNA synthetase	-2.3933597	-1.984257	-1.7692832
AARS2	alanyl-tRNA synthetase 2, mitochondrial	-1.5792001	-2.0379038	-2.416757
AASS	aminoadipate-semialdehyde synthase	1.6563952	4.08032	13.742814
AATF	apoptosis antagonizing transcription factor	-1.1702374	-1.29385	-1.644979
AATK	apoptosis-associated tyrosine kinase	2.2825842	2.3721662	2.7492025
ABAT	4-aminobutyrate aminotransferase	1.333954	9.18589	22.985546
ABCA1	ATP-binding cassette, sub-family A (ABC1), member 1	-2.222845	1.6978719	2.1922812
ABCA12	ATP-binding cassette, sub-family A (ABC1), member 12	-2.8449059	-2.906052	-12.632695
ABCA5	ATP-binding cassette, sub-family A (ABC1), member 5	-1.4901389	1.743965	2.6198223
ABCA6	ATP-binding cassette, sub-family A (ABC1), member 6	-6.7583203	6.0547915	4.1084094
ABCA9	ATP-binding cassette, sub-family A (ABC1), member 9	-3.4570377	-2.797449	-2.7594187
ABCB1	ATP-binding cassette, sub-family B (MDR/TAP), member 1	-3.6941428	1.4812688	2.0014675
ABCB1	ATP-binding cassette, sub-family B (MDR/TAP), member 1	-4.498789	1.1424894	1.4550579
ABCB10	ATP-binding cassette, sub-family B (MDR/TAP), member 10	1.2352232	1.252802	1.6817076
ABCB4	ATP-binding cassette, sub-family B (MDR/TAP), member 4	-3.608869	1.5155569	2.5953715
ABCC1	ATP-binding cassette, sub-family C (CFTR/MRP), member 1	-1.2281779	-2.2189157	-3.0993283
ABCC2	ATP-binding cassette, sub-family C (CFTR/MRP), member 2	-2.9670365	-2.2881036	-1.2237419
ABCC3	ATP-binding cassette, sub-family C (CFTR/MRP), member 3	-1.8774179	-1.1885595	-1.0571451
ABCC3	ATP-binding cassette, sub-family C (CFTR/MRP), member 3	-1.4221718	1.2025139	-2.3807018
ABCC5	ATP-binding cassette, sub-family C (CFTR/MRP), member 5	-1.6541861	-2.353647	-1.833562
ABCC5	ATP-binding cassette, sub-family C (CFTR/MRP), member 5	-1.4371359	-1.6934079	-1.2970662
ABCC6	ATP-binding cassette, sub-family C (CFTR/MRP), member 6	-2.9040382	2.177923	3.7622378
ABCC6	ATP-binding cassette, sub-family C (CFTR/MRP), member 6	-1.263604	1.8534063	1.4966877
ABCC6	ATP-binding cassette, sub-family C (CFTR/MRP), member 6	1.4829485	3.8513825	2.9978878
ABCC6	ATP-binding cassette, sub-family C (CFTR/MRP), member 6	1.2340739	2.8536246	2.1797974
ABCC6P1	ATP-binding cassette, sub-family C, member 6 pseudogene 1 (functional)	-1.4418674	3.1724463	12.154898
ABCE1	ATP-binding cassette, sub-family E (OABP), member 1	-1.0616211	-1.4679974	-1.7496217
ABCG5	ATP-binding cassette, sub-family G (WHITE), member 5	-1.2129431	4.760442	17.995886
ABCG8	ATP-binding cassette, sub-family G (WHITE), member 8	-4.176286	17.099655	153.78252
ABHD14A	abhydrolase domain containing 14A	1.498057	1.5361924	1.2632095
ABHD14A	abhydrolase domain containing 14A	1.5648847	1.5429864	1.2748208
ABHD15	abhydrolase domain containing 15	1.7030377	3.0976408	7.780467
ABHD17B	abhydrolase domain containing 17B	1.4865758	1.5695295	1.8076146
ABHD5	abhydrolase domain containing 5	1.2082933	-1.9608085	-1.3933383
ABHD6	abhydrolase domain containing 6	-1.5078272	1.0919743	2.2097433
ABI3	ABI family, member 3	1.2131578	2.273131	7.6346455
ABL1	ABL proto-oncogene 1, non-receptor tyrosine kinase	-1.6066351	-2.1834805	-2.3417368
ABL2	ABL proto-oncogene 2, non-receptor tyrosine kinase	-1.3169973	-2.6276922	-4.249062
ABLIM1	actin binding LIM protein 1	-1.6518356	-1.4572086	-2.171058
ABLIM2	actin binding LIM protein family, member 2	1.8558239	2.0956848	-2.9688423
ABLIM2	actin binding LIM protein family, member 2	3.4482493	4.1270714	3.3688304
ABLIM2	actin binding LIM protein family, member 2	2.319794	2.2049005	1.5771266
ABR	active BCR-related	-1.5321548	-3.3586168	-13.6290655
ABRACL	ABRA C-terminal like	1.40991	1.867791	4.4959846
ABTB1	ankyrin repeat and BTB (POZ) domain containing 1	1.0763471	1.1888098	1.7110468
ACAA1	acetyl-CoA acyltransferase 1	1.271478	3.1971583	7.9950237
ACAA2	acetyl-CoA acyltransferase 2	-1.4275889	1.4783922	2.3278008
ACACB	acetyl-CoA carboxylase beta	-1.1120814	3.328041	4.698888
ACAD10	acyl-CoA dehydrogenase family, member 10	1.9422535	2.4504793	2.952465
ACAD11	acyl-CoA dehydrogenase family, member 11	-1.727451	2.40492	2.9015813
ACADL	acyl-CoA dehydrogenase, long chain	2.3961735	6.676941	4.629465
ACADM	acyl-CoA dehydrogenase, C-4 to C-12 straight chain	-1.0147572	1.6866908	1.7453415
ACADSB	acyl-CoA dehydrogenase, short/branched chain	1.6490353	4.052681	4.2335186
ACADSB	acyl-CoA dehydrogenase, short/branched chain	1.0861697	1.8181634	3.0441396
ACADVL	acyl-CoA dehydrogenase, very long chain	-2.0622897	-1.2955827	-1.2740868
ACAP3	ArfGAP with coiled-coil, ankyrin repeat and PH domains 3	2.2550073	2.3386197	2.650132
ACAT1	acetyl-CoA acetyltransferase 1	-1.0410925	1.6660925	2.5185864
ACAT1	acetyl-CoA acetyltransferase 1	1.1225413	2.0373447	2.8090923
ACAT1	acetyl-CoA acetyltransferase 1	1.3251398	2.378044	3.2797108
ACAT2	acetyl-CoA acetyltransferase 2	1.7324374	1.665208	2.3768902
ACBD4	acyl-CoA binding domain containing 4	-1.5784968	1.8277549	3.5113971
ACBD5	acyl-CoA binding domain containing 5	-1.6369703	-1.2240475	1.0753018
ACBD7	acyl-CoA binding domain containing 7	1.5384132	1.5139436	1.4755657
ACE	angiotensin I converting enzyme	1.5607402	3.127737	6.6577106
ACKR3	atypical chemokine receptor 3	1.3510373	-3.3548105	-7.993189
ACLY	ATP citrate lyase	-1.5081133	-2.3559074	-2.2620344
ACMSD	aminocarboxymuconate semialdehyde decarboxylase	-6.6457276	4.877779	10.649117
ACN9	ACN9 homolog (S. cerevisiae)	1.4476264	1.586429	1.6676809
ACO2	aconitase 2, mitochondrial	1.721572	2.4651947	1.81656
ACOT1	acyl-CoA thioesterase 1	1.8814621	3.4380987	4.732192
ACOT13	acyl-CoA thioesterase 13	1.4471565	1.6315781	2.0099335
ACOT8	acyl-CoA thioesterase 8	1.1304878	1.4469837	1.974724
ACOT9	acyl-CoA thioesterase 9	1.1444561	-1.4184653	-1.9334072
ACOX1	acyl-CoA oxidase 1, palmitoyl	-1.3459187	1.7155309	3.1834233
ACOX2	acyl-CoA oxidase 2, branched chain	-8.015093	3.1900225	9.660336
ACP1	acid phosphatase 1, soluble	-1.0980753	1.0526314	1.547286
ACP1	acid phosphatase 1, soluble	1.0336053	1.5424758	1.4494492
ACSF2	acyl-CoA synthetase family member 2	1.083458	3.0825844	1.5273901
ACSL4	acyl-CoA synthetase long-chain family member 4	-3.2596588	-1.323042	1.1284341
ACSL5	acyl-CoA synthetase long-chain family member 5	-1.2917043	1.1403798	1.9465876
ACSM2B	acyl-CoA synthetase medium-chain family member 2B	-1.3093823	24.234964	398.06964
ACSM3	acyl-CoA synthetase medium-chain family member 3	-1.4296329	4.304538	4.1765437
ACSM5	acyl-CoA synthetase medium-chain family member 5	1.3251327	3.88145	5.89284
ACSM5	acyl-CoA synthetase medium-chain family member 5	1.1877065	13.592159	24.653362
ACSS1	acyl-CoA synthetase short-chain family member 1	3.8952372	29.423874	11.228946
ACSS2	acyl-CoA synthetase short-chain family member 2	1.5900447	2.550701	5.736383
ACTA2	actin, alpha 2, smooth muscle, aorta	1.2033093	-1.5002275	-2.2744143
ACTB	actin, beta	-1.9280261	-1.9965552	-1.912158
ACTBL2	actin, beta-like 2	1.8567322	-1.1053696	-1.3891017
ACTG1	actin gamma 1	1.1655308	-1.4743477	-2.050373
ACTG1P20	actin gamma 1 pseudogene 20	1.6634372	1.0294964	-1.3693979
ACTG1P4	actin gamma 1 pseudogene 4	4.2660975	1.926305	1.3732697
ACTL6A	actin-like 6A	1.0393498	-1.6720682	-1.6910493
ACTL8	actin-like 8	1.3157431	-1.0733608	-3.7045841
ACTL8	actin-like 8	1.7966533	1.2389246	-2.7254882
ACTN1	actinin, alpha 1	-1.3148754	-3.7649302	-6.010054

ACTR1A	ARP1 actin-related protein 1 homolog A, centractin alpha (yeast)	-1.1994493	-1.5848039	-1.4457349
ACTR3	ARP3 actin-related protein 3 homolog (yeast)	1.2086748	-1.5762411	-1.6735636
ACTR3BP5	ACTR3B pseudogene 5	1.95307	2.276555	2.4236166
ACTR6	ARP6 actin-related protein 6 homolog (yeast)	1.5374932	1.1467774	1.5817716
ACY1	aminoacylase 1	1.2611979	2.9436085	2.4796662
ACY3	aspartoacylase (aminocyclase) 3	-1.5956444	2.1484191	1.1534039
ACYP1	acylphosphatase 1, erythrocyte (common) type	-1.187924	-1.5794688	-2.2944734
ACYP2	acylphosphatase 2, muscle type	-1.1683619	1.161427	1.7372142
ADA	adenosine deaminase	1.3238388	-1.2111688	-1.9588927
ADAM10	ADAM metallopeptidase domain 10	1.018843	-1.8288966	-2.05043
ADAM10	ADAM metallopeptidase domain 10	1.4173176	-1.5087981	-1.8420756
ADAM12	ADAM metallopeptidase domain 12	-1.0015488	-7.078487	-9.130632
ADAM17	ADAM metallopeptidase domain 17	-1.6598041	-2.996335	-3.0020785
ADAM33	ADAM metallopeptidase domain 33	1.25294	1.4422891	2.758465
ADAM8	ADAM metallopeptidase domain 8	-1.3612128	-1.3539069	2.0842352
ADAMTS10	ADAM metallopeptidase with thrombospondin type 1 motif, 10	-3.5510633	-1.874378	-13.567153
ADAMTS7	ADAM metallopeptidase with thrombospondin type 1 motif, 7	1.7971759	2.325269	2.562517
ADAMTS7P1	ADAMTS7 pseudogene 1	3.0113025	2.4954417	3.0122237
ADAMTS9	ADAM metallopeptidase with thrombospondin type 1 motif, 9	-1.4307604	-3.971948	-18.831816
ADAMTSL4	ADAMTS-like 4	2.5267286	3.1020458	1.4996151
ADAMTSL4	ADAMTS-like 4	3.0681453	3.7912128	1.5914564
ADARB1	adenosine deaminase, RNA-specific, B1	2.686547	1.7487895	1.7890575
ADARB2	adenosine deaminase, RNA-specific, B2 (non-functional)	-1.729929	-1.1763096	1.861434
ADAT3	adenosine deaminase, tRNA-specific 3	-1.1289207	-1.2281734	-1.5385429
ADCK3	aarF domain containing kinase 3	1.1452706	2.0655456	3.2338276
ADCY3	adenylate cyclase 3	1.1556898	-2.130903	-5.9651055
ADCY4	adenylate cyclase 4	1.8919568	1.7696066	1.0736741
ADH1A	alcohol dehydrogenase 1A (class I), alpha polypeptide	-8.803693	18.448359	50.955265
ADH1B	alcohol dehydrogenase 1B (class I), beta polypeptide	-7.1117177	102.10368	268.52817
ADH1C	alcohol dehydrogenase 1C (class I), gamma polypeptide	-7.311574	11.128555	26.774025
ADH4	alcohol dehydrogenase 4 (class II), pi polypeptide	-3.850678	36.761173	116.45932
ADH5	alcohol dehydrogenase 5 (class III), chi polypeptide	2.3654225	2.3061895	3.0319936
ADH6	alcohol dehydrogenase 6 (class V)	-5.9323883	8.07837	10.629187
ADHFE1	alcohol dehydrogenase, iron containing, 1	-1.2981719	3.7665243	4.595742
ADI1	acireductone dioxygenase 1	1.9789233	2.2073247	3.3980162
ADI1	acireductone dioxygenase 1	2.6947784	2.8016133	3.817145
ADM	adrenomedullin	3.1801124	1.5528904	1.3472918
ADM2	adrenomedullin 2	2.542718	2.4518492	3.2071135
ADNP	activity-dependent neuroprotector homeobox	-1.4590684	-1.6323608	-2.117312
ADORA2B	adenosine A2b receptor	-2.1420321	-3.73596	-8.758946
ADPGK	ADP-dependent glucokinase	-1.6322374	-2.5132816	-2.505266
ADRA1A	adrenoceptor alpha 1A	1.3639425	1.7127512	1.6863935
ADRM1	adhesion regulating molecule 1	-1.5308824	-1.6592406	-2.0491903
ADSSL1	adenylosuccinate synthase like 1	1.5170299	2.2809541	2.451574
AEN	apoptosis enhancing nuclease	-1.1340308	-2.1334794	-3.0897624
AEN	apoptosis enhancing nuclease	-1.2068082	-2.1189604	-3.185028
AES	amino-terminal enhancer of split	1.7229927	1.4650978	1.3921124
AFAP1L1	actin filament associated protein 1-like 1	1.5469346	1.2868143	-2.3660386
AFF4	AF4/FMR2 family, member 4	-1.7074883	-1.7297616	-2.2387533
AFG3L1P	AFG3-like AAA ATPase 1, pseudogene	-1.8637984	-2.1734378	-1.7081387
AFM	afamin	-8.250297	16.369118	17.790264
AFP	alpha-fetoprotein	-1.5117376	-1.3398656	-1.6996315
AFTPH	aftiphilin	-2.023428	-1.4279948	-1.1881772
AGBL3	ATP/GTP binding protein-like 3	1.676267	1.6680382	1.8489499
AGFG2	ArfGAP with FG repeats 2	1.8980787	2.1207683	3.482483
AGL	amylo-alpha-1, 6-glucosidase, 4-alpha-glucanotransferase	1.0499716	2.0077865	2.1653252
AGMAT	agmatine ureohydrolase (agmatinase)	-1.7034587	2.1217735	2.3846803
AGMO	alkylglycerol monooxygenase	-3.7017798	1.6632689	2.5367386
AGO2	argonaute RISC catalytic component 2	-1.3251172	-1.9715014	-2.5325038
AGO3	argonaute RISC catalytic component 3	-1.6154405	-1.6946474	-1.5755364
AGPAT2	1-acylglycerol-3-phosphate O-acyltransferase 2	1.2547829	2.6939404	5.5466595
AGPAT3	1-acylglycerol-3-phosphate O-acyltransferase 3	-1.0283341	1.2397631	1.6384819
AGPAT4	1-acylglycerol-3-phosphate O-acyltransferase 4	-1.2639755	-3.1719043	-5.972678
AGPAT6	1-acylglycerol-3-phosphate O-acyltransferase 6	-1.7254305	-1.93498	-1.616727
AGPAT9	1-acylglycerol-3-phosphate O-acyltransferase 9	-4.77917	-3.9367042	-1.7390082
AGPS	alkylglycerone phosphate synthase	1.0269132	-1.4368702	-2.0146716
AGR2	anterior gradient 2	1.4968114	-1.063614	-21.648403
AGRN	agrin	-2.0166018	-2.5735269	-5.4578915
AGT	angiotensinogen (serpin peptidase inhibitor, clade A, member 8)	-2.0223918	2.2817771	6.1620297
AGTRAP	angiotensin II receptor-associated protein	-1.1820751	-1.4626974	-2.953699
AGXT	alanine-glyoxylate aminotransferase	-3.7205637	16.743004	128.49844
AGXT2	alanine--glyoxylate aminotransferase 2	-1.8432469	40.45593	121.83947
AHCYL2	adenosylhomocysteinase-like 2	1.3313872	1.5267202	2.3909569
AHDC1	AT hook, DNA binding motif, containing 1	1.3548962	2.874723	14.099041
AHNAK	AHNAK nucleoprotein	2.6757398	1.7678872	6.0237045
AHNAK2	AHNAK nucleoprotein 2	-1.7870455	-2.532739	-6.2149267
AHR	aryl hydrocarbon receptor	-1.5795124	-1.7586559	-1.1524302
AHR	aryl hydrocarbon receptor	-2.8305058	-2.9083264	-2.0881174
AHSA2	AHA1, activator of heat shock 90kDa protein ATPase homolog 2 (yeast)	-1.9355361	-2.1539054	-1.4190848
AHSA2	AHA1, activator of heat shock 90kDa protein ATPase homolog 2 (yeast)	4.114577	5.790955	6.1055007
AHSG	alpha-2-HS-glycoprotein	-3.159056	16.082472	6.19099
AIF1L	allograft inflammatory factor 1-like	1.8586005	1.7769458	1.8012856
AIFM1	apoptosis-inducing factor, mitochondrion-associated, 1	1.3349442	2.187906	2.4743752
AIFM2	apoptosis-inducing factor, mitochondrion-associated, 2	1.4927588	1.6260129	1.152884
AIG1	androgen-induced 1	-1.231655	-1.0032794	1.8175025
AIG1	androgen-induced 1	-1.3708866	-1.1126039	1.5969354
AIG1	androgen-induced 1	1.2349579	1.7868758	3.1069102
AIM1	absent in melanoma 1	1.6480216	1.3671795	-1.357833
AIM1L	absent in melanoma 1-like	-2.3185742	-3.0381944	-5.4414144
AJUBA	ajuba LIM protein	2.72299	1.4901127	1.0780534
AK2	adenylate kinase 2	1.2043698	1.5842367	3.0322564
AK2	adenylate kinase 2	1.5604681	1.233399	-1.3402009
AK3	adenylate kinase 3	1.2217463	1.8584489	1.9798638
AK3	adenylate kinase 3	1.1255567	1.5368035	1.7174044
AK4	adenylate kinase 4	-1.0022336	1.2014719	2.2190936
AK4	adenylate kinase 4	1.5054524	1.3866843	2.6896105
AKAP11	A kinase (PRKA) anchor protein 11	-1.5896647	-1.3442941	-1.4813532
AKAP12	A kinase (PRKA) anchor protein 12	-5.3156624	-2.5892515	-3.7185931
AKAP12	A kinase (PRKA) anchor protein 12	-3.157519	-1.8485885	-3.882656
AKAP7	A kinase (PRKA) anchor protein 7	1.4420704	1.9656487	2.2144816
AKAP8L	A kinase (PRKA) anchor protein 8-like	-1.715894	-1.5467763	-1.392869
AKAP9	A kinase (PRKA) anchor protein 9	-2.30638	1.0950937	1.0561497
AKAP9	A kinase (PRKA) anchor protein 9	-3.3671968	-1.2867246	-1.4052043
AKIRIN2	akirin 2	-1.4062961	-2.2167816	-2.2713454
AKR1A1	aldo-keto reductase family 1, member A1 (aldehyde reductase)	-1.1694318	1.513845	1.6112392
AKR1B1	aldo-keto reductase family 1, member B1 (aldose reductase)	-5.1268163	-2.6079576	-1.6720879
AKR1B10	aldo-keto reductase family 1, member B10 (aldose reductase)	-3.2466087	-1.7943634	-1.1433958
AKR1B10	aldo-keto reductase family 1, member B10 (aldose reductase)	-3.7631867	-2.019462	-1.2831584
AKR1C1	aldo-keto reductase family 1, member C1	-1.2720929	1.0405564	2.5769215

AKR1C1	aldo-keto reductase family 1, member C1	-1.0307647	1.190816	2.670361
AKR1C3	aldo-keto reductase family 1, member C3	-1.4937068	-1.1514735	1.899675
AKR1C4	aldo-keto reductase family 1, member C4	-21.241348	3.5829961	4.6501245
AKR1C4	aldo-keto reductase family 1, member C4	1.1565255	1.3594873	4.0683064
AKR7A2	aldo-keto reductase family 7, member A2 (aflatoxin aldehyde reductase)	1.5109032	1.6462008	2.7240062
AKR7A2P1	aldo-keto reductase family 7, member A2 pseudogene 1	1.5265319	4.2614584	4.8546185
AKR7A3	aldo-keto reductase family 7, member A3 (aflatoxin aldehyde reductase)	1.0881422	4.081983	4.5158315
AKR7L	aldo-keto reductase family 7-like (gene/pseudogene)	-1.5044144	4.2861514	4.219417
AKR7L	aldo-keto reductase family 7-like (gene/pseudogene)	1.0621188	4.037889	4.301787
AKT3	v-akt murine thymoma viral oncogene homolog 3	1.3703603	-1.3169926	-1.6459746
ALAD	aminolevulinate dehydratase	1.8588873	3.7163157	7.964023
ALB	albumin	-2.6746626	2.1088548	2.2994921
ALCAM	activated leukocyte cell adhesion molecule	1.4215474	1.1875875	-1.548732
ALDH1A1	aldehyde dehydrogenase 1 family, member A1	-1.0134156	2.3019404	2.6440468
ALDH1L1	aldehyde dehydrogenase 1 family, member L1	1.7468269	8.020346	23.0032
ALDH2	aldehyde dehydrogenase 2 family (mitochondrial)	-2.439912	2.010402	6.0331182
ALDH3A1	aldehyde dehydrogenase 3 family, member A1	-1.7502902	-2.856783	-3.7651803
ALDH3A1	aldehyde dehydrogenase 3 family, member A1	-1.4334686	-2.182452	-2.422192
ALDH3A2	aldehyde dehydrogenase 3 family, member A2	-1.359379	-1.2725681	1.7108719
ALDH3A2	aldehyde dehydrogenase 3 family, member A2	-1.3004544	1.2620456	2.914772
ALDH3B1	aldehyde dehydrogenase 3 family, member B1	1.6178772	-1.0542122	-1.3126996
ALDH4A1	aldehyde dehydrogenase 4 family, member A1	1.4849181	3.770914	4.3607492
ALDH5A1	aldehyde dehydrogenase 5 family, member A1	1.1892146	2.8889637	5.880041
ALDH6A1	aldehyde dehydrogenase 6 family, member A1	-1.0216006	4.882163	9.43362
ALDH7A1	aldehyde dehydrogenase 7 family, member A1	3.8781106	3.9395661	5.73043
ALDH7A1	aldehyde dehydrogenase 7 family, member A1	3.1310039	2.289066	3.3546493
ALDH9A1	aldehyde dehydrogenase 9 family, member A1	1.2663654	1.712206	2.224614
ALDOB	aldolase B, fructose-bisphosphate	-8.020092	43.869236	152.91864
ALDOB	aldolase B, fructose-bisphosphate	-4.2782884	38.597992	155.87209
ALDOC	aldolase C, fructose-bisphosphate	2.075673	3.5668907	3.577603
ALG2	ALG2, alpha-1,3/1,6-mannosyltransferase	1.700305	2.4105952	2.2647574
ALKBH2	alkB, alkylation repair homolog 2 (E. coli)	1.3459389	2.3337157	2.3986614
ALKBH3	alkB, alkylation repair homolog 3 (E. coli)	1.5192282	1.7701504	1.9811321
ALKBH4	alkB, alkylation repair homolog 4 (E. coli)	1.5528779	1.5690781	1.6760706
ALKBH5	AlkB family member 5, RNA demethylase	-1.6101902	-1.6604276	-1.188219
ALKBH7	alkB, alkylation repair homolog 7 (E. coli)	1.6278114	1.7805519	1.5746411
ALMS1	Alstrom syndrome 1	-1.4567145	-1.9450436	-2.1645262
ALOX12-AS1	ALOX12 antisense RNA 1	2.016708	2.4104154	5.02918
ALOX5	arachidonate 5-lipoxygenase	1.7999985	-1.5639484	-9.092216
ALPK1	alpha-kinase 1	-2.3831472	-1.9325936	-1.681895
ALPP	alkaline phosphatase, placental	8.307917	1.2444484	-1.1106429
ALPPL2	alkaline phosphatase, placental-like 2	1.5422144	2.1931489	2.7262692
AMACR	alpha-methylacyl-CoA racemase	-1.0789595	1.2414033	2.2916017
AMBP	alpha-1-microglobulin/bikunin precursor	-4.4336786	2.4368927	6.665854
AMD1	adenosylmethionine decarboxylase 1	-1.0529928	-1.6556292	-1.6254746
AMDHD1	amidohydrolase domain containing 1	-3.3982198	4.047218	5.385422
AMDHD2	amidohydrolase domain containing 2	-1.3177862	-1.4698032	-2.0579655
AMFR	autocrine motility factor receptor, E3 ubiquitin protein ligase	-1.1543144	-1.051535	1.7153941
AMMECR1	Alport syndrome, mental retardation, midface hypoplasia and elliptocytosis chromosomal regi	-1.1878674	-1.5624174	-1.5191685
AMN	amnion associated transmembrane protein	5.018976	5.9917502	7.932501
AMOTL1	angiomotin like 1	1.5905445	2.139502	2.622969
AMOTL2	angiomotin like 2	4.9567895	3.1165802	3.002038
AMPD2	adenosine monophosphate deaminase 2	-1.3468081	-1.7085434	-2.6511676
AMPH	amphiphysin	1.9035383	1.0173626	-2.776131
AMTN	amelotin	-1.2912514	-75.0695	-466.6878
AMY1C	amylase, alpha 1C (salivary)	-1.9085245	2.622761	2.5388322
ANAPC11	anaphase promoting complex subunit 11	1.4225285	1.3119658	1.7090651
ANAPC16	anaphase promoting complex subunit 16	1.5573083	2.178369	2.1687715
ANG	angiogenin, ribonuclease, RNase A family, 5	-1.6837033	3.8117633	5.048697
ANGPT1	angiopoietin 1	3.1836371	2.1408129	1.0640377
ANGPTL3	angiopoietin-like 3	-1.8005049	13.606026	12.468468
ANGPTL4	angiopoietin-like 4	-11.565647	-9.579512	-8.662375
ANK1	ankyrin 1, erythrocytic	-1.3149115	-3.9679008	-12.417973
ANK3	ankyrin 3, node of Ranvier (ankyrin G)	-1.2984257	2.2894678	1.1786617
ANKHD1-EIF4EBP3	ANKHD1-EIF4EBP3 readthrough	-1.5417407	1.1743882	1.0339322
ANKLE2	ankyrin repeat and LEM domain containing 2	-1.4974631	-2.6353264	-3.8659194
ANKMY1	ankyrin repeat and MYND domain containing 1	-1.2219504	1.1025659	1.6330383
ANKRA2	ankyrin repeat, family A (RFXANK-like), 2	-1.7196983	-2.0526793	-1.5463815
ANKRD11	ankyrin repeat domain 11	-1.494749	-1.9128561	-1.8862888
ANKRD11	ankyrin repeat domain 11	-2.135507	-2.4011142	-2.33645
ANKRD12	ankyrin repeat domain 12	-2.380729	-1.4206436	-1.1066926
ANKRD12	ankyrin repeat domain 12	-3.1081152	-1.6766133	-1.4856709
ANKRD13B	ankyrin repeat domain 13B	1.5904068	1.678503	2.0607781
ANKRD16	ankyrin repeat domain 16	2.0020413	2.43135	1.8994173
ANKRD18A	ankyrin repeat domain 18A	1.4346049	1.0340055	-1.9977472
ANKRD18B	ankyrin repeat domain 18B	-1.2785057	-1.7373315	-3.4953568
ANKRD2	ankyrin repeat domain 2 (stretch responsive muscle)	8.392012	6.7652674	2.879989
ANKRD20A11P	ankyrin repeat domain 20 family, member A11, pseudogene	-1.6390827	-1.8774056	-5.264535
ANKRD20A12P	ankyrin repeat domain 20 family, member A12, pseudogene	-1.5553066	-1.6257457	-3.594197
ANKRD20A12P	ankyrin repeat domain 20 family, member A12, pseudogene	-1.0984738	-1.4515402	-3.1852846
ANKRD20A12P	ankyrin repeat domain 20 family, member A12, pseudogene	-1.243669	-1.6527784	-3.3477752
ANKRD20A12P	ankyrin repeat domain 20 family, member A12, pseudogene	-1.2828505	-1.4468368	-3.3296354
ANKRD20A2	ankyrin repeat domain 20 family, member A2	-3.1598566	-2.511825	-1.9241499
ANKRD20A5P	ankyrin repeat domain 20 family, member A5, pseudogene	-2.8450537	-2.4392307	-1.9096181
ANKRD20A8P	ankyrin repeat domain 20 family, member A8, pseudogene	-1.2745675	-1.6324795	-3.3958545
ANKRD28	ankyrin repeat domain 28	1.5811049	1.2167075	-2.9829292
ANKRD29	ankyrin repeat domain 29	1.0285242	1.3792173	2.858955
ANKRD32	ankyrin repeat domain 32	1.6010725	-1.0827678	-1.4931812
ANKRD33	ankyrin repeat domain 33	1.4587227	1.7215108	2.019595
ANKRD33B	ankyrin repeat domain 33B	2.0147963	1.4427048	-2.5519364
ANKRD36	ankyrin repeat domain 36	-3.1324556	-5.937929	-4.985544
ANKRD36BP2	ankyrin repeat domain 36B pseudogene 2	-2.9740753	-4.511525	-4.4604487
ANKRD37	ankyrin repeat domain 37	1.2714621	1.3792437	2.079955
ANKRD39	ankyrin repeat domain 39	1.3365129	1.3800373	1.9975863
ANKRD46	ankyrin repeat domain 46	1.4461313	2.0925388	5.960294
ANKRD52	ankyrin repeat domain 52	1.2335614	-1.4422395	-1.6013131
ANKS6	ankyrin repeat and sterile alpha motif domain containing 6	1.502348	1.3456111	1.090492
ANLN	anillin, actin binding protein	1.6450806	-10.432414	-8.734361
ANP32AP1	acidic (leucine-rich) nuclear phosphoprotein 32 family, member A pseudogene 1	1.8443354	1.8585526	2.2600849
ANP32B	acidic (leucine-rich) nuclear phosphoprotein 32 family, member B	1.3356949	-1.1354328	-1.5434434
ANP32E	acidic (leucine-rich) nuclear phosphoprotein 32 family, member E	1.9973524	-1.5772774	-1.7082267
ANP32E	acidic (leucine-rich) nuclear phosphoprotein 32 family, member E	1.7044601	-1.4594198	-1.4514685
ANTXR2	anthrax toxin receptor 2	-2.0369515	-1.3086833	1.4784269
ANXA1	annexin A1	-1.0280036	-2.5121245	-3.0218494
ANXA10	annexin A10	-31.45192	1.1793272	1.0535995
ANXA13	annexin A13	-2.868386	4.01271	1.8578879
ANXA2	annexin A2	2.5977492	1.2366685	1.1956024
ANXA2	annexin A2	1.1058826	-1.6669964	-1.9277228
ANXA2P1	annexin A2 pseudogene 1	-1.053029	-2.1566706	-2.6784616

ANXA2P3	annexin A2 pseudogene 3	1.7621622	-1.1464558	-1.4122096
ANXA3	annexin A3	2.3295007	-1.3512322	-6.0439496
ANXA4	annexin A4	-1.3561265	-1.5127488	-2.3512042
ANXA5	annexin A5	-1.1894352	-1.7237493	-2.2362347
ANXA7	annexin A7	1.25573	1.5740821	2.5132754
ANXA8L1	annexin A8-like 1	2.3464034	1.3273767	-5.6681423
ANXA9	annexin A9	-1.5948303	2.4822443	2.8911016
AOX1	aldehyde oxidase 1	1.4042792	3.3403444	13.210667
AOX1	aldehyde oxidase 1	1.3050723	2.7546968	9.569326
AP1G2	adaptor-related protein complex 1, gamma 2 subunit	-1.894273	-1.8084654	-1.8839436
AP1S3	adaptor-related protein complex 1, sigma 3 subunit	1.7971348	1.4328812	1.274493
AP3D1	adaptor-related protein complex 3, delta 1 subunit	-1.4730471	-1.4823194	-1.7594922
AP3S1	adaptor-related protein complex 3, sigma 1 subunit	-1.128568	-1.7710049	-1.22587
AP3S2	adaptor-related protein complex 3, sigma 2 subunit	-1.0510871	1.0326387	1.6005623
AP4E1	adaptor-related protein complex 4, epsilon 1 subunit	1.0734122	-1.6440123	-1.4304518
APBB1IP	amyloid beta (A4) precursor protein-binding, family B, member 1 interacting protein	1.3257413	1.6331316	1.6376301
APBB2	amyloid beta (A4) precursor protein-binding, family B, member 2	1.8163509	2.0631077	1.7966291
APCDD1L	adenomatosis polyposis coli down-regulated 1-like	-3.165962	-36.22188	-160.42987
APCS	amyloid P component, serum	3.0519047	7.9809847	9.366784
APEX1	APEX nuclease (multifunctional DNA repair enzyme) 1	-1.1005639	-1.1125984	-1.7460232
APH1B	APH1B gamma secretase subunit	1.0290364	-1.4973079	-3.3599398
APMAP	adipocyte plasma membrane associated protein	-1.186502	2.5359232	2.4803302
APOA1	apolipoprotein A-I	-2.3692513	16.439596	56.584885
APOA1	apolipoprotein A-I	-2.0490155	17.027348	56.58006
APOA1BP	apolipoprotein A-I binding protein	2.0661614	2.199758	2.348483
APOA1BP	apolipoprotein A-I binding protein	1.3715993	1.5569707	1.5649813
APOA2	apolipoprotein A-II	-24.31277	1.8637182	6.8323607
APOA5	apolipoprotein A-V	-1.5656894	3.6639953	41.474117
APOB	apolipoprotein B	-1.8539908	3.3461509	4.0411043
APOBEC3B	apolipoprotein B mRNA editing enzyme, catalytic polypeptide-like 3B	1.1314266	-3.4989026	-4.3607078
APOBEC3C	apolipoprotein B mRNA editing enzyme, catalytic polypeptide-like 3C	1.3978108	-1.2260814	-4.1973414
APOBEC3F	apolipoprotein B mRNA editing enzyme, catalytic polypeptide-like 3F	1.3056328	-1.3154647	-4.502614
APOC1	apolipoprotein C-I	-3.079025	3.2294571	2.8523648
APOC3	apolipoprotein C-III	-1.5785103	26.533472	265.57626
APOE	apolipoprotein E	-2.8163073	1.489034	1.3586937
APOH	apolipoprotein H (beta-2-glycoprotein I)	-2.855485	6.409233	8.876256
APOM	apolipoprotein M	1.1983263	4.8274965	9.843292
APOM	apolipoprotein M	1.1550828	4.9203286	9.922535
APOO	apolipoprotein O	1.4814157	1.5690249	1.125856
APOOL	apolipoprotein O-like	1.5213124	1.4618108	1.9029903
APP	amyloid beta (A4) precursor protein	-1.2584414	-1.5420531	-1.9379587
APPL2	adaptor protein, phosphotyrosine interaction, PH domain and leucine zipper containing 2	-1.7118641	1.2770377	1.3093643
APTR	Alu-mediated CDKN1A/p21 transcriptional regulator (non-protein coding)	1.1592556	2.1696362	2.9179432
APTR	Alu-mediated CDKN1A/p21 transcriptional regulator (non-protein coding)	1.3535794	2.3364296	3.2513983
AQP11	aquaporin 11	-2.0450206	1.4047664	6.7619734
AQP3	aquaporin 3 (Gill blood group)	-9.61101	1.0772517	1.2858158
AQP5	aquaporin 5	2.3225033	2.3153627	2.5135882
AQP7	aquaporin 7	-10.994272	4.051539	6.4337587
AQP7P1	aquaporin 7 pseudogene 1	-4.7498693	3.4780486	4.617128
AQP7P3	aquaporin 7 pseudogene 3	-9.09414	5.334696	8.903953
AQP9	aquaporin 9	-3.1505957	3.1232114	21.683743
AR	androgen receptor	-1.0417614	1.6561494	3.9295518
ARAFP2	ARAF pseudogene 2	1.7592512	2.0215802	2.3208134
ARAP2	ArfGAP with RhoGAP domain, ankyrin repeat and PH domain 2	-1.7606776	-2.5430756	-2.9257262
ARCN1	archain 1	-1.7078952	-1.8239675	-1.8536372
AREG	amphiregulin	-2.433822	-4.1850696	-9.727512
AREG	amphiregulin	-2.0961854	-4.0830717	-9.333011
ARF5	ADP-ribosylation factor 5	1.3643278	1.3044605	1.7071382
ARFGAP1	ADP-ribosylation factor GTPase activating protein 1	-1.4598746	-2.231954	-2.6509373
ARFGAP2	ADP-ribosylation factor GTPase activating protein 2	1.2397201	1.5010488	1.9070603
ARFGAP3	ADP-ribosylation factor GTPase activating protein 3	-1.877208	-1.7190211	-1.7202528
ARFRP1	ADP-ribosylation factor related protein 1	1.4954051	1.4027016	2.522807
ARFRP1	ADP-ribosylation factor related protein 1	1.3903221	1.508954	1.2948701
ARG1	arginase 1	-4.3230014	93.9467	648.9471
ARG1	arginase 1	-7.281323	44.518883	316.6335
ARHGAP18	Rho GTPase activating protein 18	1.2476733	1.7090299	-1.1955932
ARHGAP28	Rho GTPase activating protein 28	1.2503314	-1.1106865	-3.1791892
ARHGAP33	Rho GTPase activating protein 33	1.598273	1.8031765	2.1670463
ARHGAP35	Rho GTPase activating protein 35	-1.6546339	-1.4624194	-1.2248908
ARHGAP4	Rho GTPase activating protein 4	-1.499678	1.3257623	2.2885408
ARHGAP42	Rho GTPase activating protein 42	1.2503788	-1.0876182	-1.5625687
ARHGAP42	Rho GTPase activating protein 42	1.8368002	1.9572192	1.7639924
ARHGAP44	Rho GTPase activating protein 44	1.1550947	2.3434622	2.2213926
ARHGAP5	Rho GTPase activating protein 5	-1.4268557	-1.456737	-1.6921208
ARHGAP5-AS1	ARHGAP5 antisense RNA 1 (head to head)	1.6757926	2.0311506	1.473577
ARHGDIB	Rho GDP dissociation inhibitor (GDI) beta	1.5952954	-1.1193575	-6.9337087
ARHGEF10L	Rho guanine nucleotide exchange factor (GEF) 10-like	-1.5716091	1.615487	2.3963938
ARHGEF10L	Rho guanine nucleotide exchange factor (GEF) 10-like	-1.9621601	1.338845	2.0716765
ARHGEF18	Rho/Rac guanine nucleotide exchange factor (GEF) 18	1.8972507	2.1685975	1.3922296
ARHGEF5	Rho guanine nucleotide exchange factor (GEF) 5	-1.6239213	-2.0449913	-2.1910636
ARID1B	AT rich interactive domain 1B (SWI1-like)	2.3383079	1.9902128	2.4308276
ARID5A	AT rich interactive domain 5A (MRF1-like)	-1.3072252	1.9682376	1.098672
ARID5B	AT rich interactive domain 5B (MRF1-like)	-1.1872963	-1.8726101	-3.0282595
ARIH2	ariadne RBR E3 ubiquitin protein ligase 2	-1.1667782	-1.5117877	-1.4862423
ARL14EP	ADP-ribosylation factor-like 14 effector protein	1.3202726	1.8021234	1.5394162
ARL2BP	ADP-ribosylation factor-like 2 binding protein	1.6538824	1.9389434	2.128139
ARL4A	ADP-ribosylation factor-like 4A	-1.5776085	-1.8054343	-1.6626676
ARL4C	ADP-ribosylation factor-like 4C	-3.881174	-8.119221	-5.073727
ARL4D	ADP-ribosylation factor-like 4D	2.6788714	3.51382	9.579114
ARL6IP6	ADP-ribosylation factor-like 6 interacting protein 6	1.0804352	-2.0392325	-2.0701597
ARMC5	armadillo repeat containing 5	1.8266348	1.8863003	1.8491764
ARMC8	armadillo repeat containing 8	1.6807187	1.2566122	1.0843701
ARMC9	armadillo repeat containing 9	1.1713306	-3.2932107	-5.2807593
ARMC9	armadillo repeat containing 9	-1.0483849	-4.184323	-7.5845323
ARMCX5	armadillo repeat containing, X-linked 5	1.2296896	1.6008568	1.4174205
ARNT2	aryl-hydrocarbon receptor nuclear translocator 2	1.7082906	-1.6620879	-4.179019
ARNTL	aryl hydrocarbon receptor nuclear translocator-like	1.1956501	1.1396103	1.6594889
ARPC1B	actin related protein 2/3 complex, subunit 1B, 41kDa	-1.4447855	-1.8660967	-2.145138
ARPC2	actin related protein 2/3 complex, subunit 2, 34kDa	1.0456058	-1.4471079	-1.7902919
ARPC2	actin related protein 2/3 complex, subunit 2, 34kDa	1.5030767	1.0316613	-1.2607508
ARPC5	actin related protein 2/3 complex, subunit 5, 16kDa	1.6616693	-1.0381906	-1.1288484
ARPC5	actin related protein 2/3 complex, subunit 5, 16kDa	1.9939774	1.5520549	1.0582342
ARPIN	actin-related protein 2/3 complex inhibitor	1.5000353	-1.0294152	-1.5165777
ARRB2	arrestin, beta 2	1.5639484	2.6559927	3.5365272
ARRDC2	arrestin domain containing 2	1.6442001	2.106403	1.6953067
ARRDC3	arrestin domain containing 3	-1.4068627	2.6366384	6.9503803
ARSD	arylsulfatase D	1.2237715	1.8220767	2.4343228
ARSE	arylsulfatase E (chondrodysplasia punctata 1)	-2.0939584	2.2443788	1.3243437
ARV1	ARV1 homolog (S. cerevisiae)	1.5286982	1.6835399	1.6757892

ASAP1	ArfGAP with SH3 domain, ankyrin repeat and PH domain 1	1.0720403	-2.7297263	-10.576096
ASAP2	ArfGAP with SH3 domain, ankyrin repeat and PH domain 2	-1.1208892	-2.1845555	-1.9487622
ASB1	ankyrin repeat and SOCS box containing 1	-1.0210798	-1.2829726	-1.7267334
ASB10	ankyrin repeat and SOCS box containing 10	2.0922728	1.2846382	1.020873
ASB13	ankyrin repeat and SOCS box containing 13	1.5407234	5.659738	13.010261
ASB9	ankyrin repeat and SOCS box containing 9	2.9737933	2.3885913	2.5911567
ASCC3	activating signal cointegrator 1 complex subunit 3	-1.2805343	-2.0027277	-3.1846416
ASF1B	anti-silencing function 1B histone chaperone	1.3313949	-3.4927497	-3.4813085
ASGR1	asialoglycoprotein receptor 1	-9.295188	5.299228	9.810863
ASGR2	asialoglycoprotein receptor 2	-3.0836434	2.0647435	2.4569976
ASIC4	acid sensing (proton gated) ion channel family member 4	1.7477982	1.8264091	4.9033318
ASL	argininosuccinate lyase	-1.3505625	2.0136006	2.3011398
ASNS	asparagine synthetase (glutamine-hydrolyzing)	-2.1195712	-3.611371	-2.8014936
ASPA	aspartoacylase	-1.3846645	-1.1912315	-3.0191212
ASPH	aspartate beta-hydroxylase	-2.2765265	-3.2420797	-3.7300503
ASPH	aspartate beta-hydroxylase	-3.0577536	-3.313233	-3.4619632
ASPHD1	aspartate beta-hydroxylase domain containing 1	-1.1183393	-1.6015702	-2.953485
ASPM	asp (abnormal spindle) homolog, microcephaly associated (Drosophila)	1.6084728	-1.4759939	-3.9612935
ASS1	argininosuccinate synthase 1	1.166819	1.7998244	2.0579207
ASS1	argininosuccinate synthase 1	1.7297617	2.5223367	3.059157
ASTN2	astrotactin 2	2.3244977	-1.0400575	-1.3460734
ASXL1	additional sex combs like transcriptional regulator 1	1.710599	-1.1389751	-1.2889298
ATAD3B	ATPase family, AAA domain containing 3B	-1.999274	-2.1996453	-2.9283233
ATF3	activating transcription factor 3	-7.57439	-8.388177	-3.6471493
ATF3	activating transcription factor 3	1.5077863	2.12654	1.82518
ATG12	autophagy related 12	-1.1133432	-1.6089642	-1.2023166
ATG16L2	autophagy related 16-like 2 (S. cerevisiae)	-2.18165	-2.7046247	-2.3879101
ATG2A	autophagy related 2A	-1.7395723	-1.4033898	-1.2020831
ATG4A	autophagy related 4A, cysteine peptidase	-1.2599808	1.0107027	1.8655047
ATG4B	autophagy related 4B, cysteine peptidase	1.1698102	1.3245344	1.6121339
ATG4B	autophagy related 4B, cysteine peptidase	1.6187477	1.658182	2.2037158
ATG5	autophagy related 5	1.5871725	1.3553785	1.6727338
ATHL1	ATH1, acid trehalase-like 1 (yeast)	-2.3025806	-1.9027303	-4.7852964
ATL2	atlastin GTPase 2	-1.3981389	-1.7218667	-1.0242019
ATOH8	atonal homolog 8 (Drosophila)	2.064297	3.0649703	3.4437158
ATP10B	ATPase, class V, type 10B	2.3786566	1.5748334	1.3065836
ATP13A1	ATPase type 13A1	-1.4972368	-1.294202	-1.8878534
ATP13A2	ATPase type 13A2	-1.7444942	-2.3421295	-3.4515784
ATP1A2	ATPase, Na+/K+ transporting, alpha 2 polypeptide	3.6989446	3.5358098	4.1612716
ATP1A3	ATPase, Na+/K+ transporting, alpha 3 polypeptide	2.019345	2.1162276	2.097877
ATP1A4	ATPase, Na+/K+ transporting, alpha 4 polypeptide	1.6741406	3.0688572	1.5032252
ATP1B1	ATPase, Na+/K+ transporting, beta 1 polypeptide	3.4006712	3.2853196	2.0063558
ATP1B3	ATPase, Na+/K+ transporting, beta 3 polypeptide	1.1779066	-1.8822311	-1.4561504
ATP2A2	ATPase, Ca++ transporting, cardiac muscle, slow twitch 2	-1.72344	-1.7948916	-1.7894998
ATP2B1	ATPase, Ca++ transporting, plasma membrane 1	-1.3502337	-2.9920793	-3.016952
ATP2B4	ATPase, Ca++ transporting, plasma membrane 4	-1.1592956	-1.7292869	-3.0454934
ATP5A1	ATP synthase, H+ transporting, mitochondrial F1 complex, alpha subunit 1, cardiac muscle	1.5018917	1.882095	1.435731
ATP5F1	ATP synthase, H+ transporting, mitochondrial Fo complex, subunit B1	1.6504445	1.8128852	1.4252069
ATP5G1	ATP synthase, H+ transporting, mitochondrial Fo complex, subunit C1 (subunit 9)	1.9301225	2.1388786	1.2591932
ATP5G2	ATP synthase, H+ transporting, mitochondrial Fo complex, subunit C2 (subunit 9)	1.4657129	1.657883	1.4036008
ATP5G3	ATP synthase, H+ transporting, mitochondrial Fo complex, subunit C3 (subunit 9)	2.3777559	2.7906194	2.2634132
ATP5H	ATP synthase, H+ transporting, mitochondrial Fo complex, subunit d	1.8436786	2.2114332	1.7686856
ATP5I	ATP synthase, H+ transporting, mitochondrial Fo complex, subunit E	1.3370707	1.6072426	1.5276098
ATP5J	ATP synthase, H+ transporting, mitochondrial Fo complex, subunit F6	1.9313837	2.6153843	2.1511357
ATP5L	ATP synthase, H+ transporting, mitochondrial Fo complex, subunit G	1.7880363	1.6618438	1.4504154
ATP5L2	ATP synthase, H+ transporting, mitochondrial Fo complex, subunit G2	1.9198775	1.7699815	1.5086944
ATP5O	ATP synthase, H+ transporting, mitochondrial F1 complex, O subunit	1.7594037	1.9739684	1.4933243
ATP6AP1L	ATPase, H+ transporting, lysosomal accessory protein 1-like	-1.4085096	1.3953265	2.1803522
ATP6V0A1	ATPase, H+ transporting, lysosomal V0 subunit a1	-1.6949912	-1.1708162	1.4035031
ATP6V0C	ATPase, H+ transporting, lysosomal 16kDa, V0 subunit c	1.4409634	1.2691715	1.7374345
ATP6V0E2	ATPase, H+ transporting V0 subunit e2	1.1465988	1.9601855	5.369996
ATP6V0E2-AS1	ATP6V0E2 antisense RNA 1	1.9373573	2.3668394	1.4914287
ATP6V1D	ATPase, H+ transporting, lysosomal 34kDa, V1 subunit D	1.5043398	1.6081305	1.4593359
ATP7A	ATPase, Cu++ transporting, alpha polypeptide	2.0009258	2.203886	2.9048176
ATP7B	ATPase, Cu++ transporting, beta polypeptide	-1.2175279	1.3072656	2.7697933
ATP8B1	ATPase, aminophospholipid transporter, class I, type 8B, member 1	1.2660141	-1.0105499	1.6649753
ATPAF2	ATP synthase mitochondrial F1 complex assembly factor 2	-1.0891109	1.0989087	1.7732143
ATPIF1	ATPase inhibitory factor 1	1.7455308	1.8727447	2.772885
ATPIF1	ATPase inhibitory factor 1	1.3049488	1.2271638	2.1044626
ATR	ATR serine/threonine kinase	-1.2760819	-1.3608198	-2.0718467
ATRX	alpha thalassemia/mental retardation syndrome X-linked	-1.5280291	-1.4372905	-1.9487969
ATRX	alpha thalassemia/mental retardation syndrome X-linked	-2.0299153	-2.2776797	-2.5221179
ATXN10	ataxin 10	2.384937	2.001293	1.4887174
ATXN1L	ataxin 1-like	1.6898497	1.6754754	1.723008
ATXN2L	ataxin 2-like	-1.0823547	1.3141443	1.627047
ATXN7L2	ataxin 7-like 2	2.268663	2.6469338	2.8796418
AUH	AU RNA binding protein/enoyl-CoA hydratase	1.2433941	2.271052	2.9068646
AUNIP	aurora kinase A and ninein interacting protein	3.1447697	-1.7125992	-2.447008
AUP1	ancient ubiquitous protein 1	-1.5838528	-1.3994365	-1.2486384
AURKA	aurora kinase A	2.457887	-1.7983396	-2.7780957
AURKAPS1	aurora kinase A pseudogene 1	1.4648163	-2.4813204	-3.3462849
AVPI1	arginine vasopressin-induced 1	2.0246954	1.6584002	1.4127282
AXL	AXL receptor tyrosine kinase	1.3223343	-1.4925653	-2.324291
AZGP1	alpha-2-glycoprotein 1, zinc-binding	-2.0815036	7.002967	21.62459
AZGP1P1	alpha-2-glycoprotein 1, zinc-binding pseudogene 1	-1.5593653	8.190198	26.586302
AZI2	5-azacytidine induced 2	1.7529604	1.8583945	1.7109407
AZIN1	antizyme inhibitor 1	1.6438186	1.3375189	1.8338239
B3GALNT2	beta-1,3-N-acetylgalactosaminyltransferase 2	-1.2839816	-1.6792552	-1.7248152
B3GALT5-AS1	B3GALT5 antisense RNA 1	1.8055762	2.1469557	2.866698
B3GNT3	UDP-GlcNAc:betaGal beta-1,3-N-acetylglucosaminyltransferase 3	-1.5059144	1.0458765	-2.4147322
B3GNT5	UDP-GlcNAc:betaGal beta-1,3-N-acetylglucosaminyltransferase 5	-1.4464531	-2.940971	-1.9616954
B3GNT7	UDP-GlcNAc:betaGal beta-1,3-N-acetylglucosaminyltransferase 7	1.9689891	1.8562505	2.0817285
B4GALNT1	beta-1,4-N-acetyl-galactosaminyl transferase 1	-1.472813	-2.6111825	-5.3920817
B4GALT2	UDP-Gal:betaGlcNAc beta 1,4- galactosyltransferase, polypeptide 2	1.0684725	-1.2435926	-1.8855245
B4GALT3	UDP-Gal:betaGlcNAc beta 1,4- galactosyltransferase, polypeptide 3	-1.1960773	-1.840242	-2.0725508
B4GALT4	UDP-Gal:betaGlcNAc beta 1,4- galactosyltransferase, polypeptide 4	-1.4733338	-1.9138323	-1.0849565
B4GALT5	UDP-Gal:betaGlcNAc beta 1,4- galactosyltransferase, polypeptide 5	-2.0649037	-2.198318	-2.3268154
B4GAT1	beta-1,4-glucuronyltransferase 1	-1.0057797	1.3673053	2.1633043
B9D1	B9 protein domain 1	-1.0612859	-1.3980559	-1.9768244
B9D2	B9 protein domain 2	-1.0459044	-1.3102634	-1.8407212
BAAT	bile acid CoA:amino acid N-acyltransferase	-2.9843745	3.833065	10.562282
BACE1	beta-site APP-cleaving enzyme 1	1.1099222	-1.1469033	1.5451524
BACE1	beta-site APP-cleaving enzyme 1	1.1311265	-1.1016079	1.7376946
BACE2	beta-site APP-cleaving enzyme 2	-1.5197424	-1.7530708	-2.9423628
BACH1	BTB and CNC homology 1, basic leucine zipper transcription factor 1	-1.9069232	-1.3238218	-1.3615762
BAG1	BCL2-associated athanogene	1.4843554	2.3135507	1.7142184
BAG1	BCL2-associated athanogene	1.6435363	2.632785	1.9285253
BAG1	BCL2-associated athanogene	1.5667748	2.4812438	1.8305689
BAG2	BCL2-associated athanogene 2	1.8549558	1.1764907	-1.406729

BAG3	BCL2-associated athanogene 3	1.5166827	1.0149832	-1.284688
BAI2	brain-specific angiogenesis inhibitor 2	-3.2407653	-8.544206	-10.387793
BAIAP2	BAI1-associated protein 2	-1.163615	-2.2922482	-1.992065
BAIAP2-AS1	BAIAP2 antisense RNA 1 (head to head)	1.331994	1.5587623	2.1559901
BAIAP2L1	BAI1-associated protein 2-like 1	-1.1624238	-1.5572172	-1.9622697
BAIAP2L2	BAI1-associated protein 2-like 2	-4.125822	1.0133226	-8.629275
BAK1	BCL2-antagonist/killer 1	1.286388	1.1350017	-1.7101953
BAMBI	BMP and activin membrane-bound inhibitor	-1.27244	1.5702542	3.3972135
BANP	BTG3 associated nuclear protein	1.1815015	1.4876441	1.5346775
BARD1	BRCA1 associated RING domain 1	1.5374086	-2.083146	-2.487652
BATF2	basic leucine zipper transcription factor, ATF-like 2	-1.0073273	2.219724	-1.0378097
BAX	BCL2-associated X protein	1.2436337	-1.4938611	-2.9286737
BAZ1A	bromodomain adjacent to zinc finger domain, 1A	-1.414201	-2.5941348	-3.3909574
BAZ1B	bromodomain adjacent to zinc finger domain, 1B	-1.2551575	-1.6373973	-1.4771987
BBC3	BCL2 binding component 3	-1.0676275	-1.2572967	-1.90089
BBIP1	BBSome interacting protein 1	1.0168099	-1.5026188	-1.1044303
BBOX1	butyrobetaine (gamma), 2-oxoglutarate dioxygenase (gamma-butyrobetaine hydroxylase) 1	-9.738895	-1.0154619	8.221461
BBS10	Bardet-Biedl syndrome 10	1.8174078	4.438438	3.5249357
BBS4	Bardet-Biedl syndrome 4	-1.4552292	-1.5038158	-1.2157583
BBS5	Bardet-Biedl syndrome 5	-2.31388	-1.6634847	-1.8213788
BBS9	Bardet-Biedl syndrome 9	-1.1937201	-1.6243613	-1.0586095
BBX	bobby sox homolog (Drosophila)	-1.1970797	-1.5929884	-1.5206324
BBX	bobby sox homolog (Drosophila)	-1.5519321	-1.9544486	-1.8964742
BCAT2	branched chain amino-acid transaminase 2, mitochondrial	1.3139956	2.2001815	3.0260258
BCDIN3D	BCDIN3 domain containing	1.3766408	1.6261079	2.3324022
BCHE	butyrylcholinesterase	1.469316	1.8584902	2.9645903
BCKDHA	branched chain keto acid dehydrogenase E1, alpha polypeptide	1.1153071	1.880867	1.7385201
BCKDHB	branched chain keto acid dehydrogenase E1, beta polypeptide	-1.0690969	1.8051057	2.8039443
BCKDHB	branched chain keto acid dehydrogenase E1, beta polypeptide	1.3139023	2.253061	3.431873
BCL2L1	BCL2-like 1	-1.0856447	-1.5589603	-2.4461288
BCL2L11	BCL2-like 11 (apoptosis facilitator)	-1.3403285	1.438793	2.2519355
BCL2L12	BCL2-like 12 (proline rich)	1.1638856	-1.6050198	-1.3622673
BCL2L15	BCL2-like 15	-1.2581458	4.160914	6.2647204
BCL2L2	BCL2-like 2	-1.447257	-1.6292609	-1.7260565
BCL6	B-cell CLL/lymphoma 6	-1.7193977	-1.3254782	1.0505743
BCL7C	B-cell CLL/lymphoma 7C	1.0406904	-1.3771871	-2.0236425
BCL9L	B-cell CLL/lymphoma 9-like	-1.6520498	-2.6723645	-3.7919161
BCLAF1	BCL2-associated transcription factor 1	-1.6444227	-2.468583	-2.0256314
BCO2	beta-carotene oxygenase 2	-1.3629379	5.913634	3.5994415
BDH1	3-hydroxybutyrate dehydrogenase, type 1	-13.908468	13.605343	43.505985
BFAR	bifunctional apoptosis regulator	-1.2651478	-1.5766122	-1.5796572
BFSP1	beaded filament structural protein 1, filensin	2.118476	1.3427871	-4.2415605
BHMT2	betaine--homocysteine S-methyltransferase 2	-2.4159703	-1.3894783	2.9034297
BICD2	bicaudal D homolog 2 (Drosophila)	1.1736606	-1.2805879	-1.863722
BID	BH3 interacting domain death agonist	-1.0138057	-1.3029178	-2.501141
BID	BH3 interacting domain death agonist	-1.2151672	-1.6017032	-3.00361
BIN3	bridging integrator 3	-1.5865755	-1.677288	-1.6767919
BIRC2	baculoviral IAP repeat containing 2	-1.5511589	-2.0884697	-2.0508237
BIRC3	baculoviral IAP repeat containing 3	1.5505004	1.1293097	-4.1469336
BIRC5	baculoviral IAP repeat containing 5	2.3338776	-3.7284017	-3.2031634
BIVM	basic, immunoglobulin-like variable motif containing	1.2506342	1.7368692	1.5017856
BLCAP	bladder cancer associated protein	1.9695421	2.0675182	2.2857182
BLCAP	bladder cancer associated protein	1.8142246	1.7466652	1.7822611
BLNK	B-cell linker	-1.9099792	1.3056197	-1.3311427
BMP1	bone morphogenetic protein 1	-1.6133847	-3.5236485	-7.30653
BMP2	bone morphogenetic protein 2	-4.9970613	-2.6147552	-3.5892878
BMPER	BMP binding endothelial regulator	2.4081066	-1.9571776	-11.172955
BMPR1A	bone morphogenetic protein receptor, type IA	1.5334786	1.0963714	1.6636839
BMPR1A	bone morphogenetic protein receptor, type IA	1.4851223	1.1134231	1.6453248
BMS1P6	BMS1 pseudogene 6	2.3076162	2.3989706	2.559281
BNIP3	BCL2/adenovirus E1B 19kDa interacting protein 3	1.3691505	1.9777422	2.7124262
BNIP3L	BCL2/adenovirus E1B 19kDa interacting protein 3-like	-1.3540225	-1.7131327	-1.2151039
BNIP3L	BCL2/adenovirus E1B 19kDa interacting protein 3-like	-3.268515	-2.8994815	-2.5574825
BOD1L1	biorientation of chromosomes in cell division 1-like 1	-1.9615264	-1.6223092	-1.77369
BOLA1	bolA family member 1	1.2678611	1.7884164	1.6880641
BOLA2B	bolA family member 2B	1.0060517	-1.2306072	-1.958262
BOLA2B	bolA family member 2B	1.046815	-1.1611753	-2.3024573
BOLA2B	bolA family member 2B	-1.0785556	-1.4239695	-2.3921309
BPHL	biphenyl hydrolase-like (serine hydrolase)	-1.4722612	1.5457333	1.7774689
BRCA1	breast cancer 1, early onset	1.5453767	-2.3781085	-3.408607
BRD2	bromodomain containing 2	1.042434	-1.0193162	1.9403752
BRD3	bromodomain containing 3	-1.6852124	-1.386925	-1.3789085
BRD3	bromodomain containing 3	-1.0570128	1.0579138	2.7595394
BRD8	bromodomain containing 8	-1.4845111	-1.7320309	-1.1912115
BRF1	BRF1, RNA polymerase III transcription initiation factor 90 kDa subunit	1.5777566	1.451775	1.5955297
BRF1	BRF1, RNA polymerase III transcription initiation factor 90 kDa subunit	1.785162	1.9059749	2.0874736
BRINP1	bone morphogenetic protein/retinoic acid inducible neural-specific 1	2.9242437	1.9045266	1.9373963
BRIX1	BRX1, biogenesis of ribosomes, homolog (S. cerevisiae)	1.3757715	-1.3656685	-1.7722021
BRMS1	breast cancer metastasis suppressor 1	-1.2180731	-1.2097571	-1.5371304
BRWD1	bromodomain and WD repeat domain containing 1	-1.5504711	-1.2738503	-1.3317013
BSDC1	BSD domain containing 1	-1.5752125	-1.491808	1.1479332
BSG	basigin (Ok blood group)	-1.9819238	-1.3291618	-1.7181649
BTBD1	BTB (POZ) domain containing 1	1.6760463	1.4032497	1.4352638
BTBD11	BTB (POZ) domain containing 11	-1.1415993	-1.5477653	1.8055173
BTD	biotinidase	-1.8554187	1.2697526	4.13604
BTF3L4	basic transcription factor 3-like 4	2.4707828	1.8023888	1.6204666
BTF3P11	basic transcription factor 3 pseudogene 11	1.5113101	1.3091595	1.0293447
BTG1	B-cell translocation gene 1, anti-proliferative	-4.436241	-1.5081518	-1.0204855
BTG3	BTG family, member 3	-1.1124504	-2.101514	-5.30283
BTNL8	butyrophilin-like 8	-1.2725918	17.624434	31.80184
BUB1	BUB1 mitotic checkpoint serine/threonine kinase	2.2439797	-4.8286195	-3.5487707
BUB1B	BUB1 mitotic checkpoint serine/threonine kinase B	1.3643364	-4.469962	-5.621183
BUB3	BUB3 mitotic checkpoint protein	1.5817564	-1.112122	-1.0361346
BUB3	BUB3 mitotic checkpoint protein	1.3393304	-1.5632104	-1.7301203
BYSL	bystin-like	1.0742594	-1.3323274	-1.8631848
BZW2	basic leucine zipper and W2 domains 2	1.9991423	1.3052363	-1.1441433
C10orf10	chromosome 10 open reading frame 10	1.2569039	1.7821664	5.1989303
C10orf88	chromosome 10 open reading frame 88	1.2677833	-1.2295015	-1.5338552
C11orf1	chromosome 11 open reading frame 1	1.3281834	1.899316	1.5931237
C11orf52	chromosome 11 open reading frame 52	-1.3533186	2.4812582	4.18085
C11orf54	chromosome 11 open reading frame 54	-1.3063583	2.9516747	4.6745763
C11orf54	chromosome 11 open reading frame 54	-1.4470035	1.6424917	2.5023544
C11orf74	chromosome 11 open reading frame 74	1.3006054	1.2623651	1.8766086
C12orf73	chromosome 12 open reading frame 73	1.7959756	1.6628407	2.224466
C14orf1	chromosome 14 open reading frame 1	1.089639	1.4262527	2.5499072
C14orf132	chromosome 14 open reading frame 132	-2.0917826	-1.8903731	-1.4165728
C14orf159	chromosome 14 open reading frame 159	1.2154081	1.575292	1.1614492
C14orf169	chromosome 14 open reading frame 169	1.8968323	1.3132774	1.6140603
C14orf37	chromosome 14 open reading frame 37	1.9154662	-1.187673	-1.2447556

C14orf80	chromosome 14 open reading frame 80	1.0076696	-3.2087815	-3.50997
C15orf52	chromosome 15 open reading frame 52	1.1275587	-1.2761178	-1.7470769
C15orf61	chromosome 15 open reading frame 61	2.219006	2.338042	2.2893865
C16orf59	chromosome 16 open reading frame 59	1.3587343	-2.2582643	-3.3768892
C16orf74	chromosome 16 open reading frame 74	1.9129752	-1.009034	-2.1286774
C16orf95	chromosome 16 open reading frame 95	1.7942415	1.9857762	2.5357783
C17orf107	chromosome 17 open reading frame 107	-2.1255248	1.1010269	-2.0450099
C17orf49	chromosome 17 open reading frame 49	-1.293967	-1.7389256	-1.7423458
C17orf51	chromosome 17 open reading frame 51	1.0465363	1.0912117	2.8175752
C17orf51	chromosome 17 open reading frame 51	-1.1392624	-1.0988678	1.9817184
C17orf51	chromosome 17 open reading frame 51	1.0663266	1.0935885	2.7158587
C17orf51	chromosome 17 open reading frame 51	1.1045064	1.0124741	2.6424901
C17orf59	chromosome 17 open reading frame 59	1.1602387	1.4813399	1.8710543
C17orf82	chromosome 17 open reading frame 82	1.9470786	1.7846379	-1.3733153
C17orf89	chromosome 17 open reading frame 89	1.5820338	1.2343976	1.1369638
C17orf89	chromosome 17 open reading frame 89	1.6381145	1.283412	1.2153771
C17orf96	chromosome 17 open reading frame 96	1.6954513	1.1374811	1.4142845
C18orf12	chromosome 18 open reading frame 12	2.6499724	2.1672027	2.3761954
C19orf12	chromosome 19 open reading frame 12	-1.2638537	1.2399957	1.7095796
C19orf12	chromosome 19 open reading frame 12	1.524035	1.8138065	2.1786315
C19orf25	chromosome 19 open reading frame 25	-1.1941785	1.2866515	-1.9275668
C19orf44	chromosome 19 open reading frame 44	1.5830655	1.769027	1.8602458
C19orf52	chromosome 19 open reading frame 52	1.0559909	-1.0063894	1.8260945
C19orf66	chromosome 19 open reading frame 66	-1.2251883	2.3711646	3.3003623
C1D	C1D nuclear receptor corepressor	1.3258641	1.5620145	1.9987979
C1GALT1	core 1 synthase, glycoprotein-N-acetylgalactosamine 3-beta-galactosyltransferase 1	-1.2706618	-2.3250704	-1.5128762
C1GALT1	core 1 synthase, glycoprotein-N-acetylgalactosamine 3-beta-galactosyltransferase 1	-1.3343565	-2.4525332	-1.6444682
C1orf112	chromosome 1 open reading frame 112	1.377738	-2.270819	-2.923831
C1orf115	chromosome 1 open reading frame 115	-1.7260654	1.9094661	3.301095
C1orf122	chromosome 1 open reading frame 122	1.4034306	1.7402853	1.8117911
C1orf131	chromosome 1 open reading frame 131	1.8528419	1.423841	1.6523832
C1orf140	uncharacterized LOC400804	4.2191486	4.0339246	5.197757
C1orf158	chromosome 1 open reading frame 158	2.1891165	2.4769642	2.4629138
C1orf159	chromosome 1 open reading frame 159	-1.4240851	-1.2638378	-1.9610077
C1orf21	chromosome 1 open reading frame 21	1.5066923	2.9555056	2.3625538
C1orf226	chromosome 1 open reading frame 226	1.4789867	4.82547	11.477414
C1orf229	chromosome 1 open reading frame 229	2.2121904	2.1444838	2.5373845
C1orf50	chromosome 1 open reading frame 50	1.0645194	1.2910355	1.5327036
C1orf52	chromosome 1 open reading frame 52	-1.5448135	-2.17952	-1.5650315
C1orf53	chromosome 1 open reading frame 53	1.3832045	1.9552417	1.7751409
C1orf56	chromosome 1 open reading frame 56	1.1635118	1.5330129	2.6768537
C1QL1	complement component 1, q subcomponent-like 1	1.9095289	1.5710472	-2.1149817
C1QTNF1	C1q and tumor necrosis factor related protein 1	-2.0501401	1.1337535	-1.8654723
C1QTNF1-AS1	C1QTNF1 antisense RNA 1	-1.4078338	2.7364821	-1.2432418
C1QTNF4	C1q and tumor necrosis factor related protein 4	-1.7246472	4.5436506	3.4234262
C1R	complement component 1, r subcomponent	1.0790782	1.9676253	1.5657157
C1R	complement component 1, r subcomponent	-1.0026698	2.0865786	1.4932997
C1RL	complement component 1, r subcomponent-like	-1.0430475	1.583232	1.9198308
C1S	complement component 1, s subcomponent	-3.1757135	1.2501848	1.2149776
C1S	complement component 1, s subcomponent	-1.4676422	2.0502405	2.2683928
C2	complement component 2	-2.8095112	1.2602704	1.2015105
C2	complement component 2	-4.386083	1.1164203	-1.0368524
C20orf24	chromosome 20 open reading frame 24	1.6517454	1.5890825	1.2322032
C20orf27	chromosome 20 open reading frame 27	-1.2146248	-1.9084715	-2.5735013
C20orf27	chromosome 20 open reading frame 27	1.5496284	-1.0150765	-1.2701235
C21orf33	chromosome 21 open reading frame 33	1.5770783	2.3505914	2.5542316
C21orf58	chromosome 21 open reading frame 58	2.7612877	3.6409776	3.5991247
C22orf29	chromosome 22 open reading frame 29	1.4323399	1.4819021	2.0097134
C2CD2L	C2CD2-like	-1.0424094	1.1051883	2.058308
C2CD3	C2 calcium-dependent domain containing 3	-1.100647	-1.7432274	-1.4907575
C2CD4C	C2 calcium-dependent domain containing 4C	1.5415336	1.5506169	1.6305825
C2orf47	chromosome 2 open reading frame 47	1.132563	1.4973224	2.240465
C2orf49	chromosome 2 open reading frame 49	1.6486555	1.2919537	1.3767562
C2orf69	chromosome 2 open reading frame 69	1.2624623	1.2523837	2.1854005
C2orf69	chromosome 2 open reading frame 69	1.5519953	1.5081755	2.53111
C2orf72	chromosome 2 open reading frame 72	1.1499449	6.9431434	11.005615
C2orf72	chromosome 2 open reading frame 72	1.4351333	2.4587119	3.1126828
C2orf76	chromosome 2 open reading frame 76	1.1221176	1.065029	2.251749
C3	complement component 3	-1.9664143	1.9285674	1.7642956
C3	complement component 3	-2.3958418	1.7807177	1.4932432
C3AR1	complement component 3a receptor 1	3.2503347	3.907115	4.459352
C3orf38	chromosome 3 open reading frame 38	1.6148338	1.5751075	2.140253
C3orf52	chromosome 3 open reading frame 52	-1.2810324	-1.9203343	-6.179528
C4B	complement component 4B (Chido blood group)	-2.9693985	2.074865	1.4945196
C4BPA	complement component 4 binding protein, alpha	-2.619399	6.819954	6.020934
C4BPB	complement component 4 binding protein, beta	1.1454849	4.1315928	4.8429465
C4orf3	chromosome 4 open reading frame 3	1.78036	1.9015023	1.8955467
C4orf46	chromosome 4 open reading frame 46	1.8839933	-2.8218515	-3.7906349
C4orf46	chromosome 4 open reading frame 46	1.8973321	-2.8851511	-2.4730577
C4orf48	chromosome 4 open reading frame 48	-1.0004802	-1.796804	-1.6946172
C5	complement component 5	-3.007958	2.4212735	2.7797174
C5orf51	chromosome 5 open reading frame 51	1.178677	1.275577	1.5423218
C5orf63	chromosome 5 open reading frame 63	-1.7948782	-2.2337737	1.1482813
C6	complement component 6	-3.4447124	9.018667	8.631901
C6orf106	chromosome 6 open reading frame 106	-1.3923959	-1.2105436	1.5595052
C6orf106	chromosome 6 open reading frame 106	1.1808897	1.1917863	1.5347998
C6orf136	chromosome 6 open reading frame 136	1.2043464	1.7843566	1.5559274
C6orf203	chromosome 6 open reading frame 203	1.4661125	1.8863348	2.2265818
C6orf211	chromosome 6 open reading frame 211	1.1949518	1.5453199	1.8327149
C6orf226	chromosome 6 open reading frame 226	1.6685055	1.8318188	2.0197947
C7orf43	chromosome 7 open reading frame 43	1.6147152	2.0757153	2.4406672
C7orf55	chromosome 7 open reading frame 55	1.6890965	2.092036	1.8181001
C7orf60	chromosome 7 open reading frame 60	2.9943278	1.1763866	1.2606668
C7orf73	chromosome 7 open reading frame 73	1.8761753	1.2347207	1.298412
C8A	complement component 8, alpha polypeptide	-8.144237	6.174751	5.2737966
C8B	complement component 8, beta polypeptide	-6.0747705	9.227594	7.623053
C8G	complement component 8, gamma polypeptide	-3.514839	6.485835	5.2991004
C8G	complement component 8, gamma polypeptide	-1.908726	5.754816	4.692093
C8orf37	chromosome 8 open reading frame 37	1.3353796	1.4189382	1.9099712
C8orf44-SGK3	C8orf44-SGK3 readthrough	-2.4203136	-2.1575503	-1.7072564
C8orf46	chromosome 8 open reading frame 46	1.6840595	8.260785	1.9278072
C8orf58	chromosome 8 open reading frame 58	3.539835	3.4552605	4.039921
C8orf82	chromosome 8 open reading frame 82	1.3303025	1.5283585	-1.0551513
C9orf156	chromosome 9 open reading frame 156	-1.4508784	-1.5964469	-1.3502426
C9orf163	chromosome 9 open reading frame 163	2.9004598	2.540404	2.6117592
C9orf173	chromosome 9 open reading frame 173	1.8751872	1.9385881	2.9457014
C9orf173	chromosome 9 open reading frame 173	2.0863059	1.8586578	2.5128741
C9orf173-AS1	C9orf173 antisense RNA 1	2.035957	2.1082087	2.4485116
C9orf3	chromosome 9 open reading frame 3	1.3886173	1.4434897	1.9468288

C9orf40	chromosome 9 open reading frame 40	1.2528334	-2.203585	-1.3176323
C9orf40	chromosome 9 open reading frame 40	1.232558	-2.9480324	-2.3072248
C9orf85	chromosome 9 open reading frame 85	-1.3885412	-1.5794516	-1.6040888
CA12	carbonic anhydrase XII	-1.9310125	-3.8439736	-6.405623
CA2	carbonic anhydrase II	-1.1802961	1.6667641	3.185818
CA5BP1	carbonic anhydrase VB pseudogene 1	2.4559557	3.0825956	1.6294757
CA6	carbonic anhydrase VI	2.4582021	2.6225252	2.7174106
CA9	carbonic anhydrase IX	2.4052973	1.6792604	-5.5561953
CAAP1	caspase activity and apoptosis inhibitor 1	1.483969	1.8444381	1.3989155
CAB39	calcium binding protein 39	-1.8954648	-1.8839905	-1.7476126
CABLES1	Cdk5 and Abl enzyme substrate 1	1.0861373	1.4312176	1.5860301
CABP5	calcium binding protein 5	3.8952398	3.5022275	4.2826266
CACFD1	calcium channel flower domain containing 1	-1.3821104	1.6259454	3.6372755
CACNA1C	calcium channel, voltage-dependent, L type, alpha 1C subunit	1.9869194	2.2629273	2.7082891
CACNA1H	calcium channel, voltage-dependent, T type, alpha 1H subunit	1.0482429	1.0714917	-2.0859187
CACNB3	calcium channel, voltage-dependent, beta 3 subunit	-1.3070973	-3.34115	-8.209712
CACTIN	cactin, spliceosome C complex subunit	1.802155	1.6999558	1.8533111
CACYBP	calcyclin binding protein	1.772444	-1.1537427	-1.5043445
CACYBP	calcyclin binding protein	1.0727183	-1.6516432	-2.3570032
CAD	carbamoyl-phosphate synthetase 2, aspartate transcarbamylase, and dihydroorotase	-1.7544049	-1.9432912	-3.0081387
CADM1	cell adhesion molecule 1	1.1902298	-2.0470202	-1.1287607
CALCOCO2	calcium binding and coiled-coil domain 2	1.4297572	2.2133205	2.7357533
CALCR	calcitonin receptor	7.7774706	2.7611628	-2.1355836
CALM1	calmodulin 1 (phosphorylase kinase, delta)	1.4482806	1.5819782	2.122507
CALML4	calmodulin-like 4	-1.5297537	11.870286	10.304202
CALU	calumenin	-1.3387685	-2.7225206	-3.6832829
CAMK1D	calcium/calmodulin-dependent protein kinase ID	-1.4198463	-2.7261844	-2.6172976
CAMK1D	calcium/calmodulin-dependent protein kinase ID	-1.3864058	-2.4459696	-2.2041593
CAMK2D	calcium/calmodulin-dependent protein kinase II delta	-1.6629871	-1.3952239	1.1029404
CAMK2N1	calcium/calmodulin-dependent protein kinase II inhibitor 1	-2.3979084	-1.0799799	-1.4691685
CAMK2N2	calcium/calmodulin-dependent protein kinase II inhibitor 2	1.0845482	1.2546389	1.6316789
CAMSAP2	calmodulin regulated spectrin-associated protein family, member 2	-1.22516	-1.5591216	-1.9116366
CAP1	CAP, adenylate cyclase-associated protein 1 (yeast)	-1.1759354	-1.6490176	-2.1381595
CAP1	CAP, adenylate cyclase-associated protein 1 (yeast)	-1.150221	-1.606376	-2.1018188
CAPN2	calpain 2, (m/II) large subunit	1.1117785	-1.3150823	-1.8326273
CAPN5	calpain 5	-2.7397232	-1.3257548	2.277163
CAPNS2	calpain, small subunit 2	2.6592653	2.137003	1.4023113
CAPZB	capping protein (actin filament) muscle Z-line, beta	1.046521	-1.4488622	-1.6584586
CARD14	caspase recruitment domain family, member 14	2.0363011	1.3668664	1.569733
CARD6	caspase recruitment domain family, member 6	-1.4328431	-1.5385841	-2.4547365
CARHSP1	calcium regulated heat stable protein 1, 24kDa	1.4926682	1.5093156	1.9299562
CARM1	coactivator-associated arginine methyltransferase 1	-1.3930097	2.4004624	3.4370801
CARS2	cysteinyl-tRNA synthetase 2, mitochondrial (putative)	1.5514153	1.3049039	1.0049119
CASC10	cancer susceptibility candidate 10	1.8997303	2.1054583	5.6123753
CASC22	cancer susceptibility candidate 22 (non-protein coding)	1.1313361	2.4427795	9.643483
CASK	calcium/calmodulin-dependent serine protein kinase (MAGUK family)	-1.1519992	-1.150549	-2.0181155
CASK	calcium/calmodulin-dependent serine protein kinase (MAGUK family)	-1.9367716	-2.7558055	-2.639254
CASKIN1	CASK interacting protein 1	2.2102127	2.1655202	1.7741827
CASP1	caspase 1, apoptosis-related cysteine peptidase	1.1643605	-1.3122874	-1.8559158
CASP10	caspase 10, apoptosis-related cysteine peptidase	-1.2775694	-1.6347654	-1.264369
CASP2	caspase 2, apoptosis-related cysteine peptidase	1.6674863	1.4760996	1.6712581
CAST	calpastatin	1.5047472	1.6782165	1.3534509
CASZ1	castor zinc finger 1	2.8607574	3.598217	4.4822445
CAT	catalase	-1.9250919	1.1709598	3.2664008
CATSPERG	catsper channel auxiliary subunit gamma	2.0425	2.3233984	2.558556
CAV1	caveolin 1, caveolae protein, 22kDa	3.3622506	2.2149556	-1.0903031
CAV1	caveolin 1, caveolae protein, 22kDa	3.3896658	2.292583	-1.0161225
CAV2	caveolin 2	2.257168	2.7127354	1.474514
CAV3	caveolin 3	9.08027	5.334745	2.3923411
CBFB	core-binding factor, beta subunit	1.170933	-1.3881439	-2.0032609
CBR1	carbonyl reductase 1	1.3825098	1.9967556	2.603479
CBR4	carbonyl reductase 4	1.0187387	2.011914	2.654601
CBS	cystathionine-beta-synthase	-1.0196854	1.6585517	1.5062757
CBWD5	COBW domain containing 5	-1.0879691	-1.5671458	-1.0594656
CBX1	chromobox homolog 1	1.8355811	-1.1555063	1.0592245
CBX2	chromobox homolog 2	1.2382187	-1.4178808	-1.8487507
CBX3	chromobox homolog 3	1.2145565	-1.5791656	-2.362232
CBX5	chromobox homolog 5	-1.0932453	-1.5191455	-1.8501759
CBX7	chromobox homolog 7	-1.2995086	1.213563	1.7855563
CBX7	chromobox homolog 7	2.3774948	2.6713262	3.058037
CC2D1B	coiled-coil and C2 domain containing 1B	-1.3692025	-1.5323001	-1.7208129
CC2D1B	coiled-coil and C2 domain containing 1B	-1.379077	-1.4899721	-1.7478151
CCBE1	collagen and calcium binding EGF domains 1	5.3403473	2.2645183	-2.4635203
CCBL1	cysteine conjugate-beta lyase, cytoplasmic	-1.7641752	1.1741508	1.5074764
CCBL2	cysteine conjugate-beta lyase 2	-1.7145643	-1.357814	1.017168
CCDC102A	coiled-coil domain containing 102A	-1.0016115	-1.0348724	-3.5105648
CCDC106	coiled-coil domain containing 106	1.6150665	2.6434224	1.5218883
CCDC125	coiled-coil domain containing 125	-1.6761658	-1.0330355	1.3442265
CCDC125	coiled-coil domain containing 125	-1.5533637	-1.0356634	1.4170043
CCDC125	coiled-coil domain containing 125	-1.410251	1.1603272	1.8772863
CCDC136	coiled-coil domain containing 136	-2.0424242	-5.2851157	-7.474779
CCDC137	coiled-coil domain containing 137	1.1512249	-1.2988558	-1.5564791
CCDC14	coiled-coil domain containing 14	-1.4348997	-3.2133338	-2.471661
CCDC149	coiled-coil domain containing 149	-1.5212421	-1.0517955	1.1634313
CCDC149	coiled-coil domain containing 149	-2.12695	2.3166413	2.477933
CCDC170	coiled-coil domain containing 170	-4.1621494	8.531871	12.161863
CCDC171	coiled-coil domain containing 171	1.9815937	2.8085866	3.1034749
CCDC171	coiled-coil domain containing 171	1.6220156	4.185447	5.662917
CCDC172	coiled-coil domain containing 172	3.4789898	2.8917785	2.901089
CCDC177	coiled-coil domain containing 177	7.041447	9.377143	10.029277
CCDC178	coiled-coil domain containing 178	-1.3519782	-2.0528002	-14.381543
CCDC178	coiled-coil domain containing 178	1.3539394	-1.0109955	-10.491618
CCDC23	coiled-coil domain containing 23	-1.1652832	-1.5541111	-1.5136826
CCDC23	coiled-coil domain containing 23	-1.4409597	-1.8118085	-1.7836201
CCDC25	coiled-coil domain containing 25	1.1237243	1.5346233	1.912366
CCDC28A	coiled-coil domain containing 28A	-1.3833429	1.6812332	2.6987267
CCDC3	coiled-coil domain containing 3	1.3360183	-1.2395896	-2.356245
CCDC34	coiled-coil domain containing 34	1.3582652	-1.9755204	-1.7193066
CCDC50	coiled-coil domain containing 50	-1.1549767	-1.481176	-1.8477448
CCDC51	coiled-coil domain containing 51	1.5906597	1.374791	-1.3063368
CCDC57	coiled-coil domain containing 57	-1.0599015	1.9105569	1.3363839
CCDC59	coiled-coil domain containing 59	-1.1121379	-1.9133693	-1.8750607
CCDC6	coiled-coil domain containing 6	-1.2945205	-1.6381468	-2.3881748
CCDC69	coiled-coil domain containing 69	1.5157645	1.5185612	1.5253928
CCDC71L	coiled-coil domain containing 71-like	-1.6552444	-1.7711558	-1.4353354
CCDC77	coiled-coil domain containing 77	1.1672854	-1.861926	-1.5620967
CCDC80	coiled-coil domain containing 80	3.7660272	-2.569012	-5.5022836
CCDC85C	coiled-coil domain containing 85C	2.0630817	2.027426	2.6336398
CCDC86	coiled-coil domain containing 86	2.0219736	1.2080824	1.021001

CCDC88A	coiled-coil domain containing 88A	-1.0322589	-1.3627558	-1.678902
CCDC88B	coiled-coil domain containing 88B	-1.1621367	1.3200206	-2.8527331
CCK	cholecystokinin	-2.301105	-7.3475246	-34.548172
CCL15	chemokine (C-C motif) ligand 15	-1.5310338	1.9015856	3.1169283
CCL2	chemokine (C-C motif) ligand 2	-4.4786844	-8.75168	-33.805935
CCL20	chemokine (C-C motif) ligand 20	-14.527544	-10.680048	-15.990301
CCL24	chemokine (C-C motif) ligand 24	2.0835085	2.0249999	2.2938404
CCM2	cerebral cavernous malformation 2	1.7543172	1.53953	2.5342445
CCNA2	cyclin A2	2.2884636	-3.3320723	-2.9421172
CCNB1	cyclin B1	2.5214682	-3.2039697	-3.2733734
CCNB1	cyclin B1	2.2543542	-1.5774603	-1.2876666
CCNB1IP1	cyclin B1 interacting protein 1, E3 ubiquitin protein ligase	1.2053307	1.2978169	1.7585766
CCNE1	cyclin E1	1.467743	-1.3434244	-1.9133521
CCNE2	cyclin E2	2.2785964	-2.6881804	-2.4605396
CCNH	cyclin H	2.6217124	1.3349049	1.1566237
CCNI	cyclin I	-1.2923037	-1.3985242	-1.647343
CCNK	cyclin K	-1.3765538	-2.2364862	-2.262114
CCNL1	cyclin L1	-2.8403597	-3.2240138	-2.485681
CCNL2	cyclin L2	-2.0969687	-1.9697527	-1.4433655
CCNL2	cyclin L2	-1.831372	-1.4496359	-1.7043412
CCNL2	cyclin L2	1.7636176	1.9294745	1.5290713
CCNO	cyclin O	-1.1402651	-1.4505183	-1.5664071
CCRL2	chemokine (C-C motif) receptor-like 2	-1.359364	-1.8388052	-5.296845
CCSAP	centriole, cilia and spindle-associated protein	1.312025	-1.8655933	-1.0152205
CCT4	chaperonin containing TCP1, subunit 4 (delta)	-1.0630462	-1.2628304	-1.6126457
CCT6A	chaperonin containing TCP1, subunit 6A (zeta 1)	1.0759534	-1.4194349	-1.720647
CCZ1	CCZ1 vacuolar protein trafficking and biogenesis associated homolog (S. cerevisiae)	-1.6453133	-1.6648644	-1.4743567
CD109	CD109 molecule	-1.02431	-1.6991421	-8.380336
CD14	CD14 molecule	1.4780097	2.3367283	2.1726503
CD151	CD151 molecule (Raph blood group)	1.2528915	-1.1181865	-1.5259881
CD164L2	CD164 sialomucin-like 2	1.4673555	1.6183883	1.610337
CD24	CD24 molecule	2.7370076	1.7011163	1.2307826
CD276	CD276 molecule	-1.075061	-1.8527073	-1.9052004
CD276	CD276 molecule	-1.2700604	-1.5045701	-2.0270474
CD2BP2	CD2 (cytoplasmic tail) binding protein 2	-1.3829312	-1.6334122	-1.863897
CD300E	CD300e molecule	2.7582295	2.032955	2.6732714
CD302	CD302 molecule	1.0907507	1.5741905	2.916111
CD302	CD302 molecule	-1.0025157	1.8213154	2.7641783
CD320	CD320 molecule	1.0253363	-1.0982391	-2.0910106
CD3EAP	CD3e molecule, epsilon associated protein	1.5741435	1.0305163	1.1056218
CD4	CD4 molecule	1.4499584	1.6157179	1.8991779
CD44	CD44 molecule (Indian blood group)	-1.3893039	-2.8572142	-5.6542373
CD46	CD46 molecule, complement regulatory protein	-1.0027188	-1.3649737	-1.508169
CD55	CD55 molecule, decay accelerating factor for complement (Cromer blood group)	-1.7233247	-2.0263007	-2.829571
CD68	CD68 molecule	-3.5674598	-1.3628808	-1.3743969
CD70	CD70 molecule	2.6456025	-1.3185391	-5.1856904
CD72	CD72 molecule	1.9612496	2.0523436	2.6153078
CD82	CD82 molecule	-1.7712431	-2.337596	-2.8076944
CD9	CD9 molecule	1.1093141	-1.9133415	-3.3673034
CD97	CD97 molecule	-1.6441492	-1.8089405	-2.4560833
CD99	CD99 molecule	-1.3114556	-1.9952164	-1.4440968
CDA	cytidine deaminase	2.7005105	1.8752806	-2.2482476
CDADC1	cytidine and dCMP deaminase domain containing 1	1.0238533	1.0264046	1.8190864
CDC14B	cell division cycle 14B	-1.865851	-1.2732863	1.2768346
CDC14C	cell division cycle 14C	1.6951634	1.8044286	2.8519583
CDC20	cell division cycle 20	2.3878086	-1.9947248	-2.7497776
CDC25B	cell division cycle 25B	-1.2206246	-3.885996	-4.367591
CDC26	cell division cycle 26	1.4480463	1.4419041	1.5123109
CDC34	cell division cycle 34	-1.400624	-1.1720046	1.5511591
CDC37	cell division cycle 37	-1.3538781	-1.4179862	-2.0008924
CDC37L1	cell division cycle 37-like 1	-1.1891869	1.210994	1.6433902
CDC42	cell division cycle 42	-1.0946083	-1.7472472	-1.9812882
CDC42EP1	CDC42 effector protein (Rho GTPase binding) 1	1.4166828	-1.0625774	-2.1991985
CDC42EP2	CDC42 effector protein (Rho GTPase binding) 2	1.0914012	-1.5442882	-1.6356317
CDC45	cell division cycle 45	1.9586558	-3.7773335	-6.8993316
CDC5L	cell division cycle 5-like	-1.3523384	-1.7735839	-1.5585289
CDC7	cell division cycle 7	1.1204563	-3.1416109	-4.309325
CDCA2	cell division cycle associated 2	1.2203649	-5.335274	-5.123806
CDCA3	cell division cycle associated 3	1.8196207	-2.241176	-2.1907918
CDCA5	cell division cycle associated 5	1.6396421	-4.3463845	-4.7268124
CDCA7	cell division cycle associated 7	1.8254371	-2.599233	-11.843275
CDCA7L	cell division cycle associated 7-like	1.3180584	-2.0654984	-1.7551552
CDCA8	cell division cycle associated 8	3.9889781	-1.8574493	-1.7540526
CDCP2	CUB domain containing protein 2	2.1540592	2.5000427	3.9502997
CDH1	cadherin 1, type 1, E-cadherin (epithelial)	-1.7202641	2.338651	2.962852
CDH10	cadherin 10, type 2 (T2-cadherin)	-1.1177474	-1.8123649	-3.193864
CDH11	cadherin 11, type 2, OB-cadherin (osteoblast)	1.2909936	-1.2859281	-5.9501066
CDH16	cadherin 16, KSP-cadherin	-2.330434	-2.7909367	-49.729145
CDH18	cadherin 18, type 2	1.1974565	-1.7165521	-2.493499
CDH19	cadherin 19, type 2	-2.260023	-1.1275712	1.0787522
CDH2	cadherin 2, type 1, N-cadherin (neuronal)	-2.2100675	-2.7953334	-2.411592
CDH22	cadherin 22, type 2	2.0932598	2.1826787	2.33161
CDIP1	cell death-inducing p53 target 1	1.960267	1.1564345	-1.6553855
CDIP1	cell death-inducing p53 target 1	1.8618848	1.698229	2.1940706
CDK1	cyclin-dependent kinase 1	2.3649933	-4.170054	-5.0331993
CDK10	cyclin-dependent kinase 10	-1.9480839	-1.3946542	-1.2443839
CDK2AP2	cyclin-dependent kinase 2 associated protein 2	1.3375616	2.2459252	2.23382
CDK3	cyclin-dependent kinase 3	1.6210867	1.8338528	2.216453
CDK4	cyclin-dependent kinase 4	-1.1781323	-1.7258989	-2.7311504
CDK5R1	cyclin-dependent kinase 5, regulatory subunit 1 (p35)	2.3573196	1.0765375	-1.2290324
CDK5RAP2	CDK5 regulatory subunit associated protein 2	-1.716569	-1.9929125	-2.2396998
CDK5RAP2	CDK5 regulatory subunit associated protein 2	-2.0627623	-2.1693563	-2.5726316
CDK5RAP3	CDK5 regulatory subunit associated protein 3	-1.7560465	-1.4255683	-1.4717612
CDK6	cyclin-dependent kinase 6	1.0383116	-1.1715977	-1.5654653
CDK9	cyclin-dependent kinase 9	1.2527852	1.3040534	1.5702205
CDKN1A	cyclin-dependent kinase inhibitor 1A (p21, Cip1)	-1.3432137	-2.1056917	-1.9781319
CDKN1B	cyclin-dependent kinase inhibitor 1B (p27, Kip1)	1.1511217	1.4659839	1.8613856
CDKN2A	cyclin-dependent kinase inhibitor 2A	1.0052303	-1.592626	-2.358531
CDKN2A	cyclin-dependent kinase inhibitor 2A	-1.173202	-2.2740974	-3.9951606
CDKN2C	cyclin-dependent kinase inhibitor 2C (p18, inhibits CDK4)	2.9403958	1.181073	1.5305635
CDKN2D	cyclin-dependent kinase inhibitor 2D (p19, inhibits CDK4)	1.723643	-1.5639099	-1.121722
CDKN3	cyclin-dependent kinase inhibitor 3	2.4238067	-2.7996125	-1.8048699
CDO1	cysteine dioxygenase type 1	1.3995088	3.0079076	10.986873
CDPF1	cysteine-rich, DPF motif domain containing 1	1.8659464	1.9728211	1.5777974
CDPF1	cysteine-rich, DPF motif domain containing 1	1.5367454	1.7033273	1.2859881
CDR2L	cerebellar degeneration-related protein 2-like	1.7281408	-1.1811674	-2.5824761
CDRT3	CMT1A duplicated region transcript 3	2.5506856	2.5095506	2.6177683
CDT1	chromatin licensing and DNA replication factor 1	1.5406036	-4.125012	-7.341541
CDX1	caudal type homeobox 1	1.2758662	2.264565	2.9767778

CEACAM1	carcinoembryonic antigen-related cell adhesion molecule 1 (biliary glycoprotein)	-1.8173847	1.0963026	-1.0527631
CEACAM19	carcinoembryonic antigen-related cell adhesion molecule 19	3.0748417	2.904287	3.4261682
CEACAM20	carcinoembryonic antigen-related cell adhesion molecule 20	6.066102	3.748601	3.5784633
CEBPA	CCAAT/enhancer binding protein (C/EBP), alpha	-1.9727405	3.220841	7.4487624
CEBPB	CCAAT/enhancer binding protein (C/EBP), beta	2.0376182	2.0845227	2.183864
CEBPG	CCAAT/enhancer binding protein (C/EBP), gamma	-1.8416834	-1.3296902	1.083675
CEBPZOS	CEBPZ opposite strand	2.1691873	2.1098735	2.1697726
CENPA	centromere protein A	2.946627	-3.11825	-3.2311242
CENPBD1	CENPB DNA-binding domains containing 1	1.3731658	1.4184834	1.6826043
CENPC	centromere protein C	-1.7323618	-2.0454822	-1.7096605
CENPF	centromere protein F, 350/400kDa	1.6358792	-3.5961502	-4.6854806
CENPH	centromere protein H	1.647217	-2.4608283	-2.453391
CENPJ	centromere protein J	-1.0390848	-2.9800518	-3.4832165
CENPM	centromere protein M	2.1618073	-3.242664	-5.02801
CENPM	centromere protein M	1.7647368	-4.284847	-7.10139
CENPN	centromere protein N	1.7801813	-1.7362238	-1.2545125
CENPP	centromere protein P	1.2752155	-1.9332992	-2.5832305
CENPQ	centromere protein Q	1.1580077	-2.6742654	-1.6968076
CENPU	centromere protein U	1.48301	-5.138367	-3.5441415
CENPW	centromere protein W	1.8416498	-3.600618	-5.011576
CEP104	centrosomal protein 104kDa	1.8859686	3.036774	4.2580175
CEP250	centrosomal protein 250kDa	1.0702803	-1.9471751	-2.5665357
CEP295	centrosomal protein 295kDa	-1.4341221	-2.0919106	-1.9380049
CEP350	centrosomal protein 350kDa	-2.725631	-1.9468241	-1.5325239
CEP41	centrosomal protein 41kDa	-1.3321297	-1.6900638	-2.106162
CEP55	centrosomal protein 55kDa	3.380514	-2.8517103	-5.5886927
CEP70	centrosomal protein 70kDa	2.1359084	1.5731863	1.0931505
CEP78	centrosomal protein 78kDa	1.0522171	-2.275311	-1.9049271
CERCAM	cerebral endothelial cell adhesion molecule	1.0670615	-1.2217553	-1.7515191
CERS4	ceramide synthase 4	-1.0478693	1.4677608	2.1747706
CERS5	ceramide synthase 5	-1.2050028	-1.8322109	-1.761167
CERS6	ceramide synthase 6	1.5443043	1.2521894	1.4605986
CES1	carboxylesterase 1	-1.7873281	1.4102172	2.1993568
CES1	carboxylesterase 1	-1.1985468	1.5082406	1.9642144
CES1	carboxylesterase 1	-2.1056192	1.2168169	1.8805809
CES1P2	carboxylesterase 1 pseudogene 2	-1.1973139	2.0231802	3.2665427
CES2	carboxylesterase 2	-2.1034904	-1.1685818	-1.3641028
CFAP36	cilia and flagella associated protein 36	-1.5694342	-1.7430832	-1.7046533
CFAP70	cilia and flagella associated protein 70	-2.1887062	1.8462611	2.808961
CFB	complement factor B	-3.7846935	2.0145075	2.9400492
CFH	complement factor H	-2.960379	1.5541457	1.3451055
CFH	complement factor H	-2.2644045	2.4546432	2.5008461
CFH	complement factor H	-2.4632092	1.87417	1.6137184
CFHR1	complement factor H-related 1	-9.43175	11.324512	18.579878
CFHR2	complement factor H-related 2	-10.8013525	13.364699	24.684967
CFHR3	complement factor H-related 3	-2.8176706	1.710417	1.5159496
CFHR4	complement factor H-related 4	-4.9307055	21.152094	5.578794
CFI	complement factor I	-1.4825758	2.543369	2.8770754
CFI	complement factor I	-2.4264257	1.7059005	1.7795906
CFL1	cofilin 1 (non-muscle)	-1.0170738	-1.9411275	-1.915567
CGB	chorionic gonadotropin, beta polypeptide	-1.3856219	-25.657263	-32.054504
CGN	cingulin	1.6669018	3.2247112	4.8019867
CGNL1	cingulin-like 1	-1.7216786	1.6619767	1.3966467
CHAF1A	chromatin assembly factor 1, subunit A (p150)	1.569513	-2.5554607	-2.640395
CHAF1B	chromatin assembly factor 1, subunit B (p60)	1.1491855	-3.6489944	-5.5588603
CHAT	choline O-acetyltransferase	2.9614193	3.4064415	3.2427764
CHCHD10	coiled-coil-helix-coiled-coil-helix domain containing 10	1.3562442	2.124968	1.2977289
CHCHD4	coiled-coil-helix-coiled-coil-helix domain containing 4	1.8089496	1.8846866	1.4307293
CHCHD7	coiled-coil-helix-coiled-coil-helix domain containing 7	2.0144415	1.4834636	2.6927776
CHD1	chromodomain helicase DNA binding protein 1	2.394097	2.240182	2.1518927
CHD1L	chromodomain helicase DNA binding protein 1-like	1.0664262	1.0815036	1.8997585
CHD2	chromodomain helicase DNA binding protein 2	-2.3234763	-2.0084033	-1.6977772
CHD3	chromodomain helicase DNA binding protein 3	-1.3176198	-1.9307952	-4.3797207
CHD4	chromodomain helicase DNA binding protein 4	-1.9766088	-1.7398537	-2.318193
CHD9	chromodomain helicase DNA binding protein 9	-3.005867	-2.1169548	-2.1396198
CHDH	choline dehydrogenase	-2.6570559	-1.2626793	1.2683617
CHDH	choline dehydrogenase	-1.6156195	1.0264722	1.9384779
CHKB	choline kinase beta	-1.9348994	-1.6410195	-2.4108377
CHML	choroideremia-like (Rab escort protein 2)	1.3271325	-1.1227736	-2.7437577
CHMP2A	charged multivesicular body protein 2A	1.1675712	1.482708	1.5400065
CHMP2B	charged multivesicular body protein 2B	1.0208782	1.0427952	1.7044691
CHMP3	charged multivesicular body protein 3	-1.0958836	-1.2871591	-1.9008938
CHN2	chimerin 2	-1.8520187	1.8267019	4.8866177
CHN2	chimerin 2	1.054734	2.5275185	6.4272695
CHORDC1	cysteine and histidine-rich domain (CHORD) containing 1	1.1300879	-1.0648762	-2.1513908
CHP1	calcineurin-like EF-hand protein 1	1.5593755	2.2501671	3.0395353
CHPF2	chondroitin polymerizing factor 2	-1.5221624	-2.6122534	-3.2450097
CHPT1	choline phosphotransferase 1	1.5719144	1.9609892	4.6473036
CHRAC1	chromatin accessibility complex 1	1.5739815	1.3903553	-1.341198
CHRD	chordin	-2.1314416	1.3629531	1.4759178
CHRDL2	chordin-like 2	-5.83751	-1.0485014	-2.6948342
CHST1	carbohydrate (keratan sulfate Gal-6) sulfotransferase 1	1.8431609	2.5129025	2.116457
CHST12	carbohydrate (chondroitin 4) sulfotransferase 12	1.755907	1.2885156	1.3178921
CHST13	carbohydrate (chondroitin 4) sulfotransferase 13	1.1177727	1.5219588	1.3064629
CHST3	carbohydrate (chondroitin 6) sulfotransferase 3	-1.032112	-3.591276	-12.807855
CHST4	carbohydrate (N-acetylglucosamine 6-O) sulfotransferase 4	1.2683342	3.3789537	1.2396909
CHST6	carbohydrate (N-acetylglucosamine 6-O) sulfotransferase 6	1.3213011	2.2025726	2.9472008
CHST8	carbohydrate (N-acetylgalactosamine 4-0) sulfotransferase 8	2.670544	2.161785	4.803539
CHTF18	CTF18, chromosome transmission fidelity factor 18 homolog (S. cerevisiae)	-1.0912235	-4.204019	-4.509773
CHUK	conserved helix-loop-helix ubiquitous kinase	1.1090704	1.0957721	1.6413753
CIAO1	cytosolic iron-sulfur assembly component 1	1.2685773	1.6487049	1.4878204
CIB2	calcium and integrin binding family member 2	-1.2597339	-1.5823063	-10.941241
CIC	capicua transcriptional repressor	-1.6560915	-1.8998448	-2.5543103
CIDEB	cell death-inducing DFFA-like effector b	-2.4742587	4.890767	6.3047237
CIDEC	cell death-inducing DFFA-like effector c	3.8660653	10.987448	3.6497228
CIDEC	cell death-inducing DFFA-like effector c	4.8520656	12.627512	4.4710555
CIDECP	cell death-inducing DFFA-like effector c pseudogene	2.415475	6.8442607	2.3433437
CINP	cyclin-dependent kinase 2 interacting protein	1.5175719	1.5894195	1.8999557
CIRH1A	cirrhosis, autosomal recessive 1A (cirhin)	1.0925388	-1.0930967	-1.7153254
CISD1	CDGSH iron sulfur domain 1	1.7006962	1.8869568	1.5043032
CISH	cytokine inducible SH2-containing protein	2.5486124	3.086672	2.4244788
CIT	citron rho-interacting serine/threonine kinase	1.71386	-3.0079112	-3.1955786
CITED4	Cbp/p300-interacting transactivator, with Glu/Asp-rich carboxy-terminal domain, 4	1.252796	2.51842	2.0312154
CKAP2	cytoskeleton associated protein 2	2.5913517	-1.7918029	-1.422613
CKAP4	cytoskeleton-associated protein 4	1.2048625	-1.6957862	-1.8128588
CKAP5	cytoskeleton associated protein 5	-1.7370067	-2.2859778	-3.1325881
CKB	creatine kinase, brain	1.2026523	2.6049654	1.4250884
CKLF	chemokine-like factor	-2.0606003	-3.2501602	-3.4668021
CKLF	chemokine-like factor	-1.9446975	-3.0279944	-4.0986753

CKMT1A	creatine kinase, mitochondrial 1A	1.6010829	1.8063093	-2.9358153
CKS2	CDC28 protein kinase regulatory subunit 2	1.894472	-2.2869017	-2.672383
CLASP1	cytoplasmic linker associated protein 1	-1.1918346	-1.6646575	-1.7031442
CLCC1	chloride channel CLIC-like 1	-1.6941466	-1.6932598	-1.4760804
CLCN5	chloride channel, voltage-sensitive 5	-1.4441309	1.9320135	2.409418
CLCN7	chloride channel, voltage-sensitive 7	-1.4115779	-1.6352975	-1.8812891
CLDN1	claudin 1	-1.8913691	1.2516149	1.121578
CLDN14	claudin 14	-5.912258	1.3662357	13.0262165
CLDN19	claudin 19	2.1171305	1.8270577	1.915919
CLDN23	claudin 23	-1.1669009	1.1163478	1.7468224
CLDN3	claudin 3	-1.8641944	2.72081	15.334921
CLDN7	claudin 7	1.0726764	2.853337	2.0111399
CLDND1	claudin domain containing 1	-1.108093	-1.5892925	-1.5532172
CLEC16A	C-type lectin domain family 16, member A	1.4146675	1.3391455	1.546635
CLEC2D	C-type lectin domain family 2, member D	-1.1659534	-1.6791623	-2.494746
CLEC2D	C-type lectin domain family 2, member D	1.2424462	-1.1210663	-1.5880579
CLEC3B	C-type lectin domain family 3, member B	9.095128	5.700311	3.5558321
CLGN	calmegin	-3.8436713	-2.996273	-2.4107933
CLHC1	clathrin heavy chain linker domain containing 1	1.8280282	2.020829	2.349465
CLIC1	chloride intracellular channel 1	-1.0963238	-1.5739883	-3.5610087
CLIC4	chloride intracellular channel 4	1.011794	-1.5848572	-1.3074266
CLIC4	chloride intracellular channel 4	-1.2390488	-1.9796971	-2.0311267
CLIC5	chloride intracellular channel 5	1.1810503	-1.2040701	-2.576003
CLIP2	CAP-GLY domain containing linker protein 2	-2.9878306	-2.4577394	-2.1818388
CLIP4	CAP-GLY domain containing linker protein family, member 4	1.0152673	-1.9726558	-1.9518944
CLK1	CDC-like kinase 1	-2.4742682	-1.9847353	-1.4487748
CLMN	calmin (calponin-like, transmembrane)	-2.0589402	1.237987	3.476541
CLMN	calmin (calponin-like, transmembrane)	-1.4014277	1.2244462	2.144951
CLN6	ceroid-lipofuscinosis, neuronal 6, late infantile, variant	-1.1282947	-1.5147815	-1.8797468
CLPB	ClpB caseinolytic peptidase B homolog (E. coli)	-1.5598016	-1.4511557	1.0371383
CLRN1-AS1	CLRN1 antisense RNA 1	1.5683764	1.6898474	1.7227061
CLRN3	clarin 3	-5.2784452	4.108323	4.18871
CLSTN1	calsyntenin 1	-1.4590651	-1.8532078	-1.4769328
CLSTN3	calsyntenin 3	-1.1844293	1.9688845	2.94825
CLTCL1	clathrin, heavy chain-like 1	1.0559043	-1.3918802	-1.8255965
CLU	clusterin	-4.5655417	3.008868	6.670684
CLUAP1	clusterin associated protein 1	1.5969481	1.6279776	1.4152181
CLUAP1	clusterin associated protein 1	1.5239877	1.5039611	1.4170508
CLYBL	citrate lyase beta like	1.0516214	2.1315587	3.0213428
CMC1	C-x(9)-C motif containing 1	1.7540028	2.0713432	1.4458941
CMC4	C-x(9)-C motif containing 4	1.0888257	1.6901913	1.8065113
CMIP	c-Maf inducing protein	-1.5984637	-2.5093608	-2.8606923
CMTM8	CKLF-like MARVEL transmembrane domain containing 8	1.1067278	2.2863617	3.7520921
CNDP2	CNDP dipeptidase 2 (metallopeptidase M20 family)	-1.8569391	-1.25452	-1.1740925
CNFN	cornifelin	1.9960884	1.4696118	1.1702293
CNGB1	cyclic nucleotide gated channel beta 1	1.7338814	2.2934968	2.069389
CNIH1	cornichon family AMPA receptor auxiliary protein 1	1.4332975	1.5978597	2.0769458
CNKSR3	CNKSR family member 3	1.186642	2.6722317	2.5305548
CNN1	calponin 1, basic, smooth muscle	3.5181763	-18.543142	-104.31815
CNN2	calponin 2	1.2730845	-2.0316865	-4.1174726
CNN3	calponin 3, acidic	1.2434496	1.030808	1.619629
CNNM2	cyclin and CBS domain divalent metal cation transport mediator 2	2.740295	2.5817595	2.5340517
CNNM3	cyclin and CBS domain divalent metal cation transport mediator 3	1.694911	2.3093238	3.7504544
CNOT3	CCR4-NOT transcription complex, subunit 3	1.3091217	1.859615	2.301082
CNOT7	CCR4-NOT transcription complex, subunit 7	-1.2008525	-1.6076324	-1.400268
CNPY3	canopy FGF signaling regulator 3	-1.4025418	-2.297258	-3.0510652
CNST	consortin, connexin sorting protein	1.1820732	1.6533961	1.9574177
CNTNAP2	contactin associated protein-like 2	1.3812597	-1.3466494	-2.4935012
CNTROB	centrobin, centrosomal BRCA2 interacting protein	-1.29591	-1.7723298	-2.127527
COA3	cytochrome c oxidase assembly factor 3	1.0898443	1.8129047	1.7638851
COA4	cytochrome c oxidase assembly factor 4 homolog (S. cerevisiae)	1.2332256	1.804588	1.6411347
COA5	cytochrome c oxidase assembly factor 5	-1.056162	1.0645634	1.6515224
COBL	cordon-bleu WH2 repeat protein	-1.4211668	1.9533107	3.626483
COBLL1	cordon-bleu WH2 repeat protein-like 1	1.5198507	3.4948869	2.8166063
COG8	component of oligomeric golgi complex 8	1.6964303	1.8121651	2.098975
COG8	component of oligomeric golgi complex 8	1.4607385	1.4168557	2.2235985
COL11A2	collagen, type XI, alpha 2	3.034697	3.3594804	3.7465827
COL12A1	collagen, type XII, alpha 1	1.2155381	-1.9644902	-2.8117466
COL16A1	collagen, type XVI, alpha 1	-4.758272	-5.5628285	-11.371243
COL16A1	collagen, type XVI, alpha 1	3.3625803	1.3005933	1.1787717
COL18A1	collagen, type XVIII, alpha 1	-2.0748255	-1.225011	1.7042234
COL4A1	collagen, type IV, alpha 1	-1.2342097	-6.3879714	-7.7307878
COL4A2	collagen, type IV, alpha 2	1.3780228	-2.9496856	-3.2505398
COL4A3BP	collagen, type IV, alpha 3 (Goodpasture antigen) binding protein	-1.1120955	1.0689802	1.6654879
COL4A3BP	collagen, type IV, alpha 3 (Goodpasture antigen) binding protein	1.004578	1.3873956	1.885063
COL4A4	collagen, type IV, alpha 4	2.639108	-1.1236005	-1.5680656
COL4A5	collagen, type IV, alpha 5	-1.1162006	-1.9738555	-2.5349722
COL4A6	collagen, type IV, alpha 6	1.1480364	-1.3923446	-4.948545
COL5A1	collagen, type V, alpha 1	-1.1863747	-6.3229795	-15.42274
COL5A1	collagen, type V, alpha 1	-1.9203714	-7.7270374	-16.047363
COL8A1	collagen, type VIII, alpha 1	4.732777	1.6219342	1.038287
COL8A2	collagen, type VIII, alpha 2	12.840017	3.939089	2.805165
COLGALT1	collagen beta(1-O)galactosyltransferase 1	-1.8003863	-4.0422835	-4.446592
COMMD3	COMM domain containing 3	1.060728	1.1526152	1.5519263
COMMD4	COMM domain containing 4	-1.002763	-1.4850869	-1.7261515
COMMD8	COMM domain containing 8	1.1512665	-1.3321146	-1.6310799
COMMD8	COMM domain containing 8	-1.188245	-2.0343587	-2.1236906
COMTD1	catechol-O-methyltransferase domain containing 1	1.2969997	1.3469257	-1.8981621
COPA	coatomer protein complex, subunit alpha	1.3095641	1.6906202	1.2688725
COPZ2	coatomer protein complex, subunit zeta 2	1.5513093	1.470164	1.6709468
COQ10A	coenzyme Q10 homolog A (S. cerevisiae)	1.5961852	1.9265724	1.4878742
COQ5	coenzyme Q5 homolog, methyltransferase (S. cerevisiae)	-1.1285827	1.1843137	1.7981714
COQ5	coenzyme Q5 homolog, methyltransferase (S. cerevisiae)	-1.1201888	1.248066	1.7972971
COQ9	coenzyme Q9	1.0706229	1.7568161	1.4897848
CORO1C	coronin, actin binding protein, 1C	1.2570112	-2.3863559	-2.5078218
CORO2A	coronin, actin binding protein, 2A	-1.1493592	1.0480953	1.7102889
CORT	cortistatin	1.4170182	1.7795024	2.0212545
COX11	COX11 cytochrome c oxidase copper chaperone	1.4122218	1.3046763	1.7639141
COX11	COX11 cytochrome c oxidase copper chaperone	1.3973408	1.9362816	2.7711966
COX14	COX14 cytochrome c oxidase assembly factor	1.4655406	2.0432677	2.462154
COX20	COX20 cytochrome c oxidase assembly factor	1.8480191	2.0718112	1.4734452
COX5A	cytochrome c oxidase subunit Va	2.665325	2.9361322	2.0318253
COX5A	cytochrome c oxidase subunit Va	2.3487399	2.79708	1.9468224
COX5B	cytochrome c oxidase subunit Vb	1.9773281	2.6170325	1.8328434
COX6A1	cytochrome c oxidase subunit VIa polypeptide 1	1.6079077	1.9697652	2.037002
COX6A2	cytochrome c oxidase subunit VIa polypeptide 2	-1.2899123	2.8302774	4.0639954
COX6B1	cytochrome c oxidase subunit VIb polypeptide 1 (ubiquitous)	1.720376	2.0666187	1.4207228
COX6B2	cytochrome c oxidase subunit VIb polypeptide 2 (testis)	2.2501004	2.4252121	2.481864
COX6C	cytochrome c oxidase subunit VIc	1.7555312	2.3489647	2.0857222

COX7A2	cytochrome c oxidase subunit VIIa polypeptide 2 (liver)	1.5928172	1.8978755	1.6532571
COX7C	cytochrome c oxidase subunit VIIc	1.9584175	2.3115964	2.0427198
COX8A	cytochrome c oxidase subunit VIIIA (ubiquitous)	1.6173288	1.8538613	1.5615343
CP	ceruloplasmin (ferroxidase)	-5.7404237	-1.5760769	-5.5566916
CP	ceruloplasmin (ferroxidase)	-4.9832244	-1.2481579	-4.3705735
CPB2	carboxypeptidase B2 (plasma)	-2.8909283	7.4373	14.527816
CPEB4	cytoplasmic polyadenylation element binding protein 4	-1.4086871	-1.1958268	1.7408245
CPNE1	copine I	1.5116192	1.4493002	1.4184382
CPNE2	copine II	-2.0086832	-1.7821851	-3.5199742
CPS1	carbamoyl-phosphate synthase 1, mitochondrial	-1.7923465	9.956891	5.33895
CPSF3	cleavage and polyadenylation specific factor 3, 73kDa	-1.0075523	-1.4227062	-1.6742964
CPSF4	cleavage and polyadenylation specific factor 4, 30kDa	1.1223373	-1.6764181	-1.2716686
CPSF6	cleavage and polyadenylation specific factor 6, 68kDa	-1.1571971	-1.8432345	-1.9243
CPT1A	carnitine palmitoyltransferase 1A (liver)	-2.4533532	-1.763854	-1.7675999
CPT2	carnitine palmitoyltransferase 2	-1.2220856	1.591337	2.1305716
CPTP	ceramide-1-phosphate transfer protein	1.6941252	2.5561168	1.8817432
CPTP	ceramide-1-phosphate transfer protein	1.1895328	1.9082121	1.4645299
CPTP	ceramide-1-phosphate transfer protein	1.5737517	2.4193902	1.810455
CRACR2A	calcium release activated channel regulator 2A	2.3026912	2.2595308	4.22612
CRACR2B	calcium release activated channel regulator 2B	2.0671413	1.904591	1.2885733
CRADD	CASP2 and RIPK1 domain containing adaptor with death domain	1.2612233	1.981494	2.7073424
CRAT	carnitine O-acetyltransferase	1.2547319	2.2647989	2.794891
CRB3	crumbs family member 3	1.4638846	3.1586456	1.7983582
CRBN	cereblon	1.3366796	1.7962055	2.2995913
CREB3	cAMP responsive element binding protein 3	-1.428761	-1.4976085	-1.804149
CREB3L1	cAMP responsive element binding protein 3-like 1	1.6531239	1.5011146	1.6477158
CREB3L2	cAMP responsive element binding protein 3-like 2	-1.0810413	-1.5092238	-1.8802394
CREB3L3	cAMP responsive element binding protein 3-like 3	-18.090755	-1.6695715	-1.3004313
CREBRF	CREB3 regulatory factor	-2.5652647	-1.1002891	1.0651023
CREG1	cellular repressor of E1A-stimulated genes 1	1.068008	1.1216893	1.952231
CRELD1	cysteine-rich with EGF-like domains 1	-1.1086886	-1.1747398	1.8039122
CRIP2	cysteine-rich protein 2	1.1477237	1.7456182	-2.0979228
CRIPAK	cysteine-rich PAK1 inhibitor	-2.1372688	-1.4867061	-1.9018003
CRIPT	cysteine-rich PDZ-binding protein	1.2131879	1.3368009	1.7697966
CRLS1	cardiolipin synthase 1	1.1521487	1.7574743	2.4192357
CROCC	ciliary rootlet coiled-coil, rootletin	-1.6377535	-1.9647602	-1.7645078
CRTC1	CREB regulated transcription coactivator 1	1.9041599	2.0261219	2.6708002
CRTC1	CREB regulated transcription coactivator 1	-1.1994191	-1.0760968	1.8462846
CRY2	cryptochrome circadian clock 2	-1.3039706	2.1212373	3.0376375
CRYAA	crystallin, alpha A	1.4102181	1.6370602	2.8405485
CRYAB	crystallin, alpha B	2.7260797	4.3109674	1.7713602
CRYBB2P1	crystallin, beta B2 pseudogene 1	-3.364508	-2.7153044	-2.7525063
CRYL1	crystallin, lambda 1	-1.2614932	3.3965573	5.645746
CRYM	crystallin, mu	-1.1018332	3.6065943	3.3439903
CRYZ	crystallin, zeta (quinone reductase)	-1.7473174	1.7892275	2.0018666
CRYZ	crystallin, zeta (quinone reductase)	-2.090632	1.4927287	1.5307416
CS	citrate synthase	2.1440587	2.3796055	1.1838055
CSAG1	chondrosarcoma associated gene 1	1.2773167	2.148741	1.156996
CSE1L	CSE1 chromosome segregation 1-like (yeast)	1.056408	-2.0573835	-1.612896
CSE1L	CSE1 chromosome segregation 1-like (yeast)	1.1408206	-1.9143561	-1.4201016
CSF1	colony stimulating factor 1 (macrophage)	1.5360553	1.1826133	-1.4257187
CSNK1D	casein kinase 1, delta	-1.1833433	-1.5828382	-1.4802557
CSNK1G2	casein kinase 1, gamma 2	-1.302782	-1.6266683	-1.9440022
CSNK2A1	casein kinase 2, alpha 1 polypeptide	-1.4005316	-1.6413678	-1.8794357
CSRP2	cysteine and glycine-rich protein 2	2.3913243	4.255669	2.7205634
CSRP2BP	CSRP2 binding protein	1.8384513	2.396782	3.2406664
CST3	cystatin C	-1.061222	1.1025677	2.0444593
CST3	cystatin C	1.0192925	2.068407	2.3543186
CST5	cystatin D	-1.0001049	2.2321343	2.588097
CSTB	cystatin B (stefin B)	1.3253342	1.2897563	1.5808562
CSTF1	cleavage stimulation factor, 3' pre-RNA, subunit 1, 50kDa	2.0336046	1.8605179	2.0517232
CSTF2	cleavage stimulation factor, 3' pre-RNA, subunit 2, 64kDa	-1.0558984	-1.4199477	-1.6759435
CSTF2T	cleavage stimulation factor, 3' pre-RNA, subunit 2, 64kDa, tau variant	2.1439183	2.5954115	2.5466504
CT45A1	cancer/testis antigen family 45, member A1	2.6297352	3.36044	2.388881
CT47A11	cancer/testis antigen family 47, member A11	2.2374501	1.7971752	2.0099967
CT55	cancer/testis antigen 55	2.0484402	2.216116	1.245133
CTAGE11P	CTAGE family, member 11, pseudogene	-1.9383202	-1.4964616	-1.0083423
CTAGE4	CTAGE family, member 4	-2.5096505	-2.0394933	-1.479412
CTAGE4	CTAGE family, member 4	-1.9431114	-1.5734855	-1.1222893
CTAGE5	CTAGE family, member 5	-1.5975108	-1.571617	-1.1888952
CTAGE5	CTAGE family, member 5	-2.6235006	-1.470884	1.0572916
CTBP2	C-terminal binding protein 2	-1.3244877	-2.0932083	-5.3289075
CTD-3080P12.3	uncharacterized LOC101928857	2.0594106	6.6134534	21.413656
CTDSP1	CTD (carboxy-terminal domain, RNA polymerase II, polypeptide A) small phosphatase 1	1.1265858	1.7032932	2.8133469
CTDSP2	CTD (carboxy-terminal domain, RNA polymerase II, polypeptide A) small phosphatase 2	1.1154397	1.4482769	2.2085094
CTDSPL	CTD (carboxy-terminal domain, RNA polymerase II, polypeptide A) small phosphatase-like	1.7110085	2.2095172	1.0794902
CTH	cystathionine gamma-lyase	-2.2269816	1.1161745	3.3157878
CTNNA1	catenin (cadherin-associated protein), alpha 1, 102kDa	-2.437477	-2.1159863	-2.7167146
CTNNAL1	catenin (cadherin-associated protein), alpha-like 1	1.503063	-1.3488358	-1.4116952
CTNNBIP1	catenin, beta interacting protein 1	1.3132148	1.9539189	2.2626965
CTPS1	CTP synthase 1	1.2581117	-1.9140486	-1.0392673
CTPS1	CTP synthase 1	1.0189778	-1.966889	1.0082618
CTRB2	chymotrypsinogen B2	1.7419684	2.058874	2.4167178
CTSC	cathepsin C	-1.5439397	-1.742942	-3.131185
CTSC	cathepsin C	1.0394847	-2.5074594	-5.7055306
CTSD	cathepsin D	-1.7151495	-1.6613487	-3.16238
CTSF	cathepsin F	1.0816157	2.0135891	2.6507003
CTSH	cathepsin H	-1.5317148	-1.5206323	-2.0674367
CTSO	cathepsin O	-1.317108	1.8551688	2.5281785
CTTN	cortactin	1.1676903	-1.167439	-1.7041916
CTXN1	cortexin 1	1.8535988	1.6124427	1.5581589
CUEDC1	CUE domain containing 1	-1.6588018	-1.9804832	-2.4536715
CUL4B	cullin 4B	-1.8226581	-1.8608074	-2.6204917
CUL5	cullin 5	1.27731	1.4467076	2.040594
CUL7	cullin 7	-2.0715787	-1.4120731	-1.8783299
CUL9	cullin 9	-1.6057938	-1.5686412	-1.802116
CUX1	cut-like homeobox 1	-1.5271773	-1.4634708	1.094981
CXADR	coxsackie virus and adenovirus receptor	-2.4454663	-1.4550316	1.4903141
CXCL1	chemokine (C-X-C motif) ligand 1 (melanoma growth stimulating activity, alpha)	-6.3213105	-6.044685	-35.31663
CXCL1	chemokine (C-X-C motif) ligand 1 (melanoma growth stimulating activity, alpha)	-8.12354	-6.773049	-21.512411
CXCL13	chemokine (C-X-C motif) ligand 13	14.325319	335.64423	185.18208
CXCL16	chemokine (C-X-C motif) ligand 16	1.299065	1.8523012	1.649237
CXCL2	chemokine (C-X-C motif) ligand 2	-4.8763356	-1.7817101	-2.7513943
CXCL2	chemokine (C-X-C motif) ligand 2	-6.6285725	-3.1890292	-6.0178223
CXCL5	chemokine (C-X-C motif) ligand 5	1.6769791	1.5721447	2.1683848
CXCL8	chemokine (C-X-C motif) ligand 8	-11.132999	-7.7998614	-35.283775
CXCR3	chemokine (C-X-C motif) receptor 3	1.9376357	2.6094756	1.4200501
CXorf67	chromosome X open reading frame 67	2.2482069	1.6754899	1.2611398
CXXC1	CXXC finger protein 1	-1.2543795	-1.485858	-1.7830619

CXXC5	CXXC finger protein 5	-1.8309767	-1.8065368	-1.4925618
CYB561	cytochrome b561	-1.3156121	-1.3260235	-1.6999495
CYB5A	cytochrome b5 type A (microsomal)	1.8041271	3.4734194	7.0910864
CYB5A	cytochrome b5 type A (microsomal)	1.5682957	3.0289798	6.329775
CYB5D1	cytochrome b5 domain containing 1	1.2363693	1.7046632	2.4244812
CYB5D2	cytochrome b5 domain containing 2	1.1084055	1.9672076	2.538496
CYB5R3	cytochrome b5 reductase 3	1.5501513	1.6392431	1.7902279
CYBRD1	cytochrome b reductase 1	1.6321092	1.7727197	2.1091373
CYBRD1	cytochrome b reductase 1	1.5584577	1.0401211	1.0725505
CYC1	cytochrome c-1	1.7261018	2.2007873	1.7170974
CYCS	cytochrome c, somatic	2.0080855	1.5611702	1.1443367
CYCS	cytochrome c, somatic	2.234094	2.5181763	1.1453497
CYCS	cytochrome c, somatic	2.3603597	1.7773719	1.275681
CYGB	cytoglobin	1.5094378	1.8320063	1.5114422
CYHR1	cysteine/histidine-rich 1	1.1974095	1.0336767	1.7893311
CYHR1	cysteine/histidine-rich 1	1.187951	2.0349112	2.549638
CYP17A1-AS1	CYP17A1 antisense RNA 1	1.0370861	1.2411247	3.0236363
CYP1A1	cytochrome P450, family 1, subfamily A, polypeptide 1	-137.13994	-39.035984	-24.97008
CYP1A2	cytochrome P450, family 1, subfamily A, polypeptide 2	-1.2486259	1.0566249	5.357575
CYP1B1	cytochrome P450, family 1, subfamily B, polypeptide 1	-5.1126485	-11.079464	-30.453012
CYP1B1	cytochrome P450, family 1, subfamily B, polypeptide 1	-6.429503	-12.4984865	-36.412727
CYP20A1	cytochrome P450, family 20, subfamily A, polypeptide 1	-1.6316153	-1.1149001	1.1628317
CYP27A1	cytochrome P450, family 27, subfamily A, polypeptide 1	1.8031093	2.8967757	4.522722
CYP2A13	cytochrome P450, family 2, subfamily A, polypeptide 13	1.7989879	2.1706393	41.885365
CYP2A7	cytochrome P450, family 2, subfamily A, polypeptide 7	-1.7136301	13.869389	616.00854
CYP2B6	cytochrome P450, family 2, subfamily B, polypeptide 6	2.3478625	2.3251987	3.619344
CYP2B6	cytochrome P450, family 2, subfamily B, polypeptide 6	-2.5793314	5.7021165	148.71121
CYP2C18	cytochrome P450, family 2, subfamily C, polypeptide 18	-14.429745	2.2014644	7.8593254
CYP2C19	cytochrome P450, family 2, subfamily C, polypeptide 19	-17.284618	9.668302	150.8676
CYP2C8	cytochrome P450, family 2, subfamily C, polypeptide 8	-9.2389345	9.250023	49.632656
CYP2C9	cytochrome P450, family 2, subfamily C, polypeptide 9	-25.8377	7.349837	134.69911
CYP2E1	cytochrome P450, family 2, subfamily E, polypeptide 1	-3.1677294	4.4438415	356.4329
CYP2J2	cytochrome P450, family 2, subfamily J, polypeptide 2	-1.5861044	-1.0214589	2.7727096
CYP2U1	cytochrome P450, family 2, subfamily U, polypeptide 1	2.4838924	3.1625712	4.369445
CYP3A4	cytochrome P450, family 3, subfamily A, polypeptide 4	-1.3594817	13.349323	672.0539
CYP3A5	cytochrome P450, family 3, subfamily A, polypeptide 5	-2.121687	1.7984998	7.646847
CYP3A5	cytochrome P450, family 3, subfamily A, polypeptide 5	-2.9056396	2.4377923	11.34953
CYP3A7	cytochrome P450, family 3, subfamily A, polypeptide 7	-2.1112318	2.576385	31.756643
CYP3A7	cytochrome P450, family 3, subfamily A, polypeptide 7	1.0189486	8.89915	146.91464
CYP4A11	cytochrome P450, family 4, subfamily A, polypeptide 11	-2.6902902	8.572263	7.7510242
CYP4B1	cytochrome P450, family 4, subfamily B, polypeptide 1	54.314075	596.8844	934.1175
CYP4F11	cytochrome P450, family 4, subfamily F, polypeptide 11	1.1160161	1.5902281	2.572352
CYP4F12	cytochrome P450, family 4, subfamily F, polypeptide 12	-1.854313	1.4134145	3.8231473
CYP4F2	cytochrome P450, family 4, subfamily F, polypeptide 2	1.380559	45.455544	331.98257
CYP4F2	cytochrome P450, family 4, subfamily F, polypeptide 2	-1.9110625	1.9676148	8.360191
CYP4F22	cytochrome P450, family 4, subfamily F, polypeptide 22	4.3831644	8.914655	1.8007952
CYP4F3	cytochrome P450, family 4, subfamily F, polypeptide 3	-1.9286395	4.7277904	32.9329
CYP4F62P	cytochrome P450, family 4, subfamily F, polypeptide 62, pseudogene	1.4457008	2.1297238	2.1963875
CYP4F8	cytochrome P450, family 4, subfamily F, polypeptide 8	-1.6344658	1.4901028	4.513739
CYP51A1	cytochrome P450, family 51, subfamily A, polypeptide 1	1.3769624	1.506243	1.9427145
CYP7B1	cytochrome P450, family 7, subfamily B, polypeptide 1	-1.0398376	1.7323856	1.4933965
CYP8B1	cytochrome P450, family 8, subfamily B, polypeptide 1	-1.2712159	89.83964	596.432
CYR61	cysteine-rich, angiogenic inducer, 61	1.9913793	-2.332736	-1.7500885
CYSTM1	cysteine-rich transmembrane module containing 1	-1.8421738	1.113162	1.3890892
CYTH2	cytohesin 2	-1.5061922	-1.8448883	-1.786498
CYTH3	cytohesin 3	2.452585	1.2982184	1.1142213
D2HGDH	D-2-hydroxyglutarate dehydrogenase	-1.5835409	-1.2338105	1.0264226
DAB2	Dab, mitogen-responsive phosphoprotein, homolog 2 (Drosophila)	2.5402339	2.4129066	1.1753908
DACH1	dachshund family transcription factor 1	3.9211516	5.65732	3.0529053
DACT1	dishevelled-binding antagonist of beta-catenin 1	-1.6006747	-6.2540083	-8.300805
DAK	dihydroxyacetone kinase 2 homolog (S. cerevisiae)	1.6450464	2.2470136	1.8834229
DAK	dihydroxyacetone kinase 2 homolog (S. cerevisiae)	1.2464228	2.056007	2.9673643
DAO	D-amino-acid oxidase	-1.5169164	13.191707	73.45344
DAPK2	death-associated protein kinase 2	-2.320279	-1.0670233	1.451742
DAXX	death-domain associated protein	-1.7205328	-1.6794211	-2.3734353
DAZAP1	DAZ associated protein 1	1.558847	-1.002779	-1.4056342
DAZAP2	DAZ associated protein 2	1.4999741	1.4179577	2.0193396
DBH-AS1	DBH antisense RNA 1	-2.206702	1.5143163	1.9441673
DBI	diazepam binding inhibitor (GABA receptor modulator, acyl-CoA binding protein)	2.021191	2.4840164	2.926146
DBN1	drebrin 1	1.1096692	-1.1189867	-4.3016977
DBNL	drebrin-like	-1.2019287	-1.4930153	-2.651981
DBT	dihydrolipoamide branched chain transacylase E2	1.0136471	2.367501	3.0614314
DCAF11	DDB1 and CUL4 associated factor 11	1.0774034	2.0041745	6.120498
DCAF13	DDB1 and CUL4 associated factor 13	1.0991002	-1.2758895	-1.9956903
DCAF16	DDB1 and CUL4 associated factor 16	1.5174822	1.5239154	1.0823663
DCAF4L2	DDB1 and CUL4 associated factor 4-like 2	1.5280684	2.2089252	2.4077878
DCAF6	DDB1 and CUL4 associated factor 6	1.2693936	1.3552266	2.1196113
DCBLD1	discoidin, CUB and LCCL domain containing 1	-1.2997164	-4.503789	-6.394725
DCBLD2	discoidin, CUB and LCCL domain containing 2	-1.2603257	-3.4985251	-3.3400617
DCBLD2	discoidin, CUB and LCCL domain containing 2	1.04117	-2.8345816	-2.5183325
DCBLD2	discoidin, CUB and LCCL domain containing 2	-1.2539617	-3.1860673	-3.3449361
DCLK1	doublecortin-like kinase 1	-2.1774116	-1.7121742	-3.810714
DCN	decorin	5.839832	3.0241132	-2.7312632
DCN	decorin	6.0561247	3.0459168	-2.5706513
DCPS	decapping enzyme, scavenger	1.5485408	1.0382994	-1.1992054
DCTN1	dynactin 1	-1.3294923	-1.5225632	-2.0326831
DCUN1D1	DCN1, defective in cullin neddylation 1, domain containing 1	1.0189302	1.2391644	1.9185079
DCUN1D5	DCN1, defective in cullin neddylation 1, domain containing 5	1.6569407	1.0333736	-1.104973
DCXR	dicarbonyl/L-xylulose reductase	1.3656232	4.3394465	17.8408
DDA1	DET1 and DDB1 associated 1	-1.2367702	-1.8113431	-2.1949816
DDAH1	dimethylarginine dimethylaminohydrolase 1	2.1203263	2.0597234	3.542571
DDB2	damage-specific DNA binding protein 2, 48kDa	-1.1783701	-1.6358054	-2.8345432
DDB2	damage-specific DNA binding protein 2, 48kDa	-1.5067147	-2.0983438	-4.410119
DDC	dopa decarboxylase (aromatic L-amino acid decarboxylase)	1.2608485	5.3897185	5.1415424
DDIAS	DNA damage-induced apoptosis suppressor	1.9032537	-2.1229343	-3.7679765
DDIT3	DNA-damage-inducible transcript 3	-2.9029853	-1.9017397	-1.5567404
DDR1	discoidin domain receptor tyrosine kinase 1	-1.0519408	-1.186325	-3.075886
DDT	D-dopachrome tautomerase	1.5650382	3.3747132	3.641952
DDTL	D-dopachrome tautomerase-like	2.2114072	5.5494127	7.3260784
DDX10	DEAD (Asp-Glu-Ala-Asp) box polypeptide 10	-1.406272	-1.6712774	-1.9413596
DDX11	DEAD/H (Asp-Glu-Ala-Asp/His) box helicase 11	-1.277793	-2.278936	-2.5091963
DDX18	DEAD (Asp-Glu-Ala-Asp) box polypeptide 18	1.7073717	1.8493463	1.6360328
DDX19A	DEAD (Asp-Glu-Ala-Asp) box polypeptide 19A	1.7188313	1.6391706	1.8428745
DDX23	DEAD (Asp-Glu-Ala-Asp) box polypeptide 23	-1.2895225	-1.5513273	-1.4176975
DDX39A	DEAD (Asp-Glu-Ala-Asp) box polypeptide 39A	1.0079913	-1.8949147	-2.8534546
DDX3X	DEAD (Asp-Glu-Ala-Asp) box helicase 3, X-linked	-1.4018099	-2.1302452	-1.753251
DDX42	DEAD (Asp-Glu-Ala-Asp) box helicase 42	-1.3220302	-1.5307449	-1.5871497
DDX5	DEAD (Asp-Glu-Ala-Asp) box helicase 5	-1.3414073	-2.044127	-1.4091746
DDX51	DEAD (Asp-Glu-Ala-Asp) box polypeptide 51	2.0751765	2.1673532	2.5937428

DDX55	DEAD (Asp-Glu-Ala-Asp) box polypeptide 55	-1.4764787	-1.7695882	-1.9443201
DEAF1	DEAF1 transcription factor	-1.8377388	-1.6186659	-1.7648027
DECR1	2,4-dienoyl CoA reductase 1, mitochondrial	1.3302928	2.5028841	3.2606287
DECR2	2,4-dienoyl CoA reductase 2, peroxisomal	1.008723	2.0501146	2.3309028
DECR2	2,4-dienoyl CoA reductase 2, peroxisomal	-1.2085615	1.7441492	1.9444976
DEDD2	death effector domain containing 2	-1.6434282	-1.0632563	1.0513334
DEF8	differentially expressed in FDCP 8 homolog (mouse)	1.3679135	-1.5695617	-2.3778582
DEF8	differentially expressed in FDCP 8 homolog (mouse)	1.1682186	-1.9691032	-3.3037786
DEFB1	defensin, beta 1	-5.8603864	1.3302137	1.0437162
DEFB132	defensin, beta 132	1.2607455	9.710199	3.4302192
DEGS2	delta(4)-desaturase, sphingolipid 2	2.0612464	1.6846787	1.5747092
DEK	DEK proto-oncogene	1.5662794	-1.5152051	-1.3602746
DENND2D	DENN/MADD domain containing 2D	-1.5543491	-1.2882379	-5.0739126
DENND4B	DENN/MADD domain containing 4B	-1.4197409	-1.6962926	-1.8229975
DENND5A	DENN/MADD domain containing 5A	1.0427446	-1.5116789	-1.6841997
DENND5B	DENN/MADD domain containing 5B	-1.5141352	1.0898689	1.4979339
DEPDC1	DEP domain containing 1	2.0898514	-3.3834891	-4.7666397
DEPDC7	DEP domain containing 7	1.257253	1.4517303	2.349849
DEPTOR	DEP domain containing MTOR-interacting protein	1.9786057	1.599147	3.0236897
DERL1	derlin 1	-1.7984549	-1.8008883	-1.2855135
DERL3	derlin 3	1.4012804	1.5529728	1.5933853
DEXI	Dexi homolog (mouse)	1.9037156	2.9424236	4.4928865
DFNA5	deafness, autosomal dominant 5	-1.255746	-2.6506395	-2.4483635
DGAT1	diacylglycerol O-acyltransferase 1	1.0915045	3.0538902	6.5528355
DGAT1	diacylglycerol O-acyltransferase 1	-1.015114	2.4336553	4.2495885
DGAT2	diacylglycerol O-acyltransferase 2	-2.0697846	2.0069003	10.763304
DGCR2	DiGeorge syndrome critical region gene 2	1.4927057	1.6654996	2.1485865
DGCR6L	DiGeorge syndrome critical region gene 6-like	-1.1166642	-1.0361767	1.6313958
DGKA	diacylglycerol kinase, alpha 80kDa	-1.4690918	-4.062728	-12.0366535
DGKD	diacylglycerol kinase, delta 130kDa	-1.3469907	-3.035764	-2.705845
DGKQ	diacylglycerol kinase, theta 110kDa	-1.4815599	-1.5618529	1.3000373
DGKZ	diacylglycerol kinase, zeta	1.025037	-1.6354914	-1.6876041
DHCR24	24-dehydrocholesterol reductase	1.2423981	1.8092033	3.9400144
DHCR7	7-dehydrocholesterol reductase	1.4301378	1.682441	3.4775581
DHFR	dihydrofolate reductase	1.4066635	-1.9043453	-1.06104
DHODH	dihydroorotate dehydrogenase (quinone)	-1.5110422	1.4659648	1.1811008
DHRS1	dehydrogenase/reductase (SDR family) member 1	-1.3065704	1.0898352	1.7572689
DHRS12	dehydrogenase/reductase (SDR family) member 12	1.0576485	1.9515191	2.2130992
DHRS3	dehydrogenase/reductase (SDR family) member 3	-2.6201272	1.2615519	2.504868
DHRS4	dehydrogenase/reductase (SDR family) member 4	1.2180095	1.5089343	2.4309547
DHRS4	dehydrogenase/reductase (SDR family) member 4	1.1611165	1.3626101	2.51802
DHRS4	dehydrogenase/reductase (SDR family) member 4	1.3480225	1.608198	3.0896406
DHRS4-AS1	DHRS4 antisense RNA 1	1.6539805	2.0115483	3.6616447
DHRS4L1	dehydrogenase/reductase (SDR family) member 4 like 1	1.8439173	1.8844651	2.559285
DHRS4L2	dehydrogenase/reductase (SDR family) member 4 like 2	1.7693417	2.1231017	4.085417
DHRS4L2	dehydrogenase/reductase (SDR family) member 4 like 2	1.1672115	1.4321536	2.8185706
DHRS7	dehydrogenase/reductase (SDR family) member 7	-1.0782168	1.542367	1.6692568
DHRSX	dehydrogenase/reductase (SDR family) X-linked	-1.2372683	1.1374239	1.7882068
DHTKD1	dehydrogenase E1 and transketolase domain containing 1	1.1629946	3.009116	7.402833
DHX16	DEAH (Asp-Glu-Ala-His) box polypeptide 16	-1.5603316	-1.5500519	-1.4372069
DHX58	DEXH (Asp-Glu-X-His) box polypeptide 58	1.423377	2.297615	3.2271404
DHX9	DEAH (Asp-Glu-Ala-His) box helicase 9	-1.3940946	-1.6806867	-2.1497748
DIABLO	diablo, IAP-binding mitochondrial protein	1.3646792	1.6842474	1.985736
DIAPH1	diaphanous-related formin 1	1.1757071	1.5280771	1.6892385
DIAPH3	diaphanous-related formin 3	1.5885687	-4.3576617	-7.1080036
DIRAS1	DIRAS family, GTP-binding RAS-like 1	-1.0294067	1.0017699	-5.7709775
DIRC1	disrupted in renal carcinoma 1	1.7518752	1.9779773	2.0428247
DIS3L	DIS3 like exosome 3'-5' exoribonuclease	1.4665334	1.5234764	1.905586
DISP2	dispatched homolog 2 (Drosophila)	-1.1986548	-2.0197566	-2.2294855
DKFZp779M0652	uncharacterized DKFZp779M0652	1.0613133	3.5962374	2.3815713
DKK1	dickkopf WNT signaling pathway inhibitor 1	9.487145	4.1592174	-1.2502121
DLAT	dihydrolipoamide S-acetyltransferase	1.7450217	2.1046035	1.52116
DLEU1	deleted in lymphocytic leukemia 1 (non-protein coding)	1.876246	1.4934438	1.5855687
DLEU1-AS1	DLEU1 antisense RNA 1	2.6822262	2.988168	1.8387041
DLGAP4	discs, large (Drosophila) homolog-associated protein 4	-1.521284	-1.712138	-2.7351367
DLGAP5	discs, large (Drosophila) homolog-associated protein 5	3.210974	-3.5843918	-4.500173
DLK2	delta-like 2 homolog (Drosophila)	3.5299642	3.753402	-1.3758826
DLX5	distal-less homeobox 5	5.623623	4.008057	3.4317899
DMAP1	DNA methyltransferase 1 associated protein 1	-1.5998875	-1.6653391	-1.5792769
DMGDH	dimethylglycine dehydrogenase	-1.4483976	3.832604	9.34984
DMKN	dermokine	1.3336179	1.3390623	-2.0631118
DMKN	dermokine	1.1090009	1.1226722	-4.029759
DMKN	dermokine	1.1432399	1.1367078	-3.7218125
DMTF1	cyclin D binding myb-like transcription factor 1	-2.4161863	-2.68584	-2.268602
DNAAF5	dynein, axonemal, assembly factor 5	-1.5090842	-1.7297724	-2.0687935
DNAH14	dynein, axonemal, heavy chain 14	-1.3911836	-1.605696	-2.2754333
DNAJA1	DnaJ (Hsp40) homolog, subfamily A, member 1	-1.5619535	-1.7562251	-1.8273215
DNAJA2	DnaJ (Hsp40) homolog, subfamily A, member 2	1.4521109	1.8136312	1.2934257
DNAJB4	DnaJ (Hsp40) homolog, subfamily B, member 4	1.2507306	-2.0022912	-1.7359438
DNAJB5	DnaJ (Hsp40) homolog, subfamily B, member 5	-1.1089163	-1.2317045	-1.5949748
DNAJB6	DnaJ (Hsp40) homolog, subfamily B, member 6	-1.0710367	-1.601989	-1.6532419
DNAJB6	DnaJ (Hsp40) homolog, subfamily B, member 6	-1.0275755	-1.4788828	-1.534313
DNAJB9	DnaJ (Hsp40) homolog, subfamily B, member 9	-2.2480261	1.2066302	1.4481027
DNAJC1	DnaJ (Hsp40) homolog, subfamily C, member 1	-1.7339844	-1.3759503	1.0678331
DNAJC1	DnaJ (Hsp40) homolog, subfamily C, member 1	-1.8163235	-1.6003622	-1.0032252
DNAJC10	DnaJ (Hsp40) homolog, subfamily C, member 10	-1.5442039	-2.2216156	-2.604353
DNAJC13	DnaJ (Hsp40) homolog, subfamily C, member 13	-1.9797077	-2.033736	-2.152153
DNAJC19	DnaJ (Hsp40) homolog, subfamily C, member 19	1.413185	2.3985767	2.4889054
DNAJC2	DnaJ (Hsp40) homolog, subfamily C, member 2	-1.0382527	-1.125051	-1.6702828
DNAJC21	DnaJ (Hsp40) homolog, subfamily C, member 21	1.262488	1.6685828	2.156776
DNAJC25	DnaJ (Hsp40) homolog, subfamily C , member 25	1.2750139	1.9727556	4.0105987
DNAJC3	DnaJ (Hsp40) homolog, subfamily C, member 3	-1.6055542	-1.389152	-1.1556807
DNAJC30	DnaJ (Hsp40) homolog, subfamily C, member 30	1.1684321	1.595946	1.7175517
DNAJC4	DnaJ (Hsp40) homolog, subfamily C, member 4	1.6328617	2.0693889	1.6026071
DNAJC6	DnaJ (Hsp40) homolog, subfamily C, member 6	2.3160067	1.2731122	-1.0050315
DNAJC9	DnaJ (Hsp40) homolog, subfamily C, member 9	1.4606489	-1.5908098	-1.3698839
DNLZ	DNL-type zinc finger	1.0125978	-1.1569185	-1.680287
DNLZ	DNL-type zinc finger	1.0228277	-1.1660544	-2.0032601
DNM1	dynamin 1	1.4895462	1.7830611	2.081327
DNMT1	DNA (cytosine-5-)-methyltransferase 1	1.258584	-1.764194	-1.8229183
DNMT3A	DNA (cytosine-5-)-methyltransferase 3 alpha	-1.2436882	-1.503148	-1.9452012
DNMT3A	DNA (cytosine-5-)-methyltransferase 3 alpha	-2.608487	-2.8670807	-4.107105
DNPH1	2'-deoxynucleoside 5'-phosphate N-hydrolase 1	1.3016999	1.4943801	1.6093335
DNTTIP2	deoxynucleotidyltransferase, terminal, interacting protein 2	-1.2218026	-1.7638583	-1.5737611
DOCK4	dedicator of cytokinesis 4	-1.6809306	1.0918767	-1.666028
DOHH	deoxyhypusine hydroxylase/monooxygenase	-1.2572838	-1.5442858	-1.744354
DOK3	docking protein 3	4.2879863	4.1596313	4.5164695
DOK7	docking protein 7	3.217992	5.095428	1.1200973
DOLPP1	dolichyldiphosphatase 1	1.0994987	1.0666572	1.732778

DONSON	downstream neighbor of SON	1.100356	-1.8614116	-1.6994504
DOT1L	DOT1-like histone H3K79 methyltransferase	1.7290211	1.7501155	2.0382395
DPAGT1	dolichyl-phosphate (UDP-N-acetylglucosamine) N-acetylglucosaminephosphotransferase 1 (G	-1.6909031	-1.6960326	-1.9527315
DPEP3	dipeptidase 3	2.3799524	2.891893	3.2524743
DPM3	dolichyl-phosphate mannosyltransferase polypeptide 3	1.4475837	1.8270006	1.5242536
DPP7	dipeptidyl-peptidase 7	-1.6932405	-1.2857908	-1.3904618
DPP9	dipeptidyl-peptidase 9	1.747157	1.150531	1.0983249
DPT	dermatopontin	3.3731227	2.542702	1.6153789
DPY19L1	dpy-19-like 1 (C. elegans)	-1.4211048	-2.5550647	-1.8425177
DPY19L1	dpy-19-like 1 (C. elegans)	-1.6355523	-2.6746452	-1.681958
DPYD	dihydropyrimidine dehydrogenase	-1.0914186	1.947547	2.4842174
DPYSL3	dihydropyrimidinase-like 3	-2.49782	-7.0871606	-12.487409
DPYSL4	dihydropyrimidinase-like 4	1.4673145	1.7045133	2.0850134
DRAM1	DNA-damage regulated autophagy modulator 1	-1.3953032	-2.4947946	-5.506224
DSE	dermatan sulfate epimerase	1.444716	-1.7576178	-5.6024923
DSG2	desmoglein 2	-1.7362454	-2.9689784	-5.3658986
DSN1	DSN1, MIS12 kinetochore complex component	-1.0137706	-3.1226377	-2.6859875
DSP	desmoplakin	-2.270509	-1.0432539	-1.5403174
DSP	desmoplakin	-2.8528087	-1.4133377	-2.1324885
DST	dystonin	1.3063815	1.5805428	1.9516757
DSTN	destrin (actin depolymerizing factor)	2.3054483	1.2589358	-1.158474
DSTNP2	destrin (actin depolymerizing factor) pseudogene 2	4.3020744	1.6416792	1.0236552
DTL	denticleless E3 ubiquitin protein ligase homolog (Drosophila)	1.4897574	-4.269376	-9.011655
DTX2	deltex 2, E3 ubiquitin ligase	1.9414345	-1.1716876	-1.653791
DTYMK	deoxythymidylate kinase (thymidylate kinase)	1.6432844	-1.4969254	-1.435697
DUS3L	dihydrouridine synthase 3-like (S. cerevisiae)	-1.0092071	-1.2163258	-1.6138469
DUSP1	dual specificity phosphatase 1	3.9726102	2.6248527	4.027043
DUSP14	dual specificity phosphatase 14	1.9726514	1.0396925	-1.1183789
DUSP23	dual specificity phosphatase 23	2.56622	2.6771579	2.0455205
DUSP28	dual specificity phosphatase 28	1.1705023	1.3753653	1.7701579
DUSP6	dual specificity phosphatase 6	-6.186949	-5.1783667	-3.762027
DUSP8	dual specificity phosphatase 8	1.3913864	1.4120123	1.649231
DUSP8	dual specificity phosphatase 8	-1.2742077	2.2931292	2.7563047
DUSP9	dual specificity phosphatase 9	-1.8204113	1.3851249	-1.3083127
DUT	deoxyuridine triphosphatase	-1.0018158	-1.6542197	-1.8145375
DUT	deoxyuridine triphosphatase	1.1127319	-1.5103405	-1.6341794
DXO	decapping exoribonuclease	-1.1226459	-1.1770114	-1.6715329
DYNC1H1	dynein, cytoplasmic 1, heavy chain 1	-2.6508791	-2.1453426	-3.0663111
DYNC1LI2	dynein, cytoplasmic 1, light intermediate chain 2	-1.6587571	-2.0102127	-1.6137005
DYNC1LI2	dynein, cytoplasmic 1, light intermediate chain 2	-1.8659067	-2.3763933	-1.9996709
DYNLL1	dynein, light chain, LC8-type 1	1.1824212	-1.0808449	-1.6998684
DYNLL1	dynein, light chain, LC8-type 1	1.0622154	-1.229296	-1.9592185
DYNLL2	dynein, light chain, LC8-type 2	1.5799854	1.7794503	2.557445
DYNLT3	dynein, light chain, Tctex-type 3	-1.3874735	-1.675565	-1.1464694
DYSF	dysferlin	-1.5739446	-3.1679623	-4.2382107
E2F1	E2F transcription factor 1	1.8763112	-4.946631	-6.0174265
E2F2	E2F transcription factor 2	1.9633752	-3.238591	-9.562704
E2F3	E2F transcription factor 3	1.1414876	-1.3678137	-1.851316
E2F6	E2F transcription factor 6	-1.2222358	-1.6884742	-1.5911882
E2F6	E2F transcription factor 6	-1.4166195	-2.0564427	-2.190163
E2F7	E2F transcription factor 7	-3.0751708	-25.865717	-46.122173
E4F1	E4F transcription factor 1	-1.1622136	-1.2891195	-1.5759673
EAPP	E2F-associated phosphoprotein	1.7913274	1.8813015	2.1812713
EBNA1BP2	EBNA1 binding protein 2	1.5712425	1.2241238	1.0117873
EBP	emopamil binding protein (sterol isomerase)	1.3859617	1.7436726	3.379566
EBPL	emopamil binding protein-like	2.279874	2.9035654	2.387071
ECHDC1	ethylmalonyl-CoA decarboxylase 1	1.7115728	2.140267	1.7854692
ECHDC2	enoyl CoA hydratase domain containing 2	-1.1220746	3.0499666	8.995053
ECHDC3	enoyl CoA hydratase domain containing 3	1.030601	2.3510635	2.970565
ECHS1	enoyl CoA hydratase, short chain, 1, mitochondrial	1.1443919	1.760744	3.1269412
ECI1	enoyl-CoA delta isomerase 1	1.7458928	2.7571023	3.238041
ECI2	enoyl-CoA delta isomerase 2	1.3109828	1.7473955	2.2415142
ECM1	extracellular matrix protein 1	1.1713288	1.0393547	-11.033589
EDA2R	ectodysplasin A2 receptor	1.1424477	-1.4862707	-3.3439536
EDIL3	EGF-like repeats and discoidin I-like domains 3	1.6843015	-2.1026034	-4.8215694
EDN1	endothelin 1	-1.1822062	-1.8430524	-8.232664
EEF1A2	eukaryotic translation elongation factor 1 alpha 2	-1.5670029	-1.236783	-6.266573
EEF1D	eukaryotic translation elongation factor 1 delta (guanine nucleotide exchange protein)	1.0069991	-1.4254625	-1.5670617
EEF1E1	eukaryotic translation elongation factor 1 epsilon 1	1.0906012	-1.38952	-1.569409
EEF2K	eukaryotic elongation factor 2 kinase	1.3384589	-1.0418519	-2.3160954
EEF2KMT	eukaryotic elongation factor 2 lysine methyltransferase	1.5486997	1.7897923	-1.0608159
EEF2KMT	eukaryotic elongation factor 2 lysine methyltransferase	1.291256	1.5336411	-1.213926
EFCAB11	EF-hand calcium binding domain 11	1.5528514	-1.1034007	-1.1770889
EFCAB14	EF-hand calcium binding domain 14	1.0316869	1.2294705	1.6266304
EFEMP1	EGF containing fibulin-like extracellular matrix protein 1	2.9426181	1.046447	-1.4102999
EFHD2	EF-hand domain family, member D2	1.0172187	-1.2913263	-2.001711
EFNA1	ephrin-A1	-1.3224479	1.9032758	2.6505022
EFNB1	ephrin-B1	-1.3711606	-1.8009391	-1.8451365
EFTUD1	elongation factor Tu GTP binding domain containing 1	-1.3589991	-1.5344275	-1.498334
EGFL7	EGF-like-domain, multiple 7	-1.2933906	-1.1296014	1.6002353
EGFR	epidermal growth factor receptor	-1.5579823	-1.48081	-1.2621789
EGLN1	egl-9 family hypoxia-inducible factor 1	1.0469741	1.6524467	1.7121592
EGLN3	egl-9 family hypoxia-inducible factor 3	2.603247	1.8695381	-1.7622224
EHBP1	EH domain binding protein 1	-2.0388725	-1.6621704	-1.7834557
EHBP1L1	EH domain binding protein 1-like 1	-2.5452638	-2.3211443	-7.5789
EHD1	EH-domain containing 1	-1.536427	-1.3064796	-1.1680175
EHD2	EH-domain containing 2	1.7438295	1.1319488	-1.1631663
EHHADH	enoyl-CoA, hydratase/3-hydroxyacyl CoA dehydrogenase	-1.611358	2.303408	6.512442
EHMT2	euchromatic histone-lysine N-methyltransferase 2	-1.9352658	-2.1044984	-2.770651
EID2	EP300 interacting inhibitor of differentiation 2	1.8833518	1.6402725	2.0645247
EIF1	eukaryotic translation initiation factor 1	1.3842939	1.7266681	2.3447587
EIF1AX	eukaryotic translation initiation factor 1A, X-linked	1.2177218	1.1998069	1.62309
EIF1AX	eukaryotic translation initiation factor 1A, X-linked	1.0834849	1.2811772	1.6209449
EIF1AX	eukaryotic translation initiation factor 1A, X-linked	1.188846	1.1820385	1.5730482
EIF1AY	eukaryotic translation initiation factor 1A, Y-linked	1.6721414	1.3744602	1.9101284
EIF2AK4	eukaryotic translation initiation factor 2 alpha kinase 4	-1.0946153	-1.7683561	-2.333531
EIF2S1	eukaryotic translation initiation factor 2, subunit 1 alpha, 35kDa	1.6150491	1.1333691	-1.1008431
EIF3B	eukaryotic translation initiation factor 3, subunit B	-1.1889038	-1.4419355	-1.6939714
EIF3B	eukaryotic translation initiation factor 3, subunit B	-1.2239377	-1.4311775	-1.659625
EIF3D	eukaryotic translation initiation factor 3, subunit D	1.2992685	1.1417813	-1.5737824
EIF3I	eukaryotic translation initiation factor 3, subunit I	1.0420611	-1.115379	-1.512778
EIF3J	eukaryotic translation initiation factor 3, subunit J	1.7329746	1.5902365	1.6920978
EIF3K	eukaryotic translation initiation factor 3, subunit K	1.6040075	1.8438066	1.0525566
EIF4E	eukaryotic translation initiation factor 4E	1.8930905	1.5124667	1.5056446
EIF4EBP1	eukaryotic translation initiation factor 4E binding protein 1	-1.4183431	-1.7639453	-1.902821
EIF4EBP2	eukaryotic translation initiation factor 4E binding protein 2	1.3357133	1.8632088	3.116719
EIF4EBP2	eukaryotic translation initiation factor 4E binding protein 2	1.8300458	2.3341203	2.633245
EIF4ENIF1	eukaryotic translation initiation factor 4E nuclear import factor 1	1.7439121	1.8230082	1.6910077
EIF4G3	eukaryotic translation initiation factor 4 gamma, 3	-2.0554347	-2.1855476	-2.2761927

ELAC1	elaC ribonuclease Z 1	1.5762258	1.7483631	2.476131
ELAVL1	ELAV like RNA binding protein 1	-1.5211585	-2.1870198	-1.7892576
ELF1	E74-like factor 1 (ets domain transcription factor)	-1.4931451	-1.4354205	-1.681633
ELF3	E74-like factor 3 (ets domain transcription factor, epithelial-specific)	-5.3905735	-2.8073735	-1.6643994
ELF4	E74-like factor 4 (ets domain transcription factor)	1.44957	-1.9529779	-6.3193145
ELFN2	extracellular leucine-rich repeat and fibronectin type III domain containing 2	-1.3403105	-2.268113	-4.6549673
ELL2	elongation factor, RNA polymerase II, 2	1.8645432	1.7688614	1.1710018
ELMOD2	ELMO/CED-12 domain containing 2	-1.1980613	-1.0090529	1.5474656
ELOVL5	ELOVL fatty acid elongase 5	1.8735784	-1.1557277	-1.9977512
ELOVL5	ELOVL fatty acid elongase 5	-1.095477	-1.5380279	-1.3180147
ELP3	elongator acetyltransferase complex subunit 3	1.4428234	1.6937696	2.2861044
ELP5	elongator acetyltransferase complex subunit 5	1.2021108	1.8060373	1.5409336
EMB	embigin	1.2044442	-1.804635	1.4287016
EMB	embigin	1.2331623	-1.2100195	1.7998389
EMC1	ER membrane protein complex subunit 1	-1.2359746	-2.15461	-2.020414
EMC2	ER membrane protein complex subunit 2	1.2768433	1.4721807	1.8884639
EMC8	ER membrane protein complex subunit 8	1.8531779	1.3961371	1.3743513
EMC9	ER membrane protein complex subunit 9	1.8416743	1.2018836	2.939573
EMILIN1	elastin microfibril interfacer 1	1.3794127	1.6308055	2.0551224
EMILIN3	elastin microfibril interfacer 3	3.3967986	2.238362	1.5005188
EML1	echinoderm microtubule associated protein like 1	-2.0607343	-3.4961326	-2.3197446
EMP1	epithelial membrane protein 1	1.725857	-1.0382243	-1.5442305
EMP2	epithelial membrane protein 2	2.147555	2.5337777	2.5238411
EMP3	epithelial membrane protein 3	-1.4321439	-5.034234	-17.258867
EMX1	empty spiracles homeobox 1	1.7952423	1.5984647	1.4910923
EN2	engrailed homeobox 2	2.0014393	2.0972185	2.4202712
ENAH	enabled homolog (Drosophila)	-1.2154928	-2.278605	-2.1246896
ENDOD1	endonuclease domain containing 1	1.2715096	-2.5443413	-1.6592263
ENDOG	endonuclease G	1.6031642	1.9484863	1.765381
ENDOV	endonuclease V	-1.0658451	1.2245289	1.7038809
ENHO	energy homeostasis associated	1.9746653	1.5261928	1.4697835
ENKD1	enkurin domain containing 1	-1.3153344	-1.0444195	-2.8245022
ENPP1	ectonucleotide pyrophosphatase/phosphodiesterase 1	-1.0124989	1.1718998	2.0130014
ENPP2	ectonucleotide pyrophosphatase/phosphodiesterase 2	3.3005466	1.5832461	1.4828899
ENPP4	ectonucleotide pyrophosphatase/phosphodiesterase 4 (putative)	-1.1707195	1.2386156	2.748885
ENTPD1	ectonucleoside triphosphate diphosphohydrolase 1	2.6506524	2.5509217	2.9280841
ENTPD4	ectonucleoside triphosphate diphosphohydrolase 4	-1.6128242	-2.38179	-1.9015056
ENTPD8	ectonucleoside triphosphate diphosphohydrolase 8	-3.7813802	8.039507	9.42284
ENY2	enhancer of yellow 2 homolog (Drosophila)	1.1145755	1.1063089	1.8619452
EPAS1	endothelial PAS domain protein 1	1.4057021	1.5623401	1.6205862
EPB41L1	erythrocyte membrane protein band 4.1-like 1	2.171694	1.5978963	-2.0320075
EPB41L3	erythrocyte membrane protein band 4.1-like 3	1.0104009	-1.9115518	-2.150413
EPB41L4A	erythrocyte membrane protein band 4.1 like 4A	1.1808327	1.4512643	2.1196108
EPB41L4A-AS1	EPB41L4A antisense RNA 1	1.4891965	1.5909557	1.6134223
EPB41L4B	erythrocyte membrane protein band 4.1 like 4B	1.0546463	2.122423	2.9931068
EPB41L5	erythrocyte membrane protein band 4.1 like 5	-2.9289112	1.8746744	4.0683527
EPDR1	ependymin related 1	1.7017732	-1.0253615	-1.0974543
EPHB2	EPH receptor B2	-2.2880392	-4.842529	-3.6831589
EPHB4	EPH receptor B4	-1.4724609	-2.033555	-3.0991368
EPHB6	EPH receptor B6	-1.5513271	-2.0328429	-2.7536082
EPHX1	epoxide hydrolase 1, microsomal (xenobiotic)	-2.310359	-2.0232804	2.001408
EPHX2	epoxide hydrolase 2, cytoplasmic	-1.0687088	4.662943	8.35788
EPHX3	epoxide hydrolase 3	1.5963343	1.6644195	1.9367701
EPRS	glutamyl-prolyl-tRNA synthetase	-1.4710068	-1.6598408	-1.830317
EPS15	epidermal growth factor receptor pathway substrate 15	1.3137062	2.3053079	2.3603327
EPS15	epidermal growth factor receptor pathway substrate 15	-3.651455	-2.4536352	-2.2530053
EPS8L3	EPS8-like 3	-3.8165624	-1.5971845	-2.9757788
EPT1	ethanolaminephosphotransferase 1 (CDP-ethanolamine-specific)	-1.83883	-1.5954912	-1.0918686
ERAP2	endoplasmic reticulum aminopeptidase 2	-1.2152086	-2.7576444	-3.5241416
ERBB3	erb-b2 receptor tyrosine kinase 3	-2.4756248	2.773349	3.1652467
ERCC2	excision repair cross-complementation group 2	1.6342609	1.5200851	1.8245564
ERCC5	excision repair cross-complementation group 5	-1.5090532	-1.2497903	1.0652466
ERCC8	excision repair cross-complementation group 8	-1.4701626	-1.57449	-2.1132758
ERGIC1	endoplasmic reticulum-golgi intermediate compartment (ERGIC) 1	2.2362635	1.5493177	1.5645181
ERGIC2	ERGIC and golgi 2	-1.395941	-2.2943158	-2.3855891
ERH	enhancer of rudimentary homolog (Drosophila)	1.7626084	1.3605106	1.3182292
ERI1	exoribonuclease 1	-1.2092853	-1.7184383	-1.9191347
ERI2	ERI1 exoribonuclease family member 2	1.2821051	1.9223702	1.9495158
ERICH2	glutamate-rich 2	-1.7776383	-1.0431792	-2.0933359
ERICH4	glutamate-rich 4	1.7366505	1.7873646	1.9591801
ERICH5	glutamate-rich 5	1.3483878	2.6683724	2.6780043
ERMARD	ER membrane-associated RNA degradation	1.2388568	1.6922976	1.8369517
ERO1L	ERO1-like (S. cerevisiae)	1.0375128	-2.0568984	-1.3008192
ERP27	endoplasmic reticulum protein 27	1.0706444	43.61843	98.16744
ERRFI1	ERBB receptor feedback inhibitor 1	-1.3520969	1.7252046	3.9177046
ERVMER34-1	endogenous retrovirus group MER34, member 1	3.2379875	1.4239386	-2.324881
ERVMER34-1	endogenous retrovirus group MER34, member 1	3.5220618	1.5579109	-2.3505416
ERVMER34-1	endogenous retrovirus group MER34, member 1	3.1899798	1.4817995	-2.3386703
ESF1	ESF1, nucleolar pre-rRNA processing protein, homolog (S. cerevisiae)	-1.3941023	-1.5106583	-1.2804792
ESPN	espin	-1.8108627	2.7827659	6.623316
ESRP2	epithelial splicing regulatory protein 2	1.2463644	2.8704634	5.0555406
ESRRA	estrogen-related receptor alpha	1.6065898	2.3485804	1.5493207
ETFA	electron-transfer-flavoprotein, alpha polypeptide	-1.8287737	-1.3504379	1.1837316
ETFB	electron-transfer-flavoprotein, beta polypeptide	-1.0344123	1.595511	2.0303707
ETFDH	electron-transferring-flavoprotein dehydrogenase	-1.303494	1.3590657	2.4107018
ETHE1	ethylmalonic encephalopathy 1	1.1724309	1.3989322	1.8273776
ETNK2	ethanolamine kinase 2	2.8615828	6.305976	16.393341
ETNK2	ethanolamine kinase 2	1.5977439	4.4904556	11.267998
ETNPPL	ethanolamine-phosphate phospho-lyase	-1.3926755	5.3815317	24.332636
ETV1	ets variant 1	1.2095715	2.6001961	3.4395616
ETV1	ets variant 1	-2.2893863	1.7391248	-1.3546902
EVA1C	eva-1 homolog C (C. elegans)	20.649553	5.5782676	2.8797114
EVL	Enah/Vasp-like	1.0245141	-1.6048751	-1.2271863
EVPL	envoplakin	1.1411246	1.48907	1.9146163
EVX1	even-skipped homeobox 1	2.6991022	2.8966248	3.3295953
EWSAT1	Ewing sarcoma associated transcript 1	2.6744998	2.8945389	2.9939091
EWSR1	EWS RNA-binding protein 1	-1.145385	-1.4204159	-2.0050435
EXO5	exonuclease 5	-1.0010834	-1.6367186	-1.2947441
EXOC3L1	exocyst complex component 3-like 1	3.033839	3.6290274	4.6143456
EXOC6	exocyst complex component 6	-1.2863394	1.0515746	1.6983719
EXOC7	exocyst complex component 7	-2.5412667	-2.1032648	-1.962768
EXOSC2	exosome component 2	1.6962925	1.1604332	-1.0608524
EXOSC3	exosome component 3	1.6153953	1.2036679	1.2127854
EXOSC8	exosome component 8	1.1142286	-1.5045989	-1.2211567
EXOSC9	exosome component 9	1.107224	-1.6886244	-1.3220245
EXT1	exostosin glycosyltransferase 1	-1.1695031	-1.7136741	-2.2205102
EXT2	exostosin glycosyltransferase 2	1.1676801	-1.9302399	-1.2071228
EYA1	EYA transcriptional coactivator and phosphatase 1	1.8469607	2.8705244	9.48956
EZH1	enhancer of zeste 1 polycomb repressive complex 2 subunit	-1.3086737	1.2165786	1.6673168

EZH2	enhancer of zeste 2 polycomb repressive complex 2 subunit	1.1226115	-3.6819205	-3.5449443
F10	coagulation factor X	-1.2369409	3.2060735	6.1649137
F11	coagulation factor XI	-4.5879717	3.3833287	5.641771
F11R	F11 receptor	-1.5232536	-1.0496379	1.5861193
F11R	F11 receptor	-1.2232614	1.2256147	2.282284
F12	coagulation factor XII (Hageman factor)	-2.270907	24.882511	124.06196
F13B	coagulation factor XIII, B polypeptide	-4.011337	8.347182	28.007645
F2	coagulation factor II (thrombin)	-3.1197224	3.9292586	6.956013
F2R	coagulation factor II (thrombin) receptor	1.1124781	-3.1570947	-5.675931
F2RL1	coagulation factor II (thrombin) receptor-like 1	-3.3323941	-2.0827727	-1.2989262
F3	coagulation factor III (thromboplastin, tissue factor)	1.5047821	-3.3327534	-4.153379
F5	coagulation factor V (proaccelerin, labile factor)	-4.1488843	3.8708053	2.3177483
F8A1	coagulation factor VIII-associated 1	1.3472855	1.1260719	1.7451785
F9	coagulation factor IX	-7.3824906	17.355242	66.44546
FABP1	fatty acid binding protein 1, liver	-14.943462	8.494967	37.851086
FADS1	fatty acid desaturase 1	1.8555175	1.3585085	2.502454
FADS3	fatty acid desaturase 3	1.9082242	1.0967319	-1.1136807
FAH	fumarylacetoacetate hydrolase (fumarylacetoacetase)	-1.1616154	1.1618588	1.7361126
FAHD1	fumarylacetoacetate hydrolase domain containing 1	1.4282376	1.6634843	2.30588
FAHD2A	fumarylacetoacetate hydrolase domain containing 2A	1.1243896	1.4628886	1.6984735
FAHD2A	fumarylacetoacetate hydrolase domain containing 2A	1.053046	1.3073581	1.5179486
FAIM	Fas apoptotic inhibitory molecule	1.8553429	1.5362446	-1.2751228
FAM107A	family with sequence similarity 107, member A	1.793936	2.932376	4.405788
FAM107B	family with sequence similarity 107, member B	-1.3539091	1.1426588	1.671046
FAM110A	family with sequence similarity 110, member A	-1.1531926	-1.8739944	-4.2629333
FAM110C	family with sequence similarity 110, member C	-1.1992499	2.2208931	3.1790507
FAM111B	family with sequence similarity 111, member B	2.7308412	-2.6412997	-2.5293303
FAM117A	family with sequence similarity 117, member A	1.4718877	1.8409745	2.1929784
FAM118A	family with sequence similarity 118, member A	2.384337	2.610097	2.6618865
FAM120A	family with sequence similarity 120A	-1.5045465	-1.4300724	-1.4877018
FAM120AOS	family with sequence similarity 120A opposite strand	1.4166179	2.006505	3.1208494
FAM120C	family with sequence similarity 120C	1.5566684	2.0552604	2.1424994
FAM122A	family with sequence similarity 122A	1.3794925	1.159729	2.4079165
FAM131C	family with sequence similarity 131, member C	1.4845487	2.4548082	3.096433
FAM134B	family with sequence similarity 134, member B	-1.0556461	2.6522102	3.6518223
FAM134C	family with sequence similarity 134, member C	-1.1388397	-1.0206631	1.8890128
FAM136A	family with sequence similarity 136, member A	1.5658658	1.4945827	-1.1622154
FAM13A	family with sequence similarity 13, member A	1.8465241	4.053176	4.132389
FAM13B	family with sequence similarity 13, member B	1.928763	1.3506345	1.4733485
FAM149B1	family with sequence similarity 149, member B1	1.2182587	1.7335951	2.2662003
FAM151A	family with sequence similarity 151, member A	-1.1544279	7.2731104	19.871485
FAM160A2	family with sequence similarity 160, member A2	-1.5789568	-1.0419718	1.2615796
FAM160B2	family with sequence similarity 160, member B2	-1.2540318	-1.4565908	-1.7990925
FAM162A	family with sequence similarity 162, member A	1.6738944	2.1817198	2.3851795
FAM171A1	family with sequence similarity 171, member A1	-1.2354043	-1.397022	-2.1748328
FAM174B	family with sequence similarity 174, member B	-1.7192	1.1842842	1.761205
FAM178B	family with sequence similarity 178, member B	1.6784129	1.3745368	1.5523945
FAM178B	family with sequence similarity 178, member B	1.9579519	2.1507375	2.6279795
FAM181A	family with sequence similarity 181, member A	1.4805703	1.6934326	2.0452905
FAM189B	family with sequence similarity 189, member B	1.0748222	-1.3038834	-1.7520994
FAM192A	family with sequence similarity 192, member A	1.4414102	1.650063	1.5772084
FAM195A	family with sequence similarity 195, member A	1.2174753	1.8575764	1.7699249
FAM195A	family with sequence similarity 195, member A	1.0570503	1.6681253	1.5461154
FAM198B	family with sequence similarity 198, member B	2.7700627	-1.0918831	-3.7473276
FAM199X	family with sequence similarity 199, X-linked	1.2859797	1.6125246	1.8417186
FAM19A2	family with sequence similarity 19 (chemokine (C-C motif)-like), member A2	1.9694003	1.0573838	-1.739544
FAM20A	family with sequence similarity 20, member A	-2.0077832	2.5721529	2.1445012
FAM20C	family with sequence similarity 20, member C	1.0074682	1.3000274	3.3252397
FAM213A	family with sequence similarity 213, member A	-1.2844684	-1.0015594	1.9880965
FAM213A	family with sequence similarity 213, member A	-1.4551038	-1.1246226	1.7770233
FAM214A	family with sequence similarity 214, member A	1.0712745	1.0590494	1.7400275
FAM214B	family with sequence similarity 214, member B	-1.174279	-1.5892277	-2.0941737
FAM216A	family with sequence similarity 216, member A	1.9445792	1.0097165	-1.168619
FAM219A	family with sequence similarity 219, member A	2.4293344	2.2899077	2.5826805
FAM21C	family with sequence similarity 21, member C	2.5044901	3.3995433	4.097241
FAM21C	family with sequence similarity 21, member C	-2.5177994	-2.7521253	-3.043802
FAM21C	family with sequence similarity 21, member C	-1.1000777	-1.2438109	-1.6086395
FAM222B	family with sequence similarity 222, member B	2.0517604	1.3572748	-1.0123564
FAM228B	family with sequence similarity 228, member B	-1.2173463	1.35202	1.6619883
FAM231A	family with sequence similarity 231, member A	2.5931957	2.1000242	1.5974457
FAM24B	family with sequence similarity 24, member B	-1.1482866	-2.56399	-3.059188
FAM27C	family with sequence similarity 27, member C	1.3951576	1.4967074	1.829522
FAM45A	family with sequence similarity 45, member A	-1.299234	1.1892974	1.8003992
FAM45A	family with sequence similarity 45, member A	1.3096807	1.8205854	2.6783285
FAM46A	family with sequence similarity 46, member A	-2.9259226	-2.2227983	-4.4070344
FAM46A	family with sequence similarity 46, member A	-1.6435623	-1.2954141	-2.2111294
FAM46C	family with sequence similarity 46, member C	-1.5717841	-1.2474016	1.204332
FAM47E	family with sequence similarity 47, member E	-1.4936612	1.8331412	1.875718
FAM49B	family with sequence similarity 49, member B	1.9502589	1.3303471	-1.1473567
FAM53C	family with sequence similarity 53, member C	-1.8811979	-2.301917	-1.8154242
FAM57A	family with sequence similarity 57, member A	-1.0936877	-2.2364852	-4.1829867
FAM58A	family with sequence similarity 58, member A	1.7494782	2.0727437	1.9151617
FAM60A	family with sequence similarity 60, member A	-1.1558675	-1.7368311	-3.6224706
FAM63A	family with sequence similarity 63, member A	1.201086	2.4095929	2.7978103
FAM66D	family with sequence similarity 66, member D	-1.3139077	-1.7212018	-2.1706734
FAM69B	family with sequence similarity 69, member B	1.727866	3.2996705	2.1392992
FAM73A	family with sequence similarity 73, member A	-1.1120489	-1.0178369	1.6574177
FAM78B	family with sequence similarity 78, member B	1.4419359	2.1480923	2.448694
FAM83D	family with sequence similarity 83, member D	3.3652487	1.0312741	1.8787177
FAM84A	family with sequence similarity 84, member A	5.5458913	2.8367326	4.1542616
FAM86B2	family with sequence similarity 86, member B2	1.160915	1.6279039	-1.4226325
FAM86B2	family with sequence similarity 86, member B2	1.3212593	1.6139973	-1.4022652
FAM86B2	family with sequence similarity 86, member B2	1.1913837	1.5466104	-1.5173426
FAM86B3P	family with sequence similarity 86, member A pseudogene	1.0359185	1.4696206	-1.5137694
FAM89B	family with sequence similarity 89, member B	-1.2074121	1.0950167	1.525688
FAM96A	family with sequence similarity 96, member A	1.4753485	2.1387863	1.802179
FAM99A	family with sequence similarity 99, member A (non-protein coding)	2.0805297	2.3374138	1.9504359
FANCC	Fanconi anemia, complementation group C	-1.1596684	1.0599018	-2.1178267
FANCE	Fanconi anemia, complementation group E	1.1338452	-1.2166921	-1.5305355
FANCF	Fanconi anemia, complementation group F	1.3685861	1.8380653	1.4806943
FANCF	Fanconi anemia, complementation group F	1.6756011	1.5685165	1.2940739
FANCG	Fanconi anemia, complementation group G	1.0684962	-2.9550016	-3.055012
FANCI	Fanconi anemia, complementation group I	1.0421566	-4.86934	-5.92984
FANCM	Fanconi anemia, complementation group M	1.514108	-1.5045741	-2.5586808
FARP1	FERM, RhoGEF (ARHGEF) and pleckstrin domain protein 1 (chondrocyte-derived)	-1.9014881	-1.6409774	-2.6806843
FARSB	phenylalanyl-tRNA synthetase, beta subunit	-1.162394	-1.668213	-2.004612
FAS	Fas cell surface death receptor	-1.3942491	-2.411025	-2.8005989
FAS	Fas cell surface death receptor	-1.6374645	-2.5614238	-3.111506
FAS	Fas cell surface death receptor	-1.2381971	-2.182866	-2.607118

FASN	fatty acid synthase	2.2912982	2.7113538	4.099869
FASTKD1	FAST kinase domains 1	1.4235511	1.0961981	-1.5021734
FAT1	FAT atypical cadherin 1	-5.424151	-4.267333	-5.3791666
FAXDC2	fatty acid hydroxylase domain containing 2	1.0980583	4.5757003	7.6239614
FBLIM1	filamin binding LIM protein 1	-1.0541515	-3.7152038	-21.5583
FBLL1	fibrillarin-like 1	4.0260096	2.4229753	2.14702
FBLN1	fibulin 1	2.3313975	1.2880849	-1.04169
FBLN1	fibulin 1	1.8584287	1.0075191	-2.2537003
FBRSL1	fibrosin-like 1	2.172497	2.8952954	3.4037633
FBRSL1	fibrosin-like 1	1.2045182	1.6383342	1.8671373
FBXL16	F-box and leucine-rich repeat protein 16	1.9973973	1.7781881	-1.0885739
FBXL17	F-box and leucine-rich repeat protein 17	1.2157	1.5522231	1.8901145
FBXL7	F-box and leucine-rich repeat protein 7	1.2193187	-1.5259036	-1.2020339
FBXO10	F-box protein 10	1.5846735	1.5843638	2.5881054
FBXO18	F-box protein, helicase, 18	-1.4239229	-1.2557545	-1.8663138
FBXO24	F-box protein 24	1.1513098	2.2682533	4.8128858
FBXO31	F-box protein 31	-1.084301	1.2519327	1.5710448
FBXO32	F-box protein 32	2.2877026	-1.0812545	-1.0531334
FBXO33	F-box protein 33	-1.4537219	-1.915703	-1.0912174
FBXO41	F-box protein 41	1.9170713	1.3865325	-2.5693476
FBXO5	F-box protein 5	1.899021	-1.9794765	-2.1371188
FBXO6	F-box protein 6	1.152375	2.0876393	2.7404144
FBXW7	F-box and WD repeat domain containing 7, E3 ubiquitin protein ligase	1.109832	-1.6410148	-1.9864025
FCER1G	Fc fragment of IgE, high affinity I, receptor for; gamma polypeptide	3.0988717	1.8329892	2.0638506
FCGRT	Fc fragment of IgG, receptor, transporter, alpha	-1.417888	2.6546185	3.8495352
FCHO1	FCH domain only 1	-1.0906422	-1.1386647	-1.6251608
FCHSD2	FCH and double SH3 domains 2	1.0334556	-1.3466165	-2.1256633
FDFT1	farnesyl-diphosphate farnesyltransferase 1	1.5455946	-1.0173725	1.8824508
FDPS	farnesyl diphosphate synthase	1.7023289	1.7025577	2.530008
FDPSP2	farnesyl diphosphate synthase pseudogene 2	1.4826707	1.5814474	2.294799
FDX1	ferredoxin 1	1.4223826	2.3039503	2.3773606
FDX1L	ferredoxin 1-like	1.96198	2.0449944	2.3250697
FDXR	ferredoxin reductase	1.6252455	1.31534	-2.0096948
FEM1C	fem-1 homolog c (C. elegans)	1.1359738	1.3063668	1.8901087
FEN1	flap structure-specific endonuclease 1	1.3533708	-2.4211035	-3.7428014
FETUB	fetuin B	-1.1697084	2.831794	4.681082
FGA	fibrinogen alpha chain	-1.6640283	3.7863145	8.451184
FGA	fibrinogen alpha chain	-1.3631632	6.97384	7.397507
FGA	fibrinogen alpha chain	-1.608125	3.9987013	7.865227
FGB	fibrinogen beta chain	-1.4782803	5.1027493	8.364036
FGF14-AS2	FGF14 antisense RNA 2	1.7074078	2.915534	2.6927114
FGFR1	fibroblast growth factor receptor 1	-1.0392417	-2.5904708	-2.6156085
FGFR1OP	FGFR1 oncogene partner	-1.1357279	-1.2001091	1.5861756
FGFR2	fibroblast growth factor receptor 2	1.4321257	1.8614044	1.304451
FGFR3	fibroblast growth factor receptor 3	-2.453754	1.8283587	1.5080509
FGFR4	fibroblast growth factor receptor 4	-1.7456865	-1.211158	1.0633787
FGG	fibrinogen gamma chain	-1.152536	4.819622	7.954692
FGGY	FGGY carbohydrate kinase domain containing	1.1847956	2.0274029	1.887483
FGL1	fibrinogen-like 1	-3.9334455	5.8475585	12.013998
FH	fumarate hydratase	1.2154279	1.5851847	1.5789996
FHL1	four and a half LIM domains 1	3.3699803	3.5895321	4.0232472
FHL2	four and a half LIM domains 2	1.6520842	-2.2799957	-7.3573875
FHL2	four and a half LIM domains 2	-1.1417847	-4.375873	-16.735462
FIBCD1	fibrinogen C domain containing 1	1.313389	1.27506	3.5548434
FIGNL2	fidgetin-like 2	2.1486814	2.0648453	2.6478853
FJX1	four jointed box 1 (Drosophila)	1.5175542	-1.1447829	-1.9319843
FKBP11	FK506 binding protein 11, 19 kDa	-1.5912303	-1.3380902	-3.4124067
FKBP11	FK506 binding protein 11, 19 kDa	-1.5193144	-1.3156668	-3.2926083
FKBP14	FK506 binding protein 14, 22 kDa	-1.4331521	-2.515347	-2.8909855
FKBP1A	FK506 binding protein 1A, 12kDa	-1.0413245	-1.2610689	-1.739717
FKBP1A	FK506 binding protein 1A, 12kDa	-1.2807366	-1.4951223	-1.916516
FKBP1A	FK506 binding protein 1A, 12kDa	-1.3000423	-1.6622405	-2.287085
FKBP3	FK506 binding protein 3, 25kDa	1.7143316	1.2441113	1.6101214
FKBP5	FK506 binding protein 5	4.78469	5.878769	6.798266
FKRP	fukutin related protein	-1.8541064	-2.1892703	-1.8001964
FLCN	folliculin	-1.4612685	-1.9671441	-2.0343177
FLJ11292	uncharacterized protein FLJ11292	-3.643011	-2.8571498	-2.7181985
FLJ13773	uncharacterized LOC246318	-2.0802684	-1.8438561	-1.6468747
FLJ31713	uncharacterized protein FLJ31713	1.3104885	6.4513097	12.082069
FLJ32255	uncharacterized LOC643977	2.0898824	1.7630513	1.7379797
FLJ37453	uncharacterized LOC729614	1.4146794	1.5959275	1.7242609
FLJ40039	uncharacterized LOC647662	2.6913307	2.523278	3.22363
FLJ43681	ribosomal protein L23a pseudogene	1.839959	1.5882483	1.3533345
FLJ44477	FLJ44477 protein	1.5296417	1.5258399	1.6703545
FLJ45743	uncharacterized LOC642484	-2.3231928	-2.1733768	-1.6548073
FLJ46906	uncharacterized LOC441172	-1.9006642	-1.4453825	1.093295
FLNA	filamin A, alpha	-1.397216	-5.865735	-27.354404
FLNB	filamin B, beta	-3.6768062	-2.623076	-3.7928872
FLRT3	fibronectin leucine rich transmembrane protein 3	2.2419772	2.5573318	-1.0010959
FLVCR1	feline leukemia virus subgroup C cellular receptor 1	1.2622291	-1.3919288	-1.5122586
FMNL2	formin-like 2	-1.2327719	-1.7981944	-1.9735593
FMO3	flavin containing monooxygenase 3	5.087503	6.4413056	11.553785
FMO4	flavin containing monooxygenase 4	-1.2425804	2.422415	2.3899488
FMO5	flavin containing monooxygenase 5	-1.2508174	8.254616	23.787899
FMO5	flavin containing monooxygenase 5	-1.1349932	9.33892	18.713926
FN1	fibronectin 1	-4.0522904	-2.3903277	-5.024895
FN3KRP	fructosamine 3 kinase related protein	-1.1841025	-1.5533872	-1.0331447
FNBP1	formin binding protein 1	1.100641	1.052272	2.0516279
FNDC3A	fibronectin type III domain containing 3A	-2.0428314	-1.1989199	-1.0978657
FNDC4	fibronectin type III domain containing 4	-1.9798177	1.3721144	1.2167566
FOLR1	folate receptor 1 (adult)	-1.1250538	4.629687	1.7249736
FOLR3	folate receptor 3 (gamma)	-1.1129018	4.3934608	1.4617975
FOSL2	FOS-like antigen 2	1.1338172	-2.1695662	-3.488909
FOXK2	forkhead box K2	1.0339605	-1.3136466	-1.6046835
FOXL1	forkhead box L1	1.1602488	-1.5449181	-4.2570286
FOXN2	forkhead box N2	1.1071783	1.4096671	1.585129
FOXN3-AS1	FOXN3 antisense RNA 1	1.6118575	1.5176581	1.2679338
FOXO3	forkhead box O3	1.9433405	2.7294953	2.8206744
FOXO3	forkhead box O3	1.9472957	2.8125234	2.7912266
FOXQ1	forkhead box Q1	-2.2336674	1.0034307	1.9093297
FOXRED2	FAD-dependent oxidoreductase domain containing 2	1.4022642	-1.771866	1.0918183
FPGS	folylpolyglutamate synthase	-1.2352239	-1.432921	-1.8783233
FPGT	fucose-1-phosphate guanylyltransferase	1.1806221	1.3992132	2.1558254
FPGT	fucose-1-phosphate guanylyltransferase	1.7721866	2.141571	2.9035704
FPR1	formyl peptide receptor 1	3.8972695	1.9294021	-1.4241542
FRAT1	frequently rearranged in advanced T-cell lymphomas 1	1.2551992	3.6416311	5.5591063
FRAT2	frequently rearranged in advanced T-cell lymphomas 2	1.4859447	2.4084992	2.289421
FRK	fyn-related Src family tyrosine kinase	1.015006	1.8383371	2.5924537
FRMD3	FERM domain containing 3	1.5950876	1.0066729	1.1559366

FRMD3	FERM domain containing 3	1.5569142	-1.1771412	1.1271416
FRMD4B	FERM domain containing 4B	-1.4659971	-2.075806	-1.7126685
FRMD6	FERM domain containing 6	1.0510879	-2.8234959	-4.7491803
FRMPD3	FERM and PDZ domain containing 3	2.015957	2.2112002	2.7712595
FSD1	fibronectin type III and SPRY domain containing 1	2.5057964	2.8055315	2.9305599
FST	follistatin	1.2179681	-2.1370912	-6.4051785
FSTL1	follistatin-like 1	3.281426	1.5372275	1.3725301
FSTL3	follistatin-like 3 (secreted glycoprotein)	1.028303	-2.6682723	-14.359822
FTL	ferritin, light polypeptide	1.0312666	1.0679417	2.4755795
FTL	ferritin, light polypeptide	-1.5235589	-1.4212303	1.7627434
FTL	ferritin, light polypeptide	-1.3566458	-1.2447711	2.0826297
FTSJ1	FtsJ RNA methyltransferase homolog 1 (E. coli)	1.2052755	-1.459298	-2.1210752
FUBP1	far upstream element (FUSE) binding protein 1	-1.0111837	-1.3713073	-1.7831435
FUBP1	far upstream element (FUSE) binding protein 1	1.0066202	-1.1945546	-1.6030704
FUK	fucokinase	-1.5682948	1.0448	1.0571332
FUNDC2	FUN14 domain containing 2	-1.032266	-1.131874	-1.7308421
FUOM	fucose mutarotase	1.151047	1.4557046	1.6470796
FUS	FUS RNA binding protein	1.1274713	-1.6183964	-1.5510793
FUT8	fucosyltransferase 8 (alpha (1,6) fucosyltransferase)	-1.2737235	-2.7112076	-1.8670563
FXN	frataxin	1.7739505	1.6865219	1.5239612
FXYD3	FXYD domain containing ion transport regulator 3	2.1050029	1.7195733	-6.6885676
FXYD5	FXYD domain containing ion transport regulator 5	-1.7269121	-5.722808	-21.39616
FYN	FYN proto-oncogene, Src family tyrosine kinase	-3.4167967	13.366616	10.018923
FZD2	frizzled class receptor 2	1.7968918	-1.3697582	-3.3516266
FZD4	frizzled class receptor 4	1.1673863	4.32637	6.658726
FZD5	frizzled class receptor 5	1.3071746	1.5969576	3.36957
FZD6	frizzled class receptor 6	1.4105086	-1.4091493	-1.8821197
FZD8	frizzled class receptor 8	3.439525	5.9583564	3.8563712
G0S2	G0/G1 switch 2	-2.0512903	-1.3015441	2.039585
G3BP1	GTPase activating protein (SH3 domain) binding protein 1	1.5047926	-1.0297962	1.2893649
G6PC	glucose-6-phosphatase, catalytic subunit	-5.4065995	24.814262	432.7317
GABARAPL1	GABA(A) receptor-associated protein like 1	-1.117048	1.2974811	2.387816
GABARAPL2	GABA(A) receptor-associated protein-like 2	1.5526115	1.6290323	2.078492
GADD45A	growth arrest and DNA-damage-inducible, alpha	-1.5936701	-3.4675643	-2.0208302
GADD45B	growth arrest and DNA-damage-inducible, beta	4.065823	2.5603142	2.6230407
GADD45G	growth arrest and DNA-damage-inducible, gamma	1.4993747	2.668957	3.4369514
GAGE7	G antigen 7	1.0484363	-1.16256	-1.5220147
GAL3ST1	galactose-3-O-sulfotransferase 1	-1.2045214	3.0928385	2.436269
GALK1	galactokinase 1	1.1989584	1.6815934	1.6728673
GALK2	galactokinase 2	-1.7401329	-1.080329	-1.102974
GALM	galactose mutarotase (aldose 1-epimerase)	-1.3158232	1.7090282	2.6699767
GALM	galactose mutarotase (aldose 1-epimerase)	-1.4783409	1.3726679	2.1926568
GALNT1	polypeptide N-acetylgalactosaminyltransferase 1	2.2355347	2.5244458	2.9685993
GALNT1	polypeptide N-acetylgalactosaminyltransferase 1	1.5458989	-1.1796147	-1.5319428
GALNT10	polypeptide N-acetylgalactosaminyltransferase 10	-1.3993783	-2.524575	-2.4347138
GALNT18	polypeptide N-acetylgalactosaminyltransferase 18	1.3598846	-1.1220453	-2.1092622
GALNT7	polypeptide N-acetylgalactosaminyltransferase 7	1.0063398	-2.3217883	-3.3729322
GALT	galactose-1-phosphate uridylyltransferase	1.1707324	2.3970156	2.6897442
GAMT	guanidinoacetate N-methyltransferase	-1.2744639	1.7712951	1.8434739
GAPDH	glyceraldehyde-3-phosphate dehydrogenase	1.9198388	1.4507428	1.3788573
GAR1	GAR1 ribonucleoprotein	1.2799065	-1.1037045	-1.9326175
GAREM	GRB2 associated, regulator of MAPK1	-1.2741152	-1.6520374	-1.7505991
GARS	glycyl-tRNA synthetase	-1.6748697	-2.1671124	-2.1079733
GART	phosphoribosylglycinamide formyltransferase, phosphoribosylglycinamide synthetase, phosph	-1.0814734	-1.4045255	-2.592846
GART	phosphoribosylglycinamide formyltransferase, phosphoribosylglycinamide synthetase, phosph	-1.0641848	-1.204264	-2.1332283
GAS2	growth arrest-specific 2	1.1848778	3.6794007	2.1612766
GAS2L1P2	growth arrest-specific 2 like 1 pseudogene 2	1.8373618	1.4921764	1.2444268
GAS5	growth arrest-specific 5 (non-protein coding)	-1.6253347	-1.4279809	-1.9880948
GATA4	GATA binding protein 4	1.0750822	1.8025736	2.1047828
GATA6	GATA binding protein 6	-2.12673	-1.234754	1.1402345
GATAD2A	GATA zinc finger domain containing 2A	-1.0621091	-1.517954	-1.7154858
GATAD2A	GATA zinc finger domain containing 2A	1.1386873	-1.1213	-2.2335818
GATM	glycine amidinotransferase (L-arginine:glycine amidinotransferase)	-1.5533358	11.775967	21.275068
GATSL2	GATS protein-like 2	1.3018163	1.8062991	2.1560943
GATSL3	GATS protein-like 3	1.8686801	2.8853354	3.9193575
GATSL3	GATS protein-like 3	1.6203414	2.4239116	3.2862628
GBA	glucosidase, beta, acid	-1.4633461	-2.1555023	-2.836648
GBA3	glucosidase, beta, acid 3 (gene/pseudogene)	-3.0436294	34.237705	110.557816
GBAP1	glucosidase, beta, acid pseudogene 1	-1.4092109	-1.770632	-2.5018985
GBAS	glioblastoma amplified sequence	2.8186185	1.8646721	1.45466
GBE1	glucan (1,4-alpha-), branching enzyme 1	1.6927774	1.7232883	1.833136
GBGT1	globoside alpha-1,3-N-acetylgalactosaminyltransferase 1	2.0681367	1.7240615	2.011637
GBP2	guanylate binding protein 2, interferon-inducible	-2.470244	-1.9701916	-1.9234723
GBP6	guanylate binding protein family, member 6	2.1812062	2.2949235	2.2911003
GC	group-specific component (vitamin D binding protein)	-5.338482	4.381862	4.997343
GCA	grancalcin, EF-hand calcium binding protein	-1.514691	-1.5399835	-1.0734277
GCC2	GRIP and coiled-coil domain containing 2	-2.047519	-1.9761983	-2.010494
GCDH	glutaryl-CoA dehydrogenase	-1.362328	1.2548082	4.8486166
GCGR	glucagon receptor	-4.125746	4.4787183	1.3410475
GCH1	GTP cyclohydrolase 1	-1.0740921	1.4943385	1.7470272
GCHFR	GTP cyclohydrolase I feedback regulator	-1.8269218	2.5384364	4.4815598
GCKR	glucokinase (hexokinase 4) regulator	-12.698932	1.1412033	2.8156202
GCLC	glutamate-cysteine ligase, catalytic subunit	1.7604743	2.1881828	4.7584324
GCN1L1	GCN1 general control of amino-acid synthesis 1-like 1 (yeast)	-1.2742127	-1.4681377	-1.6398491
GCSH	glycine cleavage system protein H (aminomethyl carrier)	1.6902516	1.7948459	2.02036
GDA	guanine deaminase	-1.8175924	1.45939	1.2087588
GDF15	growth differentiation factor 15	-1.7042793	-6.224309	-5.7123404
GDI1	GDP dissociation inhibitor 1	-1.576976	-1.9387633	-1.5156149
GEMIN4	gem (nuclear organelle) associated protein 4	1.2330546	-1.5102153	-1.78727
GEMIN6	gem (nuclear organelle) associated protein 6	1.8759568	1.6227913	1.4392165
GFER	growth factor, augmenter of liver regeneration	1.7023796	1.5974182	1.8602232
GFM2	G elongation factor, mitochondrial 2	1.5052615	1.4433583	1.6464318
GFOD1	glucose-fructose oxidoreductase domain containing 1	1.9126048	1.5442052	1.6364863
GFOD2	glucose-fructose oxidoreductase domain containing 2	1.3146982	2.2036161	2.7750542
GFRA1	GDNF family receptor alpha 1	1.7472293	1.9424088	1.9460617
GFRA1	GDNF family receptor alpha 1	3.3571181	2.8866422	2.2710707
GGA1	golgi-associated, gamma adaptin ear containing, ARF binding protein 1	-1.7698289	-1.7663679	-1.767987
GGCT	gamma-glutamylcyclotransferase	1.8746537	1.5103049	1.4213794
GGCX	gamma-glutamyl carboxylase	-1.6737319	1.9460216	-1.0346266
GGN	gametogenetin	3.016986	2.7087214	2.0804777
GGT1	gamma-glutamyltransferase 1	1.9901215	2.1987898	2.5764644
GGT5	gamma-glutamyltransferase 5	2.6365077	2.978781	1.0870714
GHITM	growth hormone inducible transmembrane protein	1.2133489	1.4700919	1.6200953
GHR	growth hormone receptor	2.163415	4.4623623	7.0538535
GID4	GID complex subunit 4	1.4073368	1.3998572	1.5860264
GID8	GID complex subunit 8	1.2580135	1.3727559	1.8121923
GIN1	gypsy retrotransposon integrase 1	1.5317483	2.161179	2.1941316
GINS1	GINS complex subunit 1 (Psf1 homolog)	2.3055508	-2.5249817	-3.0084453
GINS2	GINS complex subunit 2 (Psf2 homolog)	1.4780439	-3.710947	-4.96312

GINS3	GINS complex subunit 3 (Psf3 homolog)	-1.0538458	-1.7326382	-2.200191
GINS4	GINS complex subunit 4 (Sld5 homolog)	1.1709645	-3.6172295	-4.2944603
GIPC1	GIPC PDZ domain containing family, member 1	1.7923824	1.3898963	1.1567227
GJB1	gap junction protein, beta 1, 32kDa	-6.0474734	5.2302847	11.836047
GKAP1	G kinase anchoring protein 1	-1.3072324	1.0661777	2.2827637
GLA	galactosidase, alpha	-1.0112913	-2.3390925	-2.526959
GLCCI1	glucocorticoid induced transcript 1	2.167406	-1.2083778	1.5789256
GLDC	glycine dehydrogenase (decarboxylating)	1.2182803	6.355515	17.246128
GLI3	GLI family zinc finger 3	-1.603655	-2.6883461	-4.8130674
GLIDR	glioblastoma down-regulated RNA	1.3521807	1.7226651	1.5225619
GLIDR	glioblastoma down-regulated RNA	1.6367685	2.6145072	2.3426006
GLIPR1	GLI pathogenesis-related 1	1.4081386	-10.826599	-32.74487
GLIPR2	GLI pathogenesis-related 2	1.783996	-2.2335594	-2.7715282
GLIPR2	GLI pathogenesis-related 2	1.3198118	-2.0518627	-2.0314329
GLIS1	GLIS family zinc finger 1	-1.1307204	-1.6303962	-2.4628553
GLOD4	glyoxalase domain containing 4	-1.0282283	1.1848078	1.5700097
GLP2R	glucagon-like peptide 2 receptor	-1.3162384	-1.4254053	-2.9787776
GLRX	glutaredoxin (thioltransferase)	1.3515482	3.4239943	3.0115387
GLRX3	glutaredoxin 3	1.2963594	-1.3518069	-1.5931158
GLRX5	glutaredoxin 5	1.7352614	2.3993635	2.5728726
GLS	glutaminase	-1.15989	-1.5514784	-1.8041146
GLS	glutaminase	-1.1695681	-3.6497252	-6.7494392
GLS2	glutaminase 2 (liver, mitochondrial)	-1.2896043	1.2059625	10.648727
GLTPD2	glycolipid transfer protein domain containing 2	-1.9914342	3.6176772	5.6042924
GLTSCR2	glioma tumor suppressor candidate region gene 2	-1.107879	-1.0236961	-1.7968386
GLUD1	glutamate dehydrogenase 1	-1.2897877	1.2373731	2.7784073
GLUD1P3	glutamate dehydrogenase 1 pseudogene 3	3.0154796	3.4914079	4.026219
GLUD2	glutamate dehydrogenase 2	-1.1449237	1.3405093	3.1814642
GLUL	glutamate-ammonia ligase	2.267929	5.283875	4.082788
GLYAT	glycine-N-acyltransferase	-1.4796553	50.844566	227.32242
GLYAT	glycine-N-acyltransferase	1.7223427	6.501755	15.355143
GLYATL1	glycine-N-acyltransferase-like 1	1.0065489	33.378147	37.201946
GLYCTK	glycerate kinase	1.0316379	9.115101	13.759201
GLYCTK	glycerate kinase	-2.325733	4.857374	7.30022
GM2A	GM2 ganglioside activator	1.5273496	-1.035375	-2.0045273
GMNN	geminin, DNA replication inhibitor	1.7289543	-1.3874471	-1.3534864
GMPR	guanosine monophosphate reductase	3.4014075	4.8366194	3.9444447
GMPR2	guanosine monophosphate reductase 2	1.2220681	1.8623154	1.324135
GMPR2	guanosine monophosphate reductase 2	1.6848582	2.3015335	1.8297522
GMPS	guanine monphosphate synthase	1.2221001	-1.2868438	-1.532071
GNA12	guanine nucleotide binding protein (G protein) alpha 12	-1.4895444	-2.0506139	-1.58896
GNA13	guanine nucleotide binding protein (G protein), alpha 13	-1.5901746	-2.2434986	-1.7378231
GNAI2	guanine nucleotide binding protein (G protein), alpha inhibiting activity polypeptide 2	1.3879895	1.0582285	1.6496038
GNAI3	guanine nucleotide binding protein (G protein), alpha inhibiting activity polypeptide 3	1.2920774	1.2135881	1.9151707
GNAO1	guanine nucleotide binding protein (G protein), alpha activating activity polypeptide O	1.8621274	1.9394535	1.9160026
GNAZ	guanine nucleotide binding protein (G protein), alpha z polypeptide	-1.1120031	-1.9466966	-1.5623845
GNB1L	guanine nucleotide binding protein (G protein), beta polypeptide 1-like	1.2450199	1.0044242	1.8087287
GNE	glucosamine (UDP-N-acetyl)-2-epimerase/N-acetylmannosamine kinase	-1.1352944	1.3150411	1.9698602
GNG10	guanine nucleotide binding protein (G protein), gamma 10	-1.1771427	-1.9872568	-1.3021029
GNG10	guanine nucleotide binding protein (G protein), gamma 10	1.6680818	1.1032094	1.6440322
GNG4	guanine nucleotide binding protein (G protein), gamma 4	1.2751113	-4.270746	-11.464582
GNL1	guanine nucleotide binding protein-like 1	-1.2026794	-1.2702426	-1.8097204
GNL2	guanine nucleotide binding protein-like 2 (nucleolar)	-1.1248869	-1.5236174	-1.9780171
GNL3L	guanine nucleotide binding protein-like 3 (nucleolar)-like	-1.2462415	-1.2408868	-2.047048
GNL3L	guanine nucleotide binding protein-like 3 (nucleolar)-like	-1.1294727	-1.1806368	-2.0543404
GNMT	glycine N-methyltransferase	1.097819	11.421196	199.33806
GNPTAB	N-acetylglucosamine-1-phosphate transferase, alpha and beta subunits	1.0350897	-1.8675114	-1.6349226
GOLGA2P5	golgin A2 pseudogene 5	-1.7890908	-1.7501293	-1.6560007
GOLGA2P6	golgin A2 pseudogene 6	-2.5186656	-2.3353474	-1.8294842
GOLGA2P7	golgin A2 pseudogene 7	-1.500639	-1.7104142	-1.125371
GOLGA2P7	golgin A2 pseudogene 7	2.6478279	2.761437	3.3093739
GOLGA3	golgin A3	-1.2364069	-1.4709073	-1.618579
GOLGA6L4	golgin A6 family-like 4	-3.4345593	-3.539499	-3.9499478
GOLGA6L4	golgin A6 family-like 4	-2.947577	-2.5242357	-2.087752
GOLGA6L9	golgin A6 family-like 9	-2.8405747	-2.2570736	-1.5314451
GOLGA6L9	golgin A6 family-like 9	-2.7109582	-2.3791142	-1.9808382
GOLGA8R	golgin A8 family, member R	-2.401241	-3.4416323	-3.5855067
GOLGB1	golgin B1	-3.7237415	-2.4518304	-2.501372
GOLM1	golgi membrane protein 1	-1.452718	-1.8574283	-1.2731448
GOLPH3L	golgi phosphoprotein 3-like	1.2008051	1.931775	1.513554
GOLT1A	golgi transport 1A	-1.2158064	3.7961624	4.420178
GOPC	golgi-associated PDZ and coiled-coil motif containing	1.6124405	-1.081477	-1.0994632
GOSR1	golgi SNAP receptor complex member 1	1.190059	1.2932717	1.5514987
GOT1	glutamic-oxaloacetic transaminase 1, soluble	1.6996853	2.3829305	1.9438666
GP1BB	glycoprotein Ib (platelet), beta polypeptide	-1.4944066	-2.768521	-10.744359
GP1BB	glycoprotein Ib (platelet), beta polypeptide	-1.1526692	-1.8519241	-3.8017092
GP9	glycoprotein IX (platelet)	1.8259275	2.1380324	2.6918387
GPANK1	G patch domain and ankyrin repeats 1	1.3347363	1.6572928	1.4975053
GPATCH4	G patch domain containing 4	-1.1496227	-1.2359706	-1.773778
GPATCH4	G patch domain containing 4	1.750699	1.5290451	1.0173051
GPC1	glypican 1	1.4402441	-1.670177	-1.9954147
GPC2	glypican 2	-1.8654395	-3.1354222	-3.2450116
GPC4	glypican 4	1.7122082	1.177059	-1.2137339
GPCPD1	glycerophosphocholine phosphodiesterase GDE1 homolog (S. cerevisiae)	-1.238934	1.0072948	2.0947096
GPD1	glycerol-3-phosphate dehydrogenase 1 (soluble)	-1.7600297	3.5334532	7.9280767
GPD2	glycerol-3-phosphate dehydrogenase 2 (mitochondrial)	1.466743	-1.4035403	-2.1677043
GPER1	G protein-coupled estrogen receptor 1	-1.2463137	2.0167196	2.596968
GPER1	G protein-coupled estrogen receptor 1	-1.4120395	1.7954456	2.1946185
GPHN	gephyrin	1.2974411	1.9393362	2.186665
GPI	glucose-6-phosphate isomerase	1.5595515	1.7789781	1.2175249
GPM6B	glycoprotein M6B	59.173935	106.5449	35.86759
GPM6B	glycoprotein M6B	27.569302	56.356464	13.930653
GPN2	GPN-loop GTPase 2	1.645348	1.448593	2.22974
GPN3	GPN-loop GTPase 3	1.5314294	1.2402972	1.583968
GPR119	G protein-coupled receptor 119	1.7627441	2.1812568	2.763009
GPR125	G protein-coupled receptor 125	-1.2312957	-1.0220664	1.569726
GPR152	G protein-coupled receptor 152	1.9025714	1.919849	1.6722398
GPR155	G protein-coupled receptor 155	-1.3803957	1.4141083	2.1025295
GPR155	G protein-coupled receptor 155	-1.3995928	1.4383932	2.0933776
GPR25	G protein-coupled receptor 25	1.4766275	2.4721935	4.1063704
GPR56	G protein-coupled receptor 56	-4.118652	-9.801097	-55.386223
GPR87	G protein-coupled receptor 87	-1.2130384	-2.776108	-10.246007
GPR88	G protein-coupled receptor 88	-1.0979259	6.7140446	5.655978
GPR88	G protein-coupled receptor 88	1.9743934	2.128652	2.434463
GPR98	G protein-coupled receptor 98	-2.4721518	4.2266145	5.581701
GPRASP2	G protein-coupled receptor associated sorting protein 2	1.1010165	1.4598767	2.3021483
GPRC5B	G protein-coupled receptor, class C, group 5, member B	-1.8850638	-1.8291686	-11.472256
GPRC5C	G protein-coupled receptor, class C, group 5, member C	-1.161831	2.4336698	3.382948
GPRC5C	G protein-coupled receptor, class C, group 5, member C	-1.0320526	1.6505921	1.8658518

GPSM1	G-protein signaling modulator 1	1.1853521	-1.3076203	-2.970818
GPSM1	G-protein signaling modulator 1	1.9295697	1.8411599	1.8154117
GPSM3	G-protein signaling modulator 3	1.6441945	1.8373709	2.2558823
GPT	glutamic-pyruvate transaminase (alanine aminotransferase)	1.8107401	14.25147	26.637695
GPX1	glutathione peroxidase 1	1.6971602	1.6250961	1.2553424
GPX2	glutathione peroxidase 2 (gastrointestinal)	-9.704315	-2.9396968	2.6712165
GPX3	glutathione peroxidase 3 (plasma)	-1.3243089	2.5535464	3.9812593
GPX3	glutathione peroxidase 3 (plasma)	-1.4951847	2.0553274	3.1474218
GPX4	glutathione peroxidase 4	-1.0650501	1.2165524	1.8373708
GPX8	glutathione peroxidase 8 (putative)	1.5759869	-1.1525421	-1.2647254
GPX8	glutathione peroxidase 8 (putative)	1.1165268	-2.2079806	-2.4512901
GRAMD1C	GRAM domain containing 1C	-1.4214032	3.0190418	5.409701
GRAMD4	GRAM domain containing 4	-1.3717595	-1.4174072	-1.7055589
GRB14	growth factor receptor-bound protein 14	-6.420382	-2.1951866	-1.9626863
GRB7	growth factor receptor-bound protein 7	-1.3266276	-1.243929	-1.6477563
GRHPR	glyoxylate reductase/hydroxypyruvate reductase	1.5246203	3.49438	4.599132
GRIA3	glutamate receptor, ionotropic, AMPA 3	-4.5287604	-1.7232777	-4.317273
GRIA3	glutamate receptor, ionotropic, AMPA 3	-4.239099	-1.6588326	-4.0066967
GRIN2C	glutamate receptor, ionotropic, N-methyl D-aspartate 2C	1.294429	-1.6545869	-4.25639
GRIPAP1	GRIP1 associated protein 1	-1.4853269	-1.7111121	-1.2456543
GRK5	G protein-coupled receptor kinase 5	-1.9025245	-1.1028388	1.8961734
GRPEL2	GrpE-like 2, mitochondrial (E. coli)	-1.0317013	-1.4509586	-1.962293
GRTP1	growth hormone regulated TBC protein 1	-1.8307121	1.9384409	3.0407767
GSDMB	gasdermin B	-2.993245	-2.8313782	1.045418
GSDMD	gasdermin D	-1.0256093	1.4666946	1.5018528
GSG1	germ cell associated 1	-2.712699	-2.019354	-1.7144604
GSK3A	glycogen synthase kinase 3 alpha	1.5045108	1.5498947	1.4961321
GSK3B	glycogen synthase kinase 3 beta	-1.2595195	-1.5698532	-1.3189563
GSTA2	glutathione S-transferase alpha 2	-1.0724266	4.281555	8.336945
GSTA5	glutathione S-transferase alpha 5	1.271446	5.732731	12.471652
GSTK1	glutathione S-transferase kappa 1	1.2784901	1.9150774	2.2530596
GSTK1	glutathione S-transferase kappa 1	1.2653885	2.0891404	2.3739038
GSTO1	glutathione S-transferase omega 1	1.1479976	1.642855	2.5163238
GSTT1	glutathione S-transferase theta 1	1.1120359	2.3005793	2.3096805
GSTT2	glutathione S-transferase theta 2 (gene/pseudogene)	1.0976051	1.5329281	2.9586432
GSTT2B	glutathione S-transferase theta 2B (gene/pseudogene)	1.2023007	1.4862516	2.816698
GSTZ1	glutathione S-transferase zeta 1	1.006106	1.3276128	2.611354
GTF2A2	general transcription factor IIA, 2, 12kDa	1.7781482	1.3026686	1.3788915
GTF2H5	general transcription factor IIH, polypeptide 5	1.466534	1.3814504	1.6669735
GTPBP2	GTP binding protein 2	-1.8865213	-2.1761312	-2.4176416
GTPBP4	GTP binding protein 4	1.5752919	1.0455883	-1.2350925
GTSE1-AS1	GTSE1 antisense RNA 1 (head to head)	1.2162182	1.5599844	1.74915
GULP1	GULP, engulfment adaptor PTB domain containing 1	1.1327279	3.1688075	1.593056
GUSBP1	glucuronidase, beta pseudogene 1	-2.7422884	-2.090246	-1.8045589
GUSBP1	glucuronidase, beta pseudogene 1	-2.1912913	-1.6194736	-1.7084354
GYPA	glycophorin A (MNS blood group)	1.3524973	2.2823899	3.1358511
GYPC	glycophorin C (Gerbich blood group)	-1.2164364	-1.0501724	-3.5455396
GYS1	glycogen synthase 1 (muscle)	2.1574202	2.1650555	1.7923985
GZMM	granzyme M (lymphocyte met-ase 1)	1.6251762	1.6885545	2.2026122
H19	H19, imprinted maternally expressed transcript (non-protein coding)	15.817989	5.5985494	1.2816899
H19	H19, imprinted maternally expressed transcript (non-protein coding)	40.92466	12.783794	2.3809235
H1F0	H1 histone family, member 0	-1.2714132	-1.2363192	2.647681
H2AFV	H2A histone family, member V	1.7095937	-1.2810287	1.5116326
H2AFX	H2A histone family, member X	2.232558	-1.7213018	-1.156795
H2AFY	H2A histone family, member Y	-1.2485743	-2.1335335	-2.6203065
H2AFZ	H2A histone family, member Z	1.2643262	-2.929794	-2.2722995
H3F3A	H3 histone, family 3A	1.54724	1.3130575	1.6417136
H3F3A	H3 histone, family 3A	1.4990761	1.2231371	1.6002501
H3F3C	H3 histone, family 3C	-1.2442834	-1.8053515	1.026094
HAAO	3-hydroxyanthranilate 3,4-dioxygenase	1.2069257	2.7034593	6.5424995
HAAO	3-hydroxyanthranilate 3,4-dioxygenase	-1.1639134	2.3406854	6.838064
HABP2	hyaluronan binding protein 2	-24.520197	2.124273	5.719767
HABP4	hyaluronan binding protein 4	1.1577055	1.030715	1.5423735
HACL1	2-hydroxyacyl-CoA lyase 1	-1.1268516	1.3334384	3.1357694
HADH	hydroxyacyl-CoA dehydrogenase	1.1379583	1.2750502	2.650193
HAGH	hydroxyacylglutathione hydrolase	1.1316818	2.033975	6.2572026
HAO1	hydroxyacid oxidase (glycolate oxidase) 1	-5.324315	6.025444	18.340807
HAO2	hydroxyacid oxidase 2 (long chain)	-2.221304	38.697285	53.280884
HAR1A	highly accelerated region 1A (non-protein coding)	2.0930126	2.2431521	2.6102178
HAUS1	HAUS augmin-like complex, subunit 1	-1.0479059	-2.2644203	-2.451328
HAUS5	HAUS augmin-like complex, subunit 5	-1.207691	-2.8356693	-2.3401237
HAUS6	HAUS augmin-like complex, subunit 6	1.9737009	-1.3005774	-1.7897325
HAUS7	HAUS augmin-like complex, subunit 7	1.0696036	-1.7906687	-1.9783756
HBP1	HMG-box transcription factor 1	-2.2052093	-1.5712483	-1.1871711
HBS1L	HBS1-like translational GTPase	1.4262989	1.2778772	1.5023345
HBZ	hemoglobin, zeta	2.2749724	2.4623077	2.6639397
HCCS	holocytochrome c synthase	1.2097687	-1.0513501	-1.6126238
HCFC2	host cell factor C2	1.5211794	2.232996	2.8800302
HCG18	HLA complex group 18 (non-protein coding)	-1.407606	-1.3144184	-2.189188
HCG18	HLA complex group 18 (non-protein coding)	-1.1053262	1.0836251	1.854136
HCG18	HLA complex group 18 (non-protein coding)	3.291175	2.1778486	2.0013094
HCG18	HLA complex group 18 (non-protein coding)	-1.1813631	1.2124293	3.2427077
HCG4B	HLA complex group 4B (non-protein coding)	-1.0711381	1.5184481	2.7549288
HCN2	hyperpolarization activated cyclic nucleotide gated potassium channel 2	1.2059921	1.8003947	1.9965423
HDAC3	histone deacetylase 3	1.4384035	1.235076	1.8243461
HDAC6	histone deacetylase 6	-1.7109871	1.4121574	1.5646619
HDAC7	histone deacetylase 7	-1.0366123	-1.4117851	-2.3408053
HDDC3	HD domain containing 3	1.3467823	1.7839224	1.6004485
HDGF	hepatoma-derived growth factor	-1.1538849	-1.176676	-1.8314881
HDGFRP2	hepatoma-derived growth factor-related protein 2	3.1441789	3.2673254	3.378878
HDHD2	haloacid dehalogenase-like hydrolase domain containing 2	1.5261378	1.8791574	3.114284
HDLBP	high density lipoprotein binding protein	-1.2813455	-1.3635207	-1.5708802
HEATR1	HEAT repeat containing 1	-1.1750097	-1.3052069	-2.4671135
HEBP1	heme binding protein 1	1.8204716	2.5387323	2.5499542
HECTD3	HECT domain containing E3 ubiquitin protein ligase 3	-2.0456839	-1.0032467	-1.0933928
HEG1	heart development protein with EGF-like domains 1	2.5609012	1.28113	-1.2539802
HELZ2	helicase with zinc finger 2, transcriptional coactivator	-1.520944	-1.223012	1.1249058
HERC1	HECT and RLD domain containing E3 ubiquitin protein ligase family member 1	-1.9167703	-1.1685534	-1.2441758
HERC2P2	hect domain and RLD 2 pseudogene 2	-1.6700205	-1.8147949	-1.4754418
HERC4	HECT and RLD domain containing E3 ubiquitin protein ligase 4	-1.1036627	-1.6302536	-1.4604276
HERPUD1	homocysteine-inducible, endoplasmic reticulum stress-inducible, ubiquitin-like domain memb	1.0667201	1.8530374	1.9096478
HES4	hes family bHLH transcription factor 4	-1.4734052	-1.8844709	-3.640428
HES6	hes family bHLH transcription factor 6	1.6964301	1.9571011	2.2243981
HES6	hes family bHLH transcription factor 6	1.2120878	1.2883372	2.6477473
HEXIM1	hexamethylene bis-acetamide inducible 1	-1.0477353	-1.1534982	1.6120108
HEXIM2	hexamethylene bis-acetamide inducible 2	1.3908548	2.7544203	3.0550735
HFE2	hemochromatosis type 2 (juvenile)	-2.4419746	11.931589	8.633455
HGD	homogentisate 1,2-dioxygenase	-1.479005	1.6895994	4.088133
HHAT	hedgehog acyltransferase	-2.1141877	-2.3321161	-1.3990654

HHEX	hematopoietically expressed homeobox	-1.8365693	1.7124778	3.371973
HIBCH	3-hydroxyisobutyryl-CoA hydrolase	-1.1141946	1.1183659	1.6517785
HIC2	hypermethylated in cancer 2	-1.010099	-1.3889172	-1.6809733
HIF1A	hypoxia inducible factor 1, alpha subunit (basic helix-loop-helix transcription factor)	1.6169987	-1.3918127	-3.2054043
HIF1A	hypoxia inducible factor 1, alpha subunit (basic helix-loop-helix transcription factor)	-1.1066368	-2.8251872	-6.426299
HIF3A	hypoxia inducible factor 3, alpha subunit	3.6438751	4.3167377	5.0716844
HIGD1A	HIG1 hypoxia inducible domain family, member 1A	1.7817757	1.8569837	2.4657352
HIGD1A	HIG1 hypoxia inducible domain family, member 1A	1.5083193	1.8124195	2.4259167
HIGD1A	HIG1 hypoxia inducible domain family, member 1A	2.8997953	5.169891	6.76116
HILPDA	hypoxia inducible lipid droplet-associated	2.0943434	1.466889	1.1657147
HINT2	histidine triad nucleotide binding protein 2	1.2702533	2.1544402	2.151034
HIPK2	homeodomain interacting protein kinase 2	1.1652395	-1.0888779	-1.9172455
HIPK2	homeodomain interacting protein kinase 2	-1.424185	-1.2628919	-2.067412
HIRA	histone cell cycle regulator	1.858477	1.9645199	1.8395519
HIST1H1B	histone cluster 1, H1b	2.9507499	-2.7266262	-6.2981954
HIST1H1C	histone cluster 1, H1c	1.193174	1.4638784	1.7340517
HIST1H2AB	histone cluster 1, H2ab	3.315537	1.7394086	1.253274
HIST1H2AC	histone cluster 1, H2ac	1.0906932	1.9719249	4.81205
HIST1H2AC	histone cluster 1, H2ac	1.8697295	1.8568892	2.4135835
HIST1H2AD	histone cluster 1, H2ad	1.2754967	2.0643156	2.220629
HIST1H2AE	histone cluster 1, H2ae	1.5301836	1.7465965	2.1435132
HIST1H2AI	histone cluster 1, H2ai	3.1446474	-2.532661	-3.5367472
HIST1H2BB	histone cluster 1, H2bb	1.0863458	1.2255318	1.5967366
HIST1H2BC	histone cluster 1, H2bc	1.2032534	1.2927307	1.8360989
HIST1H2BD	histone cluster 1, H2bd	1.0718594	1.1893916	1.8182985
HIST1H2BG	histone cluster 1, H2bg	1.1950902	1.2487038	1.6407685
HIST1H2BI	histone cluster 1, H2bi	1.1448816	1.2705252	1.6855195
HIST1H2BJ	histone cluster 1, H2bj	1.3177724	1.0007083	1.8576744
HIST1H2BK	histone cluster 1, H2bk	1.068118	1.2737999	1.628594
HIST1H2BK	histone cluster 1, H2bk	1.0454096	1.1825615	1.5797073
HIST1H2BL	histone cluster 1, H2bl	-1.1249304	-1.0031959	1.5018995
HIST1H2BM	histone cluster 1, H2bm	1.0015166	1.086941	1.5337459
HIST1H2BN	histone cluster 1, H2bn	1.5037736	1.3080887	1.5545342
HIST1H2BO	histone cluster 1, H2bo	-1.1885802	-1.0363349	1.5358531
HIST1H3A	histone cluster 1, H3a	1.7047726	1.295305	1.7392704
HIST1H3B	histone cluster 1, H3b	4.9038644	-1.52572	-1.763826
HIST1H3C	histone cluster 1, H3c	1.1442224	-1.5373896	1.2023796
HIST1H3D	histone cluster 1, H3d	3.4371812	1.0352621	1.4371312
HIST1H3E	histone cluster 1, H3e	1.842182	1.0878136	1.5205055
HIST1H3F	histone cluster 1, H3f	3.6486533	1.0264503	1.3311306
HIST1H3G	histone cluster 1, H3g	1.5109835	-1.9694861	-1.2175149
HIST1H3H	histone cluster 1, H3h	3.0373983	-1.2400838	-1.0077847
HIST1H3J	histone cluster 1, H3j	5.562699	3.0390255	4.1108875
HIST1H4A	histone cluster 1, H4a	2.9218178	1.1407858	1.7791674
HIST1H4B	histone cluster 1, H4b	3.5780213	1.5089114	1.6536291
HIST1H4C	histone cluster 1, H4c	2.94427	1.3798062	1.4089075
HIST1H4D	histone cluster 1, H4d	5.6911564	2.3572643	2.2487502
HIST1H4F	histone cluster 1, H4f	1.5015507	1.2665427	3.1465058
HIST1H4H	histone cluster 1, H4h	1.7655237	-1.092154	1.5918506
HIST1H4I	histone cluster 1, H4i	1.6711166	-1.1205279	1.7611232
HIST1H4J	histone cluster 1, H4j	1.4305922	1.5448298	3.2145603
HIST1H4K	histone cluster 1, H4k	1.590585	-1.18126	1.6835237
HIST1H4K	histone cluster 1, H4k	1.1178476	1.2669699	3.6211333
HIST1H4L	histone cluster 1, H4l	2.7289708	1.1940321	1.248258
HIST2H2AA4	histone cluster 2, H2aa4	1.4695804	3.1908624	3.0346494
HIST2H2BF	histone cluster 2, H2bf	1.5597086	1.563689	2.0011454
HIST2H3D	histone cluster 2, H3d	1.0675557	-2.4469774	-1.4603525
HIST2H4B	histone cluster 2, H4b	1.924099	1.235989	2.7265077
HIST3H3	histone cluster 3, H3	1.4324311	1.0757214	1.5152318
HIVEP2	human immunodeficiency virus type I enhancer binding protein 2	-1.216824	-1.4644827	-1.9803339
HJURP	Holliday junction recognition protein	2.6934023	-4.3254447	-3.7388182
HKDC1	hexokinase domain containing 1	-3.190588	-2.616603	-3.4842672
HKDC1	hexokinase domain containing 1	-2.3505337	-1.7844845	-2.1647284
HLA-A	major histocompatibility complex, class I, A	-2.0804853	-1.9235767	-1.5692239
HLA-DMA	major histocompatibility complex, class II, DM alpha	1.2179872	-1.0560944	-2.3285363
HLA-DMA	major histocompatibility complex, class II, DM alpha	1.0210055	-1.0845681	-2.335716
HLA-DMB	major histocompatibility complex, class II, DM beta	1.0907773	1.6081175	1.4165893
HLA-DRA	major histocompatibility complex, class II, DR alpha	-4.3520894	-1.358804	-1.9488064
HLA-DRB3	major histocompatibility complex, class II, DR beta 3	1.7633114	1.8505708	2.213258
HLA-DRB4	major histocompatibility complex, class II, DR beta 4	-1.7033608	-1.6391557	-1.3753546
HLA-DRB5	major histocompatibility complex, class II, DR beta 5	-1.796278	-1.5498407	-1.1261541
HLA-L	major histocompatibility complex, class I, L (pseudogene)	1.3557968	1.4069737	1.5342592
HLF	hepatic leukemia factor	-1.1040055	8.667857	27.694878
HLTF	helicase-like transcription factor	-1.4458474	-1.9610819	-1.7499808
HM13	histocompatibility (minor) 13	-1.7715194	-1.4181951	-1.6222086
HMCES	5-hydroxymethylcytosine (hmC) binding, ES cell-specific	-1.2725793	1.7006114	2.22677
HMG20B	high mobility group 20B	1.9365923	2.297445	2.6730819
HMG20B	high mobility group 20B	1.3914664	1.3606441	1.9022084
HMGA1	high mobility group AT-hook 1	-6.2322326	-8.712003	-13.3724985
HMGB1	high mobility group box 1	-1.4372172	-2.5077457	-1.9381363
HMGB2	high mobility group box 2	1.7623069	-2.1052	-1.271612
HMGB3	high mobility group box 3	1.7886384	-1.4945352	-1.134425
HMGB3	high mobility group box 3	1.7114487	-1.481416	-1.2088437
HMGB3P1	high mobility group box 3 pseudogene 1	3.1124864	1.3038709	1.242114
HMGCL	3-hydroxymethyl-3-methylglutaryl-CoA lyase	-1.4972531	1.8918271	3.1554322
HMGCR	3-hydroxy-3-methylglutaryl-CoA reductase	1.0952171	-1.0394591	1.9728985
HMGCS1	3-hydroxy-3-methylglutaryl-CoA synthase 1 (soluble)	1.4556772	1.4224138	3.0142298
HMGCS2	3-hydroxy-3-methylglutaryl-CoA synthase 2 (mitochondrial)	-19.08319	4.5177064	5.196171
HMGN2	high mobility group nucleosomal binding domain 2	1.7524304	1.1874832	1.8370857
HMGXB3	HMG box domain containing 3	-1.0617839	-1.4779378	-1.7637175
HMGXB4	HMG box domain containing 4	-1.0318211	-1.645975	-1.2569699
HMHB1	histocompatibility (minor) HB-1	3.2621577	3.3101733	3.9587238
HMMR	hyaluronan-mediated motility receptor (RHAMM)	3.3618965	-1.6947916	-2.3066735
HMOX2	heme oxygenase (decycling) 2	1.0986866	1.9194686	1.5137589
HN1	hematological and neurological expressed 1	1.797014	-1.274139	1.3222618
HN1L	hematological and neurological expressed 1-like	1.6051835	1.4407213	1.2418189
HN1L	hematological and neurological expressed 1-like	1.2038474	-1.206481	-1.7397212
HNF1A	HNF1 homeobox A	-1.3916836	2.0244453	1.5648972
HNMT	histamine N-methyltransferase	-1.7042627	-1.2305269	-1.4122814
HNRNPA0	heterogeneous nuclear ribonucleoprotein A0	1.9397374	1.578082	1.8018365
HNRNPA1	heterogeneous nuclear ribonucleoprotein A1	1.667671	1.331791	1.1044769
HNRNPA1L2	heterogeneous nuclear ribonucleoprotein A1-like 2	1.708414	-1.0080596	-1.2742169
HNRNPA1L2	heterogeneous nuclear ribonucleoprotein A1-like 2	1.2542561	-1.3118306	-1.7526428
HNRNPA1L2	heterogeneous nuclear ribonucleoprotein A1-like 2	1.6348448	-1.0152823	-1.289389
HNRNPA3	heterogeneous nuclear ribonucleoprotein A3	1.2828786	-1.2125921	-1.8208995
HNRNPA3	heterogeneous nuclear ribonucleoprotein A3	1.2350271	-1.4165548	-1.5953602
HNRNPA3	heterogeneous nuclear ribonucleoprotein A3	1.23175	-1.5911411	-1.8629845
HNRNPD	heterogeneous nuclear ribonucleoprotein D (AU-rich element RNA binding protein 1, 37kDa)	2.002092	-1.0536387	-1.326138
HNRNPDL	heterogeneous nuclear ribonucleoprotein D-like	1.2998009	-1.7790413	-2.5066206

HNRNPH3	heterogeneous nuclear ribonucleoprotein H3 (2H9)	-1.6311889	-2.9053996	-2.6419733
HNRNPLL	heterogeneous nuclear ribonucleoprotein L-like	1.6994318	1.4582188	1.6365708
HNRNPU	heterogeneous nuclear ribonucleoprotein U (scaffold attachment factor A)	-1.2273002	-1.655729	-1.6540685
HOGA1	4-hydroxy-2-oxoglutarate aldolase 1	11.5386715	36.942135	24.508982
HOMER2	homer homolog 2 (Drosophila)	-1.6107088	1.3518026	3.3534582
HOMER3	homer homolog 3 (Drosophila)	-1.3383452	-2.2847536	-4.6498427
HOMER3	homer homolog 3 (Drosophila)	1.2086266	-1.687736	-3.6844542
HOMER3	homer homolog 3 (Drosophila)	1.2357975	-1.6939771	-3.737188
HOOK3	hook microtubule-tethering protein 3	-2.178646	-2.0506904	-1.817906
HOPX	HOP homeobox	2.332325	6.2600884	1.0180242
HOTAIR	HOX transcript antisense RNA	-1.2719781	1.1558203	1.6827301
HOXA10	homeobox A10	1.7933915	2.2281928	2.3718545
HOXA3	homeobox A3	-1.5714244	1.7088569	2.194251
HOXB13	homeobox B13	1.8793331	3.0071867	1.6139947
HOXB9	homeobox B9	1.3025787	-1.6190714	-4.945534
HOXC9	homeobox C9	1.8440193	1.2452923	-1.2785065
HP	haptoglobin	-3.050097	1.864117	1.8945924
HPGD	hydroxyprostaglandin dehydrogenase 15-(NAD)	-3.1090183	1.9603758	1.9379672
HPN	hepsin	-4.7096663	3.701506	15.978892
HPR	haptoglobin-related protein	-5.0695457	2.3086379	2.3077848
HPS1	Hermansky-Pudlak syndrome 1	-1.498108	-1.7474265	-3.367118
HPS6	Hermansky-Pudlak syndrome 6	1.815101	1.5332855	1.6413398
HPX	hemopexin	-5.0236263	6.0113606	12.587253
HR	hair growth associated	1.840791	1.6002196	2.0533762
HRAS	Harvey rat sarcoma viral oncogene homolog	1.2440574	-1.3095757	-1.616514
HRASLS	HRAS-like suppressor	1.6352173	1.5235254	1.7613196
HRASLS5	HRAS-like suppressor family, member 5	1.9724891	1.8495848	1.8907434
HRCT1	histidine rich carboxyl terminus 1	2.0074248	2.6892197	4.3239474
HRG	histidine-rich glycoprotein	-4.559589	15.471137	56.525085
HRSP12	heat-responsive protein 12	-1.3285881	6.9988804	9.901845
HS1BP3-IT1	HS1BP3 intronic transcript 1 (non-protein coding)	1.9885123	2.1181796	2.5838308
HS6ST1	heparan sulfate 6-O-sulfotransferase 1	1.3766426	1.09786	2.0905507
HSBP1L1	heat shock factor binding protein 1-like 1	1.7257495	1.9333198	1.2916096
HSD11B1	hydroxysteroid (11-beta) dehydrogenase 1	-4.013833	6.168311	3.8453345
HSD11B2	hydroxysteroid (11-beta) dehydrogenase 2	-2.2652745	2.6442425	1.9586173
HSD17B10	hydroxysteroid (17-beta) dehydrogenase 10	1.1131206	1.5167546	1.9472204
HSD17B11	hydroxysteroid (17-beta) dehydrogenase 11	-1.600846	1.6809086	3.7366204
HSD17B12	hydroxysteroid (17-beta) dehydrogenase 12	1.2776029	1.7696557	1.9000549
HSD17B2	hydroxysteroid (17-beta) dehydrogenase 2	-2.6783133	1.8896883	-1.3641263
HSD17B4	hydroxysteroid (17-beta) dehydrogenase 4	-1.1412276	1.5025644	1.8403338
HSD17B6	hydroxysteroid (17-beta) dehydrogenase 6	-2.155405	21.508665	55.044827
HSD17B8	hydroxysteroid (17-beta) dehydrogenase 8	1.2150583	2.415353	2.2291071
HSD3B7	hydroxy-delta-5-steroid dehydrogenase, 3 beta- and steroid delta-isomerase 7	-1.8469558	1.4218074	1.6452414
HSF1	heat shock transcription factor 1	2.3675365	2.6056857	2.6851819
HSF1	heat shock transcription factor 1	1.518151	1.6684948	1.1382082
HSP90AA1	heat shock protein 90kDa alpha (cytosolic), class A member 1	-1.4497874	-1.8343092	-1.7245152
HSP90AA2P	heat shock protein 90kDa alpha (cytosolic), class A member 2, pseudogene	-1.7772441	-2.145554	-2.0966232
HSP90AB1	heat shock protein 90kDa alpha (cytosolic), class B member 1	-1.572201	-1.7778491	-2.3810596
HSP90AB1	heat shock protein 90kDa alpha (cytosolic), class B member 1	-2.2450674	-2.7812815	-3.8805783
HSP90B1	heat shock protein 90kDa beta (Grp94), member 1	-1.5786974	-1.879836	-2.0369508
HSPA14	heat shock 70kDa protein 14	1.1614046	-1.6118044	-1.3081672
HSPA4L	heat shock 70kDa protein 4-like	-1.0686555	-1.1454577	-1.866188
HSPA4L	heat shock 70kDa protein 4-like	1.0012543	-1.1067466	-1.87591
HSPA4L	heat shock 70kDa protein 4-like	1.1821647	-1.4917225	-1.8492918
HSPB1	heat shock 27kDa protein 1	1.29036	-1.743055	-2.1501856
HSPB3	heat shock 27kDa protein 3	4.0258336	2.0488892	-10.44674
HSPB8	heat shock 22kDa protein 8	-1.1936158	-1.4655693	-2.126455
HSPBAP1	HSPB (heat shock 27kDa) associated protein 1	-1.2093604	-1.16936	-2.4847136
HSPE1	heat shock 10kDa protein 1	1.4456805	1.5332989	1.3765011
HSPG2	heparan sulfate proteoglycan 2	-2.4712021	-4.6552787	-11.660844
HSPH1	heat shock 105kDa/110kDa protein 1	-1.1092725	-1.39332	-1.6428034
HSPH1	heat shock 105kDa/110kDa protein 1	-1.6247772	-2.2004662	-2.357956
HTATSF1	HIV-1 Tat specific factor 1	-1.3973325	-1.6101309	-1.4133892
HTT	huntingtin	-1.9780197	-1.9020691	-1.8797772
HULC	hepatocellular carcinoma up-regulated long non-coding RNA	-9.86217	6.1130714	8.031609
HUWE1	HECT, UBA and WWE domain containing 1, E3 ubiquitin protein ligase	-1.9045838	-1.6509237	-2.5976784
HYAL1	hyaluronoglucosaminidase 1	1.6865569	3.5957282	4.2667837
HYAL4	hyaluronoglucosaminidase 4	2.066791	2.2763782	2.3412044
HYI	hydroxypyruvate isomerase (putative)	-1.6635036	-2.2620573	-1.9451181
HYI	hydroxypyruvate isomerase (putative)	-1.5065299	-1.90589	-1.771225
HYKK	hydroxylysine kinase	1.7892967	1.8046892	2.6564522
IAH1	isoamyl acetate-hydrolyzing esterase 1 homolog (S. cerevisiae)	1.4853363	1.5895295	1.4478488
IAPP	islet amyloid polypeptide	2.9130843	2.819523	4.120904
IARS	isoleucyl-tRNA synthetase	-1.1930505	-1.1895838	-2.2542806
IARS	isoleucyl-tRNA synthetase	-1.4468491	-1.4145961	-2.8402872
ICAM1	intercellular adhesion molecule 1	-2.1704237	-3.1706932	-11.321669
ICAM3	intercellular adhesion molecule 3	1.02302	1.62558	1.3541167
ICK	intestinal cell (MAK-like) kinase	1.5930513	1.2436517	1.5304614
ICMT	isoprenylcysteine carboxyl methyltransferase	1.5706239	-1.0787053	1.5054512
ID1	inhibitor of DNA binding 1, dominant negative helix-loop-helix protein	2.3707001	2.6836894	7.1494284
ID2	inhibitor of DNA binding 2, dominant negative helix-loop-helix protein	-4.441088	-1.365003	1.8870823
ID2	inhibitor of DNA binding 2, dominant negative helix-loop-helix protein	-2.2086265	1.4022835	3.59911
ID3	inhibitor of DNA binding 3, dominant negative helix-loop-helix protein	-1.5450593	-1.7459619	-1.2199726
IDE	insulin-degrading enzyme	-1.2045485	-1.5458608	-1.6167909
IDH1	isocitrate dehydrogenase 1 (NADP+), soluble	-1.1820487	1.3597133	2.7276864
IDH2	isocitrate dehydrogenase 2 (NADP+), mitochondrial	1.143884	1.278878	-1.9025887
IDNK	idnK, gluconokinase homolog (E. coli)	1.1702797	3.1945736	5.4275513
IDS	iduronate 2-sulfatase	1.0821944	-1.6666404	-1.6873122
IDUA	iduronidase, alpha-L-	-1.5991539	-1.3449017	-1.1736126
IER3	immediate early response 3	-6.321384	-11.762157	-17.090654
IER3	immediate early response 3	-7.2341814	-13.923927	-21.057833
IER5	immediate early response 5	-1.1457459	-1.5085598	-1.7790949
IFFO1	intermediate filament family orphan 1	1.4779116	1.2229381	2.05983
IFFO2	intermediate filament family orphan 2	1.4171556	-1.6397207	-2.161032
IFI27	interferon, alpha-inducible protein 27	1.5696081	-1.2006155	-2.2712631
IFI27L2	interferon, alpha-inducible protein 27-like 2	1.624979	-1.1975952	-2.31449
IFI44	interferon-induced protein 44	-1.512348	-2.3503432	-1.512584
IFIH1	interferon induced with helicase C domain 1	-2.6621735	-1.5985003	-1.5681617
IFIT1	interferon-induced protein with tetratricopeptide repeats 1	2.3042905	3.0077758	3.196754
IFIT2	interferon-induced protein with tetratricopeptide repeats 2	-1.4070758	1.1880167	1.6495429
IFIT3	interferon-induced protein with tetratricopeptide repeats 3	-1.6638038	-1.7588091	-1.6366948
IFITM3	interferon induced transmembrane protein 3	1.485113	1.616476	-1.1176673
IFITM4P	interferon induced transmembrane protein 4 pseudogene	1.7867063	1.8861688	-1.0283921
IFNGR2	interferon gamma receptor 2 (interferon gamma transducer 1)	-1.8834145	-1.9646589	-2.6555805
IFRD1	interferon-related developmental regulator 1	-2.7086427	-2.9783168	-2.4263422
IFRD2	interferon-related developmental regulator 2	-1.1582396	-1.1366613	-1.5296931
IFT122	intraflagellar transport 122	-1.3105143	-1.8955365	-1.5006264
IFT20	intraflagellar transport 20	-1.7073202	-1.6146419	-1.4928383
IFT80	intraflagellar transport 80	1.0427127	1.9234166	-1.1257113

IFT88	intraflagellar transport 88	-1.8381449	-1.1845857	1.2191479
IGBP1	immunoglobulin (CD79A) binding protein 1	1.3318985	1.558576	1.2305025
IGF1R	insulin-like growth factor 1 receptor	-2.3837562	-3.7098134	-3.9142585
IGF2-AS	IGF2 antisense RNA	-1.6119936	3.0948122	3.2084534
IGF2BP2	insulin-like growth factor 2 mRNA binding protein 2	-1.4642136	-1.7080382	-1.6498299
IGF2BP2-AS1	IGF2BP2 antisense RNA 1	1.482908	1.3504215	3.9394584
IGF2BP3	insulin-like growth factor 2 mRNA binding protein 3	-5.436511	-10.128812	-11.070697
IGF2R	insulin-like growth factor 2 receptor	-2.3035932	-2.3067493	-1.8844297
IGFBP1	insulin-like growth factor binding protein 1	-95.53144	-70.141685	-27.561028
IGFBP2	insulin-like growth factor binding protein 2, 36kDa	1.1780704	4.656752	4.9369493
IGFBP3	insulin-like growth factor binding protein 3	-1.3915207	-5.6373553	-9.755324
IGFBP4	insulin-like growth factor binding protein 4	-1.0988722	-2.2568603	-2.4315524
IGFBP6	insulin-like growth factor binding protein 6	1.2159134	-2.0125082	-10.262916
IGFBP7	insulin-like growth factor binding protein 7	1.4896653	-1.5663702	-5.9616284
IGFL3	IGF-like family member 3	3.0087202	-10.525592	-85.725464
IGFLR1	IGF-like family receptor 1	1.5895984	3.8267674	2.5339465
IGSF9	immunoglobulin superfamily, member 9	3.800281	3.8403747	4.6756434
IHH	indian hedgehog	4.599266	5.102056	4.2817693
IKBIP	IKBKB interacting protein	-1.5854447	-3.1548364	-5.226452
IKBIP	IKBKB interacting protein	1.0176436	-2.2687654	-2.9425912
IKBKB	inhibitor of kappa light polypeptide gene enhancer in B-cells, kinase beta	-1.5824333	-1.5481715	-1.2774296
IKBKG	inhibitor of kappa light polypeptide gene enhancer in B-cells, kinase gamma	-1.5698072	-1.9170394	-1.9733559
IL11RA	interleukin 11 receptor, alpha	1.3245095	1.3350295	3.859862
IL13RA1	interleukin 13 receptor, alpha 1	1.353335	1.5584352	1.9457442
IL15	interleukin 15	-1.4680731	-1.5580766	-1.883
IL15RA	interleukin 15 receptor, alpha	-1.2416971	-1.2817457	-1.9057366
IL17RC	interleukin 17 receptor C	1.3178108	1.9687625	2.6217623
IL18	interleukin 18	1.8029763	2.1006181	-1.8228472
IL1R1	interleukin 1 receptor, type I	1.7651447	1.1750321	1.3834435
IL1R2	interleukin 1 receptor, type II	8.708017	11.394501	4.097135
IL22RA1	interleukin 22 receptor, alpha 1	-1.6340542	2.0716877	3.3527765
IL27RA	interleukin 27 receptor, alpha	-2.0320337	-1.7952236	-6.8376155
IL2RG	interleukin 2 receptor, gamma	1.1082041	3.4171278	11.973706
IL6R	interleukin 6 receptor	-1.1459899	1.6985419	5.785129
IL6ST	interleukin 6 signal transducer	-1.4669447	-1.218926	-1.5710481
IL7R	interleukin 7 receptor	4.270204	-1.9195602	-63.8353
ILF3	interleukin enhancer binding factor 3, 90kDa	-1.1890564	-2.8894289	-5.339027
ILF3	interleukin enhancer binding factor 3, 90kDa	-1.5633451	-1.6235039	-1.7195029
ILF3-AS1	ILF3 antisense RNA 1 (head to head)	-1.0630883	1.5232022	-1.1167793
ILVBL	ilvB (bacterial acetolactate synthase)-like	1.2186404	1.6744161	1.1052872
IMP3	IMP3, U3 small nucleolar ribonucleoprotein	1.7685467	1.7246462	2.3422155
IMPA1	inositol(myo)-1(or 4)-monophosphatase 1	1.2278801	1.3695014	2.425192
IMPAD1	inositol monophosphatase domain containing 1	2.016671	1.06667	1.1476215
IMPDH1	IMP (inosine 5'-monophosphate) dehydrogenase 1	1.1576035	-1.4207298	-2.089783
INAFM1	InaF-motif containing 1	-1.4082438	-1.2178903	-1.9550576
INF2	inverted formin, FH2 and WH2 domain containing	-1.5875359	-1.5899075	-2.9745183
INF2	inverted formin, FH2 and WH2 domain containing	-1.508551	-1.8047372	-8.705574
INF2	inverted formin, FH2 and WH2 domain containing	-1.3645405	-1.4892836	-2.4962342
INHBB	inhibin, beta B	1.4379735	1.3066164	-2.0272589
INIP	INTS3 and NABP interacting protein	1.4483539	1.680355	1.4830898
INIP	INTS3 and NABP interacting protein	1.6607764	1.406232	1.3312671
INPP5A	inositol polyphosphate-5-phosphatase, 40kDa	-1.1690698	1.0208428	1.6260002
INPP5D	inositol polyphosphate-5-phosphatase, 145kDa	1.0604341	-1.346252	-7.495946
INPP5K	inositol polyphosphate-5-phosphatase K	1.4906591	1.8977346	1.6166707
INPPL1	inositol polyphosphate phosphatase-like 1	-1.2308645	-1.6615534	-1.9736544
INSIG1	insulin induced gene 1	1.5459421	1.1980149	3.3455875
INSR	insulin receptor	-1.2952926	2.0447762	3.5742362
INTS4	integrator complex subunit 4	-1.4048474	-1.6166197	-1.73504
INTS6	integrator complex subunit 6	-1.6943685	-1.4741845	-1.5207783
IP6K3	inositol hexakisphosphate kinase 3	2.2144587	1.354813	1.2571096
IPO4	importin 4	-1.1727645	-1.3540674	-2.4226794
IPO5	importin 5	-1.2444669	-1.4945532	-2.0169096
IPO7	importin 7	1.0037488	-1.2139235	-1.7297585
IPO8	importin 8	1.3466642	1.5581433	1.5875939
IPW	imprinted in Prader-Willi syndrome (non-protein coding)	-1.2297539	-1.543522	-1.5780859
IPW	imprinted in Prader-Willi syndrome (non-protein coding)	1.4386709	-1.0102632	-1.5589285
IPW	imprinted in Prader-Willi syndrome (non-protein coding)	1.1170309	-1.2129246	-1.9568148
IQCJ-SCHIP1	IQCJ-SCHIP1 readthrough	1.3176943	-1.8550217	-2.1163278
IQCK	IQ motif containing K	-1.5442201	-1.3049895	-2.8686476
IQGAP1	IQ motif containing GTPase activating protein 1	-1.4434102	-1.537982	-3.0101817
IQGAP2	IQ motif containing GTPase activating protein 2	1.5041168	2.1264136	2.686905
IQGAP3	IQ motif containing GTPase activating protein 3	1.0064955	-2.5897245	-5.6519775
IQSEC1	IQ motif and Sec7 domain 1	-1.0852213	1.2148802	1.630946
IQSEC2	IQ motif and Sec7 domain 2	1.8769075	1.9881139	1.9622207
IQSEC2	IQ motif and Sec7 domain 2	-1.0013449	-1.2881837	-2.8822062
IQSEC3	IQ motif and Sec7 domain 3	2.473369	2.2737997	2.618795
IRAK1	interleukin-1 receptor-associated kinase 1	-1.2731541	-1.6764054	-2.1608407
IRAK1	interleukin-1 receptor-associated kinase 1	-1.5385873	-1.6894293	-2.2822316
IRF1	interferon regulatory factor 1	-3.6634185	-4.1095133	-4.086177
IRF3	interferon regulatory factor 3	-1.7903467	-1.9656699	-1.889008
IRF3	interferon regulatory factor 3	-1.8994788	-1.7435583	-1.9979151
IRF5	interferon regulatory factor 5	-1.5672343	-1.0578836	1.0202157
IRF7	interferon regulatory factor 7	-5.9755025	-1.3769797	-1.304234
IRF8	interferon regulatory factor 8	-1.0910943	1.1099738	-2.559174
ISCA2	iron-sulfur cluster assembly 2	1.843963	2.1674738	2.2583349
ISCU	iron-sulfur cluster assembly enzyme	1.3080854	1.5567272	1.4825939
ISG20	interferon stimulated exonuclease gene 20kDa	-2.220751	1.6716974	1.3249507
ISOC1	isochorismatase domain containing 1	-1.1531072	1.3028485	2.20995
ITCH	itchy E3 ubiquitin protein ligase	-1.0113542	2.0041192	2.8496697
ITFG3	integrin alpha FG-GAP repeat containing 3	1.5414295	1.2721169	1.5633758
ITGA1	integrin, alpha 1	-1.7330298	-1.0198003	1.4978752
ITGA10	integrin, alpha 10	4.0885067	5.2886734	4.465163
ITGA11	integrin, alpha 11	-1.0946367	-4.0395117	-4.7035713
ITGA3	integrin, alpha 3 (antigen CD49C, alpha 3 subunit of VLA-3 receptor)	-1.5452709	-4.0272655	-6.443793
ITGA5	integrin, alpha 5 (fibronectin receptor, alpha polypeptide)	-1.3772403	-2.6283114	-3.854451
ITGA6	integrin, alpha 6	-1.6139237	-1.693737	1.1217495
ITGA7	integrin, alpha 7	-1.2862264	1.8433341	3.656569
ITGAV	integrin, alpha V	-1.7023851	-3.209328	-3.9989333
ITGB1	integrin, beta 1 (fibronectin receptor, beta polypeptide, antigen CD29 includes MDF2, MSK12	-1.442199	-4.1351204	-5.2010007
ITGB3BP	integrin beta 3 binding protein (beta3-endonexin)	-1.1893289	-1.6648842	-1.7598375
ITGB4	integrin, beta 4	2.0106697	-1.8246834	-5.9146643
ITIH1	inter-alpha-trypsin inhibitor heavy chain 1	-9.417253	4.9496903	4.9411945
ITIH2	inter-alpha-trypsin inhibitor heavy chain 2	-10.977715	1.4087611	-1.1472391
ITIH4	inter-alpha-trypsin inhibitor heavy chain family, member 4	-6.037774	3.607724	4.6710906
ITPKA	inositol-trisphosphate 3-kinase A	-1.2343559	1.8336067	1.2127473
ITPKC	inositol-trisphosphate 3-kinase C	1.4425704	1.5764306	1.2226146
ITPR3	inositol 1,4,5-trisphosphate receptor, type 3	-2.7192774	-3.065577	-16.350079
ITPRIP	inositol 1,4,5-trisphosphate receptor interacting protein	-2.3820138	-3.6366994	-4.106964
IVD	isovaleryl-CoA dehydrogenase	1.452185	2.5422342	4.409746

IYD	iodotyrosine deiodinase	-1.7859085	6.1249433	8.020478
JADE1	jade family PHD finger 1	1.9524914	2.031855	1.2771516
JAG1	jagged 1	-2.5750299	-3.2540424	-8.715187
JAGN1	jagunal homolog 1 (Drosophila)	1.4095138	1.696549	1.9293422
JAK1	Janus kinase 1	-1.6800454	-1.5048122	-1.8496984
JARID2	jumonji, AT rich interactive domain 2	1.7620183	2.0236423	2.3727899
JDP2	Jun dimerization protein 2	2.8986254	1.8154786	2.3327706
JMJD4	jumonji domain containing 4	1.6668909	1.4990102	1.9121681
JMJD6	jumonji domain containing 6	1.4434763	1.0652043	-2.0013344
JMJD7-PLA2G4B	JMJD7-PLA2G4B readthrough	-1.7467358	-1.2189883	1.6505145
JMY	junction mediating and regulatory protein, p53 cofactor	-1.1698916	1.4534403	1.8471063
JOSD1	Josephin domain containing 1	-1.3467515	-1.7034897	-1.5982258
JPH1	junctophilin 1	1.2893693	-1.3523966	-1.506315
JPH2	junctophilin 2	4.1629624	3.2631578	1.3398132
JUN	jun proto-oncogene	-2.8931115	-2.1365488	-1.1836987
JUND	jun D proto-oncogene	-4.4574986	-3.8081355	-1.79964
JUP	junction plakoglobin	-4.0759306	-1.5427983	1.014704
KANK1	KN motif and ankyrin repeat domains 1	-1.8103138	1.2551012	1.2353132
KANK1	KN motif and ankyrin repeat domains 1	-1.1423529	1.9236462	2.0139766
KANK3	KN motif and ankyrin repeat domains 3	1.4131392	1.1776026	-1.9812729
KANK3	KN motif and ankyrin repeat domains 3	2.9894083	3.689026	4.0019903
KANK3	KN motif and ankyrin repeat domains 3	2.560227	2.734718	2.9728932
KANSL1-AS1	KANSL1 antisense RNA 1	1.0688338	1.4796829	1.5128949
KANSL1-AS1	KANSL1 antisense RNA 1	-1.0427121	1.476163	1.6449361
KAT2A	K(lysine) acetyltransferase 2A	-1.8927324	-1.8387265	-2.2208524
KAT2B	K(lysine) acetyltransferase 2B	-1.0323454	1.2840477	2.5733502
KAT8	K(lysine) acetyltransferase 8	1.1431708	1.476966	1.6183512
KBTBD13	kelch repeat and BTB (POZ) domain containing 13	1.248388	2.7470753	19.393818
KBTBD6	kelch repeat and BTB (POZ) domain containing 6	1.9326619	1.7666689	1.6730645
KCNB1	potassium channel, voltage gated Shab related subfamily B, member 1	8.045951	6.8438873	10.595238
KCND3	potassium channel, voltage gated Shal related subfamily D, member 3	1.5275539	1.8769838	2.2014594
KCNH3	potassium channel, voltage gated eag related subfamily H, member 3	2.9442534	3.167309	4.1577015
KCNJ16	potassium channel, inwardly rectifying subfamily J, member 16	1.6561313	4.5254326	2.6988358
KCNJ8	potassium channel, inwardly rectifying subfamily J, member 8	2.1608667	6.398984	4.098216
KCNK1	potassium channel, two pore domain subfamily K, member 1	1.1946107	-1.1724116	-1.935756
KCNK15	potassium channel, two pore domain subfamily K, member 15	1.7297579	1.8849751	2.3897536
KCNK5	potassium channel, two pore domain subfamily K, member 5	1.0436898	3.6543853	12.687871
KCNMB1	potassium channel subfamily M regulatory beta subunit 1	2.126264	2.0190334	2.3865051
KCNMB4	potassium channel subfamily M regulatory beta subunit 4	2.7770197	2.72645	2.8816698
KCP	kielin/chordin-like protein	1.5747533	1.6288555	2.086367
KCTD1	potassium channel tetramerization domain containing 1	1.1468084	1.1220427	-1.5698861
KCTD21	potassium channel tetramerization domain containing 21	1.7382553	2.239376	4.2969756
KCTD3	potassium channel tetramerization domain containing 3	1.0829264	1.3181365	2.113519
KCTD6	potassium channel tetramerization domain containing 6	-1.3725294	-1.0360585	1.8025761
KCTD9	potassium channel tetramerization domain containing 9	-1.6770604	-2.0079947	-2.8413627
KCTD9	potassium channel tetramerization domain containing 9	-1.5817267	-1.9218729	-2.5987477
KDELC2	KDEL (Lys-Asp-Glu-Leu) containing 2	1.2455384	-3.2600713	-4.695595
KDELR3	KDEL (Lys-Asp-Glu-Leu) endoplasmic reticulum protein retention receptor 3	-1.1157861	-1.5226551	-3.3693357
KDM2A	lysine (K)-specific demethylase 2A	-2.1561942	-2.156381	-2.1431131
KDM4A	lysine (K)-specific demethylase 4A	1.5520986	1.3181729	2.0284064
KDM4B	lysine (K)-specific demethylase 4B	1.5072048	1.6411873	2.0450006
KDM8	lysine (K)-specific demethylase 8	1.963651	1.9806063	2.1209314
KDR	kinase insert domain receptor	9.270897	10.134223	11.560132
KDSR	3-ketodihydrosphingosine reductase	1.317086	1.6054647	1.1358029
KHDRBS1	KH domain containing, RNA binding, signal transduction associated 1	1.5292505	1.0140878	-1.0351171
KHDRBS3	KH domain containing, RNA binding, signal transduction associated 3	-1.1129906	1.5773408	1.5195664
KHK	ketohexokinase (fructokinase)	-1.2721486	8.2269125	13.658095
KHNYN	KH and NYN domain containing	-1.674801	-1.5334387	1.296809
KIAA0020	KIAA0020	1.2699817	-1.0922318	-1.8421185
KIAA0040	KIAA0040	2.941536	2.8646564	1.867034
KIAA0101	KIAA0101	2.6816392	-2.7800198	-4.1307874
KIAA0319L	KIAA0319-like	-1.5125209	-1.2907219	-1.1387882
KIAA0368	KIAA0368	-1.9040349	-1.5278769	-1.8935673
KIAA0586	KIAA0586	-1.1769754	-1.6603031	-1.5053335
KIAA0753	KIAA0753	-1.0601864	-1.1117723	-1.6034397
KIAA0895L	KIAA0895-like	-1.58923	-1.9358932	-1.609626
KIAA0930	KIAA0930	-1.7415	-2.1599426	-1.903171
KIAA1143	KIAA1143	-1.5137879	-1.6298269	-1.6801437
KIAA1147	KIAA1147	-1.4830885	-1.4522524	1.75604
KIAA1147	KIAA1147	-1.939797	-1.9469713	1.3565557
KIAA1161	KIAA1161	-1.4205142	1.2656467	2.2965503
KIAA1191	KIAA1191	1.2274723	1.5510772	1.8307779
KIAA1217	KIAA1217	-1.0513399	-1.3869635	-1.7128581
KIAA1462	KIAA1462	3.2472882	1.3824762	-1.4586916
KIAA1462	KIAA1462	2.7520964	1.1990564	-2.1324294
KIAA1549	KIAA1549	-1.6994932	-1.6607854	-2.650815
KIAA1551	KIAA1551	-1.7476954	-1.1902733	1.5357862
KIAA1598	KIAA1598	-1.2923136	1.2240212	1.5044557
KIAA1671	KIAA1671	-2.8397074	-2.277891	-2.2653942
KIAA1671	KIAA1671	-2.002435	-1.5318646	-1.5203466
KIAA1683	KIAA1683	-2.0007882	2.1749542	3.1110032
KIAA1875	KIAA1875	2.2161949	2.5587478	2.6351442
KIAA1919	KIAA1919	-1.033866	1.250556	2.4082832
KIAA2013	KIAA2013	1.0478334	1.1367601	1.6331402
KIAA2018	KIAA2018	-1.0938368	1.4292654	1.8968298
KIF11	kinesin family member 11	1.9281951	-3.0961924	-3.2374039
KIF13B	kinesin family member 13B	-1.4552336	1.731212	1.6589267
KIF1B	kinesin family member 1B	-1.7481126	-1.0318091	1.5882754
KIF22	kinesin family member 22	-1.0755152	-3.6211674	-5.6944747
KIF22	kinesin family member 22	1.1296326	-3.543573	-5.36406
KIF23	kinesin family member 23	1.7658224	-3.5151982	-4.437385
KIF26B	kinesin family member 26B	1.2937592	1.6519475	2.1967878
KIF2A	kinesin heavy chain member 2A	-1.4901439	-2.4177263	-3.3631299
KIF2C	kinesin family member 2C	2.1487186	-3.719662	-3.5489209
KIF5B	kinesin family member 5B	-1.0125698	-1.6053182	-1.4514933
KIFC2	kinesin family member C2	-2.1208253	1.0781478	1.4364069
KIFC3	kinesin family member C3	-1.6633369	-1.9759538	-2.7806954
KIRREL	kin of IRRE like (Drosophila)	-1.1729779	-2.7196176	-5.85736
KITLG	KIT ligand	1.3084044	-1.0957412	-2.462714
KLC1	kinesin light chain 1	1.1336268	-1.6595298	-1.8925335
KLC2	kinesin light chain 2	-1.0967904	-1.4994454	-1.5574203
KLF11	Kruppel-like factor 11	-1.794819	-2.541398	-2.838805
KLF12	Kruppel-like factor 12	-1.1049694	1.2414386	2.0989938
KLF13	Kruppel-like factor 13	1.4987339	2.3532917	3.4614897
KLF3	Kruppel-like factor 3 (basic)	-1.2754227	1.3066126	2.0376391
KLF5	Kruppel-like factor 5 (intestinal)	-1.0360866	-3.067753	-2.2694108
KLF9	Kruppel-like factor 9	2.6838164	4.09817	5.981994
KLHDC2	kelch domain containing 2	1.0517874	1.5899932	1.7268274
KLHDC9	kelch domain containing 9	1.0401157	3.476872	5.565304

KLHL17	kelch-like family member 17	-1.5065672	-1.9461414	-1.891284
KLHL29	kelch-like family member 29	1.3924774	1.3413192	1.6371617
KLHL29	kelch-like family member 29	-1.1394027	-1.5095567	-1.9575288
KLHL5	kelch-like family member 5	-2.2834997	-3.1807504	-5.142399
KLHL7	kelch-like family member 7	1.0322104	-1.9513849	-1.059561
KLHL7	kelch-like family member 7	1.1429691	-1.9176323	1.0413016
KLHL9	kelch-like family member 9	2.0932496	2.032873	2.0810468
KLKB1	kallikrein B, plasma (Fletcher factor) 1	-4.0973554	2.7007983	4.886486
KLLN	killin, p53-regulated DNA replication inhibitor	1.3913026	1.8338082	-1.0027275
KLRG2	killer cell lectin-like receptor subfamily G, member 2	5.08836	4.5798407	6.032132
KMT2A	lysine (K)-specific methyltransferase 2A	-3.1538022	-2.3932297	-2.9894407
KMT2E	lysine (K)-specific methyltransferase 2E	-1.4383769	-1.5645124	-1.7065934
KMT2E-AS1	KMT2E antisense RNA 1 (head to head)	2.1310673	1.942207	2.0416217
KNG1	kininogen 1	-5.488182	28.761538	73.54354
KNSTRN	kinetochore-localized astrin/SPAG5 binding protein	2.0377333	-1.0670496	1.2820259
KPNA2	karyopherin alpha 2 (RAG cohort 1, importin alpha 1)	1.6675353	-1.9119855	-2.0660372
KPNA3	karyopherin alpha 3 (importin alpha 4)	1.6942422	1.6841327	1.6390926
KPNA4	karyopherin alpha 4 (importin alpha 3)	-1.3668277	-1.0972999	-1.5803552
KPNB1	karyopherin (importin) beta 1	1.5332439	1.1130941	-1.1069412
KRBOX4	KRAB box domain containing 4	-1.6238291	-1.5474821	-1.0799308
KRT19	keratin 19, type I	1.122049	-1.4632027	-1.9548874
KRT19P2	keratin 19 pseudogene 2	1.208689	-1.4742823	-2.1888678
KRT222	keratin 222, type II	1.1361321	2.3214278	4.2660637
KRT4	keratin 4, type II	7.022699	2.638006	-2.1118395
KRT5	keratin 5, type II	67.6534	32.72813	3.7201843
KRT6A	keratin 6A, type II	36.08951	27.525015	-1.3230515
KRT7	keratin 7, type II	1.86952	2.2853463	2.7181013
KRT73	keratin 73, type II	2.6469116	2.3969028	2.4333446
KRT8	keratin 8, type II	-1.1369929	-1.9126533	-2.8142407
KRT81	keratin 81, type II	1.4498204	1.7825041	2.15661
KRT83	keratin 83, type II	-1.0629427	-1.9484513	-2.7796376
KRT8P12	keratin 8 pseudogene 12	-1.8817556	-3.4406583	-4.547759
KRTAP10-10	keratin associated protein 10-10	2.1022825	1.8612672	1.9449816
KRTAP1-3	keratin associated protein 1-3	2.1013334	1.6850383	1.7577235
KRTAP1-4	keratin associated protein 1-4	2.0251122	1.6047465	1.620643
KRTAP2-1	keratin associated protein 2-1	2.0954177	2.1132622	2.643098
KRTAP2-4	keratin associated protein 2-4	1.4753233	1.3995004	1.9029117
KRTAP3-3	keratin associated protein 3-3	1.7502292	2.843596	3.196352
KRTAP5-11	keratin associated protein 5-11	1.1875658	1.4305531	1.5796534
KRTAP5-2	keratin associated protein 5-2	2.8882816	2.4340837	2.9203076
KRTAP5-4	keratin associated protein 5-4	2.097795	1.6692381	1.7072457
KRTAP9-1	keratin associated protein 9-1	1.9288919	2.0901713	1.9902419
KRTAP9-6	keratin associated protein 9-6	2.0150282	2.0394962	2.24451
KYNU	kynureninase	-6.853303	-6.9757004	-9.279091
L2HGDH	L-2-hydroxyglutarate dehydrogenase	-3.7599227	-2.5033162	-1.8181074
L3HYPDH	L-3-hydroxyproline dehydratase (trans-)	1.269489	-1.1265658	-1.6379694
LACTB2	lactamase, beta 2	1.8694433	1.6987666	1.3582957
LAD1	ladinin 1	1.1831919	-1.3096215	-7.4999866
LAMA1	laminin, alpha 1	-1.5842782	-2.1319554	-7.4502363
LAMA3	laminin, alpha 3	-2.4280279	-1.8783822	1.5704122
LAMA3	laminin, alpha 3	-1.2275213	1.5763257	11.4110155
LAMA5	laminin, alpha 5	-2.0341923	-2.7297513	-4.110849
LAMA5	laminin, alpha 5	-2.9236915	-2.6718497	-2.3155663
LAMB1	laminin, beta 1	2.1631327	1.9275838	2.4827027
LAMB1	laminin, beta 1	-1.7607043	-1.3942881	-2.7089498
LAMB3	laminin, beta 3	-3.2457774	-4.76128	-3.8713508
LAMC1	laminin, gamma 1 (formerly LAMB2)	1.0937178	-1.3141247	-1.9924387
LAMP2	lysosomal-associated membrane protein 2	-1.591592	1.4133383	1.7227303
LAMTOR5	late endosomal/lysosomal adaptor, MAPK and MTOR activator 5	1.7801306	1.739636	1.6384897
LAP3	leucine aminopeptidase 3	1.3462499	1.4457266	1.9132674
LAPTM4A	lysosomal protein transmembrane 4 alpha	1.3756601	1.440498	1.8767289
LARGE	like-glycosyltransferase	-1.3336267	-2.0313284	1.3483351
LARP1B	La ribonucleoprotein domain family, member 1B	1.2236738	2.0170352	2.7122657
LARP1B	La ribonucleoprotein domain family, member 1B	-1.5142549	1.1988902	1.5636367
LARP4	La ribonucleoprotein domain family, member 4	-1.4219732	-1.4869435	-1.6645409
LARP6	La ribonucleoprotein domain family, member 6	1.0686146	-2.6112862	-8.835104
LARS	leucyl-tRNA synthetase	-1.7276461	-1.8063581	-2.2193036
LASP1	LIM and SH3 protein 1	-1.7337658	-2.5666912	-2.832967
LAT	linker for activation of T cells	-2.1571147	-2.0247033	-1.0267439
LBHD1	LBH domain containing 1	1.2619265	1.4066675	1.5680778
LBX2	ladybird homeobox 2	1.8011224	2.061165	1.6651499
LBX2-AS1	LBX2 antisense RNA 1	1.0684257	1.4121236	2.3794196
LCE1A	late cornified envelope 1A	2.3560455	1.2078944	1.1420734
LCE1C	late cornified envelope 1C	2.1244702	1.6017952	1.7092195
LCE1D	late cornified envelope 1D	1.5119632	2.0243332	2.1435628
LCE1E	late cornified envelope 1E	1.454008	1.5700754	1.4637309
LCN2	lipocalin 2	-10.254714	-1.6656457	-3.9502237
LDHB	lactate dehydrogenase B	-2.058645	1.0726057	-1.7419744
LDHC	lactate dehydrogenase C	3.5262487	2.6611364	2.3829257
LDHD	lactate dehydrogenase D	1.5683019	6.6984844	7.898194
LEAP2	liver expressed antimicrobial peptide 2	-2.3792348	18.819801	65.31618
LEF1	lymphoid enhancer-binding factor 1	1.2180752	-1.8670751	-4.2709246
LEMD2	LEM domain containing 2	-1.3820329	-1.7832098	-1.7253371
LEPR	leptin receptor	-2.2628047	-1.640072	-1.7337017
LEPROTL1	leptin receptor overlapping transcript-like 1	-3.8899865	-2.6022015	-2.5112638
LETM1	leucine zipper-EF-hand containing transmembrane protein 1	1.0966864	-1.2356186	-1.58914
LETM1	leucine zipper-EF-hand containing transmembrane protein 1	2.9068923	3.2131388	3.254943
LETMD1	LETM1 domain containing 1	1.0908544	1.8426986	1.4756036
LFNG	LFNG O-fucosylpeptide 3-beta-N-acetylglucosaminyltransferase	2.1143386	2.4942746	2.6614044
LGALS1	lectin, galactoside-binding, soluble, 1	2.2993991	-2.369229	-5.96262
LGALS3	lectin, galactoside-binding, soluble, 3	5.4152913	6.154735	8.202203
LGALS4	lectin, galactoside-binding, soluble, 4	-3.4176745	3.3556151	1.9311547
LGALS8	lectin, galactoside-binding, soluble, 8	1.3886018	2.02317	-1.4002886
LGALSL	lectin, galactoside-binding-like	1.0338324	1.7098902	1.9979635
LGR4	leucine-rich repeat containing G protein-coupled receptor 4	-1.2770927	1.0189699	1.9554024
LGR6	leucine-rich repeat containing G protein-coupled receptor 6	3.1629133	2.0729773	1.961447
LHB	luteinizing hormone beta polypeptide	-1.2870706	-2.6993747	-2.8902943
LHFP	lipoma HMGIC fusion partner	1.5417712	-1.5802636	-1.5972545
LHFPL2	lipoma HMGIC fusion partner-like 2	-2.189289	-3.0655167	-3.8235662
LHPP	phospholysine phosphohistidine inorganic pyrophosphate phosphatase	1.2738047	1.5722522	5.241213
LHPP	phospholysine phosphohistidine inorganic pyrophosphate phosphatase	1.6689981	-1.0974874	1.0218961
LHX2	LIM homeobox 2	1.4838437	-2.0938056	-3.1804953
LIF	leukemia inhibitory factor	-2.6203063	-7.445741	-26.127714
LIG1	ligase I, DNA, ATP-dependent	-1.1953005	-3.1194096	-4.108791
LIMD1	LIM domains containing 1	1.1156889	1.6961247	2.1665504
LIMK1	LIM domain kinase 1	-1.5471245	-2.5900958	-4.5341845
LIMK2	LIM domain kinase 2	1.1152672	-1.8477709	-4.6602936
LIMS1	LIM and senescent cell antigen-like domains 1	-1.1197726	-1.7396116	-2.4390888
LIMS2	LIM and senescent cell antigen-like domains 2	1.9830931	1.7468754	-2.4487348

LIN28B	lin-28 homolog B (C. elegans)	1.6276137	-1.4887298	1.1515126
LIN7B	lin-7 homolog B (C. elegans)	1.2295763	1.5817947	2.3864732
LIN9	lin-9 DREAM MuvB core complex component	1.4867368	-1.5759506	-2.0758247
LINC00087	long intergenic non-protein coding RNA 87	1.8358889	3.5141115	9.660307
LINC00094	long intergenic non-protein coding RNA 94	1.3956097	1.3956907	1.7975482
LINC00106	long intergenic non-protein coding RNA 106	1.3432354	1.9575759	3.2823012
LINC00152	long intergenic non-protein coding RNA 152	1.1277398	-1.5188801	-3.6742425
LINC00176	long intergenic non-protein coding RNA 176	2.6444335	2.6468482	3.0502303
LINC00211	long intergenic non-protein coding RNA 211	1.4365958	1.8402637	2.1793566
LINC00261	long intergenic non-protein coding RNA 261	-2.5617743	1.4609818	5.9047017
LINC00265	long intergenic non-protein coding RNA 265	-1.5717386	-1.4011685	-1.3138707
LINC00265	long intergenic non-protein coding RNA 265	1.7762877	2.2536788	2.56195
LINC00322	long intergenic non-protein coding RNA 322	1.8296632	1.8250998	1.712117
LINC00339	long intergenic non-protein coding RNA 339	1.084453	1.6058747	2.106657
LINC00460	long intergenic non-protein coding RNA 460	2.0824494	1.3653364	1.3543642
LINC00467	long intergenic non-protein coding RNA 467	1.0224121	1.3802373	1.7126784
LINC00467	long intergenic non-protein coding RNA 467	-1.0695918	1.2504109	1.6171314
LINC00504	long intergenic non-protein coding RNA 504	-2.373454	-2.0523458	-1.9092262
LINC00504	long intergenic non-protein coding RNA 504	-3.114404	-2.5456614	-1.8188152
LINC00523	long intergenic non-protein coding RNA 523	1.8432004	1.590525	1.6960776
LINC00526	long intergenic non-protein coding RNA 526	-1.2165797	2.5577283	3.3277268
LINC00626	long intergenic non-protein coding RNA 626	-2.2870893	3.1058729	4.6090174
LINC00630	long intergenic non-protein coding RNA 630	2.5561426	2.55603	5.9288425
LINC00639	long intergenic non-protein coding RNA 639	1.4292687	1.9202636	2.2794633
LINC00657	long intergenic non-protein coding RNA 657	1.5827999	1.8263192	1.5011181
LINC00659	long intergenic non-protein coding RNA 659	-1.8451678	2.2068336	-1.8072726
LINC00659	long intergenic non-protein coding RNA 659	-1.454604	2.132892	-1.577448
LINC00659	long intergenic non-protein coding RNA 659	-1.5647229	2.707637	-1.3695751
LINC00659	long intergenic non-protein coding RNA 659	-1.4629114	2.96601	-1.261186
LINC00667	long intergenic non-protein coding RNA 667	1.2373215	1.6278821	2.182812
LINC00673	long intergenic non-protein coding RNA 673	1.1429613	-2.0640502	-4.472977
LINC00839	long intergenic non-protein coding RNA 839	1.3357631	-1.2621101	-2.3898175
LINC00847	long intergenic non-protein coding RNA 847	1.5143172	2.421563	1.4606383
LINC00861	long intergenic non-protein coding RNA 861	2.0205233	2.0598497	2.2402356
LINC00869	long intergenic non-protein coding RNA 869	-2.1042778	1.2853627	2.2917974
LINC00869	long intergenic non-protein coding RNA 869	-1.0944024	1.8482486	2.7455647
LINC00869	long intergenic non-protein coding RNA 869	1.0944088	2.2022426	3.2601864
LINC00869	long intergenic non-protein coding RNA 869	-1.8180283	1.3279365	2.4625437
LINC00877	long intergenic non-protein coding RNA 877	1.3796852	1.5553025	2.157759
LINC00883	long intergenic non-protein coding RNA 883	1.456133	-1.4050285	-3.1493254
LINC00884	long intergenic non-protein coding RNA 884	5.08336	6.8852158	7.167148
LINC00887	long intergenic non-protein coding RNA 887	2.5037367	2.5435164	3.4458249
LINC00887	long intergenic non-protein coding RNA 887	2.2008698	2.162362	2.9290712
LINC00888	long intergenic non-protein coding RNA 888	1.8204663	2.584947	1.8902476
LINC00888	long intergenic non-protein coding RNA 888	2.4969287	2.938806	3.661494
LINC00937	long intergenic non-protein coding RNA 937	1.9041035	1.8848323	2.232972
LINC00941	long intergenic non-protein coding RNA 941	2.5121508	2.4971845	2.7311604
LINC00963	long intergenic non-protein coding RNA 963	1.7082543	1.7426317	2.0182002
LINC00963	long intergenic non-protein coding RNA 963	2.4778016	2.8013513	2.8879936
LINC00963	long intergenic non-protein coding RNA 963	1.4780968	1.3202883	1.6824315
LINC00963	long intergenic non-protein coding RNA 963	1.619299	1.5610008	1.7353754
LINC00998	long intergenic non-protein coding RNA 998	-1.6377226	-1.9937937	-2.1537044
LINC00999	long intergenic non-protein coding RNA 999	3.4445775	3.8829293	4.700723
LINC01000	long intergenic non-protein coding RNA 1000	1.8099027	1.6855757	1.8366898
LINC01000	long intergenic non-protein coding RNA 1000	1.6491742	1.5431463	1.5760838
LINC01000	long intergenic non-protein coding RNA 1000	1.4432589	1.4264523	1.5345074
LINC01001	long intergenic non-protein coding RNA 1001	1.5313346	1.8483833	2.019263
LINC01001	long intergenic non-protein coding RNA 1001	1.500651	1.8575114	2.0952723
LINC01002	long intergenic non-protein coding RNA 1002	1.535599	1.8394014	1.4790763
LINC01002	long intergenic non-protein coding RNA 1002	1.5084383	1.5234615	1.8259407
LINC01003	long intergenic non-protein coding RNA 1003	1.3007776	1.5143228	1.6535972
LINC01006	long intergenic non-protein coding RNA 1006	1.1287344	3.1628263	4.0556664
LINC01021	long intergenic non-protein coding RNA 1021	-1.1880403	-1.2950901	-2.8225362
LINC01023	long intergenic non-protein coding RNA 1023	1.1281992	1.8747365	1.5295678
LINC01088	long intergenic non-protein coding RNA 1088	2.0379233	2.0788636	2.127344
LINC01091	long intergenic non-protein coding RNA 1091	-2.1936302	3.034462	1.6894507
LINC01091	long intergenic non-protein coding RNA 1091	-2.017682	3.2196465	1.7284107
LINC01104	long intergenic non-protein coding RNA 1104	1.574831	1.5755585	2.4141953
LINC01114	long intergenic non-protein coding RNA 1114	1.9639049	2.1697092	1.8974718
LINC01116	long intergenic non-protein coding RNA 1116	1.3548613	1.1174847	-3.4933074
LINC01121	long intergenic non-protein coding RNA 1121	3.1879408	2.8675418	2.6043155
LINC01122	long intergenic non-protein coding RNA 1122	1.9278743	2.15315	2.6767712
LINC01124	long intergenic non-protein coding RNA 1124	2.2899857	3.2774374	3.3169692
LINC01124	long intergenic non-protein coding RNA 1124	2.5195863	3.432369	3.6546218
LINC01133	long intergenic non-protein coding RNA 1133	2.8246183	-1.2684813	-1.1300246
LINC01137	long intergenic non-protein coding RNA 1137	-1.4671499	-1.4094077	-1.5667417
LINC01137	long intergenic non-protein coding RNA 1137	-1.3910044	-1.3729072	-1.5049903
LINC01158	long intergenic non-protein coding RNA 1158	2.9220529	3.218698	2.8088253
LINC01158	long intergenic non-protein coding RNA 1158	2.4125202	2.6821961	2.47613
LINC01237	long intergenic non-protein coding RNA 1237	1.4313816	1.5314436	1.3746601
LINC01268	long intergenic non-protein coding RNA 1268	-1.7543977	-1.4053786	-1.2726738
LINC01279	long intergenic non-protein coding RNA 1279	5.597194	-1.1337922	-2.923042
LINC01279	long intergenic non-protein coding RNA 1279	5.2949123	1.2965351	-2.4973958
LINC01298	long intergenic non-protein coding RNA 1298	2.1913028	2.2935302	2.845634
LINC01315	long intergenic non-protein coding RNA 1315	1.7897987	2.160795	-1.058271
LINC01405	long intergenic non-protein coding RNA 1405	3.8950121	3.655719	2.8330925
LINC01564	long intergenic non-protein coding RNA 1564	-7.73592	-3.6166174	1.1042403
LINC01564	long intergenic non-protein coding RNA 1564	1.021062	1.3763266	2.897249
LINC01564	long intergenic non-protein coding RNA 1564	-3.2849143	-1.2903161	2.860398
LINGO1	leucine rich repeat and Ig domain containing 1	1.319274	1.4372991	1.6085312
LINGO4	leucine rich repeat and Ig domain containing 4	1.6401306	1.9348882	2.4389434
LIPC	lipase, hepatic	-4.4438972	1.8214138	1.2687362
LIPT2	lipoyl(octanoyl) transferase 2 (putative)	1.3505551	1.1064976	2.3058617
LLGL2	lethal giant larvae homolog 2 (Drosophila)	-1.5290387	1.6485796	3.6194544
LLPH	LLP homolog, long-term synaptic facilitation (Aplysia)	1.6813602	1.1546224	1.1350807
LMAN2L	lectin, mannose-binding 2-like	-1.2882591	-1.6339602	-1.5592997
LMBRD1	LMBR1 domain containing 1	1.1078144	2.0671186	2.9352913
LMCD1	LIM and cysteine-rich domains 1	1.7266996	-2.8579764	-5.043061
LMF2	lipase maturation factor 2	-1.4398984	-1.503221	-1.4763316
LMNA	lamin A/C	1.0838557	-1.4600574	-2.6421816
LMNA	lamin A/C	1.0674236	-1.3587328	-2.6867924
LMNB2	lamin B2	1.6600245	-1.4455787	-3.041345
LMNTD2	lamin tail domain containing 2	-1.7039807	1.3921151	1.0907482
LMX1B	LIM homeobox transcription factor 1, beta	1.2647632	1.6022186	2.9791586
lnc-AKIRIN1-1	lnc-AKIRIN1-1:2	1.3748202	1.3970898	2.1310754
lnc-ANKRD11-5	lnc-ANKRD11-5:1	1.2108022	2.3267524	2.4003687
lnc-ANP32A-3	lnc-ANP32A-3:1	1.2159456	-2.859137	-4.9433384
lnc-BCKDHB-1	lnc-BCKDHB-1:2	2.1810267	2.0272713	2.0244844
lnc-BOLA2B-1	lnc-BOLA2B-1:1	1.7949905	2.8317187	3.7052288

lnc-CCDC71L-1	lnc-CCDC71L-1:3	2.014642	2.2358851	2.1189198
lnc-CCDC8-1	lnc-CCDC8-1:1	1.5888323	1.731984	2.1514704
lnc-CDH4-1	lnc-CDH4-1:1	2.3678186	2.934553	3.770321
lnc-CPM-1	lnc-CPM-1:1	2.3643806	1.832245	1.044563
lnc-CSAG1-1	lnc-CSAG1-1:2	1.1717913	7.0382605	3.017832
lnc-DAOA-4	lnc-DAOA-4:3	1.4785016	1.2511089	-5.1907225
lnc-DNAI1-1	lnc-DNAI1-1:1	1.6506194	1.5831035	2.0267289
lnc-EGLN1-1	lnc-EGLN1-1:1	1.1155324	1.3483603	2.2670014
lnc-EPHA1-1	lnc-EPHA1-1:1	-1.0325947	-1.2748294	-1.8951175
lnc-EYS-2	lnc-EYS-2:1	1.8235699	2.385948	1.5645881
lnc-FAM105B-1	lnc-FAM105B-1:1	2.1840768	1.9717522	1.9668887
lnc-FAM105B-1	lnc-FAM105B-1:1	2.278091	2.0090134	1.9519187
lnc-FARS2-2	lnc-FARS2-2:1	2.265481	-1.0168667	-1.1511393
lnc-GDF10-2	lnc-GDF10-2:1	2.354714	2.4231238	2.4212956
lnc-GGCT-1	lnc-GGCT-1:12	1.9775684	2.2600129	2.5876873
lnc-GLIPR1-3	lnc-GLIPR1-3:1	2.9391167	3.5001075	4.6484976
lnc-GOLGA8J-3	lnc-GOLGA8J-3:2	1.1913444	1.0551138	-3.1617408
lnc-LTBP3-2	lnc-LTBP3-2:3	9.741374	11.08231	12.727204
lnc-LTBP3-2	lnc-LTBP3-2:3	4.4677525	5.5791416	7.488633
lnc-LTBP3-2	lnc-LTBP3-2:4	1.4755962	1.6012262	3.1302106
lnc-MMRN1-2	lnc-MMRN1-2:5	-1.1677045	-1.2681409	-2.42884
lnc-MMRN1-2	lnc-MMRN1-2:1	-2.2622542	-2.035725	-3.7666912
lnc-MMRN1-2	lnc-MMRN1-2:2	-2.2798014	-2.0947802	-4.5558624
lnc-MOXD1-1	lnc-MOXD1-1:1	4.2949834	2.8692732	3.0921857
lnc-NAV1-3	lnc-NAV1-3:1	1.5811255	1.6687183	2.0972934
lnc-NDUFA4-2	lnc-NDUFA4-2:1	1.3214225	1.7523693	1.1528021
lnc-NGDN-1	lnc-NGDN-1:1	2.1378849	2.4110525	2.5948172
lnc-NOD1-1	lnc-NOD1-1:1	4.507947	3.145441	2.969168
lnc-PABPC4-2	lnc-PABPC4-2:4	4.0055723	3.7931676	4.6198597
lnc-PLEKHH2-1	lnc-PLEKHH2-1:1	1.0348175	1.7200288	2.7423127
lnc-PPA2-1	lnc-PPA2-1:1	1.945181	1.7685956	1.7231716
lnc-RAD23B-2	lnc-RAD23B-2:1	2.4921155	1.6507138	1.8639351
lnc-RP11-1105G2.3.1-3	lnc-RP11-1105G2.3.1-3:1	1.4775094	1.2869567	4.2916126
lnc-RP11-195B21.3.1-2	lnc-RP11-195B21.3.1-2:1	-1.4955158	-1.4082358	-2.5974271
lnc-RP11-410N8.4.1-2	lnc-RP11-410N8.4.1-2:1	2.097877	2.0524416	2.9636905
lnc-RP11-410N8.4.1-2	lnc-RP11-410N8.4.1-2:1	1.525816	-1.0023682	1.1327771
lnc-RP11-778D12.2.1-4	lnc-RP11-778D12.2.1-4:5	5.847357	4.192019	3.2048118
lnc-RP3-377D14.1.1-3	lnc-RP3-377D14.1.1-3:19	-1.0760087	-1.0865631	-2.0627453
lnc-RTL1-2	lnc-RTL1-2:1	4.422101	4.895727	6.0798426
lnc-SIK1-2	lnc-SIK1-2:1	2.4273107	6.026487	3.4873009
lnc-SIK1-2	lnc-SIK1-2:1	2.2892258	6.006354	3.3339152
lnc-SLC38A8-1	lnc-SLC38A8-1:1	1.7759404	1.920396	1.8490733
lnc-ST3GAL1-1	lnc-ST3GAL1-1:1	1.7152299	1.5369463	1.7378031
lnc-TARDBP-2	lnc-TARDBP-2:2	1.8421596	1.962307	2.7490528
lnc-TBC1D29-1	lnc-TBC1D29-1:1	2.066151	2.3994086	1.4326794
lnc-TMEM178-1	lnc-TMEM178-1:9	1.6198671	2.087056	2.7328496
lnc-TOP1MT-2	lnc-TOP1MT-2:1	1.6580813	2.4862134	2.6223977
lnc-TSHZ1-1	lnc-TSHZ1-1:1	2.6405299	1.5655923	1.6250676
lnc-USP35-1	lnc-USP35-1:6	1.5753679	-1.1241746	-1.5240116
lnc-UXS1-4	lnc-UXS1-4:1	1.6692259	1.6781123	4.7914085
LNX2	ligand of numb-protein X 2	1.4806508	2.0409982	1.8578224
LOC100128002	uncharacterized LOC100128002	3.1539512	2.4989545	2.951117
LOC100128320	uncharacterized LOC100128320	3.6053789	3.7569127	2.8868806
LOC100128364	uncharacterized LOC100128364	3.4204834	3.7412648	4.265022
LOC100128714	uncharacterized LOC100128714	2.854063	1.6472417	1.37655
LOC100128851	uncharacterized LOC100128851	1.7636156	-1.1733787	-1.0952171
LOC100129115	uncharacterized LOC100129115	1.7869904	2.0087888	2.5833285
LOC100129406	uncharacterized LOC100129406	1.9922059	1.895402	2.4485974
LOC100130027	uncharacterized LOC100130027	1.1737905	1.3946245	2.266191
LOC100130152	uncharacterized LOC100130152	4.31114	4.998816	5.9086843
LOC100130238	uncharacterized LOC100130238	2.6756814	2.4462442	2.8576944
LOC100130456	uncharacterized LOC100130456	2.4160635	2.142806	2.2008905
LOC100130654	uncharacterized LOC100130654	1.610473	-1.0596505	-1.216053
LOC100131262	uncharacterized LOC100131262	1.513374	1.0232161	-6.491584
LOC100131831	uncharacterized LOC100131831	1.1136055	1.7999102	1.9652478
LOC100132363	uncharacterized LOC100132363	1.8008946	2.1830134	2.58008
LOC100132874	uncharacterized LOC100132874	2.9140217	3.2104845	3.8382983
LOC100133182	uncharacterized LOC100133182	2.1685538	2.7093606	3.1357043
LOC100133286	uncharacterized LOC100133286	3.0066633	2.925964	3.672346
LOC100133985	uncharacterized LOC100133985	1.2662944	1.8511281	3.4614425
LOC100270746	uncharacterized LOC100270746	2.2588866	3.2026415	2.2065623
LOC100288911	uncharacterized LOC100288911	2.9239812	2.19811	1.7256851
LOC100289026	putative uncharacterized protein FLJ00310-like	-1.0345309	-1.2256788	-2.6307206
LOC100294145	uncharacterized LOC100294145	1.7554934	1.7884332	2.0308158
LOC100422737	uncharacterized LOC100422737	2.2873628	1.7810315	2.0173576
LOC100499194	uncharacterized LOC100499194	3.007044	3.2428157	3.791462
LOC100506253	uncharacterized LOC100506253	-1.1187485	3.6401548	2.6781137
LOC100506411	uncharacterized LOC100506411	-1.1535809	-1.5562476	-3.7134464
LOC100506688	uncharacterized LOC100506688	1.9090359	5.4480395	1.8361187
LOC100506860	uncharacterized LOC100506860	1.0404994	1.8580103	-1.3034194
LOC100506860	uncharacterized LOC100506860	1.1041905	2.1426682	-1.264733
LOC100507006	uncharacterized LOC100507006	3.0561411	3.2830224	3.9520645
LOC100507747	polycystic kidney disease protein 1-like 3-like	2.2065763	2.5407307	3.061916
LOC100996291	uncharacterized LOC100996291	1.9410106	1.9966756	2.5618443
LOC100996579	uncharacterized LOC100996579	1.6129094	1.5561212	1.7918985
LOC100996724	phosphodiesterase 4D interacting protein-like	-1.5239866	1.1799661	1.5416808
LOC101927100	uncharacterized LOC101927100	1.4319062	2.8774521	4.031444
LOC101927151	uncharacterized LOC101927151	1.9357252	1.9580691	5.6154184
LOC101927151	uncharacterized LOC101927151	1.0654937	1.2778605	-2.5516982
LOC101927151	uncharacterized LOC101927151	1.3278898	1.2286731	-2.3474686
LOC101927151	uncharacterized LOC101927151	1.3289647	1.6139504	-1.9598149
LOC101927686	uncharacterized LOC101927686	1.5835068	1.6529486	1.2488818
LOC101927764	uncharacterized LOC101927764	2.1475267	1.9672456	2.173949
LOC101927974	uncharacterized LOC101927974	1.350361	1.8672023	3.2011724
LOC101928076	uncharacterized LOC101928076	1.3045517	1.6414521	1.3141654
LOC101928673	uncharacterized LOC101928673	2.7562442	3.7817745	3.7495434
LOC101928738	uncharacterized LOC101928738	1.5887525	1.5962713	1.7057991
LOC101928738	uncharacterized LOC101928738	1.7929485	1.8325433	2.253477
LOC101928858	uncharacterized LOC101928858	3.9702656	18.362019	17.945904
LOC101928858	uncharacterized LOC101928858	3.9275172	17.966913	16.78614
LOC101928858	uncharacterized LOC101928858	3.3245876	20.899548	8.773948
LOC101928858	uncharacterized LOC101928858	5.528862	24.492332	22.80726
LOC101928858	uncharacterized LOC101928858	3.1312943	13.832436	13.385137
LOC101928858	uncharacterized LOC101928858	3.7049654	18.160194	9.707321
LOC101928858	uncharacterized LOC101928858	3.1932683	21.166702	8.360214
LOC101928958	uncharacterized LOC101928958	4.2845535	12.635518	-2.7626739
LOC101928991	coiled-coil domain-containing protein 92-like	1.5126866	4.3017116	8.761825
LOC101929243	uncharacterized LOC101929243	1.2775571	2.559603	2.4136467
LOC101929450	uncharacterized LOC101929450	1.0501862	10.358123	9.51818

LOC101929450	uncharacterized LOC101929450	-1.2043804	9.87508	7.550538
LOC101929452	uncharacterized LOC101929452	1.9687799	2.5528271	2.7246168
LOC101930375	uncharacterized LOC101930375	1.6290392	1.6262345	1.650029
LOC101930506	uncharacterized LOC101930506	1.7524709	2.47318	2.2440267
LOC102467146	uncharacterized LOC102467146	3.8831635	4.7657633	5.3807545
LOC102723908	uncharacterized LOC102723908	1.144063	1.6449926	1.4565359
LOC102724030	uncharacterized LOC102724030	1.0776749	2.4766371	2.1706278
LOC102724279	uncharacterized LOC102724279	1.6482065	1.7505134	2.0313241
LOC102724462	uncharacterized LOC102724462	-1.2580217	-1.5725486	-3.49077
LOC102724930	uncharacterized LOC102724930	2.346352	1.7657932	1.9101168
LOC102725053	unconventional myosin-Vb-like	1.4244559	1.4767814	1.7017243
LOC102725353	uncharacterized LOC102725353	-3.0917652	-2.675019	-2.5317943
LOC115110	uncharacterized LOC115110	-2.4034233	-1.2073665	-2.7361329
LOC145837	uncharacterized LOC145837	-3.2752068	3.6938665	10.15246
LOC148709	actin pseudogene	1.7315416	-1.1157655	-1.5109746
LOC149950	uncharacterized LOC149950	1.7392045	1.7974303	1.9347942
LOC151174	uncharacterized LOC151174	1.9323452	3.4292443	18.522205
LOC254896	uncharacterized LOC254896	1.3107237	1.9751302	2.3071566
LOC257152	uncharacterized LOC257152	-2.904765	-2.014069	-2.2311118
LOC283335	uncharacterized LOC283335	2.1040366	2.1683908	1.9923873
LOC283352	uncharacterized LOC283352	2.3587723	1.6169344	1.4337714
LOC283485	uncharacterized LOC283485	-1.1482391	-2.5492334	-12.79019
LOC283911	uncharacterized LOC283911	-1.075218	1.6472073	2.648927
LOC284379	solute carrier family 7 (cationic amino acid transporter, y+ system), member 3 pseudogene	1.9255514	2.0085742	1.9486661
LOC284454	uncharacterized LOC284454	1.9275072	1.9506773	1.8822062
LOC284581	uncharacterized LOC284581	-5.5579104	-4.087051	-3.3376193
LOC284930	uncharacterized LOC284930	1.9465097	1.6440758	1.6167992
LOC284933	uncharacterized LOC284933	2.036771	2.52413	3.0245905
LOC285095	uncharacterized LOC285095	2.404979	2.0676162	2.1844091
LOC285626	uncharacterized LOC285626	-2.136755	3.696497	267.74316
LOC286382	uncharacterized LOC286382	1.673759	1.9867283	2.5659895
LOC286437	uncharacterized LOC286437	-1.7926528	-1.170475	-1.8699709
LOC339803	uncharacterized LOC339803	1.570432	1.7690775	1.5204589
LOC388210	uncharacterized LOC388210	1.7864777	2.7009633	3.7783992
LOC388210	uncharacterized LOC388210	1.9902828	1.9492747	1.6496445
LOC388210	uncharacterized LOC388210	2.5690145	1.7453774	2.0291934
LOC388780	uncharacterized LOC388780	-1.1172265	1.2972506	-2.7650075
LOC389033	placenta-specific 9 pseudogene	1.7739303	1.9961438	2.7774773
LOC389834	ankyrin repeat domain 57 pseudogene	1.3539006	1.6994576	1.8805754
LOC389834	ankyrin repeat domain 57 pseudogene	-1.2189456	1.9534622	1.596889
LOC389906	zinc finger protein 839 pseudogene	1.4714352	-1.1236224	-2.4254763
LOC400043	uncharacterized LOC400043	-1.1914917	-1.4341508	-2.1249914
LOC400043	uncharacterized LOC400043	-2.1623282	-1.9881638	-2.506735
LOC400558	uncharacterized LOC400558	2.178705	2.5810468	3.0497618
LOC400863	uncharactered LOC400863	3.427062	2.360411	2.5138206
LOC401320	uncharacterized LOC401320	-3.0396228	-2.0852053	-1.755736
LOC401320	uncharacterized LOC401320	-3.0635607	-1.8962258	-1.697841
LOC440173	uncharacterized LOC440173	1.771946	1.3670756	1.5244437
LOC441081	POM121 membrane glycoprotein (rat) pseudogene	1.8873384	2.49717	4.079914
LOC441455	makorin ring finger protein 1 pseudogene	-1.4063299	-1.5983853	-1.4186925
LOC641746	glycine cleavage system protein H (aminomethyl carrier) pseudogene	1.3867129	1.5320661	1.829559
LOC642236	FSHD region gene 1 pseudogene	1.8301177	1.5797572	1.5662032
LOC642366	uncharacterized LOC642366	2.4117718	3.8938258	1.8714038
LOC642423	golgin A2 pseudogene	1.3844547	2.744692	1.9469258
LOC643454	adaptor-related protein complex 3, sigma 1 subunit pseudogene	1.0130672	-1.5340519	-1.0828872
LOC643549	uncharacterized LOC643549	-1.3020235	1.5843155	1.9488332
LOC644189	acyl-CoA thioesterase 4 pseudogene	1.1744932	1.5492439	2.6355302
LOC644277	uncharacterized LOC644277	1.9247792	2.5560074	3.6319668
LOC644277	uncharacterized LOC644277	1.9081577	2.5386348	3.750372
LOC644794	uncharacterized LOC644794	2.3814595	2.3766918	2.7176003
LOC645166	lymphocyte-specific protein 1 pseudogene	1.1234454	1.0887145	-2.9432364
LOC645261	PP565	-1.4268698	1.6613863	2.3005977
LOC645553	uncharacterized LOC645553	3.4810615	4.1728673	4.967021
LOC646214	p21 protein (Cdc42/Rac)-activated kinase 2 pseudogene	1.7846106	1.2361035	-1.0345799
LOC646743	uncharacterized LOC646743	1.5220679	2.2829905	2.9409425
LOC653712	intraflagellar transport 122 homolog (Chlamydomonas) pseudogene	1.5373483	2.2978961	5.1249213
LOC727751	golgin A2 pseudogene	-1.9344758	-2.1706274	-1.4079388
LOC728975	uncharacterized LOC728975	1.109053	2.3204083	1.3051189
LOC729080	glycine cleavage system protein H (aminomethyl carrier) pseudogene	1.5747429	1.7111658	1.9726548
LOC729680	uncharacterized LOC729680	1.9017067	1.0589381	1.3738438
LOC729887	uncharacterized LOC729887	1.2010212	1.8717154	1.3118021
LOC729970	hCG2028352-like	1.0954099	2.02782	3.1418185
LOC730102	quinone oxidoreductase-like protein 2 pseudogene	1.5626179	1.5401765	1.7916902
LOC730257	uncharacterized LOC730257	-1.5692366	1.0244552	-1.3649933
LOC93622	Morf4 family associated protein 1-like 1 pseudogene	1.1295754	1.2525803	1.7190315
LOC93622	Morf4 family associated protein 1-like 1 pseudogene	1.1643015	1.3882699	1.8288208
LONP2	lon peptidase 2, peroxisomal	1.1726811	1.8927737	2.4818094
LPCAT3	lysophosphatidylcholine acyltransferase 3	1.5545287	2.288464	3.2173328
LPGAT1	lysophosphatidylglycerol acyltransferase 1	-1.5626994	1.031993	1.1978898
LPHN2	latrophilin 2	-1.0629047	-1.4298736	-1.5420325
LPIN1	lipin 1	2.0999665	2.1690824	2.938834
LPIN2	lipin 2	-1.051371	2.4139223	2.6657324
LPL	lipoprotein lipase	1.3345457	1.885963	2.5774329
LPP	LIM domain containing preferred translocation partner in lipoma	-1.959714	-1.9695487	-2.488223
LRBA	LPS-responsive vesicle trafficking, beach and anchor containing	-1.8865582	-1.3301755	-1.3638698
LRG1	leucine-rich alpha-2-glycoprotein 1	-2.4100797	2.3563797	1.3870783
LRIG3	leucine-rich repeats and immunoglobulin-like domains 3	1.0201488	1.5212038	-1.1210712
LRP1	low density lipoprotein receptor-related protein 1	-4.4690676	-2.1971283	-2.3997223
LRP12	low density lipoprotein receptor-related protein 12	-2.2401192	-4.0795975	-3.7926369
LRP1B	low density lipoprotein receptor-related protein 1B	2.4384363	10.919251	2.5322604
LRP5	low density lipoprotein receptor-related protein 5	-2.060798	-1.8452685	-1.8369595
LRP8	low density lipoprotein receptor-related protein 8, apolipoprotein e receptor	1.3359364	-2.4823222	-3.3480837
LRR1	leucine rich repeat protein 1	1.9311243	-1.2984953	-1.6401863
LRRC1	leucine rich repeat containing 1	2.1852553	1.4294367	-1.1922619
LRRC14	leucine rich repeat containing 14	1.1796774	1.5922878	3.1619117
LRRC20	leucine rich repeat containing 20	1.6436237	1.4104222	1.3533016
LRRC26	leucine rich repeat containing 26	2.022269	1.9912239	1.9958276
LRRC37BP1	leucine rich repeat containing 37B pseudogene 1	-1.3143231	-1.820775	-1.4921579
LRRC57	leucine rich repeat containing 57	1.2176175	1.0485196	1.7763456
LRRC73	leucine rich repeat containing 73	1.0783551	2.3104894	1.5263138
LRRC75B	leucine rich repeat containing 75B	-2.5650532	-1.2905056	1.2970917
LRRC8A	leucine rich repeat containing 8 family, member A	-1.0388182	1.4841044	2.1976192
LRRC8B	leucine rich repeat containing 8 family, member B	-1.4338652	-1.59201	-1.5270827
LRRC8E	leucine rich repeat containing 8 family, member E	-1.7585802	-1.8465787	-2.145994
LRRFIP1	leucine rich repeat (in FLII) interacting protein 1	-1.3319929	-1.4199419	-2.3592331
LRRFIP1	leucine rich repeat (in FLII) interacting protein 1	-1.4021702	-1.8607883	-3.7245457
LRRN2	leucine rich repeat neuronal 2	1.5839041	1.2760093	1.4136406
LSM2	LSM2 homolog, U6 small nuclear RNA associated (S. cerevisiae)	1.0637438	-1.315995	-1.6441349
LSM2	LSM2 homolog, U6 small nuclear RNA associated (S. cerevisiae)	1.2089628	-1.276872	-1.563047

LSM6	LSM6 homolog, U6 small nuclear RNA associated (S. cerevisiae)	1.5047082	1.2541047	1.1416221
LSM7	LSM7 homolog, U6 small nuclear RNA associated (S. cerevisiae)	1.2689618	-1.375173	-1.5249145
LSM8	LSM8 homolog, U6 small nuclear RNA associated (S. cerevisiae)	1.6327205	1.0151867	-1.006637
LSP1	lymphocyte-specific protein 1	-1.3128734	-1.3389007	-2.684606
LSR	lipolysis stimulated lipoprotein receptor	-2.779328	-1.3689827	-1.4965411
LSS	lanosterol synthase (2,3-oxidosqualene-lanosterol cyclase)	1.6570983	2.4101021	4.6611633
LTB4R2	leukotriene B4 receptor 2	1.6470544	1.8504689	1.9212945
LTBP3	latent transforming growth factor beta binding protein 3	-3.9928014	-2.143677	-5.9275827
LTBP4	latent transforming growth factor beta binding protein 4	2.0256934	1.6286322	1.5132761
LUC7L	LUC7-like (S. cerevisiae)	-1.4376056	-1.6586342	-1.9246587
LUC7L2	LUC7-like 2 (S. cerevisiae)	1.1403474	1.1893152	1.5090126
LUM	lumican	2.2111351	-2.2311115	-17.86983
LUZP4	leucine zipper protein 4	1.6404765	1.0926704	-1.2842132
LY6D	lymphocyte antigen 6 complex, locus D	12.221703	35.42129	1.7613097
LY6E	lymphocyte antigen 6 complex, locus E	1.0632175	1.3266535	-1.9177887
LY6G6D	lymphocyte antigen 6 complex, locus G6D	5.8046813	5.8373637	7.534572
LY9	lymphocyte antigen 9	5.2367573	5.029	8.001431
LY96	lymphocyte antigen 96	-1.5848341	-1.5687544	-3.3067932
LYAR	Ly1 antibody reactive	1.3856522	-1.0991488	-1.7946863
LYPD1	LY6/PLAUR domain containing 1	-1.1517224	-4.056964	-3.6590424
LYPD1	LY6/PLAUR domain containing 1	-1.7047673	-6.556615	-5.758983
LYPLA2	lysophospholipase II	1.324109	1.5788542	1.5867
LYPLAL1	lysophospholipase-like 1	-1.116659	1.5753143	2.1667144
LYRM9	LYR motif containing 9	1.8442228	3.6546695	6.69413
LYSMD1	LysM, putative peptidoglycan-binding, domain containing 1	1.6155651	1.5712045	-1.1630958
LYSMD4	LysM, putative peptidoglycan-binding, domain containing 4	-1.1983845	-1.3315594	-1.7209779
LZTS1	leucine zipper, putative tumor suppressor 1	1.8410313	-3.2117453	-13.445378
LZTS2	leucine zipper, putative tumor suppressor 2	-1.0721858	-1.1818815	-2.7414136
LZTS2	leucine zipper, putative tumor suppressor 2	2.667816	2.8616483	2.98716
MAB21L2	mab-21-like 2 (C. elegans)	2.413542	2.7973707	2.7012758
MAD2L1	MAD2 mitotic arrest deficient-like 1 (yeast)	1.9742887	-3.4069426	-2.8354328
MADD	MAP-kinase activating death domain	-1.7693003	-1.6960735	-1.852105
MAFA	v-maf avian musculoaponeurotic fibrosarcoma oncogene homolog A	1.7074193	1.690329	2.234294
MAFB	v-maf avian musculoaponeurotic fibrosarcoma oncogene homolog B	1.0177836	2.4475865	3.4654603
MAFB	v-maf avian musculoaponeurotic fibrosarcoma oncogene homolog B	-1.0856537	2.490549	3.6194744
MAFF	v-maf avian musculoaponeurotic fibrosarcoma oncogene homolog F	-14.923137	-16.578236	-24.400553
MAFG	v-maf avian musculoaponeurotic fibrosarcoma oncogene homolog G	-1.8151377	-2.8090262	-1.9670715
MAFIP	MAFF interacting protein (pseudogene)	2.4054198	2.6361048	3.3997962
MAFK	v-maf avian musculoaponeurotic fibrosarcoma oncogene homolog K	-3.370514	-3.4426472	-3.5171297
MAGEA1	melanoma antigen family A, 1	2.779297	2.1161897	1.4598731
MAGEA6	melanoma antigen family A, 6	1.6970701	1.6176912	1.4049202
MAGED4B	melanoma antigen family D, 4B	-1.8848338	-4.206301	-6.466676
MAGT1	magnesium transporter 1	1.1310745	1.4804931	1.9712845
MAL2	mal, T-cell differentiation protein 2 (gene/pseudogene)	-1.033784	1.5978875	1.4971247
MALAT1	metastasis associated lung adenocarcinoma transcript 1 (non-protein coding)	-2.5436835	-2.730466	-1.2919221
MAMLD1	mastermind-like domain containing 1	1.7552551	1.6176875	-1.0903256
MAN1A1	mannosidase, alpha, class 1A, member 1	-2.294048	-1.0029584	1.6634785
MAN1A2	mannosidase, alpha, class 1A, member 2	-1.5134839	-1.4276898	1.101575
MAN1B1	mannosidase, alpha, class 1B, member 1	-1.304995	-2.157047	-2.3133643
MAN2C1	mannosidase, alpha, class 2C, member 1	-2.119267	-1.5889059	-1.3294957
MANEA	mannosidase, endo-alpha	-1.1371686	1.098995	2.3689532
MANEAL	mannosidase, endo-alpha-like	1.104014	1.5874982	2.468245
MAOA	monoamine oxidase A	5.4497666	10.37893	9.598165
MAOB	monoamine oxidase B	-1.2773956	1.9268045	2.640185
MAP2	microtubule-associated protein 2	1.5987587	1.5512601	-1.3642496
MAP2K3	mitogen-activated protein kinase kinase 3	1.2047329	1.0479861	1.8778602
MAP2K4	mitogen-activated protein kinase kinase 4	1.3452551	1.2316853	1.586526
MAP3K13	mitogen-activated protein kinase kinase kinase 13	1.3096148	1.2536784	1.5511557
MAP3K13	mitogen-activated protein kinase kinase kinase 13	1.266341	1.2797709	1.5599185
MAP3K3	mitogen-activated protein kinase kinase kinase 3	1.287852	1.2224679	1.6675869
MAP3K4	mitogen-activated protein kinase kinase kinase 4	-3.0846531	-3.1436815	-2.762182
MAP3K5	mitogen-activated protein kinase kinase kinase 5	1.7472477	2.1459875	1.3952972
MAP3K6	mitogen-activated protein kinase kinase kinase 6	1.0339003	-1.1306568	-2.5463815
MAP3K9	mitogen-activated protein kinase kinase kinase 9	-1.2265176	-1.6715463	-2.4128523
MAP4	microtubule-associated protein 4	-1.7020211	-2.363265	-2.8502755
MAP4K5	mitogen-activated protein kinase kinase kinase kinase 5	-1.3592544	-1.7098284	-1.931725
MAP7	microtubule-associated protein 7	-2.909172	-1.1591151	1.7710531
MAP7D1	MAP7 domain containing 1	1.1454303	-1.2833234	-2.4404235
MAP7D1	MAP7 domain containing 1	1.1384991	-1.2172781	-2.031988
MAP7D2	MAP7 domain containing 2	2.6568503	1.5621566	-7.1616473
MAP7D3	MAP7 domain containing 3	1.9544646	1.1366768	-1.04242
MAPK7	mitogen-activated protein kinase 7	-1.1003795	-1.7484473	-1.9419562
MAPKAP1	mitogen-activated protein kinase associated protein 1	-1.430991	-1.6464916	-1.4361838
MAPKAPK3	mitogen-activated protein kinase-activated protein kinase 3	1.237494	-1.0036192	-2.093325
MAPKBP1	mitogen-activated protein kinase binding protein 1	-1.3867167	-1.6042306	-1.6002572
MAPRE1	microtubule-associated protein, RP/EB family, member 1	1.2431756	-1.5150113	-2.3925207
MAPRE1	microtubule-associated protein, RP/EB family, member 1	1.3913058	-1.1905609	-1.5401655
MAPRE2	microtubule-associated protein, RP/EB family, member 2	-1.6623458	-1.7521547	1.4706149
MAPT	microtubule-associated protein tau	2.0516288	2.627759	3.879623
MARC1	mitochondrial amidoxime reducing component 1	2.2119348	2.0382931	4.2233233
MARC2	mitochondrial amidoxime reducing component 2	1.0999925	2.7744339	3.3181794
MARC2	mitochondrial amidoxime reducing component 2	1.5811694	3.7486796	4.6620383
MARCH6	membrane-associated ring finger (C3HC4) 6, E3 ubiquitin protein ligase	-1.4052725	1.1146001	1.5911134
MARCKS	myristoylated alanine-rich protein kinase C substrate	-1.4204837	-2.5200026	-2.9628255
MARCKSL1	MARCKS-like 1	-1.4802505	-2.4815476	-3.7629364
MARS	methionyl-tRNA synthetase	-1.5078872	-1.9923747	-1.7456777
MARVELD2	MARVEL domain containing 2	-1.4684213	1.4263006	1.9582713
MARVELD2	MARVEL domain containing 2	-1.9735518	1.0760859	1.3210192
MAST2	microtubule associated serine/threonine kinase 2	-1.6164469	-2.4811628	-2.3338985
MAST4	microtubule associated serine/threonine kinase family member 4	-2.0496337	-2.1230707	-4.1037464
MAT1A	methionine adenosyltransferase I, alpha	-2.4698684	3.202051	15.049508
MATN1-AS1	MATN1 antisense RNA 1	3.1637368	3.0688388	3.6510031
MATR3	matrin 3	-1.2733145	-1.5847554	-1.52261
MAVS	mitochondrial antiviral signaling protein	1.4729551	1.5984225	1.6991887
MAVS	mitochondrial antiviral signaling protein	1.5826739	1.6924045	1.3107766
MAVS	mitochondrial antiviral signaling protein	1.7867292	1.7606593	1.7606282
MAVS	mitochondrial antiviral signaling protein	1.5233334	1.7432458	1.7001657
MBD1	methyl-CpG binding domain protein 1	-1.3564816	-1.4644332	-1.5157485
MBIP	MAP3K12 binding inhibitory protein 1	-1.0850399	1.588496	1.2132812
MBLAC1	metallo-beta-lactamase domain containing 1	1.9145595	1.9299997	2.094482
MBLAC2	metallo-beta-lactamase domain containing 2	2.3872616	2.463132	2.7669382
MBOAT7	membrane bound O-acyltransferase domain containing 7	-1.0456344	-1.3409972	-1.68771
MBP	myelin basic protein	1.1768451	1.6145706	2.1095805
MCCC1	methylcrotonoyl-CoA carboxylase 1 (alpha)	1.1208171	2.3485248	1.682538
MCCC2	methylcrotonoyl-CoA carboxylase 2 (beta)	-1.5548893	-1.0176347	1.3508348
MCEE	methylmalonyl CoA epimerase	-1.1111044	2.114452	2.500203
MCF2L-AS1	MCF2L antisense RNA 1	1.1502895	3.354192	1.548475
MCM2	minichromosome maintenance complex component 2	1.1303544	-3.4003432	-6.094077
MCM3	minichromosome maintenance complex component 3	1.2788198	-1.9554454	-3.1032248

MCM3AP	minichromosome maintenance complex component 3 associated protein	-1.6347944	-1.5724143	-1.4627019
MCM4	minichromosome maintenance complex component 4	1.3475741	-2.2313073	-3.7342138
MCM5	minichromosome maintenance complex component 5	1.1164188	-2.817243	-4.856501
MCM5	minichromosome maintenance complex component 5	1.0234474	-2.8362687	-5.44369
MCM7	minichromosome maintenance complex component 7	1.0676969	-2.4449148	-3.2105465
MCM8	minichromosome maintenance complex component 8	-1.0789616	-3.2667112	-4.1879163
MCRS1	microspherule protein 1	-1.361951	-1.5628675	-1.9774204
MCTP1	multiple C2 domains, transmembrane 1	2.2961981	2.940245	2.499165
MCUR1	mitochondrial calcium uniporter regulator 1	1.3753089	1.3939595	1.6284572
MDC1	mediator of DNA-damage checkpoint 1	-1.2764357	-1.8079847	-2.573628
MDGA1	MAM domain containing glycosylphosphatidylinositol anchor 1	-1.1131179	1.7178149	4.076656
MDH1	malate dehydrogenase 1, NAD (soluble)	2.1048293	2.3402903	1.6781358
MDH1	malate dehydrogenase 1, NAD (soluble)	2.3867083	2.7754335	2.013331
MDH1B	malate dehydrogenase 1B, NAD (soluble)	-3.5747955	-2.759699	-3.10907
MDK	midkine (neurite growth-promoting factor 2)	-2.2965024	-3.9265208	-10.83665
MDM2	MDM2 proto-oncogene, E3 ubiquitin protein ligase	-1.0939163	-1.2275927	-2.7091055
MDM2	MDM2 proto-oncogene, E3 ubiquitin protein ligase	1.3722408	-1.0039293	-1.7881353
MDM2	MDM2 proto-oncogene, E3 ubiquitin protein ligase	-1.0970392	-1.3985773	-2.0820036
MDM2	MDM2 proto-oncogene, E3 ubiquitin protein ligase	1.0245125	-1.4535439	-1.9130508
MDP1	magnesium-dependent phosphatase 1	1.4366068	1.405236	2.0219223
ME3	malic enzyme 3, NADP(+)-dependent, mitochondrial	1.1626787	-1.0472783	-1.5879991
MEAF6	MYST/Esa1-associated factor 6	-2.0220323	-1.8755507	-1.7862192
MECOM	MDS1 and EVI1 complex locus	1.373651	-1.0778416	-1.8167634
MED11	mediator complex subunit 11	1.0698076	1.498215	1.8375947
MED15	mediator complex subunit 15	-1.2073989	-1.5107045	-2.0316615
MED18	mediator complex subunit 18	1.914329	2.2416077	2.1186428
MED25	mediator complex subunit 25	-1.3943552	-1.5701065	-1.5420972
MED26	mediator complex subunit 26	1.4236608	1.2709938	1.5550425
MEF2BNB	MEF2B neighbor	1.5119417	1.5619695	1.9848326
MEFV	Mediterranean fever	2.49634	2.5832725	3.185155
MEG3	maternally expressed 3 (non-protein coding)	2.1683202	1.8905342	1.8269504
MEG3	maternally expressed 3 (non-protein coding)	2.3405087	2.2991486	1.95838
MEGF6	multiple EGF-like-domains 6	-1.499698	-2.0205932	-7.7945127
MEGF9	multiple EGF-like-domains 9	1.024491	2.209975	2.292984
MELK	maternal embryonic leucine zipper kinase	2.081069	-3.2045274	-4.5395894
MESDC1	mesoderm development candidate 1	1.1751485	-1.2603803	-1.6914316
MESP1	mesoderm posterior basic helix-loop-helix transcription factor 1	1.7033836	3.3202908	5.884706
MEST	mesoderm specific transcript	1.4168963	1.233336	1.7564809
MET	MET proto-oncogene, receptor tyrosine kinase	-1.0504925	-1.095447	-1.5639179
METRNL	meteorin, glial cell differentiation regulator-like	1.4238542	1.0139793	-2.8475406
METTL1	methyltransferase like 1	-1.0234797	-1.3745834	-1.7014681
METTL1	methyltransferase like 1	-1.182086	-1.4996598	-2.0307777
METTL15	methyltransferase like 15	1.2410101	1.4593163	1.9154986
METTL15	methyltransferase like 15	-1.5326486	-1.1010356	1.125319
METTL25	methyltransferase like 25	1.7132038	1.5369691	1.595135
METTL7A	methyltransferase like 7A	2.59792	6.3522577	8.8878
METTL7B	methyltransferase like 7B	-1.3708563	3.8532915	8.688242
METTL8	methyltransferase like 8	1.1795673	-1.0563833	-1.714218
METTL8	methyltransferase like 8	-1.0551125	-1.3246628	-2.047146
MEX3D	mex-3 RNA binding family member D	-1.8500105	2.5636735	3.7266803
MFAP3L	microfibrillar-associated protein 3-like	1.3720528	1.4820306	2.3362694
MFF	mitochondrial fission factor	1.7427787	1.7331781	1.5341511
MFGE8	milk fat globule-EGF factor 8 protein	1.0595778	1.6975399	-2.170275
MFN1	mitofusin 1	1.2033589	1.3515637	1.6225852
MFN2	mitofusin 2	1.5729517	1.5947953	1.4839159
MFSD1	major facilitator superfamily domain containing 1	-1.5054379	-2.494455	-2.242107
MFSD10	major facilitator superfamily domain containing 10	-1.8330392	-1.6129763	-3.0484238
MFSD11	major facilitator superfamily domain containing 11	-1.3948337	-2.070629	1.0356647
MFSD2A	major facilitator superfamily domain containing 2A	1.1121551	2.7046502	12.217856
MFSD3	major facilitator superfamily domain containing 3	-1.2887199	1.0105648	1.646353
MFSD6	major facilitator superfamily domain containing 6	-1.3401749	-1.5119157	1.0350434
MFSD9	major facilitator superfamily domain containing 9	-1.671715	-1.1123089	1.4886125
MGA	MGA, MAX dimerization protein	2.940308	3.1187103	3.1695998
MGAT1	mannosyl (alpha-1,3-)-glycoprotein beta-1,2-N-acetylglucosaminyltransferase	-1.0243281	1.2454284	1.787921
MGAT2	mannosyl (alpha-1,6-)-glycoprotein beta-1,2-N-acetylglucosaminyltransferase	1.6429077	1.5226448	2.492886
MGAT4A	mannosyl (alpha-1,3-)-glycoprotein beta-1,4-N-acetylglucosaminyltransferase, isozyme A	-1.6730292	-1.55065	1.5400591
MGC10814	uncharacterized protein MGC10814	-2.477842	-2.299279	-1.922756
MGC27345	uncharacterized protein MGC27345	1.1192117	-1.1937044	-2.0047765
MGC34796	sepiapterin reductase (7,8-dihydrobiopterin:NADP+ oxidoreductase) pseudogene	2.0052552	2.5487893	2.3894515
MGC45922	uncharacterized LOC284365	2.237186	2.2487926	2.730678
MGEA5	meningioma expressed antigen 5 (hyaluronidase)	-1.3379246	-1.3396692	1.6891204
MGEA5	meningioma expressed antigen 5 (hyaluronidase)	-3.1528015	-4.0440536	-2.0613627
MGLL	monoglyceride lipase	1.0992562	1.2016262	-2.444689
MGLL	monoglyceride lipase	1.1934431	-1.0046196	-1.9077259
MGME1	mitochondrial genome maintenance exonuclease 1	1.2314979	-1.5128237	-2.1629195
MGMT	O-6-methylguanine-DNA methyltransferase	1.217301	1.6522	2.3508737
MGP	matrix Gla protein	1.6087453	5.7515907	1.9249179
MGP	matrix Gla protein	2.077854	7.2791595	2.3865044
MGST1	microsomal glutathione S-transferase 1	1.0634114	1.3921838	2.4609246
MGST2	microsomal glutathione S-transferase 2	1.4289755	1.5900762	1.56966
MIA2	melanoma inhibitory activity 2	-5.0768347	-1.3735496	-1.2075723
MIA3	melanoma inhibitory activity family, member 3	-1.9654132	-1.4816278	-1.0835896
MIATNB	MIAT neighbor (non-protein coding)	3.0322745	2.71108	2.8885574
MIB2	mindbomb E3 ubiquitin protein ligase 2	-1.7153519	-1.4504573	-1.4492153
MICA	MHC class I polypeptide-related sequence A	1.5941252	-1.3326569	-1.0317131
MICALL1	MICAL-like 1	1.1729864	-2.4137144	-4.527822
MICALL2	MICAL-like 2	-2.2970905	-4.168444	-6.480067
MICB	MHC class I polypeptide-related sequence B	1.3064102	-1.5299218	-1.0613078
MICB	MHC class I polypeptide-related sequence B	-1.3592061	-3.8642197	-6.6155434
MID1IP1	MID1 interacting protein 1	1.8341639	2.754067	6.08254
MIDN	midnolin	1.2238988	-1.0628783	1.660853
MIEF1	mitochondrial elongation factor 1	1.5105361	1.7162826	2.0166538
MIEN1	migration and invasion enhancer 1	1.597672	1.4443442	1.7619541
MIER3	mesoderm induction early response 1, family member 3	1.1583221	1.21646	1.8667219
MIF	macrophage migration inhibitory factor (glycosylation-inhibiting factor)	1.5072049	1.1546674	-1.0330983
MIF-AS1	MIF antisense RNA 1	1.6306027	1.1021292	-1.199658
MINOS1	mitochondrial inner membrane organizing system 1	1.657544	1.5172323	1.138602
MINOS1-NBL1	MINOS1-NBL1 readthrough	1.0555543	-1.9952904	-3.8144844
MIR143HG	MIR143 host gene (non-protein coding)	1.4697058	7.9625807	73.05332
MIR4435-1HG	MIR4435-1 host gene (non-protein coding)	1.0119369	-1.936817	-3.2921522
MIS18A	MIS18 kinetochore protein A	-1.2067113	-3.1080196	-2.942381
MITD1	MIT, microtubule interacting and transport, domain containing 1	-1.3019874	-1.7126023	-1.7460455
MITD1	MIT, microtubule interacting and transport, domain containing 1	-1.0670456	-1.5630074	-1.3976786
MITF	microphthalmia-associated transcription factor	3.1307669	2.5868943	1.6182415
MITF	microphthalmia-associated transcription factor	2.5769832	2.0330012	1.3486098
MKKS	McKusick-Kaufman syndrome	1.6079614	1.4491981	1.3125885
MKL1	megakaryoblastic leukemia (translocation) 1	-1.0177623	-2.0778227	-2.7284088
MKLN1	muskelin 1, intracellular mediator containing kelch motifs	-1.9355164	-1.1185027	1.1982056
MKNK1	MAP kinase interacting serine/threonine kinase 1	-1.6818116	-1.4759558	-1.4337357

MKNK2	MAP kinase interacting serine/threonine kinase 2	1.3055727	1.3463116	2.1411157
MLKL	mixed lineage kinase domain-like	-1.1425339	-1.9044615	-6.537581
MLLT1	myeloid/lymphoid or mixed-lineage leukemia (trithorax homolog, Drosophila); translocated to	-1.6787244	-1.7292168	-2.185169
MLLT4	myeloid/lymphoid or mixed-lineage leukemia (trithorax homolog, Drosophila); translocated to	-2.1705978	-2.7602131	-1.9413227
MLLT4	myeloid/lymphoid or mixed-lineage leukemia (trithorax homolog, Drosophila); translocated to	-1.4318712	-1.6009369	-1.2556654
MLLT6	myeloid/lymphoid or mixed-lineage leukemia (trithorax homolog, Drosophila); translocated to	-1.2009854	-1.7943358	-2.346393
MLPH	melanophilin	-2.067172	-4.642061	-9.401424
MLX	MLX, MAX dimerization protein	1.2778596	1.6795402	1.5872622
MLXIP	MLX interacting protein	-1.2165122	-1.666304	-1.5280129
MLXIPL	MLX interacting protein-like	-2.3296103	2.7475016	4.9811373
MLYCD	malonyl-CoA decarboxylase	1.6958772	1.6934648	2.2382083
MMAA	methylmalonic aciduria (cobalamin deficiency) cblA type	-1.3063788	1.6704584	2.7710216
MMAB	methylmalonic aciduria (cobalamin deficiency) cblB type	1.5423363	1.976219	4.655576
MMACHC	methylmalonic aciduria (cobalamin deficiency) cblC type, with homocystinuria	2.5577893	3.4696345	1.5953367
MMD	monocyte to macrophage differentiation-associated	1.1939883	-1.9750975	-3.8413026
MME	membrane metallo-endopeptidase	1.1294498	1.6915262	2.943702
MME	membrane metallo-endopeptidase	1.438971	2.3398063	3.6863592
MME	membrane metallo-endopeptidase	1.3553576	2.032393	3.6942644
MMP14	matrix metallopeptidase 14 (membrane-inserted)	-3.922251	-7.1550064	-10.787789
MMP17	matrix metallopeptidase 17 (membrane-inserted)	2.3581374	2.1280797	2.1964128
MMP19	matrix metallopeptidase 19	-1.0037726	4.9207225	6.6482167
MMP19	matrix metallopeptidase 19	-1.323238	-1.507981	-3.9093466
MMP24	matrix metallopeptidase 24 (membrane-inserted)	2.0601513	1.39827	-1.0136707
MMP24-AS1	MMP24 antisense RNA 1	1.5345544	1.5448116	2.4284708
MMP25-AS1	MMP25 antisense RNA 1	2.4373536	1.9909365	2.1121736
MMP25-AS1	MMP25 antisense RNA 1	1.6135732	2.0416949	2.4003866
MMP28	matrix metallopeptidase 28	1.8668047	1.8450315	1.567632
MND1	meiotic nuclear divisions 1 homolog (S. cerevisiae)	1.8859353	-3.2622514	-3.3735497
MOB1B	MOB kinase activator 1B	1.552863	2.0322256	2.4052625
MOB3B	MOB kinase activator 3B	-1.2396228	2.134072	-1.1856049
MOCS1	molybdenum cofactor synthesis 1	1.1639459	2.8586557	3.5972006
MOCS1	molybdenum cofactor synthesis 1	1.296144	3.1471987	4.0842943
MOCS2	molybdenum cofactor synthesis 2	-1.0773228	-1.0455006	1.7553214
MOCS3	molybdenum cofactor synthesis 3	5.625818	6.026905	7.6425567
MOG	myelin oligodendrocyte glycoprotein	2.2389166	2.1621706	2.314026
MOGAT1	monoacylglycerol O-acyltransferase 1	1.0766556	3.2223406	13.266947
MOGS	mannosyl-oligosaccharide glucosidase	1.32191	1.4987512	1.763267
MOK	MOK protein kinase	1.0842614	-2.393093	-3.9276865
MON1A	MON1 secretory trafficking family member A	1.0125762	1.3455552	1.5282418
MON1B	MON1 secretory trafficking family member B	2.5438783	2.6427453	3.1915326
MORC4	MORC family CW-type zinc finger 4	-1.7820641	-2.3813758	-1.8055793
MORF4L2	mortality factor 4 like 2	1.0128614	-1.475043	-1.5123941
MOSPD1	motile sperm domain containing 1	1.6956043	-1.1290857	-1.909628
MOSPD3	motile sperm domain containing 3	1.7708302	1.778296	1.6323849
MOXD1	monooxygenase, DBH-like 1	-1.1008755	-2.580017	-4.7189302
MPC1	mitochondrial pyruvate carrier 1	2.5223277	3.689279	3.6285703
MPC1	mitochondrial pyruvate carrier 1	2.1037219	2.9124947	2.7040565
MPC2	mitochondrial pyruvate carrier 2	1.3178288	2.114881	2.880785
MPDZ	multiple PDZ domain protein	-1.2131032	2.4232352	2.4989429
MPDZ	multiple PDZ domain protein	-2.1151612	1.3313161	1.5498853
MPHOSPH6	M-phase phosphoprotein 6	1.2634709	-1.8680488	-1.9627969
MPI	mannose phosphate isomerase	1.4924357	1.8686291	1.5440975
MPP1	membrane protein, palmitoylated 1, 55kDa	1.9545631	1.9926845	4.9071145
MPP3	membrane protein, palmitoylated 3 (MAGUK p55 subfamily member 3)	-1.0009778	-1.7568154	-2.1326172
MPP5	membrane protein, palmitoylated 5 (MAGUK p55 subfamily member 5)	1.8209311	1.2766173	1.5673705
MPPE1	metallophosphoesterase 1	1.0406423	1.4180279	1.6216052
MPRIP	myosin phosphatase Rho interacting protein	1.5408872	-1.1714607	-1.6749194
MPRIP	myosin phosphatase Rho interacting protein	-1.2318814	-2.3669941	-2.7297719
MPST	mercaptopyruvate sulfurtransferase	-1.2355658	1.6578087	2.535831
MPV17	MpV17 mitochondrial inner membrane protein	1.0416209	1.0961089	-1.5105872
MPV17L2	MPV17 mitochondrial membrane protein-like 2	1.3941662	1.1201193	1.6017859
MPZL1	myelin protein zero-like 1	-1.2333844	-1.9317015	-1.7096547
MPZL2	myelin protein zero-like 2	-1.4643171	1.9073675	1.4886918
MPZL3	myelin protein zero-like 3	-2.1184342	-2.4558218	-1.8397034
MRC2	mannose receptor, C type 2	-1.3607063	-9.385184	-11.954235
MRE11A	MRE11 meiotic recombination 11 homolog A (S. cerevisiae)	-1.7843959	-2.5992439	-2.6901503
MRGBP	MRG/MORF4L binding protein	-1.3046303	-1.4630512	-1.5694351
MROH6	maestro heat-like repeat family member 6	1.2613425	2.0408106	2.6958861
MRPL15	mitochondrial ribosomal protein L15	1.9561647	2.143584	1.22806
MRPL16	mitochondrial ribosomal protein L16	1.5860776	2.153768	2.359914
MRPL17	mitochondrial ribosomal protein L17	1.5550344	1.2695047	-1.126876
MRPL19	mitochondrial ribosomal protein L19	1.3388472	1.3293555	1.9032873
MRPL20	mitochondrial ribosomal protein L20	1.5124705	1.5063529	1.6970567
MRPL21	mitochondrial ribosomal protein L21	1.591864	1.6749977	1.4009104
MRPL23	mitochondrial ribosomal protein L23	1.177771	2.384072	27.915937
MRPL3	mitochondrial ribosomal protein L3	1.5269569	1.4251881	1.3398763
MRPL30	mitochondrial ribosomal protein L30	1.4999716	1.370149	1.7139051
MRPL32	mitochondrial ribosomal protein L32	1.4414566	1.568677	1.4532422
MRPL33	mitochondrial ribosomal protein L33	1.8575149	1.2084944	-1.1748818
MRPL34	mitochondrial ribosomal protein L34	1.2878096	1.6139256	1.5823306
MRPL4	mitochondrial ribosomal protein L4	1.7073525	1.9656787	1.5831531
MRPL40	mitochondrial ribosomal protein L40	1.4996377	1.9744327	1.8559309
MRPL41	mitochondrial ribosomal protein L41	1.287604	1.9739432	1.9316965
MRPL47	mitochondrial ribosomal protein L47	1.6408921	1.5226412	1.0285652
MRPL52	mitochondrial ribosomal protein L52	-1.1180611	-1.4254899	-2.8538465
MRPL53	mitochondrial ribosomal protein L53	1.504622	1.5125195	1.633986
MRPL53	mitochondrial ribosomal protein L53	1.4905504	1.4166028	1.5522918
MRPL57	mitochondrial ribosomal protein L57	-1.5912743	-1.4494019	-2.1869905
MRPS12	mitochondrial ribosomal protein S12	1.9660212	2.5984538	1.5985647
MRPS14	mitochondrial ribosomal protein S14	1.425825	1.2297649	1.7150711
MRPS18B	mitochondrial ribosomal protein S18B	1.3173181	1.9110181	1.5450194
MRPS18C	mitochondrial ribosomal protein S18C	1.5134788	1.4057796	1.714825
MRPS21	mitochondrial ribosomal protein S21	1.5270904	1.47959	1.0656816
MRPS22	mitochondrial ribosomal protein S22	1.5621834	1.8441294	1.7787769
MRPS24	mitochondrial ribosomal protein S24	1.8564154	1.8312347	1.2947152
MRPS26	mitochondrial ribosomal protein S26	1.4492888	1.7345355	1.8350518
MRPS28	mitochondrial ribosomal protein S28	1.2932686	1.2811213	1.5314387
MRPS31	mitochondrial ribosomal protein S31	1.1094278	1.1369691	1.5509616
MRPS36	mitochondrial ribosomal protein S36	2.5497012	3.0359902	2.7434378
MRPS36	mitochondrial ribosomal protein S36	1.8934911	2.2755656	2.0586345
MRPS9	mitochondrial ribosomal protein S9	1.4113349	1.6006943	1.4169831
MRS2	MRS2 magnesium transporter	2.3681822	2.1950758	1.3376678
MSANTD3	Myb/SANT-like DNA-binding domain containing 3	-2.022092	-3.6407616	-4.7709713
MSC	musculin	-1.7441576	-5.6347604	-11.210152
MSH2	mutS homolog 2	1.3657681	-1.6709228	-1.8444034
MSH2	mutS homolog 2	1.2526543	-1.9371582	-2.1382372
MSH6	mutS homolog 6	1.0857667	-1.5406811	-1.2084804
MSL1	male-specific lethal 1 homolog (Drosophila)	-1.1761168	-1.149332	-1.7077862
MSN	moesin	-1.114746	-1.4479489	-2.6119192

MSRA	methionine sulfoxide reductase A	1.0286692	1.216047	2.13665
MSRB1	methionine sulfoxide reductase B1	1.3370475	1.6399834	2.4634125
MSRB2	methionine sulfoxide reductase B2	1.0847708	1.6515819	2.3736844
MSRB2	methionine sulfoxide reductase B2	1.6063927	1.4639752	2.3660233
MSRB3	methionine sulfoxide reductase B3	1.8713772	1.7001891	1.110351
MST1	macrophage stimulating 1 (hepatocyte growth factor-like)	-1.6119541	2.0644145	3.9936423
MT1A	metallothionein 1A	1.9283023	1.4370764	2.1300244
MT1B	metallothionein 1B	2.2808797	1.5438545	2.3768451
MT1E	metallothionein 1E	2.4735858	1.7486929	2.551889
MT1HL1	metallothionein 1H-like 1	2.04735	1.4333498	2.3326044
MT1L	metallothionein 1L (gene/pseudogene)	1.8916855	1.2439278	2.0244207
MT1M	metallothionein 1M	5.751116	3.957135	5.453292
MT1X	metallothionein 1X	3.4774284	6.163529	13.14254
MT1X	metallothionein 1X	1.7644141	1.4284062	2.5457838
MT2A	metallothionein 2A	1.7435216	1.3819433	2.168264
MT3	metallothionein 3	2.04808	1.8041701	1.8527532
MTAP	methylthioadenosine phosphorylase	-1.1642841	-1.3865912	-1.9851971
MTAP	methylthioadenosine phosphorylase	1.1460978	-1.0604047	-1.9273847
MTCH2	mitochondrial carrier 2	-1.1379476	1.2313529	1.7566553
MTERF4	mitochondrial transcription termination factor 4	1.2284833	1.2824955	1.584985
MTERF4	mitochondrial transcription termination factor 4	1.2314718	1.3161631	1.5886097
MTF2	metal response element binding transcription factor 2	-1.1664441	-1.7869415	-1.8257697
MTFR1L	mitochondrial fission regulator 1-like	1.7848955	2.376718	2.0036612
MTFR2	mitochondrial fission regulator 2	2.0481036	-2.841731	-3.8701832
MTHFD1L	methylenetetrahydrofolate dehydrogenase (NADP+ dependent) 1-like	-1.4467999	-3.3954823	-10.146819
MTHFD1L	methylenetetrahydrofolate dehydrogenase (NADP+ dependent) 1-like	-1.321629	-2.1843007	-5.352004
MTHFS	5,10-methenyltetrahydrofolate synthetase (5-formyltetrahydrofolate cyclo-ligase)	-1.1281924	1.3274446	2.541371
MTMR10	myotubularin related protein 10	1.0658463	1.6383216	2.267638
MTMR14	myotubularin related protein 14	1.3606402	1.9053382	2.0974858
MTMR14	myotubularin related protein 14	1.3337079	1.5277419	2.4661837
MTMR2	myotubularin related protein 2	1.4938135	-1.1825835	-1.8589449
MTMR2	myotubularin related protein 2	1.3988289	-1.4402567	-2.3960621
MTMR4	myotubularin related protein 4	1.5314211	1.9873815	2.4986286
MTR	5-methyltetrahydrofolate-homocysteine methyltransferase	-1.3099636	-1.3603383	-1.6449682
MTRF1L	mitochondrial translational release factor 1-like	1.0278679	-1.2245712	-1.5833968
MTSS1	metastasis suppressor 1	-1.650249	1.5740122	2.5185459
MTTP	microsomal triglyceride transfer protein	-5.706053	2.9143724	8.067139
MTURN	maturin, neural progenitor differentiation regulator homolog (Xenopus)	-1.657078	-1.643143	-1.0276991
MUC15	mucin 15, cell surface associated	-3.2083273	-3.031824	-1.0275496
MUC2	mucin 2, oligomeric mucus/gel-forming	1.4134815	1.4679686	1.6713687
MUC3A	mucin 3A, cell surface associated	2.399427	2.2290156	2.354145
MUC4	mucin 4, cell surface associated	1.9267288	2.0722198	2.595053
MUC4	mucin 4, cell surface associated	2.2098436	2.5336518	2.4501674
MUC4	mucin 4, cell surface associated	1.5704432	1.660413	2.1582322
MUC6	mucin 6, oligomeric mucus/gel-forming	2.0092642	2.1266367	2.506702
MUM1	melanoma associated antigen (mutated) 1	-1.5189532	-1.0480942	1.1051446
MUM1L1	melanoma associated antigen (mutated) 1-like 1	-1.7737485	2.2675064	1.8317535
MUS81	MUS81 structure-specific endonuclease subunit	-1.4216969	-1.7047981	-2.434139
MUSK	muscle, skeletal, receptor tyrosine kinase	1.8992244	1.8211119	5.399678
MUT	methylmalonyl CoA mutase	1.0591944	2.7890787	4.081848
MVB12B	multivesicular body subunit 12B	1.1346676	1.0840216	1.6652462
MVD	mevalonate (diphospho) decarboxylase	1.5077908	1.6983428	2.9235876
MVK	mevalonate kinase	1.7521774	1.9173385	3.5729809
MXD1	MAX dimerization protein 1	-1.9063431	-2.3251367	-1.0233544
MXI1	MAX interactor 1, dimerization protein	1.1163262	1.4713733	2.2919872
MXI1	MAX interactor 1, dimerization protein	-1.6388319	-1.0614479	1.2921258
MXRA7	matrix-remodelling associated 7	1.5716046	-1.2473977	-1.8053074
MXRA7	matrix-remodelling associated 7	1.1544372	-1.8863815	-2.562405
MYADM	myeloid-associated differentiation marker	1.1775261	-1.739795	-7.735231
MYBBP1A	MYB binding protein (P160) 1a	-1.0211724	-1.1317691	-1.8668643
MYBL1	v-myb avian myeloblastosis viral oncogene homolog-like 1	2.2414277	-1.6721245	-7.218888
MYC	v-myc avian myelocytomatosis viral oncogene homolog	1.7853158	-1.3045882	-1.5477716
MYCBP2	MYC binding protein 2, E3 ubiquitin protein ligase	-1.5359689	1.7606795	-1.197012
MYCBP2	MYC binding protein 2, E3 ubiquitin protein ligase	-1.5127138	1.9971881	1.0223761
MYCL	v-myc avian myelocytomatosis viral oncogene lung carcinoma derived homolog	1.3781476	2.2865129	9.488726
MYEOV2	myeloma overexpressed 2	1.5365223	1.4460602	1.2476124
MYH14	myosin, heavy chain 14, non-muscle	2.7003217	2.7738433	2.873204
MYH9	myosin, heavy chain 9, non-muscle	-2.0736945	-3.767457	-4.701165
MYL12A	myosin, light chain 12A, regulatory, non-sarcomeric	1.9764773	1.6442932	1.0323048
MYL12B	myosin, light chain 12B, regulatory	1.589962	1.3839055	1.1110501
MYL12B	myosin, light chain 12B, regulatory	1.5767385	1.3305278	-1.070644
MYL6B	myosin, light chain 6B, alkali, smooth muscle and non-muscle	1.093701	-1.3684208	-3.4904387
MYLIP	myosin regulatory light chain interacting protein	1.7379223	1.7204131	1.8253963
MYLK	myosin light chain kinase	-1.194601	-1.5471169	-1.6701355
MYO15B	myosin XVB pseudogene	1.5382506	-1.0127877	-1.0843493
MYO15B	myosin XVB pseudogene	1.3342425	2.6286323	3.7068315
MYO19	myosin XIX	1.2674377	-1.216057	-3.8247063
MYO19	myosin XIX	-1.0224309	-1.0546682	-2.014732
MYO1B	myosin IB	-1.2502278	1.4798138	2.193056
MYO1C	myosin IC	-2.003066	-1.7229613	-1.6157769
MYO1E	myosin IE	1.2525387	-1.2469113	-1.5157709
MYO5A	myosin VA (heavy chain 12, myoxin)	-1.8470944	-2.4283266	-2.3344378
MYO6	myosin VI	-1.7329019	-1.8169289	-1.8522506
MYO9A	myosin IXA	-1.4109197	-1.1252168	-1.5245723
MYO9B	myosin IXB	-1.725893	-2.3808312	-1.9201051
MYOF	myoferlin	-1.5624765	-2.6274781	-7.30147
MYOF	myoferlin	-1.0856905	-1.8362018	-4.651067
MYRF	myelin regulatory factor	-1.2420449	-1.614685	-2.0066268
MZF1	myeloid zinc finger 1	-1.5617129	-1.665626	-1.3945544
MZT1	mitotic spindle organizing protein 1	1.6035748	-1.5938762	-1.9996068
MZT2B	mitotic spindle organizing protein 2B	1.5378829	1.3284165	1.2473817
N4BP2L2	NEDD4 binding protein 2-like 2	-1.5599412	-1.3487576	-1.2924777
N6AMT2	N-6 adenine-specific DNA methyltransferase 2 (putative)	1.2170691	1.3218867	3.1312513
NAA25	N(alpha)-acetyltransferase 25, NatB auxiliary subunit	1.1158959	-1.3133996	-1.6395065
NAA38	N(alpha)-acetyltransferase 38, NatC auxiliary subunit	1.39937	1.4706595	1.9695134
NAA50	N(alpha)-acetyltransferase 50, NatE catalytic subunit	-1.0448356	-1.7531997	-1.9869987
NAA60	N(alpha)-acetyltransferase 60, NatF catalytic subunit	1.1592965	1.5363882	2.0120034
NAAA	N-acylethanolamine acid amidase	-1.1350853	1.9000467	3.172329
NAAA	N-acylethanolamine acid amidase	-1.1071717	2.0316331	3.2805972
NAB1	NGFI-A binding protein 1 (EGR1 binding protein 1)	-1.3232753	-1.3839434	-1.8568426
NAB1	NGFI-A binding protein 1 (EGR1 binding protein 1)	-1.8631448	-2.3175342	-2.9412036
NAB2	NGFI-A binding protein 2 (EGR1 binding protein 2)	1.444061	1.9642092	1.7132987
NABP1	nucleic acid binding protein 1	2.3955991	1.7640746	-1.081923
NACA	nascent polypeptide-associated complex alpha subunit	1.5981041	1.4193809	1.1300669
NACA2	nascent polypeptide-associated complex alpha subunit 2	1.6444536	1.3753887	1.100041
NACAP1	nascent-polypeptide-associated complex alpha polypeptide pseudogene 1	2.5667684	2.402204	1.7693965
NACC1	nucleus accumbens associated 1, BEN and BTB (POZ) domain containing	1.5228679	1.0391923	1.2394989
NACC2	NACC family member 2, BEN and BTB (POZ) domain containing	1.845917	-1.0477691	-1.2793334
NAE1	NEDD8 activating enzyme E1 subunit 1	1.640819	1.1958373	1.6620588

NAGA	N-acetylgalactosaminidase, alpha-	1.1663977	1.0757155	1.5957674
NAGLU	N-acetylglucosaminidase, alpha	-1.2952489	-1.6533498	-1.6784883
NAMPT	nicotinamide phosphoribosyltransferase	-1.314999	1.9981493	1.4243302
NAMPT	nicotinamide phosphoribosyltransferase	-2.7070801	-1.2774615	-1.7857738
NAMPT	nicotinamide phosphoribosyltransferase	-3.3866963	-2.1426854	-2.3741624
NAP1L1	nucleosome assembly protein 1-like 1	1.6154737	1.1551628	-1.3951919
NAP1L2	nucleosome assembly protein 1-like 2	1.0486916	1.1226475	1.8556345
NAP1L5	nucleosome assembly protein 1-like 5	1.5906193	1.1382911	2.0650935
NAP1L6	nucleosome assembly protein 1-like 6	1.3968815	1.4550854	1.7728848
NAPEPLD	N-acyl phosphatidylethanolamine phospholipase D	1.2805022	1.7929413	1.91143
NAPRT	nicotinate phosphoribosyltransferase	1.4472619	3.4556775	3.77719
NAT1	N-acetyltransferase 1 (arylamine N-acetyltransferase)	1.8312526	1.159349	1.6604466
NAT10	N-acetyltransferase 10 (GCN5-related)	1.6077672	1.439635	1.3395656
NAT14	N-acetyltransferase 14 (GCN5-related, putative)	1.6835845	1.5213495	1.4859117
NAT2	N-acetyltransferase 2 (arylamine N-acetyltransferase)	1.683958	3.8492162	6.2239437
NAT8	N-acetyltransferase 8 (GCN5-related, putative)	-2.5428884	3.051807	1.7699456
NAV2	neuron navigator 2	-1.2132396	-1.6693516	-3.1055183
NBAS	neuroblastoma amplified sequence	-1.7948492	-1.4976646	-1.387012
NBEAL1	neurobeachin-like 1	-2.8331437	-1.4884171	-1.6689558
NBEAP1	neurobeachin pseudogene 1	-1.1542612	-2.2965324	-3.5325456
NBPF10	neuroblastoma breakpoint family, member 10	-2.3190002	-1.710343	-1.4786816
NBPF14	neuroblastoma breakpoint family, member 14	-2.3014455	-1.9803338	-1.6760983
NBR1	neighbor of BRCA1 gene 1	1.0901203	1.165796	1.5719701
NCAM1	neural cell adhesion molecule 1	-2.9895012	-6.032524	-5.648406
NCAPD2	non-SMC condensin I complex, subunit D2	-1.1913377	-2.9325762	-3.0526285
NCAPD2	non-SMC condensin I complex, subunit D2	1.8386109	-1.4744272	-1.4076735
NCAPD3	non-SMC condensin II complex, subunit D3	-1.173399	-2.9285462	-2.2329643
NCBP2-AS2	NCBP2 antisense RNA 2 (head to head)	1.7854333	2.0646799	1.8002275
NCEH1	neutral cholesterol ester hydrolase 1	-1.1829414	-1.2913342	-2.3651605
NCF1	neutrophil cytosolic factor 1	2.5065312	2.9657476	4.2786417
NCF2	neutrophil cytosolic factor 2	-1.4678795	-6.3969116	-26.36275
NCK2	NCK adaptor protein 2	1.7465092	1.6066965	1.433472
NCKAP5L	NCK-associated protein 5-like	1.5498074	1.5141083	1.6121396
NCLN	nicalin	-1.1564567	-1.724296	-1.841471
NCOA7	nuclear receptor coactivator 7	-2.5496883	-1.9201245	-2.173347
NCOR2	nuclear receptor corepressor 2	-1.0181913	-1.5015303	-1.4804286
NCS1	neuronal calcium sensor 1	1.1544367	-1.8367366	-2.1146271
ND1	NADH dehydrogenase, subunit 1 (complex I)	-2.6187797	-1.6258175	-2.5242505
NDC1	NDC1 transmembrane nucleoporin	-1.1419479	-2.0055318	-1.7887851
NDC80	NDC80 kinetochore complex component	2.6811762	-2.8502054	-3.2851865
NDE1	nudE neurodevelopment protein 1	1.4169478	-1.3008732	-1.7131097
NDFIP1	Nedd4 family interacting protein 1	1.4375309	1.5351324	2.1769197
NDRG1	N-myc downstream regulated 1	-2.289152	1.0426756	2.6853328
NDRG2	NDRG family member 2	1.5134064	3.0310214	2.128672
NDRG2	NDRG family member 2	1.550699	3.1373978	2.151435
NDST1	N-deacetylase/N-sulfotransferase (heparan glucosaminyl) 1	2.3076599	1.4859672	1.5096931
NDST2	N-deacetylase/N-sulfotransferase (heparan glucosaminyl) 2	1.7249988	1.674063	2.6360624
NDUFA1	NADH dehydrogenase (ubiquinone) 1 alpha subcomplex, 1, 7.5kDa	1.7005267	1.7719415	1.6696587
NDUFA11	NADH dehydrogenase (ubiquinone) 1 alpha subcomplex, 11, 14.7kDa	1.719862	1.5041898	1.3469031
NDUFA12	NADH dehydrogenase (ubiquinone) 1 alpha subcomplex, 12	1.8206253	1.9696993	1.6327376
NDUFA12	NADH dehydrogenase (ubiquinone) 1 alpha subcomplex, 12	1.6350156	1.9372666	1.599951
NDUFA2	NADH dehydrogenase (ubiquinone) 1 alpha subcomplex, 2, 8kDa	1.5542681	2.023138	2.4442286
NDUFA3	NADH dehydrogenase (ubiquinone) 1 alpha subcomplex, 3, 9kDa	1.4415973	1.4858592	2.2904813
NDUFA5	NADH dehydrogenase (ubiquinone) 1 alpha subcomplex, 5	1.4450496	1.8386724	1.901854
NDUFA6	NADH dehydrogenase (ubiquinone) 1 alpha subcomplex, 6, 14kDa	1.9317038	2.1028018	1.8370934
NDUFA6-AS1	NDUFA6 antisense RNA 1 (head to head)	1.115476	1.3027846	2.3640985
NDUFA7	NADH dehydrogenase (ubiquinone) 1 alpha subcomplex, 7, 14.5kDa	1.6192329	1.6468934	1.7327654
NDUFA8	NADH dehydrogenase (ubiquinone) 1 alpha subcomplex, 8, 19kDa	2.1675062	2.4342484	1.7249242
NDUFAB1	NADH dehydrogenase (ubiquinone) 1, alpha/beta subcomplex, 1, 8kDa	1.3996367	1.7151006	1.8899858
NDUFAF1	NADH dehydrogenase (ubiquinone) complex I, assembly factor 1	-1.094501	1.2433866	1.9571819
NDUFAF2	NADH dehydrogenase (ubiquinone) complex I, assembly factor 2	-1.3186301	-1.6987871	-1.8497627
NDUFAF3	NADH dehydrogenase (ubiquinone) complex I, assembly factor 3	1.6980762	1.4919951	1.860974
NDUFAF4	NADH dehydrogenase (ubiquinone) complex I, assembly factor 4	1.6115835	1.3388442	-1.2117631
NDUFAF6	NADH dehydrogenase (ubiquinone) complex I, assembly factor 6	1.6436763	2.077663	1.711151
NDUFB1	NADH dehydrogenase (ubiquinone) 1 beta subcomplex, 1, 7kDa	1.6952282	2.1137433	2.245802
NDUFB10	NADH dehydrogenase (ubiquinone) 1 beta subcomplex, 10, 22kDa	2.040045	2.5135024	2.1944797
NDUFB2	NADH dehydrogenase (ubiquinone) 1 beta subcomplex, 2, 8kDa	1.6713344	1.6886879	1.6471567
NDUFB2-AS1	NDUFB2 antisense RNA 1	2.2464828	2.2639215	2.7707093
NDUFB3	NADH dehydrogenase (ubiquinone) 1 beta subcomplex, 3, 12kDa	1.7897404	1.9432737	1.6425385
NDUFB4	NADH dehydrogenase (ubiquinone) 1 beta subcomplex, 4, 15kDa	1.8452901	2.0850346	1.9511685
NDUFB5	NADH dehydrogenase (ubiquinone) 1 beta subcomplex, 5, 16kDa	1.6125128	2.3274794	1.6292917
NDUFB5	NADH dehydrogenase (ubiquinone) 1 beta subcomplex, 5, 16kDa	2.1244023	2.87784	2.0712035
NDUFB6	NADH dehydrogenase (ubiquinone) 1 beta subcomplex, 6, 17kDa	1.5819428	1.7569892	1.9486626
NDUFB7	NADH dehydrogenase (ubiquinone) 1 beta subcomplex, 7, 18kDa	1.4149979	1.651746	1.5058064
NDUFB8	NADH dehydrogenase (ubiquinone) 1 beta subcomplex, 8, 19kDa	1.5223128	1.4977487	1.3149178
NDUFB8	NADH dehydrogenase (ubiquinone) 1 beta subcomplex, 8, 19kDa	1.7481757	1.7196159	1.512182
NDUFB9	NADH dehydrogenase (ubiquinone) 1 beta subcomplex, 9, 22kDa	1.2243296	1.6782057	1.1414958
NDUFC1	NADH dehydrogenase (ubiquinone) 1, subcomplex unknown, 1, 6kDa	1.268823	1.546521	1.5110054
NDUFC2	NADH dehydrogenase (ubiquinone) 1, subcomplex unknown, 2, 14.5kDa	1.447198	2.0753102	2.0515175
NDUFC2	NADH dehydrogenase (ubiquinone) 1, subcomplex unknown, 2, 14.5kDa	1.5220939	1.5449424	2.2271876
NDUFS3	NADH dehydrogenase (ubiquinone) Fe-S protein 3, 30kDa (NADH-coenzyme Q reductase)	1.4257132	1.7447736	1.3728821
NDUFS5	NADH dehydrogenase (ubiquinone) Fe-S protein 5, 15kDa (NADH-coenzyme Q reductase)	1.6929586	1.3261466	1.3189046
NDUFS6	NADH dehydrogenase (ubiquinone) Fe-S protein 6, 13kDa (NADH-coenzyme Q reductase)	1.8626716	1.6220161	1.5985152
NDUFV2	NADH dehydrogenase (ubiquinone) flavoprotein 2, 24kDa	1.1266515	1.4345233	1.6709237
NDUFV3	NADH dehydrogenase (ubiquinone) flavoprotein 3, 10kDa	1.1400151	1.4138888	1.7496545
NEAT1	nuclear paraspeckle assembly transcript 1 (non-protein coding)	-2.1028824	-1.9859936	1.1742513
NECAP2	NECAP endocytosis associated 2	-1.0235963	-1.3011912	-1.5583627
NEDD1	neural precursor cell expressed, developmentally down-regulated 1	1.0572965	-1.4561601	-2.2483394
NEDD8	neural precursor cell expressed, developmentally down-regulated 8	1.5397913	1.3264563	1.3748925
NEGR1	neuronal growth regulator 1	1.6216656	1.9052489	3.012835
NEK4	NIMA-related kinase 4	1.0901049	1.2238199	1.6587659
NEK6	NIMA-related kinase 6	-1.2457823	1.5126864	1.0310093
NEK9	NIMA-related kinase 9	1.3243189	1.4728475	1.683579
NES	nestin	-2.2192721	-5.258358	-15.913249
NET1	neuroepithelial cell transforming 1	-1.2806145	1.9104621	2.8940842
NET1	neuroepithelial cell transforming 1	-1.3946041	1.5377417	1.8579984
NEU1	sialidase 1 (lysosomal sialidase)	-1.5206677	-2.287761	-1.6712079
NEURL1B	neuralized E3 ubiquitin protein ligase 1B	2.3059587	-1.5810835	-2.871115
NEURL2	neuralized E3 ubiquitin protein ligase 2	1.5238345	2.6517572	1.4758871
NEUROG3	neurogenin 3	1.7216898	2.6514335	4.3015456
NF2	neurofibromin 2 (merlin)	1.9033674	-1.0220524	1.0698233
NFATC2IP	nuclear factor of activated T-cells, cytoplasmic, calcineurin-dependent 2 interacting protein	-1.6890023	-1.7120671	-2.2082384
NFE2L3	nuclear factor, erythroid 2-like 3	-1.0550513	-3.512516	-12.405306
NFKB1	nuclear factor of kappa light polypeptide gene enhancer in B-cells 1	-1.373037	-1.5167549	-2.1767378
NFKBIA	nuclear factor of kappa light polypeptide gene enhancer in B-cells inhibitor, alpha	-1.7419021	-1.4153091	-1.1368794
NFKBIE	nuclear factor of kappa light polypeptide gene enhancer in B-cells inhibitor, epsilon	-2.4095945	-1.9054573	-5.3194895
NFS1	NFS1 cysteine desulfurase	1.0350393	1.4575802	1.6673054
NFX1	nuclear transcription factor, X-box binding 1	1.5308939	1.4946489	1.8365265
NFYB	nuclear transcription factor Y, beta	5.4581156	3.8463912	2.8711793

NFYC	nuclear transcription factor Y, gamma	1.6410067	1.3593565	-1.0436125
NFYC	nuclear transcription factor Y, gamma	1.3350983	1.6411375	1.3038758
NGEF	neuronal guanine nucleotide exchange factor	-1.4300997	3.1402187	3.442918
NGFRAP1	nerve growth factor receptor (TNFRSF16) associated protein 1	2.692709	2.4608462	2.5985615
NGRN	neugrin, neurite outgrowth associated	-1.9619292	-1.832756	-1.6465976
NHLRC3	NHL repeat containing 3	-1.5217733	-1.2257566	1.7224956
NHP2	NHP2 ribonucleoprotein	1.8271514	1.4982318	1.174985
NID1	nidogen 1	-1.139723	-1.7802416	-2.2692857
NIFK	nucleolar protein interacting with the FHA domain of MKI67	1.524095	1.0646731	-1.0873241
NINJ1	ninjurin 1	1.4926593	1.3411313	1.7068489
NIP7	NIP7, nucleolar pre-rRNA processing protein	2.409407	2.704914	2.6194272
NIPSNAP1	nipsnap homolog 1 (C. elegans)	-1.0552751	1.7298677	2.7809513
NISCH	nischarin	-1.4732678	-1.572029	-1.2457914
NIT1	nitrilase 1	-1.3016645	1.3631296	1.9576949
NIT2	nitrilase family, member 2	1.3135176	1.7715931	2.612591
NKAIN2	Na+/K+ transporting ATPase interacting 2	1.6070517	1.506701	1.1938446
NKAPL	NFKB activating protein-like	2.3290973	2.8497381	2.444934
NKTR	natural killer cell triggering receptor	-1.6723448	-1.6226331	-1.473489
NKTR	natural killer cell triggering receptor	-2.3917427	-2.282309	-2.4913685
NKX3-1	NK3 homeobox 1	-1.0119429	-2.0912347	1.1445462
NLE1	notchless homolog 1 (Drosophila)	1.367106	1.1229109	-1.6303543
NLGN1	neuroligin 1	2.1165338	2.0089877	1.9372528
NLGN2	neuroligin 2	-1.3172547	-1.9177155	-1.6942469
NLRC3	NLR family, CARD domain containing 3	1.555347	1.7253778	2.006559
NLRX1	NLR family member X1	1.0472306	1.2108613	1.7838875
NMB	neuromedin B	1.1872941	1.0097376	-5.109271
NMD3	NMD3 ribosome export adaptor	-1.2004092	-1.4811846	-1.5360559
NME1	NME/NM23 nucleoside diphosphate kinase 1	1.2133062	-1.1559671	-2.1350036
NME2	NME/NM23 nucleoside diphosphate kinase 2	1.0695596	-1.1822842	-1.9351473
NME3	NME/NM23 nucleoside diphosphate kinase 3	-1.071851	1.4092879	1.5375593
NME4	NME/NM23 nucleoside diphosphate kinase 4	1.0721471	-1.3680266	-2.9957287
NMNAT1	nicotinamide nucleotide adenylyltransferase 1	3.3313558	3.747559	3.6802588
NMT1	N-myristoyltransferase 1	1.1273396	1.2484945	1.9020756
NMU	neuromedin U	4.1545563	-1.96816	-1.1469499
NNAT	neuronatin	3.5424788	2.809777	2.4559047
NNMT	nicotinamide N-methyltransferase	1.6251882	2.0428426	-1.0948896
NOBOX	NOBOX oogenesis homeobox	2.5431097	2.3997326	2.2506897
NOL12	nucleolar protein 12	1.0744346	-1.2421783	-1.5553645
NOL3	nucleolar protein 3 (apoptosis repressor with CARD domain)	1.4969093	2.5652807	2.6970184
NOL8	nucleolar protein 8	-1.3590201	-1.8889016	-2.4934957
NOMO1	NODAL modulator 1	-2.0250523	-2.3848188	-3.0807586
NOP16	NOP16 nucleolar protein	1.6606758	1.5334581	-1.2331297
NOP2	NOP2 nucleolar protein	-1.0735078	-1.2598472	-1.8841842
NOP56	NOP56 ribonucleoprotein	1.5418706	-1.1077249	-1.1790684
NOP56	NOP56 ribonucleoprotein	-1.1211118	-1.3752557	-1.8120229
NOP56	NOP56 ribonucleoprotein	1.2492992	-1.3257388	-1.5558395
NOS3	nitric oxide synthase 3 (endothelial cell)	2.0646906	2.0943923	2.070339
NOTCH1	notch 1	1.6927136	1.8910635	1.9352032
NOXA1	NADPH oxidase activator 1	-2.155936	1.2684524	1.5624914
NPC1	Niemann-Pick disease, type C1	-2.3442397	-4.3876634	-3.4092646
NPIPA5	nuclear pore complex interacting protein family, member A5	-2.0611959	-1.9095798	-1.8993313
NPIPB5	nuclear pore complex interacting protein family, member B5	-3.2119286	-2.8001897	-2.6876175
NPIPB5	nuclear pore complex interacting protein family, member B5	-3.028403	-2.7126117	-2.8799736
NPM1	nucleophosmin (nucleolar phosphoprotein B23, numatrin)	1.2010098	-1.146603	-1.7838769
NPM1	nucleophosmin (nucleolar phosphoprotein B23, numatrin)	1.2831547	-1.0911797	-1.6388333
NPM3	nucleophosmin/nucleoplasmin 3	1.5526407	1.2470264	-1.5675576
NPR1	natriuretic peptide receptor 1	2.2234147	3.441881	-1.4504908
NPR3	natriuretic peptide receptor 3	3.3672116	1.7653955	-1.3696939
NPR3	natriuretic peptide receptor 3	5.5558996	3.528511	1.6238471
NQO2	NAD(P)H dehydrogenase, quinone 2	-1.1552776	1.1405053	1.9971952
NR0B2	nuclear receptor subfamily 0, group B, member 2	-6.5240417	2.0794547	15.047624
NR1H3	nuclear receptor subfamily 1, group H, member 3	-1.363126	1.9008656	2.3013954
NR1H4	nuclear receptor subfamily 1, group H, member 4	-3.7119625	2.8458416	2.7931602
NR1I2	nuclear receptor subfamily 1, group I, member 2	-5.8217106	1.8058543	5.516089
NR1I3	nuclear receptor subfamily 1, group I, member 3	-1.3156533	6.136888	65.06505
NR2C2AP	nuclear receptor 2C2-associated protein	-1.3216964	-1.711104	-1.9489939
NR2F1	nuclear receptor subfamily 2, group F, member 1	-2.2466745	-1.4260864	-1.1425012
NR2F2	nuclear receptor subfamily 2, group F, member 2	1.6312397	2.153027	2.0650685
NR2F6	nuclear receptor subfamily 2, group F, member 6	1.4648747	1.7734641	2.6462462
NR5A2	nuclear receptor subfamily 5, group A, member 2	-2.6322348	1.6946851	2.4832044
NRAS	neuroblastoma RAS viral (v-ras) oncogene homolog	-1.2134818	-1.7460835	-1.617974
NRAS	neuroblastoma RAS viral (v-ras) oncogene homolog	-1.028578	-1.5036954	-1.2979479
NRBF2	nuclear receptor binding factor 2	-1.8636206	-1.688885	-1.3659889
NRCAM	neuronal cell adhesion molecule	-1.3166786	-2.1370878	-3.6038735
NREP	neuronal regeneration related protein	1.201654	-1.914656	-1.4398762
NRG1	neuregulin 1	-1.4644065	-1.5872985	-1.6331145
NRM	nurim (nuclear envelope membrane protein)	-1.3008007	-3.2826018	-7.0892434
NRP1	neuropilin 1	-1.2316815	-2.4834197	-3.7743871
NRP1	neuropilin 1	-1.2168254	-2.3827631	-1.7652498
NRTN	neurturin	-1.4287931	1.5404085	2.4863176
NSD1	nuclear receptor binding SET domain protein 1	-1.2685357	-1.9111111	-1.6740085
NSDHL	NAD(P) dependent steroid dehydrogenase-like	2.1800587	2.6525402	3.3648543
NSG1	neuron specific gene family member 1	5.955843	2.7697654	-1.2204876
NSMAF	neutral sphingomyelinase (N-SMase) activation associated factor	-1.1938033	-2.0844986	-2.2215035
NSMCE1	non-SMC element 1 homolog (S. cerevisiae)	1.182385	1.584004	1.6194406
NSUN4	NOP2/Sun domain family, member 4	-1.6109455	-1.5114667	-1.2449476
NSUN5	NOP2/Sun domain family, member 5	-1.3126531	-1.3757924	-1.8268433
NSUN5	NOP2/Sun domain family, member 5	-1.0791734	-1.1636121	-1.5154542
NSUN6	NOP2/Sun domain family, member 6	-1.5238036	1.1110779	1.3488402
NT5C	5', 3'-nucleotidase, cytosolic	1.5210133	1.4732742	1.2038697
NT5C3A	5'-nucleotidase, cytosolic IIIA	-1.5781678	-1.3781924	-1.8436396
NT5C3A	5'-nucleotidase, cytosolic IIIA	-1.5602984	-1.377294	-1.802237
NT5DC1	5'-nucleotidase domain containing 1	1.3444318	1.5360813	1.891683
NT5M	5',3'-nucleotidase, mitochondrial	2.016903	3.0206065	1.7493476
NTAN1	N-terminal asparagine amidase	-1.3418525	-1.5231527	-2.0470788
NTN1	netrin 1	1.4035833	-1.5425028	-2.7461972
NTN4	netrin 4	-1.492819	-5.271299	-5.8304257
NTPCR	nucleoside-triphosphatase, cancer-related	1.217689	1.0777614	-1.5077678
NUAK1	NUAK family, SNF1-like kinase, 1	1.5057374	1.111937	-1.6053575
NUAK2	NUAK family, SNF1-like kinase, 2	1.329332	-2.054776	-3.0268211
NUCB1	nucleobindin 1	-2.0187855	-1.3690625	-1.5541171
NUDCD2	NudC domain containing 2	1.6242585	1.5315723	2.1450984
NUDT1	nudix (nucleoside diphosphate linked moiety X)-type motif 1	1.4538866	-2.1352518	-2.680436
NUDT12	nudix (nucleoside diphosphate linked moiety X)-type motif 12	-1.1546173	1.7578722	1.8929706
NUDT16	nudix (nucleoside diphosphate linked moiety X)-type motif 16	2.445766	5.2776484	10.586234
NUDT16L1	nudix (nucleoside diphosphate linked moiety X)-type motif 16-like 1	1.5163666	1.7636962	2.4679008
NUDT16P1	nudix (nucleoside diphosphate linked moiety X)-type motif 16 pseudogene 1	3.0724008	4.9448495	5.614679
NUDT3	nudix (nucleoside diphosphate linked moiety X)-type motif 3	1.3802916	1.6277335	1.7926414
NUDT7	nudix (nucleoside diphosphate linked moiety X)-type motif 7	1.2781878	2.9907262	5.7453246

NUDT8	nudix (nucleoside diphosphate linked moiety X)-type motif 8	1.3574357	2.344665	2.88802
NUDT8	nudix (nucleoside diphosphate linked moiety X)-type motif 8	2.578663	2.6959984	2.7975245
NUDT9	nudix (nucleoside diphosphate linked moiety X)-type motif 9	1.1342331	1.3631954	2.100972
NUF2	NUF2, NDC80 kinetochore complex component	2.1697042	-2.7326472	-5.021162
NUMBL	numb homolog (Drosophila)-like	1.6906506	1.383572	1.444636
NUP107	nucleoporin 107kDa	1.194965	-1.4250381	-1.5323756
NUP205	nucleoporin 205kDa	-1.5416037	-2.4573162	-2.6982458
NUP37	nucleoporin 37kDa	1.832665	1.2056575	-1.0679022
NUP50	nucleoporin 50kDa	1.5000854	1.1562121	1.0129075
NUP50	nucleoporin 50kDa	1.0806444	-1.3989214	-1.8635851
NUP50-AS1	NUP50 antisense RNA 1 (head to head)	1.9413712	1.4586004	1.5950302
NUP62	nucleoporin 62kDa	1.2281026	-1.4785937	-1.5239968
NUP62	nucleoporin 62kDa	-1.2503356	-2.0191767	-2.9269147
NUP85	nucleoporin 85kDa	-1.0098737	-1.5499741	-1.943427
NUP93	nucleoporin 93kDa	-1.4499748	-3.173522	-4.3041425
NUP98	nucleoporin 98kDa	-1.4821539	-1.6951778	-1.8597405
NUP98	nucleoporin 98kDa	1.6408067	1.004599	-1.0554372
NUPL1	nucleoporin like 1	-1.2246102	-2.3335514	-1.9288356
NUPL2	nucleoporin like 2	2.566158	3.6593847	2.330715
NUPR1L	nuclear protein, transcriptional regulator, 1-like	1.5928203	2.1818528	2.8784754
NUSAP1	nucleolar and spindle associated protein 1	2.3488379	-3.2047887	-2.6473026
NUTF2	nuclear transport factor 2	-1.0199627	-1.6586686	-2.117375
NUTM2A-AS1	NUTM2A antisense RNA 1	1.5223725	1.5067394	-2.2188509
NUTM2F	NUT family member 2F	2.032132	3.2035007	4.643709
NXF1	nuclear RNA export factor 1	-2.0299163	-1.8641179	-1.4993552
NXF2	nuclear RNA export factor 2	-1.3085947	1.2980834	-1.6050917
NXF5	nuclear RNA export factor 5	-1.289391	1.3500901	-1.5842329
NXN	nucleoredoxin	1.1807598	-1.6253892	-1.5039417
NXPH4	neurexophilin 4	-1.0195082	2.9604318	1.6324952
NXT1	nuclear transport factor 2-like export factor 1	1.1587651	-1.4910669	-1.9536629
NYX	nyctalopin	2.362114	2.3097742	2.7246492
OARD1	O-acyl-ADP-ribose deacylase 1	-1.1138546	1.3767622	1.6982584
OAZ2	ornithine decarboxylase antizyme 2	-1.0559982	1.1501987	1.5797501
OBSL1	obscurin-like 1	1.1607454	1.5402403	2.4926455
OCEL1	occludin/ELL domain containing 1	1.087216	2.432917	5.505145
ODF2L	outer dense fiber of sperm tails 2-like	-2.856491	-2.2925758	-1.881359
ODF3B	outer dense fiber of sperm tails 3B	-2.025458	1.0089003	-2.2826562
OGDHL	oxoglutarate dehydrogenase-like	2.0600395	5.0250015	3.8037536
OGFR	opioid growth factor receptor	1.5582063	1.562288	1.7846622
OGFRL1	opioid growth factor receptor-like 1	1.8385122	-1.6098309	1.0338861
OIP5	Opa interacting protein 5	2.7788832	-2.05788	-1.4700041
OIP5-AS1	OIP5 antisense RNA 1	1.623056	1.4828731	2.1466029
OLFML2A	olfactomedin-like 2A	1.0114342	-1.5982558	-11.934977
OLFML3	olfactomedin-like 3	1.1772757	-2.7563672	-12.07306
OMA1	OMA1 zinc metallopeptidase	-1.5822095	-1.1749808	1.0710273
OMD	osteomodulin	30.28025	69.286156	1.5968366
ONECUT1	one cut homeobox 1	-6.7174697	2.4726017	4.8696556
ONECUT2	one cut homeobox 2	-1.6212828	1.2525382	2.2413266
OPN3	opsin 3	1.4695792	1.5522468	2.4283354
OR10H2	olfactory receptor, family 10, subfamily H, member 2	1.6400696	3.0241196	5.3926444
OR11A1	olfactory receptor, family 11, subfamily A, member 1	3.0623522	2.277942	2.093352
OR2H1	olfactory receptor, family 2, subfamily H, member 1	1.8992897	1.7550877	1.948488
ORAI3	ORAI calcium release-activated calcium modulator 3	1.1304551	1.5877584	2.198181
ORC4	origin recognition complex, subunit 4	1.5326712	1.5508918	2.0041912
ORM1	orosomucoid 1	-1.5383923	3.9199524	3.602149
ORM2	orosomucoid 2	-1.5976838	4.300617	3.8707464
ORMDL2	ORMDL sphingolipid biosynthesis regulator 2	1.524993	1.3847275	1.4306041
ORMDL3	ORMDL sphingolipid biosynthesis regulator 3	1.0561459	1.2832382	2.1606035
OS9	osteosarcoma amplified 9, endoplasmic reticulum lectin	-1.5457853	-1.098056	1.2714353
OSBP	oxysterol binding protein	-1.7284006	-1.1249363	1.1158245
OSBPL10	oxysterol binding protein-like 10	1.7101033	-1.285437	-3.2577493
OSBPL11	oxysterol binding protein-like 11	1.0305678	2.1586187	1.8921587
OSBPL1A	oxysterol binding protein-like 1A	2.6472168	3.5369177	3.0240092
OSBPL2	oxysterol binding protein-like 2	-1.7980728	-1.5294229	-1.3539836
OSBPL3	oxysterol binding protein-like 3	1.3048841	-2.2928774	-4.263913
OSBPL3	oxysterol binding protein-like 3	-1.8112706	-4.593018	-5.2964315
OSBPL5	oxysterol binding protein-like 5	2.8411734	2.4930587	1.8317039
OSBPL6	oxysterol binding protein-like 6	-1.1467816	1.4389735	1.897276
OSBPL7	oxysterol binding protein-like 7	1.0151926	-1.4325169	-2.4044197
OSCAR	osteoclast associated, immunoglobulin-like receptor	1.8112676	2.2413354	3.297582
OSGEPL1	O-sialoglycoprotein endopeptidase-like 1	1.0434994	1.2546878	1.6970396
OST4	oligosaccharyltransferase 4 homolog (S. cerevisiae)	1.5440221	1.3458036	1.3682386
OSTF1	osteoclast stimulating factor 1	1.4552473	1.1516268	1.5206164
OTC	ornithine carbamoyltransferase	-5.487248	13.701648	102.102196
OTOF	otoferlin	2.263482	2.324152	2.0609505
OTOS	otospiralin	1.8403752	2.4448073	3.4577084
OTUD6B	OTU domain containing 6B	1.5797421	1.3094578	1.5414425
OTUD6B-AS1	OTUD6B antisense RNA 1 (head to head)	-1.1050814	1.8865938	2.8305154
OTUD7B	OTU deubiquitinase 7B	-1.1807384	1.1514618	1.8400828
OVCA2	ovarian tumor suppressor candidate 2	1.6942486	1.902925	1.7181665
OXER1	oxoeicosanoid (OXE) receptor 1	-1.4897532	1.3238748	4.205455
OXLD1	oxidoreductase-like domain containing 1	1.4222941	1.7929155	1.7665358
OXSM	3-oxoacyl-ACP synthase, mitochondrial	1.8123778	2.3227615	2.341881
OXSR1	oxidative stress responsive 1	1.5189648	1.0040662	1.1419325
OXT	oxytocin/neurophysin I prepropeptide	1.97357	2.0781267	2.4498818
P3H1	prolyl 3-hydroxylase 1	-1.2619791	-2.2685015	-2.1746469
P3H2	prolyl 3-hydroxylase 2	2.5381587	1.807004	-2.059656
P4HA1	prolyl 4-hydroxylase, alpha polypeptide I	1.8554432	-1.1283493	-1.0117123
P4HA2	prolyl 4-hydroxylase, alpha polypeptide II	-2.160186	-4.6640434	-3.0124037
P4HA2	prolyl 4-hydroxylase, alpha polypeptide II	-1.3056972	-3.417144	-1.7591463
PAAF1	proteasomal ATPase-associated factor 1	1.3894116	1.4690343	1.7334044
PAAF1	proteasomal ATPase-associated factor 1	1.3646293	1.4479933	1.8070861
PABPC1	poly(A) binding protein, cytoplasmic 1	-1.1760433	-1.254549	-1.5129775
PABPC1L	poly(A) binding protein, cytoplasmic 1-like	-4.32429	-3.0318594	-4.4992914
PACS1	phosphofurin acidic cluster sorting protein 1	-1.1617208	-1.5050236	-1.9570155
PACS2	phosphofurin acidic cluster sorting protein 2	1.5227813	1.388312	-1.0182718
PACSIN2	protein kinase C and casein kinase substrate in neurons 2	-1.4399606	-2.4869406	-3.116184
PACSIN2	protein kinase C and casein kinase substrate in neurons 2	-1.5465765	-2.2945938	-2.513626
PAF1	Paf1, RNA polymerase II associated factor, homolog (S. cerevisiae)	-1.6631922	-1.720875	-1.4099277
PAFAH1B1	platelet-activating factor acetylhydrolase 1b, regulatory subunit 1 (45kDa)	-1.2157875	-1.716128	-1.0606927
PAFAH1B1	platelet-activating factor acetylhydrolase 1b, regulatory subunit 1 (45kDa)	1.3893905	1.5205125	1.766383
PAFAH1B3	platelet-activating factor acetylhydrolase 1b, catalytic subunit 3 (29kDa)	-1.3069813	-2.1357687	-5.5581217
PAFAH2	platelet-activating factor acetylhydrolase 2, 40kDa	-1.0513654	1.8129923	2.8523412
PAG1	phosphoprotein membrane anchor with glycosphingolipid microdomains 1	-1.3932433	1.637807	1.6337258
PAGE2	P antigen family, member 2 (prostate associated)	1.9720787	1.4625417	-1.3271596
PAGE2B	P antigen family, member 2B	1.8714163	1.3826613	-1.456428
PAGE4	P antigen family, member 4 (prostate associated)	2.5903597	105.92159	49.364613
PAGR1	PAXIP1 associated glutamate-rich protein 1	1.1537902	1.1713716	1.6063305
PAH	phenylalanine hydroxylase	-8.393303	6.086577	41.652924

PAIP1	poly(A) binding protein interacting protein 1	1.622658	1.5392443	1.6509027
PAIP2B	poly(A) binding protein interacting protein 2B	2.1452627	5.023154	7.5204577
PAK1	p21 protein (Cdc42/Rac)-activated kinase 1	-1.4462352	-1.5832613	-1.5447603
PALLD	palladin, cytoskeletal associated protein	1.528973	-1.5095423	-1.7203535
PALLD	palladin, cytoskeletal associated protein	1.4063053	-1.8440468	-1.8355398
PALM	paralemmin	1.2735353	-1.4134474	-2.850336
PALM3	paralemmin 3	1.195406	2.59747	2.9149885
PANK1	pantothenate kinase 1	1.5617838	2.8319833	4.570646
PANK2	pantothenate kinase 2	-1.3473107	-1.6495785	-1.4592139
PANK3	pantothenate kinase 3	1.0569367	1.3018734	1.5654261
PANX1	pannexin 1	-1.1203109	-2.0516422	-1.6746336
PAOX	polyamine oxidase (exo-N4-amino)	1.0425296	1.4399258	1.5083144
PAPLN	papilin, proteoglycan-like sulfated glycoprotein	-1.716907	-1.4727529	-7.1514726
PAPOLA	poly(A) polymerase alpha	-1.4556463	-1.721672	-1.8777118
PAPPA	pregnancy-associated plasma protein A, pappalysin 1	-1.9615877	-4.978483	-26.769804
PAPSS1	3'-phosphoadenosine 5'-phosphosulfate synthase 1	-1.0555805	-1.4303974	-1.8190961
PAPSS2	3'-phosphoadenosine 5'-phosphosulfate synthase 2	1.2426691	2.835415	1.5795631
PAQR3	progestin and adipoQ receptor family member III	1.4750226	1.0912752	1.9671533
PAQR5	progestin and adipoQ receptor family member V	-1.2857982	1.7923567	-3.471161
PAQR9	progestin and adipoQ receptor family member IX	-2.6378875	2.00533	8.593299
PARD3	par-3 family cell polarity regulator	-1.7438252	-1.5292848	-1.3938856
PARD6B	par-6 family cell polarity regulator beta	-1.3802346	1.5368567	1.6173563
PARD6G-AS1	PARD6G antisense RNA 1	1.19531	1.5370276	1.6543334
PARP12	poly (ADP-ribose) polymerase family, member 12	-1.4948068	-1.7117715	-2.020838
PARP14	poly (ADP-ribose) polymerase family, member 14	-3.3810394	-2.5315552	-2.9517279
PARP6	poly (ADP-ribose) polymerase family, member 6	-1.5463624	-1.3227627	-1.4382651
PARPBP	PARP1 binding protein	1.3738711	-2.8965008	-2.8512485
PARS2	prolyl-tRNA synthetase 2, mitochondrial (putative)	1.2998192	1.7877983	1.7437959
PARVA	parvin, alpha	1.7925667	1.1717594	1.3271915
PARVB	parvin, beta	-1.3317175	-1.3598408	-2.547933
PAWR	PRKC, apoptosis, WT1, regulator	1.6278791	-1.0657296	-1.2754135
PAXIP1-AS2	PAXIP1 antisense RNA 2	-1.2365557	1.0882986	1.7626085
PBK	PDZ binding kinase	2.256534	-3.1419961	-2.991727
PBLD	phenazine biosynthesis-like protein domain containing	1.0422016	1.7254323	2.1774812
PBRM1	polybromo 1	-1.069772	-1.6135902	-1.5318183
PBX3	pre-B-cell leukemia homeobox 3	-1.6552913	-1.7632596	-1.4966074
PC	pyruvate carboxylase	-1.055979	2.002386	2.6907632
PCAT19	prostate cancer associated transcript 19 (non-protein coding)	1.2713147	1.2639045	1.5507158
PCBD2	pterin-4 alpha-carbinolamine dehydratase/dimerization cofactor of hepatocyte nuclear factor 1	1.0201503	1.0797694	1.7759415
PCBP4	poly(rC) binding protein 4	-1.0602655	-1.1065849	-2.4096215
PCBP4	poly(rC) binding protein 4	1.12591	-1.0527899	-2.1617641
PCCA	propionyl CoA carboxylase, alpha polypeptide	-1.7018524	1.8268688	3.070104
PCCB	propionyl CoA carboxylase, beta polypeptide	-1.2820411	1.2545097	2.2551773
PCDH1	protocadherin 1	-2.6280894	1.1644615	-1.1379052
PCDH7	protocadherin 7	2.0436034	1.0506024	-3.2245872
PCDHB11	protocadherin beta 11	-1.4805335	-1.6236995	-1.5709549
PCDHB14	protocadherin beta 14	-1.7539325	-1.5528336	-1.2861704
PCDHB16	protocadherin beta 16	-1.9795173	-2.5289547	-2.3908446
PCDHGA7	protocadherin gamma subfamily A, 7	1.8621813	1.9988949	2.1495793
PCDHGA8	protocadherin gamma subfamily A, 8	-1.4596038	-1.9845337	-1.5862699
PCGF5	polycomb group ring finger 5	1.2923363	1.7521031	1.8818753
PCGF5	polycomb group ring finger 5	1.8487864	2.1444252	1.992011
PCK2	phosphoenolpyruvate carboxykinase 2 (mitochondrial)	-2.4754987	2.6397252	9.718252
PCK2	phosphoenolpyruvate carboxykinase 2 (mitochondrial)	-1.3792955	2.3126576	6.966567
PCM1	pericentriolar material 1	-1.8041344	-1.7855747	-2.0771887
PCMT1	protein-L-isoaspartate (D-aspartate) O-methyltransferase	-1.3489195	-1.7219875	-1.3651136
PCNA	proliferating cell nuclear antigen	1.3915578	-2.2779436	-2.6156142
PCNA	proliferating cell nuclear antigen	1.4366482	-2.1528585	-2.4196086
PCNA	proliferating cell nuclear antigen	1.1621205	-2.8120232	-3.4137478
PCNT	pericentrin	-1.8054978	-2.2991006	-2.2584286
PCNXL4	pecanex-like 4 (Drosophila)	-1.1071264	-1.2877907	-1.7259711
PCNXL4	pecanex-like 4 (Drosophila)	2.2471416	1.2680235	-1.0646769
PCOLCE	procollagen C-endopeptidase enhancer	2.9665873	2.418522	-3.9449942
PCSK4	proprotein convertase subtilisin/kexin type 4	-1.6034908	1.2174288	-1.3930311
PCSK5	proprotein convertase subtilisin/kexin type 5	-1.6896721	-3.2714934	-4.5329175
PCSK6	proprotein convertase subtilisin/kexin type 6	-1.1338714	2.870091	3.865807
PCSK6	proprotein convertase subtilisin/kexin type 6	-1.0569072	2.1104236	2.6110783
PCSK7	proprotein convertase subtilisin/kexin type 7	-1.0992601	-1.4227651	-1.7094992
PCSK9	proprotein convertase subtilisin/kexin type 9	2.7055395	1.405801	2.1978054
PCTP	phosphatidylcholine transfer protein	1.9536239	2.9004881	4.273812
PCYOX1	prenylcysteine oxidase 1	1.032642	1.6604654	3.070298
PCYOX1	prenylcysteine oxidase 1	-1.2638544	1.3908597	2.5464075
PCYT2	phosphate cytidylyltransferase 2, ethanolamine	1.46609	2.4833002	4.213048
PCYT2	phosphate cytidylyltransferase 2, ethanolamine	1.230094	2.2910454	3.3823218
PCYT2	phosphate cytidylyltransferase 2, ethanolamine	1.1763612	2.1966856	3.2564483
PDCD4-AS1	PDCD4 antisense RNA 1	2.1714816	1.3563311	1.0992537
PDCD5	programmed cell death 5	1.6402268	1.4252048	1.0740098
PDCD6	programmed cell death 6	1.5527941	1.600518	1.530243
PDCD6IP	programmed cell death 6 interacting protein	-1.7992134	-1.9349276	-1.814447
PDCD7	programmed cell death 7	-1.4116126	-1.8146054	-1.7091007
PDCL3	phosducin-like 3	1.1757905	-1.3898783	-1.9380255
PDDC1	Parkinson disease 7 domain containing 1	1.3172674	1.1469067	-1.715444
PDE12	phosphodiesterase 12	1.5805784	1.3961998	1.6920699
PDE1C	phosphodiesterase 1C, calmodulin-dependent 70kDa	1.1754282	-1.9864054	-3.5841558
PDE4A	phosphodiesterase 4A, cAMP-specific	1.329948	1.0173856	-2.174459
PDE4DIP	phosphodiesterase 4D interacting protein	-1.370601	1.1621073	1.6457615
PDE4DIP	phosphodiesterase 4D interacting protein	2.1694756	5.161437	9.534468
PDE4DIP	phosphodiesterase 4D interacting protein	1.4343282	3.3262637	5.458485
PDE5A	phosphodiesterase 5A, cGMP-specific	2.0845277	-1.0203015	-3.2890253
PDE6G	phosphodiesterase 6G, cGMP-specific, rod, gamma	2.8772483	3.439498	1.9273531
PDE8B	phosphodiesterase 8B	3.1039636	13.500586	9.019712
PDHA1	pyruvate dehydrogenase (lipoamide) alpha 1	1.666971	1.8705868	1.4120598
PDHB	pyruvate dehydrogenase (lipoamide) beta	1.6726335	1.8412104	2.1377244
PDIA6	protein disulfide isomerase family A, member 6	-1.6800356	-2.1878116	-2.6233656
PDIK1L	PDLIM1 interacting kinase 1 like	1.374793	1.872762	4.116185
PDK2	pyruvate dehydrogenase kinase, isozyme 2	-1.2303929	1.9252774	1.590831
PDK4	pyruvate dehydrogenase kinase, isozyme 4	-11.042562	-5.643052	-2.7543752
PDLIM5	PDZ and LIM domain 5	-1.44435	-1.5900346	-1.7392702
PDLIM7	PDZ and LIM domain 7 (enigma)	-2.4443944	-11.303598	-31.324926
PDLIM7	PDZ and LIM domain 7 (enigma)	1.1097139	-3.0817	-2.9126465
PDLIM7	PDZ and LIM domain 7 (enigma)	-2.8230207	-9.4290285	-26.312956
PDP2	pyruvate dehyrogenase phosphatase catalytic subunit 2	1.2286301	1.2487586	1.8773028
PDPK1	3-phosphoinositide dependent protein kinase 1	-1.1027669	1.141964	1.5817282
PDPN	podoplanin	3.1149948	2.273959	-1.9791857
PDPR	pyruvate dehydrogenase phosphatase regulatory subunit	-1.3755329	-1.2857653	-1.6917474
PDSS1	prenyl (decaprenyl) diphosphate synthase, subunit 1	1.7279354	1.4179425	-1.5736322
PDSS2	prenyl (decaprenyl) diphosphate synthase, subunit 2	-1.3331616	1.2681086	1.5108316
PDSS2	prenyl (decaprenyl) diphosphate synthase, subunit 2	-1.1138505	1.3264046	1.9367951
PDXDC1	pyridoxal-dependent decarboxylase domain containing 1	-1.0162921	1.2213731	2.028408

PDXP	pyridoxal (pyridoxine, vitamin B6) phosphatase	1.2593639	1.8058293	4.4041367
PDZD11	PDZ domain containing 11	-1.2674472	-1.344582	-1.6326469
PDZD8	PDZ domain containing 8	-1.6746393	-1.2857244	-1.1018846
PDZK1	PDZ domain containing 1	1.3689364	5.2450514	4.793864
PDZK1IP1	PDZK1 interacting protein 1	1.7161785	4.7728076	1.2330457
PEA15	phosphoprotein enriched in astrocytes 15	1.3246611	-1.9948847	-2.4958327
PEA15	phosphoprotein enriched in astrocytes 15	1.1745094	-2.2850578	-2.618362
PEAK1	pseudopodium-enriched atypical kinase 1	-1.4302047	-1.3830887	-1.8093941
PEBP1	phosphatidylethanolamine binding protein 1	1.3189111	2.6897	5.7279673
PECR	peroxisomal trans-2-enoyl-CoA reductase	-1.0353254	3.2678216	5.1321883
PEG10	paternally expressed 10	1.7594001	2.9344394	9.188355
PELI1	pellino E3 ubiquitin protein ligase 1	-1.9684349	-1.2623072	1.1038096
PELI3	pellino E3 ubiquitin protein ligase family member 3	1.3053046	-1.1124607	-2.0711815
PEPD	peptidase D	1.0373676	2.3417966	3.750346
PER2	period circadian clock 2	-3.3367589	-1.8414313	-4.176274
PES1	pescadillo ribosomal biogenesis factor 1	-1.3451939	-1.3095413	-1.844408
PEX11A	peroxisomal biogenesis factor 11 alpha	-1.5031807	1.1931443	2.0955513
PEX11B	peroxisomal biogenesis factor 11 beta	1.6483406	1.7704343	2.0456553
PEX11G	peroxisomal biogenesis factor 11 gamma	1.280314	2.5089698	3.747078
PEX12	peroxisomal biogenesis factor 12	1.3513315	1.8672757	1.9668204
PEX14	peroxisomal biogenesis factor 14	1.6720082	1.4469763	2.1485243
PEX16	peroxisomal biogenesis factor 16	1.057555	1.4664282	1.647053
PEX7	peroxisomal biogenesis factor 7	1.1704739	1.3531641	2.0160396
PFDN1	prefoldin subunit 1	-1.1132878	-1.2761544	-1.5717275
PFDN4	prefoldin subunit 4	-1.1178035	-1.9705222	-2.1202488
PFKFB2	6-phosphofructo-2-kinase/fructose-2,6-biphosphatase 2	1.2176473	1.2416967	-1.6319745
PFKL	phosphofructokinase, liver	2.1585462	2.761227	3.0091584
PFKP	phosphofructokinase, platelet	-1.9483517	-4.950847	-8.193542
PFKP	phosphofructokinase, platelet	-1.8714963	-5.3911676	-8.771774
PFN1P2	profilin 1 pseudogene 2	2.7834187	1.7671856	1.9743298
PGA4	pepsinogen 4, group I (pepsinogen A)	-1.579075	10.500438	25.64097
PGAM1	phosphoglycerate mutase 1 (brain)	1.9221683	1.5818888	1.3860418
PGAM4	phosphoglycerate mutase family member 4	2.2237113	1.9988496	1.8909849
PGAP2	post-GPI attachment to proteins 2	-1.074276	1.141851	1.5441847
PGAP3	post-GPI attachment to proteins 3	1.0581552	1.2823037	1.5631652
PGK1	phosphoglycerate kinase 1	1.760669	1.6603545	1.4051056
PGM1	phosphoglucomutase 1	1.2511903	1.4986944	1.8623271
PGM2	phosphoglucomutase 2	1.8883833	1.4142772	2.2295196
PGM2L1	phosphoglucomutase 2-like 1	-3.1010554	-5.7157154	-8.897013
PGP	phosphoglycolate phosphatase	1.1056631	3.182873	4.5043526
PGPEP1	pyroglutamyl-peptidase I	-2.6662786	-1.5354977	-1.2580527
PGRMC1	progesterone receptor membrane component 1	1.2735727	1.6732432	2.5320587
PHACTR2	phosphatase and actin regulator 2	-1.6211013	-1.9235823	-2.965444
PHACTR2	phosphatase and actin regulator 2	1.690677	1.171913	-1.2961937
PHF1	PHD finger protein 1	-1.6821414	-1.6162884	-2.6456
PHF11	PHD finger protein 11	-1.4873599	-1.8408332	-2.5633347
PHF13	PHD finger protein 13	-1.1008879	-1.4076785	-1.7972875
PHF14	PHD finger protein 14	-1.599914	-1.5252177	-1.4336871
PHF19	PHD finger protein 19	1.4791123	-2.9174294	-4.1081753
PHF19	PHD finger protein 19	1.6455864	-3.9815476	-10.70341
PHF6	PHD finger protein 6	-1.0348523	-1.4606259	-1.5992326
PHGDH	phosphoglycerate dehydrogenase	-2.013099	-4.212573	-2.0511005
PHIP	pleckstrin homology domain interacting protein	-1.5789326	-1.5431523	-1.724577
PHIP	pleckstrin homology domain interacting protein	-1.555025	-1.4958854	-1.5679784
PHKA2	phosphorylase kinase, alpha 2 (liver)	-1.6251829	-1.061158	1.2229148
PHLDA2	pleckstrin homology-like domain, family A, member 2	1.1668006	-1.6083312	-3.1463718
PHLDA3	pleckstrin homology-like domain, family A, member 3	1.4661925	-1.1136959	-2.1903512
PHLDB1	pleckstrin homology-like domain, family B, member 1	-1.4899408	-3.0421276	-6.1731367
PHOSPHO1	phosphatase, orphan 1	2.4828482	2.8674622	3.970728
PHPT1	phosphohistidine phosphatase 1	1.3939072	1.0474961	-1.5999792
PHRF1	PHD and ring finger domains 1	1.1251417	1.2948273	1.5399873
PHTF1	putative homeodomain transcription factor 1	1.2887925	-1.0147514	-1.9060564
PHTF2	putative homeodomain transcription factor 2	1.4332147	-1.9256092	-1.750679
PHYH	phytanoyl-CoA 2-hydroxylase	1.0813915	1.4213095	2.2868636
PI4K2B	phosphatidylinositol 4-kinase type 2 beta	1.0628967	1.2101622	3.0048056
PI4KA	phosphatidylinositol 4-kinase, catalytic, alpha	-1.6529663	-1.6363552	-2.4976997
PI4KA	phosphatidylinositol 4-kinase, catalytic, alpha	-1.9027123	-1.9034173	-2.589836
PIAS2	protein inhibitor of activated STAT, 2	1.0975306	-1.533376	-1.3283012
PIAS3	protein inhibitor of activated STAT, 3	1.4582487	-1.3374213	-1.8253196
PID1	phosphotyrosine interaction domain containing 1	-1.4961843	2.1386156	3.1573238
PIDD1	p53-induced death domain protein 1	-1.5268523	-2.3996801	-2.7615726
PIEZO1	piezo-type mechanosensitive ion channel component 1	-2.0162578	-4.2888823	-5.5607643
PIEZO2	piezo-type mechanosensitive ion channel component 2	-1.4165552	-1.4424638	-1.6900774
PIF1	PIF1 5'-to-3' DNA helicase	3.6520905	-2.098983	-2.052933
PIGG	phosphatidylinositol glycan anchor biosynthesis, class G	-1.3646996	-1.4800555	-1.6515435
PIGH	phosphatidylinositol glycan anchor biosynthesis, class H	-1.1383388	1.0967904	1.8499688
PIGL	phosphatidylinositol glycan anchor biosynthesis, class L	-1.1827103	1.1697061	2.6185925
PIGP	phosphatidylinositol glycan anchor biosynthesis, class P	1.3800371	1.6326591	1.9889374
PIGS	phosphatidylinositol glycan anchor biosynthesis, class S	1.7633542	1.3820103	1.119792
PIK3R1	phosphoinositide-3-kinase, regulatory subunit 1 (alpha)	2.0052624	4.257456	7.1704583
PILRA	paired immunoglobin-like type 2 receptor alpha	-2.7923527	-1.8448014	-1.6608613
PILRB	paired immunoglobin-like type 2 receptor beta	-2.5959234	-1.8871127	-1.5459522
PIM3	Pim-3 proto-oncogene, serine/threonine kinase	2.432539	3.0683641	4.463094
PIN4	protein (peptidylprolyl cis/trans isomerase) NIMA-interacting, 4 (parvulin)	1.4126879	1.300645	1.9940847
PINK1	PTEN induced putative kinase 1	-1.1946802	-1.2546368	1.8725832
PIPOX	pipecolic acid oxidase	-1.5424618	3.6901793	13.566608
PITPNA	phosphatidylinositol transfer protein, alpha	1.4003682	1.366441	1.7953546
PITPNM3	PITPNM family member 3	-1.4133931	-2.829297	-21.275103
PITRM1	pitrilysin metallopeptidase 1	-1.9898778	-1.9077673	-2.2587306
PITX3	paired-like homeodomain 3	1.4489138	2.3531559	3.5272021
PKD1	polycystic kidney disease 1 (autosomal dominant)	1.8520786	2.2439425	2.5825012
PKLR	pyruvate kinase, liver and RBC	-1.2489556	3.2323418	11.099814
PKM	pyruvate kinase, muscle	-1.2096639	-1.9375149	-4.213927
PKMYT1	protein kinase, membrane associated tyrosine/threonine 1	1.7470462	-3.1885424	-3.4705145
PKNOX1	PBX/knotted 1 homeobox 1	1.0369251	1.1243125	1.5131727
PKP2	plakophilin 2	2.8970082	3.8449671	4.4439545
PKP4	plakophilin 4	-1.7496307	-1.7559057	-1.9128518
PKP4	plakophilin 4	-1.5409828	-1.5684304	-1.7756897
PLA1A	phospholipase A1 member A	-5.471059	-1.6063488	-10.05268
PLA2G12A	phospholipase A2, group XIIA	1.0846312	1.4277679	1.873218
PLA2G12B	phospholipase A2, group XIIB	-4.892796	8.742494	32.963142
PLA2G15	phospholipase A2, group XV	-1.0470755	-1.1901064	2.3107126
PLA2G16	phospholipase A2, group XVI	1.7122091	2.8664227	6.684782
PLA2G1B	phospholipase A2, group IB (pancreas)	-2.0545082	7.3134036	4.8704143
PLA2G4C	phospholipase A2, group IVC (cytosolic, calcium-independent)	-2.6006172	1.2635719	-1.1937554
PLAA	phospholipase A2-activating protein	-1.0854944	-1.4252665	-1.5533848
PLAC8	placenta-specific 8	37.789623	21.29378	3.6794817
PLCB1	phospholipase C, beta 1 (phosphoinositide-specific)	-3.026305	-1.6372701	-1.4951974
PLCL1	phospholipase C-like 1	5.2159257	6.662147	15.317916

PLCL2	phospholipase C-like 2	1.2341083	1.6967714	1.71561
PLCXD1	phosphatidylinositol-specific phospholipase C, X domain containing 1	2.7587547	11.616483	6.709994
PLCXD3	phosphatidylinositol-specific phospholipase C, X domain containing 3	1.0724467	2.9381764	2.8226578
PLEK2	pleckstrin 2	-1.5368282	-3.2008886	-11.094903
PLEKHA1	pleckstrin homology domain containing, family A (phosphoinositide binding specific) member	-1.8397211	-1.7489614	-1.5462414
PLEKHA2	pleckstrin homology domain containing, family A (phosphoinositide binding specific) member	2.8301542	1.6431035	1.0789928
PLEKHA3	pleckstrin homology domain containing, family A (phosphoinositide binding specific) member	1.8532499	1.2363058	-1.0284345
PLEKHA4	pleckstrin homology domain containing, family A (phosphoinositide binding specific) member	-1.6373489	-1.0584136	-5.046332
PLEKHA6	pleckstrin homology domain containing, family A member 6	1.0580244	1.3826396	-1.5552521
PLEKHB2	pleckstrin homology domain containing, family B (evectins) member 2	1.2367369	1.0531105	-1.5317777
PLEKHF1	pleckstrin homology domain containing, family F (with FYVE domain) member 1	-1.2648253	1.9012835	3.1609209
PLEKHF2	pleckstrin homology domain containing, family F (with FYVE domain) member 2	1.5749843	2.027221	1.9872956
PLEKHG2	pleckstrin homology domain containing, family G (with RhoGef domain) member 2	-1.5200347	-3.2283664	-19.986744
PLEKHG4	pleckstrin homology domain containing, family G (with RhoGef domain) member 4	2.7225823	2.0884933	-5.1294065
PLEKHH3	pleckstrin homology domain containing, family H (with MyTH4 domain) member 3	1.0486041	1.2755177	1.67546
PLEKHO1	pleckstrin homology domain containing, family O member 1	-1.7767519	-7.265096	-12.847448
PLG	plasminogen	-3.0267675	19.199827	508.2388
PLGLB1	plasminogen-like B1	-2.7495143	2.8860693	4.0650764
PLGLB1	plasminogen-like B1	-17.646204	3.1947963	7.757507
PLGLB1	plasminogen-like B1	-10.627792	2.451696	3.5243068
PLIN3	perilipin 3	-1.2567813	-2.3856645	-5.616717
PLIN4	perilipin 4	-1.5457199	-1.4436625	-3.43849
PLK2	polo-like kinase 2	-1.1769615	-3.7316601	-5.046314
PLK3	polo-like kinase 3	-1.5571725	-3.405448	-5.045188
PLOD1	procollagen-lysine, 2-oxoglutarate 5-dioxygenase 1	-1.6538473	-2.0632832	-1.4709932
PLOD2	procollagen-lysine, 2-oxoglutarate 5-dioxygenase 2	1.3697802	-2.457064	-2.7703412
PLOD3	procollagen-lysine, 2-oxoglutarate 5-dioxygenase 3	-1.155388	-1.3281342	-1.8338645
PLP2	proteolipid protein 2 (colonic epithelium-enriched)	-1.1128616	-1.8849796	-6.3062687
PLS1	plastin 1	-1.6287426	1.3438448	2.5968556
PLSCR4	phospholipid scramblase 4	1.0930991	1.3444673	2.735998
PLXDC2	plexin domain containing 2	1.1194762	-1.6593773	-1.3134673
PLXNA1	plexin A1	-1.2666798	-2.8465202	-3.8418725
PLXNA3	plexin A3	-1.6859504	-1.541896	-2.1542404
PLXNB1	plexin B1	-1.51756	-1.1676111	-2.0070589
PLXNB2	plexin B2	-1.4766731	-1.7041801	-2.113729
PLXNB2	plexin B2	-1.6720632	-2.1188736	-2.489339
PMAIP1	phorbol-12-myristate-13-acetate-induced protein 1	-2.7930872	-10.120496	-12.663573
PMEPA1	prostate transmembrane protein, androgen induced 1	-1.1901872	-5.108514	-12.600315
PMEPA1	prostate transmembrane protein, androgen induced 1	-1.7396852	-5.7952404	-11.697295
PMF1	polyamine-modulated factor 1	-1.6746416	-1.6211259	-1.7860116
PML	promyelocytic leukemia	1.5794276	-1.8920286	-3.158414
PML	promyelocytic leukemia	1.3195136	-1.0427458	-3.242212
PMM1	phosphomannomutase 1	1.0698372	2.1554682	2.2791228
PMVK	phosphomevalonate kinase	1.1469171	1.6170651	1.9684097
PNKD	paroxysmal nonkinesigenic dyskinesia	1.6231065	1.6284318	1.3843827
PNKP	polynucleotide kinase 3'-phosphatase	-1.4685799	-1.7843496	-2.257963
PNPLA2	patatin-like phospholipase domain containing 2	1.8095659	2.0600173	2.696014
PNPLA3	patatin-like phospholipase domain containing 3	1.0147148	1.1383337	2.450563
PNPLA4	patatin-like phospholipase domain containing 4	1.8050977	3.1874647	3.3677223
PNPLA7	patatin-like phospholipase domain containing 7	-1.8138047	1.6625282	1.8040607
PNPO	pyridoxamine 5'-phosphate oxidase	1.4960618	1.7052927	2.4410977
PNPT1	polyribonucleotide nucleotidyltransferase 1	1.1120685	-1.3480481	-1.7088214
PNRC1	proline-rich nuclear receptor coactivator 1	-1.8426205	-1.1854345	-1.1675556
PNRC1	proline-rich nuclear receptor coactivator 1	-1.5854262	1.0628854	1.0129654
PNRC2	proline-rich nuclear receptor coactivator 2	-1.453732	-1.9526119	-1.6052706
POC1A	POC1 centriolar protein A	1.461975	-2.5781868	-2.53403
PODXL	podocalyxin-like	-2.5191948	-4.4141235	-2.2732656
POGK	pogo transposable element with KRAB domain	-1.0403038	-1.2742171	-1.7671385
POLA1	polymerase (DNA directed), alpha 1, catalytic subunit	1.1246635	-1.8022358	-2.531888
POLD1	polymerase (DNA directed), delta 1, catalytic subunit	1.1302698	-1.9326186	-2.0996237
POLD3	polymerase (DNA-directed), delta 3, accessory subunit	-1.1247964	-2.7503676	-2.9340112
POLD4	polymerase (DNA-directed), delta 4, accessory subunit	-1.0833741	1.4283897	1.8707571
POLDIP2	polymerase (DNA-directed), delta interacting protein 2	1.1829185	1.6503159	2.1379097
POLE3	polymerase (DNA directed), epsilon 3, accessory subunit	1.2129617	-1.59164	-1.2226807
POLE4	polymerase (DNA-directed), epsilon 4, accessory subunit	-1.190297	-1.6206938	-1.167133
POLH	polymerase (DNA directed), eta	1.2090868	-1.3012636	-2.6282196
POLM	polymerase (DNA directed), mu	1.262433	1.996625	1.6511543
POLM	polymerase (DNA directed), mu	1.6574422	2.2226057	2.2994347
POLR1B	polymerase (RNA) I polypeptide B, 128kDa	1.6632779	1.8000814	1.3466134
POLR1E	polymerase (RNA) I polypeptide E, 53kDa	1.5894457	1.083962	-1.0252132
POLR2D	polymerase (RNA) II (DNA directed) polypeptide D	-1.1511481	-1.5375184	-1.4135982
POLR2E	polymerase (RNA) II (DNA directed) polypeptide E, 25kDa	1.823181	1.5867726	2.1372507
POLR2E	polymerase (RNA) II (DNA directed) polypeptide E, 25kDa	1.4507422	1.6360513	3.486433
POLR2F	polymerase (RNA) II (DNA directed) polypeptide F	1.5839225	1.2209747	1.6317849
POLR2F	polymerase (RNA) II (DNA directed) polypeptide F	2.5145361	2.4214473	2.5033073
POLR2G	polymerase (RNA) II (DNA directed) polypeptide G	1.3343707	1.4803586	1.685661
POLR2H	polymerase (RNA) II (DNA directed) polypeptide H	1.5930599	1.3715913	1.5780513
POLR2H	polymerase (RNA) II (DNA directed) polypeptide H	3.6328347	4.5743575	4.851619
POLR2J2	polymerase (RNA) II (DNA directed) polypeptide J2	2.721537	2.9022582	2.8819284
POLR2L	polymerase (RNA) II (DNA directed) polypeptide L, 7.6kDa	1.9653053	2.0276754	2.292392
POLR3E	polymerase (RNA) III (DNA directed) polypeptide E (80kD)	1.3228959	1.2653683	1.5286597
POLR3GL	polymerase (RNA) III (DNA directed) polypeptide G (32kD)-like	1.190436	1.2058896	1.9536316
POLR3GL	polymerase (RNA) III (DNA directed) polypeptide G (32kD)-like	1.2202854	1.2695031	2.0314555
POLR3K	polymerase (RNA) III (DNA directed) polypeptide K, 12.3 kDa	1.8167065	1.3886536	1.1046588
POMT1	protein-O-mannosyltransferase 1	1.181907	1.2749876	1.8715316
POMT2	protein-O-mannosyltransferase 2	-1.6923574	-2.1658998	-2.4833927
PON1	paraoxonase 1	-2.71315	4.787054	8.302191
PON2	paraoxonase 2	-1.582358	-2.0639827	-2.541839
PON3	paraoxonase 3	-1.7308103	3.021851	5.0314455
POP1	processing of precursor 1, ribonuclease P/MRP subunit (S. cerevisiae)	1.8198558	-1.1495138	-2.1786783
POP4	processing of precursor 4, ribonuclease P/MRP subunit (S. cerevisiae)	1.1812284	1.2062428	1.5493693
POP5	processing of precursor 5, ribonuclease P/MRP subunit (S. cerevisiae)	2.2416174	2.4929128	3.2861736
POP7	processing of precursor 7, ribonuclease P/MRP subunit (S. cerevisiae)	1.6040232	1.2585338	1.2406139
POR	P450 (cytochrome) oxidoreductase	-2.1341217	1.0039061	2.905242
PORCN	porcupine homolog (Drosophila)	1.2991611	-1.9869474	-2.8396454
POSTN	periostin, osteoblast specific factor	1.058071	-31.010681	-191.16913
POTEF	POTE ankyrin domain family, member F	-1.1971134	-2.0221453	-3.0347505
POTEI	POTE ankyrin domain family, member I	1.4014336	-1.513217	-2.069036
POTEKP	POTE ankyrin domain family, member K, pseudogene	1.5698409	-1.2893163	-1.8099872
POTEM	POTE ankyrin domain family, member M	1.1235296	-1.8117287	-2.470253
POU3F1	POU class 3 homeobox 1	1.5923773	1.186819	1.3743517
PPA2	pyrophosphatase (inorganic) 2	1.4121262	1.6161472	1.8725467
PPAP2B	phosphatidic acid phosphatase type 2B	-1.2618858	1.8752512	1.4952475
PPAPDC1B	phosphatidic acid phosphatase type 2 domain containing 1B	-1.8893771	-1.9955788	-2.2952611
PPAPDC1B	phosphatidic acid phosphatase type 2 domain containing 1B	-2.5591145	-2.0395973	-3.5181243
PPARA	peroxisome proliferator-activated receptor alpha	-1.1910479	1.6742792	3.2591665
PPARD	peroxisome proliferator-activated receptor delta	-1.5154196	-1.4496768	1.2557163
PPARG	peroxisome proliferator-activated receptor gamma	-1.2143391	-1.821094	-3.20899
PPARG	peroxisome proliferator-activated receptor gamma	-2.4901302	-3.8671827	-5.947599

PPARGC1A	peroxisome proliferator-activated receptor gamma, coactivator 1 alpha	2.117482	2.473133	1.4479969
PPBP	pro-platelet basic protein (chemokine (C-X-C motif) ligand 7)	12.366824	-1.2363647	-13.1988
PPDPF	pancreatic progenitor cell differentiation and proliferation factor	1.9897119	2.102543	2.5324569
PPFIBP1	PTPRF interacting protein, binding protein 1 (liprin beta 1)	-1.2994043	-1.5737629	-2.2904403
PPHLN1	periphilin 1	1.5474494	1.0781308	1.2847273
PPIF	peptidylprolyl isomerase F	1.7302759	1.6842782	1.6942848
PPIH	peptidylprolyl isomerase H (cyclophilin H)	1.4171938	-1.2251121	-1.5938781
PPL	periplakin	1.6413764	2.6641417	4.495278
PPM1B	protein phosphatase, Mg2+/Mn2+ dependent, 1B	1.7270341	1.9085584	2.390709
PPM1D	protein phosphatase, Mg2+/Mn2+ dependent, 1D	1.7447498	1.0407171	-1.0192946
PPM1D	protein phosphatase, Mg2+/Mn2+ dependent, 1D	1.0433803	-1.6197847	-1.8705052
PPM1F	protein phosphatase, Mg2+/Mn2+ dependent, 1F	1.1636245	-1.3689017	-1.8035953
PPP1CB	protein phosphatase 1, catalytic subunit, beta isozyme	1.5293251	1.526576	-1.4118173
PPP1R13L	protein phosphatase 1, regulatory subunit 13 like	1.0531585	-1.0958421	-2.7317011
PPP1R14A	protein phosphatase 1, regulatory (inhibitor) subunit 14A	3.886972	4.5912557	5.388136
PPP1R14B	protein phosphatase 1, regulatory (inhibitor) subunit 14B	1.2819388	-1.1209797	-2.320082
PPP1R15A	protein phosphatase 1, regulatory subunit 15A	-3.128669	-5.374864	-6.7616463
PPP1R16A	protein phosphatase 1, regulatory subunit 16A	1.291341	3.3438528	6.1528006
PPP1R16B	protein phosphatase 1, regulatory subunit 16B	2.5704741	2.749508	3.1551428
PPP1R18	protein phosphatase 1, regulatory subunit 18	-1.1080756	-2.0222685	-5.818918
PPP1R1C	protein phosphatase 1, regulatory (inhibitor) subunit 1C	-1.2171595	1.5121038	1.3316059
PPP1R2	protein phosphatase 1, regulatory (inhibitor) subunit 2	-1.1496384	-1.6929617	-1.5216793
PPP1R3B	protein phosphatase 1, regulatory subunit 3B	1.6023629	4.170734	8.733561
PPP1R3C	protein phosphatase 1, regulatory subunit 3C	1.829521	6.139836	9.890606
PPP1R8	protein phosphatase 1, regulatory subunit 8	1.570424	1.2025388	1.4192661
PPP1R9B	protein phosphatase 1, regulatory subunit 9B	-1.1060655	-1.1288857	-1.6575216
PPP2R1B	protein phosphatase 2, regulatory subunit A, beta	-1.2166907	1.494624	-1.7280895
PPP2R2D	protein phosphatase 2, regulatory subunit B, delta	-1.3968389	-1.5146503	-1.5024205
PPP2R5A	protein phosphatase 2, regulatory subunit B', alpha	-1.4042743	1.3374991	2.0277715
PPP3CA	protein phosphatase 3, catalytic subunit, alpha isozyme	1.0801668	-1.4518954	-1.5875568
PPP4R2	protein phosphatase 4, regulatory subunit 2	-1.9733928	-1.7638904	-1.398352
PPP6C	protein phosphatase 6, catalytic subunit	1.591508	1.4133716	1.4111096
PPP6R2	protein phosphatase 6, regulatory subunit 2	1.0184474	1.3272423	2.0512526
PPP6R2	protein phosphatase 6, regulatory subunit 2	1.1672112	1.4048364	2.4108896
PPRC1	peroxisome proliferator-activated receptor gamma, coactivator-related 1	-1.6411145	-2.3895512	-3.4934893
PPT1	palmitoyl-protein thioesterase 1	-1.0033364	-2.0780256	-2.8742661
PQBP1	polyglutamine binding protein 1	1.126187	1.3043776	1.5304526
PQLC1	PQ loop repeat containing 1	1.1422099	1.7764132	2.6272926
PQLC1	PQ loop repeat containing 1	1.7957472	2.7524235	4.251072
PRADC1	protease-associated domain containing 1	1.9581033	2.378636	2.030913
PRAME	preferentially expressed antigen in melanoma	1.9928877	2.2860408	2.58813
PRAP1	proline-rich acidic protein 1	1.3331174	3.1791425	8.656308
PRB1	proline-rich protein BstNI subfamily 1	2.466167	2.7512386	2.9439423
PRB3	proline-rich protein BstNI subfamily 3	4.596301	4.814433	4.8747587
PRC1	protein regulator of cytokinesis 1	2.155073	-3.5090475	-3.921276
PRCP	prolylcarboxypeptidase (angiotensinase C)	1.5571966	2.1468625	1.703129
PRDM4	PR domain containing 4	-1.6577178	-1.8720189	-2.2468693
PRDX1	peroxiredoxin 1	1.3662322	1.2794145	1.864833
PRDX1	peroxiredoxin 1	1.5221689	1.5137411	2.0866609
PRDX5	peroxiredoxin 5	-1.0663708	1.1129934	-1.5645849
PRDX6	peroxiredoxin 6	1.688777	2.5601575	2.9937341
PRELID1	PRELI domain containing 1	1.3101406	1.5750737	1.635495
PREP	prolyl endopeptidase	1.4419987	1.1907552	1.5210679
PRICKLE1	prickle homolog 1 (Drosophila)	2.844967	1.1889337	-1.3841908
PRIM1	primase, DNA, polypeptide 1 (49kDa)	1.4463995	-1.5517588	-1.4031957
PRIM2	primase, DNA, polypeptide 2 (58kDa)	1.4747392	-1.7185615	-1.7011555
PRIMA1	proline rich membrane anchor 1	1.4775344	1.4690728	1.8164558
PRKAB2	protein kinase, AMP-activated, beta 2 non-catalytic subunit	-1.0206084	1.7870579	2.7350445
PRKACB	protein kinase, cAMP-dependent, catalytic, beta	-1.5306889	-1.8745022	-1.4889337
PRKAG2	protein kinase, AMP-activated, gamma 2 non-catalytic subunit	-1.4541851	1.9945606	4.2356544
PRKAG3	protein kinase, AMP-activated, gamma 3 non-catalytic subunit	2.8009467	2.628281	2.6145587
PRKAR1B	protein kinase, cAMP-dependent, regulatory, type I, beta	-1.174592	-1.506868	-5.1916842
PRKCA	protein kinase C, alpha	-1.181476	-1.5772825	-1.8250333
PRKCD	protein kinase C, delta	-1.2386205	-1.669111	-2.7540736
PRKD2	protein kinase D2	-1.4568706	-1.6381607	-1.6531724
PRKRA	protein kinase, interferon-inducible double stranded RNA dependent activator	1.5207522	1.3510678	1.2487332
PRKXP1	protein kinase, X-linked, pseudogene 1	1.312054	1.090205	-1.7614883
PRLHR	prolactin releasing hormone receptor	1.8479471	1.6390257	1.8752719
PRMT1	protein arginine methyltransferase 1	1.5294397	1.0204266	-1.7633232
PRMT2	protein arginine methyltransferase 2	-1.0565951	-2.9319885	-2.8202715
PRMT2	protein arginine methyltransferase 2	-1.5468525	-2.6531944	-2.5647466
PROC	protein C (inactivator of coagulation factors Va and VIIIa)	-1.6952593	4.025756	4.3219094
PROCR	protein C receptor, endothelial	1.4576942	-1.5646285	-2.645956
PRODH	proline dehydrogenase (oxidase) 1	2.922215	9.914066	8.481032
PRODH	proline dehydrogenase (oxidase) 1	3.730886	10.943111	10.305259
PRODH2	proline dehydrogenase (oxidase) 2	-10.504548	2.5493338	4.929577
PROS1	protein S (alpha)	3.1470556	3.8379102	3.2707446
PROS1	protein S (alpha)	2.5843923	2.1619542	1.5418209
PROSC	proline synthetase co-transcribed homolog (bacterial)	-1.6695971	-1.0307456	1.1444094
PROSER1	proline and serine rich 1	1.2322077	-1.3143536	-1.5249206
PRPF38A	pre-mRNA processing factor 38A	1.0609272	-1.2914741	-1.5671666
PRPF4	pre-mRNA processing factor 4	1.0128323	-1.3109425	-1.5202165
PRPS1	phosphoribosyl pyrophosphate synthetase 1	1.6348746	-1.1982169	1.3170611
PRPS2	phosphoribosyl pyrophosphate synthetase 2	1.9594755	3.627283	1.0428609
PRR13	proline rich 13	1.4079375	1.5607363	1.4292936
PRR14	proline rich 14	-1.6260829	-1.5905417	-2.0942075
PRR15L	proline rich 15-like	5.248927	24.42362	3.3540165
PRR25	proline rich 25	1.9968823	2.2172713	2.6271267
PRR33	proline rich 33	2.4455802	2.3015025	3.279583
PRR5	proline rich 5 (renal)	-1.409256	-1.37819	-1.6155232
PRR5-ARHGAP8	PRR5-ARHGAP8 readthrough	2.8264065	3.9520113	2.590829
PRRC2B	proline-rich coiled-coil 2B	1.4046575	1.4683328	1.7947924
PRRC2B	proline-rich coiled-coil 2B	-1.5859592	-1.4295096	-1.7651081
PRRC2C	proline-rich coiled-coil 2C	-2.8387072	-2.7867413	-3.31287
PRSS23	protease, serine, 23	4.0311413	-1.4704738	-6.749785
PRSS53	protease, serine, 53	-1.1039598	1.8225701	2.7377203
PSCA	prostate stem cell antigen	6.4129453	16.810858	1.935464
PSD	pleckstrin and Sec7 domain containing	3.4725518	3.565217	4.580523
PSD3	pleckstrin and Sec7 domain containing 3	-1.6644516	-1.2464813	-2.3919876
PSD4	pleckstrin and Sec7 domain containing 4	4.521947	5.126934	6.2444234
PSEN2	presenilin 2	1.3389653	1.5148451	2.0968683
PSIP1	PC4 and SFRS1 interacting protein 1	1.0481284	-1.5212489	-1.1375718
PSKH1	protein serine kinase H1	1.5040909	1.6326652	2.1801412
PSMA4	proteasome (prosome, macropain) subunit, alpha type, 4	-1.1699281	-1.6695702	-1.3474768
PSMB8	proteasome (prosome, macropain) subunit, beta type, 8	1.0202066	-1.3979235	-2.0189939
PSMB9	proteasome (prosome, macropain) subunit, beta type, 9	1.087276	-1.7370065	-1.8476969
PSMC3	proteasome (prosome, macropain) 26S subunit, ATPase, 3	1.1590804	-1.25798	-1.5799619
PSMD1	proteasome (prosome, macropain) 26S subunit, non-ATPase, 1	-1.7382405	-2.1176229	-1.9787778
PSMD10	proteasome (prosome, macropain) 26S subunit, non-ATPase, 10	-2.8590949	-2.5644107	-2.4980552

PSMD11	proteasome (prosome, macropain) 26S subunit, non-ATPase, 11	-1.0986776	-1.5559741	-1.5471585
PSMD2	proteasome (prosome, macropain) 26S subunit, non-ATPase, 2	-1.2012035	-1.7110931	-1.5224186
PSMD2	proteasome (prosome, macropain) 26S subunit, non-ATPase, 2	-1.4659908	-2.2329397	-2.1681902
PSME4	proteasome (prosome, macropain) activator subunit 4	-1.2916957	-1.8331017	-2.127471
PSMF1	proteasome (prosome, macropain) inhibitor subunit 1 (PI31)	1.310776	1.4545423	1.7612568
PSMG2	proteasome (prosome, macropain) assembly chaperone 2	-1.4605329	-1.5073204	-1.5948648
PSMG3	proteasome (prosome, macropain) assembly chaperone 3	-1.5667125	-1.941177	-1.7144358
PSMG4	proteasome (prosome, macropain) assembly chaperone 4	1.7759126	1.7135448	1.7252265
PSRC1	proline/serine-rich coiled-coil 1	2.3704371	-1.1413141	-1.7581364
PSTK	phosphoseryl-tRNA kinase	1.0850346	-1.5321295	-1.6590513
PTAFR	platelet-activating factor receptor	3.084805	3.4755917	4.1545143
PTBP1	polypyrimidine tract binding protein 1	-1.0117803	-1.2822992	-1.8147972
PTBP3	polypyrimidine tract binding protein 3	-1.6857365	-2.2360203	-2.9280732
PTEN	phosphatase and tensin homolog	1.2327238	1.2426542	1.9384719
PTGDS	prostaglandin D2 synthase 21kDa (brain)	1.847804	2.045678	3.1372485
PTGER4	prostaglandin E receptor 4 (subtype EP4)	-1.2288353	-1.4026341	-3.6855655
PTGES3	prostaglandin E synthase 3 (cytosolic)	-1.4867773	-2.1013515	-1.6429707
PTGFRN	prostaglandin F2 receptor inhibitor	-1.013698	-2.0620356	-2.438961
PTGR1	prostaglandin reductase 1	-1.9432986	1.3392751	1.2125583
PTGR1	prostaglandin reductase 1	-2.1148598	1.0376462	1.6160941
PTH2	parathyroid hormone 2	1.9044268	1.9484373	2.2621937
PTK2B	protein tyrosine kinase 2 beta	-1.3658217	1.9649636	2.418543
PTMA	prothymosin, alpha	1.8519374	-1.022148	-1.4191473
PTMA	prothymosin, alpha	1.2100697	-1.61863	-2.242037
PTMS	parathymosin	-1.0431645	1.4021616	1.6734873
PTN	pleiotrophin	1.0895468	-2.4947484	-1.7865334
PTOV1	prostate tumor overexpressed 1	-1.727359	-1.8108225	-1.8438356
PTP4A1	protein tyrosine phosphatase type IVA, member 1	-2.192208	-1.614895	-1.3454503
PTP4A1	protein tyrosine phosphatase type IVA, member 1	-1.1892161	1.0858153	1.7532418
PTPLA	protein tyrosine phosphatase-like (proline instead of catalytic arginine), member A	2.215374	2.374867	1.3020089
PTPLAD1	protein tyrosine phosphatase-like A domain containing 1	1.116312	1.2570305	2.3575518
PTPLAD1	protein tyrosine phosphatase-like A domain containing 1	-1.0334415	1.0193194	1.8500731
PTPLB	protein tyrosine phosphatase-like (proline instead of catalytic arginine), member b	1.0088706	-1.3469493	1.9589536
PTPN1	protein tyrosine phosphatase, non-receptor type 1	1.7912292	1.0759736	-1.02677
PTPN18	protein tyrosine phosphatase, non-receptor type 18 (brain-derived)	-1.068603	1.5915154	2.781822
PTPN2	protein tyrosine phosphatase, non-receptor type 2	-1.4039959	-1.7788346	-2.2779992
PTPN23	protein tyrosine phosphatase, non-receptor type 23	-1.4686601	-1.7381098	-2.140805
PTPRH	protein tyrosine phosphatase, receptor type, H	-2.8794496	-1.6061515	-3.6186483
PTPRJ	protein tyrosine phosphatase, receptor type, J	-1.2961332	-1.2878994	1.7637912
PTPRM	protein tyrosine phosphatase, receptor type, M	-1.7877512	1.101743	1.0994505
PTPRU	protein tyrosine phosphatase, receptor type, U	1.5896505	1.1580895	-1.5576677
PTPRU	protein tyrosine phosphatase, receptor type, U	-1.3013725	-1.6036085	-3.014765
PTRF	polymerase I and transcript release factor	1.8628994	1.0686307	-1.3593181
PTRF	polymerase I and transcript release factor	-2.309544	-2.1438565	-1.8114859
PTTG1	pituitary tumor-transforming 1	2.2474377	-3.2876508	-2.8096216
PTTG1IP	pituitary tumor-transforming 1 interacting protein	-1.459441	-1.9764649	-2.2425797
PTTG2	pituitary tumor-transforming 2	2.2269824	-3.1612158	-2.6330042
PTX3	pentraxin 3, long	-2.1785076	-10.068036	-35.018745
PURA	purine-rich element binding protein A	-1.0205433	1.4863998	1.6357266
PURB	purine-rich element binding protein B	1.496126	-1.0353122	2.035133
PURB	purine-rich element binding protein B	-1.5001023	-2.3004653	-3.2725606
PUS3	pseudouridylate synthase 3	1.4976153	1.59413	1.8052102
PUSL1	pseudouridylate synthase-like 1	1.5158112	1.5406213	1.0724198
PVRL2	poliovirus receptor-related 2 (herpesvirus entry mediator B)	-1.3184805	-1.7453992	-1.8582197
PVRL2	poliovirus receptor-related 2 (herpesvirus entry mediator B)	1.4230487	1.095833	2.4387388
PVRL3	poliovirus receptor-related 3	1.093752	1.3461905	1.8372335
PVRL4	poliovirus receptor-related 4	-1.1620592	-2.312332	-13.581745
PVT1	Pvt1 oncogene (non-protein coding)	-1.2127165	-2.097534	-4.0743303
PVT1	Pvt1 oncogene (non-protein coding)	1.6992388	1.4154702	1.5103441
PVT1	Pvt1 oncogene (non-protein coding)	1.8930726	1.5723208	1.6993073
PWP2	PWP2 periodic tryptophan protein homolog (yeast)	-1.2451591	-1.5187594	-2.0587528
PWWP2B	PWWP domain containing 2B	-1.0353264	-1.352496	-2.9958901
PXMP2	peroxisomal membrane protein 2, 22kDa	1.9020078	6.1437364	6.7423644
PXMP4	peroxisomal membrane protein 4, 24kDa	2.0700185	2.0366468	2.0579958
PXN	paxillin	1.7725276	-1.068639	-1.5994097
PYCR2	pyrroline-5-carboxylate reductase family, member 2	1.470123	1.5150707	1.4909934
PYGL	phosphorylase, glycogen, liver	1.6615311	1.7635839	-1.0244907
PYGO2	pygopus family PHD finger 2	2.9537036	3.4081235	3.263145
PYY2	peptide YY, 2 (pseudogene)	10.938482	10.746635	12.981932
PZP	pregnancy-zone protein	1.3707113	10.499107	1.8486563
QDPR	quinoid dihydropteridine reductase	1.2608	1.7423338	4.9896502
QPRT	quinolinate phosphoribosyltransferase	1.1141566	1.466604	2.2763877
QSER1	glutamine and serine rich 1	1.0372007	-1.1910436	-2.452855
QTRTD1	queuine tRNA-ribosyltransferase domain containing 1	-1.1587645	-1.540261	-1.593218
R3HDM1	R3H domain containing 1	1.7078518	1.1513547	1.0558848
R3HDM4	R3H domain containing 4	-1.4639902	-1.5927761	-1.7774692
RAB11FIP4	RAB11 family interacting protein 4 (class II)	-1.6092757	1.395842	2.254165
RAB15	RAB15, member RAS oncogene family	-1.3070345	-1.6274136	-1.64724
RAB17	RAB17, member RAS oncogene family	-1.386492	5.731774	11.726479
RAB17	RAB17, member RAS oncogene family	-1.6919117	5.2794538	10.096574
RAB1B	RAB1B, member RAS oncogene family	1.5147406	1.4696665	1.5153884
RAB20	RAB20, member RAS oncogene family	1.3473443	2.0727506	2.2563472
RAB26	RAB26, member RAS oncogene family	1.5374371	3.670524	4.469522
RAB35	RAB35, member RAS oncogene family	1.5203508	-1.0017382	-1.119397
RAB37	RAB37, member RAS oncogene family	1.0785227	2.7233233	1.1758846
RAB43	RAB43, member RAS oncogene family	-1.3384131	1.0872945	1.6879247
RAB7B	RAB7B, member RAS oncogene family	-1.2226533	-1.999715	-12.6154995
RAB8A	RAB8A, member RAS oncogene family	1.129889	1.0609081	1.670134
RABEPK	Rab9 effector protein with kelch motifs	-1.2343779	-1.5210952	-1.7399218
RABEPK	Rab9 effector protein with kelch motifs	1.2740878	1.8051503	1.7477024
RABGAP1L	RAB GTPase activating protein 1-like	-8.856643	-5.0366335	-3.7272377
RABL2B	RAB, member of RAS oncogene family-like 2B	-3.4971287	-3.1305666	-2.517824
RAC2	ras-related C3 botulinum toxin substrate 2 (rho family, small GTP binding protein Rac2)	-1.5876443	-2.7350898	-6.327938
RAC2	ras-related C3 botulinum toxin substrate 2 (rho family, small GTP binding protein Rac2)	-1.5896512	-2.7524126	-4.658281
RAC2	ras-related C3 botulinum toxin substrate 2 (rho family, small GTP binding protein Rac2)	1.4405375	1.7170149	3.6463814
RACGAP1	Rac GTPase activating protein 1	2.5518675	-1.9552412	-2.5276356
RAD51	RAD51 recombinase	2.032423	-3.0100265	-4.2456746
RAD51C	RAD51 paralog C	1.1574684	-1.7835321	-2.63002
RAD51D	RAD51 paralog D	1.2386833	2.9605176	2.4558413
RAD54L	RAD54-like (S. cerevisiae)	1.470777	-5.5960336	-8.3021
RAD54L2	RAD54-like 2 (S. cerevisiae)	-1.1773274	-1.6171272	-1.2885661
RAD9A	RAD9 homolog A (S. pombe)	-2.1169446	-1.7031242	-1.4894208
RAI1	retinoic acid induced 1	-1.492694	-1.9855578	-2.843881
RAI14	retinoic acid induced 14	-1.9394543	-3.5910878	-4.4895077
RAI2	retinoic acid induced 2	1.1267582	1.9725639	2.039674
RALBP1	ralA binding protein 1	-1.7665875	-2.2147589	-1.9676634
RALGAPB	Ral GTPase activating protein, beta subunit (non-catalytic)	-1.5722336	-1.7106394	-1.8271989
RAN	RAN, member RAS oncogene family	1.5269864	-1.0611376	-1.1688728
RANBP1	RAN binding protein 1	1.5916357	-1.2424521	-1.514335

RANBP10	RAN binding protein 10	-1.253947	1.6892802	1.9221257
RANBP9	RAN binding protein 9	-1.4426472	-1.8721763	-1.5205541
RANGRF	RAN guanine nucleotide release factor	-1.0766075	-1.9054244	-4.8064713
RAPH1	Ras association (RalGDS/AF-6) and pleckstrin homology domains 1	2.0092108	2.0870023	2.4949002
RARA	retinoic acid receptor, alpha	1.3194947	1.4364582	1.5613066
RARA	retinoic acid receptor, alpha	1.36336	1.4626198	1.6480734
RARS2	arginyl-tRNA synthetase 2, mitochondrial	1.8088939	1.4292623	1.2584269
RASSF1	Ras association (RalGDS/AF-6) domain family member 1	-1.1220692	-1.7301338	-2.880706
RASSF4	Ras association (RalGDS/AF-6) domain family member 4	-1.0634401	1.8552148	2.6162925
RB1CC1	RB1-inducible coiled-coil 1	-1.5279021	-1.2453564	-1.1122274
RBAK-RBAKDN	RBAK-RBAKDN readthrough	2.4331875	2.518887	3.0042896
RBBP4	retinoblastoma binding protein 4	-1.7375269	-1.678919	-3.099881
RBBP8	retinoblastoma binding protein 8	1.0259598	1.0094324	-2.091106
RBFA	ribosome binding factor A (putative)	1.5597874	1.2753676	1.1872932
RBKS	ribokinase	1.2383212	2.0336523	2.371649
RBL2	retinoblastoma-like 2	-1.0517935	1.0173832	1.5682518
RBM10	RNA binding motif protein 10	2.8317463	3.1706328	3.1306126
RBM25	RNA binding motif protein 25	-1.2397447	-1.6460434	-1.5407442
RBM28	RNA binding motif protein 28	1.1178986	-1.3713036	-1.657105
RBM3	RNA binding motif (RNP1, RRM) protein 3	2.0396938	1.0846537	-1.4547557
RBM3	RNA binding motif (RNP1, RRM) protein 3	2.062761	1.1419725	-1.4995034
RBM41	RNA binding motif protein 41	-1.6146195	-1.1867765	-1.1618239
RBM43	RNA binding motif protein 43	1.7025007	1.9803799	1.5692767
RBM45	RNA binding motif protein 45	1.4931734	1.5117651	1.6053838
RBM47	RNA binding motif protein 47	-1.7713006	1.2911545	1.5127971
RBMS1	RNA binding motif, single stranded interacting protein 1	1.1262473	-1.2360376	-2.3007069
RBMS1	RNA binding motif, single stranded interacting protein 1	1.0599344	-1.3670377	-2.816501
RBMS1	RNA binding motif, single stranded interacting protein 1	1.0235738	-1.4807231	-2.3955717
RBMXL1	RNA binding motif protein, X-linked-like 1	1.6771137	1.1906848	-1.0180113
RBP4	retinol binding protein 4, plasma	-1.1893889	4.2243366	6.9690614
RBP5	retinol binding protein 5, cellular	-2.1091614	13.139217	49.7925
RBX1	ring-box 1, E3 ubiquitin protein ligase	1.604274	1.2890166	1.575681
RC3H2	ring finger and CCCH-type domains 2	-1.2173626	-1.3572133	-1.5924549
RCAN2	regulator of calcineurin 2	1.6538503	1.6254077	2.8082786
RCC2	regulator of chromosome condensation 2	-1.2126111	-2.7964814	-4.5764637
RCCD1	RCC1 domain containing 1	1.5384974	-1.2393728	-1.359209
RCL1	RNA terminal phosphate cyclase-like 1	-1.3500248	2.0662813	-1.0361704
RCL1	RNA terminal phosphate cyclase-like 1	1.1606507	2.2854083	1.4170817
RCN1	reticulocalbin 1, EF-hand calcium binding domain	-1.2783159	-2.298451	-3.0476656
RCOR1	REST corepressor 1	1.685208	1.408375	1.3886455
RDH11	retinol dehydrogenase 11 (all-trans/9-cis/11-cis)	-1.160173	-1.0357776	1.9203852
RDH12	retinol dehydrogenase 12 (all-trans/9-cis/11-cis)	-8.426882	3.7434282	6.8221726
RDH14	retinol dehydrogenase 14 (all-trans/9-cis/11-cis)	1.3155987	1.4363304	1.6568806
RDH16	retinol dehydrogenase 16 (all-trans)	1.0480137	18.853888	23.704102
RDH5	retinol dehydrogenase 5 (11-cis/9-cis)	-1.1144531	4.3344717	17.16563
RECK	reversion-inducing-cysteine-rich protein with kazal motifs	3.0789323	1.8984873	2.321505
RECQL	RecQ helicase-like	-1.1396085	-1.2740898	-1.7312431
REEP4	receptor accessory protein 4	-1.2175355	-2.004834	-1.6090987
REEP5	receptor accessory protein 5	1.2166735	1.881482	2.801892
REEP6	receptor accessory protein 6	-1.0018805	2.1597607	5.595819
REEP6	receptor accessory protein 6	-1.0171822	1.6988775	3.6867738
RELB	v-rel avian reticuloendotheliosis viral oncogene homolog B	-1.7756616	-2.2637732	-7.130223
RELL1	RELT-like 1	1.1942025	-1.2873567	-1.716671
RELT	RELT tumor necrosis factor receptor	-1.0374247	-1.4704269	-1.5822084
REPS1	RALBP1 associated Eps domain containing 1	1.2461916	1.7265389	2.58352
REPS2	RALBP1 associated Eps domain containing 2	1.7067198	2.8209815	1.2839376
REREP3	arginine-glutamic acid dipeptide (RE) repeats pseudogene 3	2.6869166	1.8941526	1.5562606
RETSAT	retinol saturase (all-trans-retinol 13,14-reductase)	-1.0844116	1.8010405	2.005638
REXO1	REX1, RNA exonuclease 1 homolog (S. cerevisiae)	1.6044834	1.1884431	1.2486185
REXO2	RNA exonuclease 2	1.6843446	1.1865717	-1.0513874
RFC4	replication factor C (activator 1) 4, 37kDa	1.2298815	-2.7623243	-2.985618
RFPL4AL1	ret finger protein-like 4A-like 1	-2.3955665	-1.9596052	-1.1490731
RFPL4AL1	ret finger protein-like 4A-like 1	-2.3202546	-1.9184778	-1.138864
RFTN1	raftlin, lipid raft linker 1	2.109385	2.298499	2.480018
RFWD3	ring finger and WD repeat domain 3	-1.0047265	-2.5411026	-3.1117947
RFX1	regulatory factor X, 1 (influences HLA class II expression)	-1.8911002	-1.5705857	-1.6892558
RFX5	regulatory factor X, 5 (influences HLA class II expression)	1.3692029	-1.8053337	-2.2725034
RFX7	regulatory factor X, 7	1.279939	-1.5853504	-1.7974557
RGL1	ral guanine nucleotide dissociation stimulator-like 1	1.6191436	1.0551589	-1.2819463
RGL2	ral guanine nucleotide dissociation stimulator-like 2	-1.8086721	-1.4646679	-3.060184
RGN	regucalcin	1.1594807	4.755982	15.243539
RGP1	RGP1 retrograde golgi transport homolog (S. cerevisiae)	1.385057	1.7726833	1.591449
RGPD5	RANBP2-like and GRIP domain containing 5	-1.6198678	-1.5381207	-1.7144629
RGPD5	RANBP2-like and GRIP domain containing 5	-1.6485785	-1.8424164	-1.7949237
RGS10	regulator of G-protein signaling 10	1.1973659	-1.08251	-1.7994884
RGS14	regulator of G-protein signaling 14	-2.5569506	-1.0199649	-1.1509469
RGS19	regulator of G-protein signaling 19	1.192071	-1.4031291	-4.631009
RGS2	regulator of G-protein signaling 2	-1.6516575	1.1092103	-3.2632565
RGS3	regulator of G-protein signaling 3	1.6682543	1.7225289	1.879911
RHBDF1	rhomboid 5 homolog 1 (Drosophila)	-1.4660575	-1.6567208	-2.1373823
RHBDF2	rhomboid 5 homolog 2 (Drosophila)	-1.2983973	-1.44826	-2.4583173
RHEB	Ras homolog enriched in brain	1.5355498	1.0901617	1.0992622
RHEBL1	Ras homolog enriched in brain like 1	-2.978527	-4.6138983	-4.311577
RHNO1	RAD9-HUS1-RAD1 interacting nuclear orphan 1	1.5451708	-1.5730643	-1.7373755
RHOA	ras homolog family member A	2.479235	1.9049008	2.208495
RHOB	ras homolog family member B	1.501968	2.079831	4.833331
RHOB	ras homolog family member B	2.0572886	2.9993837	5.8307276
RHOBTB1	Rho-related BTB domain containing 1	-1.1695354	-2.8031845	-7.580559
RHOBTB3	Rho-related BTB domain containing 3	1.9731483	1.5961797	1.131834
RHOD	ras homolog family member D	1.040118	-1.2192668	-2.2667005
RHOQ	ras homolog family member Q	1.7454697	1.1277487	-1.4561496
RHOQ	ras homolog family member Q	1.2271813	-1.3754125	-2.2905645
RHOQ	ras homolog family member Q	1.206665	-1.5977095	-2.5764952
RHOU	ras homolog family member U	-1.4526602	2.1546257	3.4372447
RHPN2	rhophilin, Rho GTPase binding protein 2	-1.0016509	1.2487558	1.5087903
RIBC2	RIB43A domain with coiled-coils 2	1.3750986	-3.9892018	-9.064559
RILPL2	Rab interacting lysosomal protein-like 2	2.0592434	1.791495	2.1247344
RIMBP3	RIMS binding protein 3	2.5533988	2.5668592	1.7491544
RIN1	Ras and Rab interactor 1	1.0406626	-1.4253303	-5.8167443
RINL	Ras and Rab interactor-like	-1.1623483	1.2772328	-1.5622252
RIOK1	RIO kinase 1	-1.5306457	-1.6081429	-2.0598257
RIPK1	receptor (TNFRSF)-interacting serine-threonine kinase 1	1.1792547	1.1832495	1.6528605
RIPK2	receptor-interacting serine-threonine kinase 2	-1.4424242	-1.9455676	-1.7865162
RLTPR	RGD motif, leucine rich repeats, tropomodulin domain and proline-rich containing	1.6088985	1.6917381	2.2272084
RMDN1	regulator of microtubule dynamics 1	1.4809846	1.9561286	2.0447853
RMDN3	regulator of microtubule dynamics 3	-1.0726014	-1.0711204	1.6892574
RMI1	RecQ mediated genome instability 1	2.020243	1.4455787	1.3322456
RMI2	RecQ mediated genome instability 2	1.1102003	-3.6894643	-5.37289
RNA18S5	RNA, 18S ribosomal 5	-4.6189117	-2.6971195	-3.6750247

RNASE4	ribonuclease, RNase A family, 4	-1.8764919	1.6406866	1.6013471
RNASEH2A	ribonuclease H2, subunit A	1.435595	-1.5775485	-1.9654248
RNASEH2B	ribonuclease H2, subunit B	-1.3492362	-2.0625477	-2.3624806
RNASEH2B	ribonuclease H2, subunit B	1.5702211	2.1031396	3.4856267
RNASET2	ribonuclease T2	1.7385283	1.7649382	1.7541771
RND3	Rho family GTPase 3	-1.5701071	-3.9314647	-3.2128134
RNF103	ring finger protein 103	-1.3879901	1.3614091	2.572155
RNF113A	ring finger protein 113A	1.6457961	1.5009779	1.299666
RNF115	ring finger protein 115	1.5131605	-1.0059112	-1.6695158
RNF123	ring finger protein 123	-1.0601102	1.1687881	1.606597
RNF125	ring finger protein 125, E3 ubiquitin protein ligase	1.1501967	2.4920878	10.5847645
RNF126	ring finger protein 126	1.3825662	1.5204887	1.5741385
RNF13	ring finger protein 13	1.262859	1.6223862	1.9372876
RNF130	ring finger protein 130	1.2548239	1.6081	1.8222256
RNF141	ring finger protein 141	1.5695659	1.938388	2.5413013
RNF17	ring finger protein 17	-1.0542529	-1.3809367	-1.5600181
RNF181	ring finger protein 181	1.1026065	1.4883689	1.6303531
RNF207	ring finger protein 207	-1.2503878	-1.092105	-2.5939386
RNF213	ring finger protein 213	1.334634	-1.6686631	-3.04473
RNF216	ring finger protein 216	-1.6137717	-1.7873296	-1.3614534
RNF222	ring finger protein 222	4.533067	4.898802	5.410768
RNF43	ring finger protein 43	-1.4530909	2.4809577	4.281939
RNF44	ring finger protein 44	-1.1043019	-1.1295205	-1.8611054
RNF5	ring finger protein 5, E3 ubiquitin protein ligase	1.0329405	1.6477214	1.7167467
RNMT	RNA (guanine-7-) methyltransferase	-1.439207	-1.78937	-1.8609258
RNPEP	arginyl aminopeptidase (aminopeptidase B)	1.5684197	1.2459254	1.5332663
RNPEPL1	arginyl aminopeptidase (aminopeptidase B)-like 1	-1.031996	1.3346138	1.6411489
ROM1	retinal outer segment membrane protein 1	1.8490003	1.7922585	-1.7002419
ROR1	receptor tyrosine kinase-like orphan receptor 1	2.021742	-1.0483254	-1.8463334
ROR1	receptor tyrosine kinase-like orphan receptor 1	2.0047514	-1.023281	-1.9519181
ROR2	receptor tyrosine kinase-like orphan receptor 2	1.515648	-1.060769	-3.6014097
RORA	RAR-related orphan receptor A	-3.2753186	1.5528971	2.9899886
RORC	RAR-related orphan receptor C	1.6604714	6.323297	10.82159
ROS1	ROS proto-oncogene 1 , receptor tyrosine kinase	1.8553642	1.0888735	-3.032479
RPAP2	RNA polymerase II associated protein 2	-1.1536018	-1.1834686	-1.5090133
RPH3AL	rabphilin 3A-like (without C2 domains)	-1.3972384	-1.0998211	-1.6502049
RPIA	ribose 5-phosphate isomerase A	1.4020898	-1.0950239	-1.5716027
RPL10A	ribosomal protein L10a	1.4191962	1.5622345	1.2787457
RPL10L	ribosomal protein L10-like	1.5109686	1.3145702	1.0071349
RPL13	ribosomal protein L13	1.5527732	1.356159	-1.5252742
RPL13AP17	ribosomal protein L13a pseudogene 17	3.1084807	2.603554	1.8904262
RPL13AP3	ribosomal protein L13a pseudogene 3	2.040535	1.6509489	1.4365838
RPL13P5	ribosomal protein L13 pseudogene 5	-1.2847656	-1.3185339	-2.6577141
RPL19	ribosomal protein L19	1.614997	1.5989279	1.3491529
RPL19P12	ribosomal protein L19 pseudogene 12	1.6166501	1.3039968	-1.010184
RPL21	ribosomal protein L21	-1.2235051	-1.3309835	-1.5474592
RPL22	ribosomal protein L22	1.6827426	1.3449223	1.186923
RPL22L1	ribosomal protein L22-like 1	-1.1169318	-1.0989792	-2.1424036
RPL23A	ribosomal protein L23a	1.1442724	-1.0803131	-1.7138369
RPL23AP53	ribosomal protein L23a pseudogene 53	1.1819881	-1.3766385	-1.8558326
RPL23AP7	ribosomal protein L23a pseudogene 7	1.0697532	-1.8689665	-3.0875092
RPL28	ribosomal protein L28	1.3492955	1.5022167	1.0286604
RPL29	ribosomal protein L29	1.6516044	1.6864148	1.1701216
RPL29	ribosomal protein L29	1.5207824	1.4983658	1.0307128
RPL29P2	ribosomal protein L29 pseudogene 2	1.8699428	1.8297178	1.2589958
RPL30	ribosomal protein L30	1.6094643	1.6781915	1.3122118
RPL37A	ribosomal protein L37a	1.5052941	1.5554092	1.3207012
RPL38	ribosomal protein L38	1.3558697	1.5292506	1.3838696
RPL6	ribosomal protein L6	1.4991492	1.5890338	1.3523196
RPL7	ribosomal protein L7	1.5970833	1.6675462	1.3369068
RPL7	ribosomal protein L7	1.8087786	1.8431143	1.3749313
RPN1	ribophorin I	-1.6394056	-1.8746705	-1.8125827
RPN2	ribophorin II	-1.6088926	-1.6900282	-1.7192907
RPP25L	ribonuclease P/MRP 25kDa subunit-like	2.3357706	3.1596708	2.72566
RPP30	ribonuclease P/MRP 30kDa subunit	1.2861092	1.1973703	2.0234604
RPRD1B	regulation of nuclear pre-mRNA domain containing 1B	2.4315424	2.60561	2.9049559
RPS10P7	ribosomal protein S10 pseudogene 7	1.8781031	1.7617369	1.5009524
RPS13	ribosomal protein S13	1.5674057	1.4512783	1.1691558
RPS15A	ribosomal protein S15a	-1.1010019	-1.2769784	-1.6715579
RPS23	ribosomal protein S23	1.5049639	1.2790253	1.138703
RPS23	ribosomal protein S23	1.2248746	1.5695717	-1.3123599
RPS26	ribosomal protein S26	1.8250636	1.8243984	1.893067
RPS27L	ribosomal protein S27-like	1.0757899	-1.3469263	-1.8062644
RPS29	ribosomal protein S29	1.1343158	1.6060705	2.1632426
RPS3	ribosomal protein S3	1.5832447	1.2024709	-1.3389672
RPS3A	ribosomal protein S3A	-1.0199474	-1.1320922	-1.507906
RPS6KA2	ribosomal protein S6 kinase, 90kDa, polypeptide 2	2.5300064	2.4291108	1.8765152
RPS6KA3	ribosomal protein S6 kinase, 90kDa, polypeptide 3	-1.0066426	1.8776487	2.3224902
RPS6KA3	ribosomal protein S6 kinase, 90kDa, polypeptide 3	-1.7356312	-1.628367	-1.3840325
RPS6KA4	ribosomal protein S6 kinase, 90kDa, polypeptide 4	1.0471345	-1.391912	-2.2989247
RPS6KC1	ribosomal protein S6 kinase, 52kDa, polypeptide 1	-1.3486078	-1.8413444	-1.9408102
RPSAP58	ribosomal protein SA pseudogene 58	1.5670258	1.0633218	-1.1268421
RPUSD1	RNA pseudouridylate synthase domain containing 1	1.649466	1.265132	1.0255433
RPUSD2	RNA pseudouridylate synthase domain containing 2	1.4248449	1.5225757	1.8254015
RRAD	Ras-related associated with diabetes	-3.2400253	-12.490572	-53.97538
RRAS2	related RAS viral (r-ras) oncogene homolog 2	1.4817243	2.0309317	2.6137283
RRAS2	related RAS viral (r-ras) oncogene homolog 2	1.3617187	1.8389134	2.3348124
RRBP1	ribosome binding protein 1	-2.382449	-1.808749	-2.1713457
RRH	retinal pigment epithelium-derived rhodopsin homolog	3.4202123	2.9214096	2.4521081
RRM1	ribonucleotide reductase M1	1.6504219	-1.7201014	-1.6615005
RRM2	ribonucleotide reductase M2	1.6766675	-5.8525586	-4.475679
RRM2B	ribonucleotide reductase M2 B (TP53 inducible)	1.17302	-1.4949006	-1.971009
RRNAD1	ribosomal RNA adenine dimethylase domain containing 1	-1.0996274	1.2291025	2.115038
RRP12	ribosomal RNA processing 12 homolog (S. cerevisiae)	-1.8337637	-1.6985476	-3.6222389
RRP1B	ribosomal RNA processing 1B	1.0111244	-1.7346252	-1.5075966
RRP7B	ribosomal RNA processing 7 homolog B (S. cerevisiae)	1.9790347	1.9562457	2.160267
RRP8	ribosomal RNA processing 8, methyltransferase, homolog (yeast)	1.5492129	1.5743756	1.3634862
RRS1	RRS1 ribosome biogenesis regulator homolog (S. cerevisiae)	2.3297453	1.536383	1.4566714
RSBN1L	round spermatid basic protein 1-like	1.6716921	1.4732153	1.5819658
RSPH9	radial spoke head 9 homolog (Chlamydomonas)	1.8853744	2.2043715	2.145263
RSU1	Ras suppressor protein 1	-1.0551295	-2.0483143	-2.3567965
RTBDN	retbindin	1.5051231	1.2461013	1.2706237
RTEL1	regulator of telomere elongation helicase 1	-2.5806377	-3.0748453	-3.912257
RTN3	reticulon 3	1.4714985	1.7848296	2.2822547
RTTN	rotatin	1.0748202	-1.8275604	-3.7024896
RUNDC3B	RUN domain containing 3B	-1.1127056	4.4307137	6.547975
RUNX1	runt-related transcription factor 1	-2.9379957	-6.8660393	-8.577706
RUNX1T1	runt-related transcription factor 1; translocated to, 1 (cyclin D-related)	2.7557876	-1.2789216	-3.9016948
RUNX2	runt-related transcription factor 2	1.3195276	-2.4773886	-6.563897

RUSC1-AS1	RUSC1 antisense RNA 1	1.9500811	2.0006595	2.9085832
RWDD1	RWD domain containing 1	1.5189661	1.2664983	1.2181379
RXRA	retinoid X receptor, alpha	-1.0478582	1.7393024	2.1787484
RYK	receptor-like tyrosine kinase	1.6009375	-1.0345782	-1.0140151
S100A11	S100 calcium binding protein A11	-1.3041822	-2.344632	-8.162612
S100A11	S100 calcium binding protein A11	-1.1495898	-2.1410668	-7.401721
S100A13	S100 calcium binding protein A13	1.1231278	-1.1824	-2.7911413
S100A16	S100 calcium binding protein A16	-1.593593	-2.1066058	-2.7072299
S100A2	S100 calcium binding protein A2	1.3139844	-8.072749	-46.406925
S100A3	S100 calcium binding protein A3	-1.0846531	-3.0141277	-27.543798
S100A4	S100 calcium binding protein A4	1.689459	-1.0782202	-3.8848274
S100A8	S100 calcium binding protein A8	3.129639	4.505037	3.0040154
S100A9	S100 calcium binding protein A9	-1.1501279	3.1641576	-1.3592334
S1PR2	sphingosine-1-phosphate receptor 2	-2.33424	-3.2863295	-8.300289
S1PR3	sphingosine-1-phosphate receptor 3	-1.0025796	-1.5807168	-1.7810214
SAA1	serum amyloid A1	2.216389	10.228992	5.6872087
SAA2	serum amyloid A2	4.195646	28.895573	12.528652
SAA2	serum amyloid A2	1.8141922	14.993388	7.6329236
SAA3P	serum amyloid A3 pseudogene	3.9357064	52.734615	21.739925
SAA4	serum amyloid A4, constitutive	-1.4138948	12.63948	11.133908
SAAL1	serum amyloid A-like 1	1.1911659	-1.1729579	-1.9370742
SAC3D1	SAC3 domain containing 1	1.1346983	-1.5372447	-1.8465458
SACS	sacsin molecular chaperone	-1.9070169	-3.267948	-4.2146015
SAGE1	sarcoma antigen 1	-1.6705943	-1.467449	-1.1562945
SALL3	spalt-like transcription factor 3	1.7739699	1.5757313	1.5852847
SAMD11	sterile alpha motif domain containing 11	1.4122546	1.6111649	1.6606139
SAMD9L	sterile alpha motif domain containing 9-like	-1.392728	-2.0363889	-2.4457421
SAMHD1	SAM domain and HD domain 1	1.7735698	2.0557287	2.3200848
SAMM50	SAMM50 sorting and assembly machinery component	1.5728974	1.7597389	1.1868871
SAP30	Sin3A-associated protein, 30kDa	2.05337	1.0430568	1.3057413
SAP30L	SAP30-like	1.3537072	2.0413892	2.2432334
SAR1A	secretion associated, Ras related GTPase 1A	1.5909793	1.2251196	1.562159
SAR1B	secretion associated, Ras related GTPase 1B	-1.0093913	1.2863849	3.0070333
SARAF	store-operated calcium entry-associated regulatory factor	-1.0685123	1.0346735	1.8140234
SARDH	sarcosine dehydrogenase	-1.6943325	1.3319358	1.4711806
SARDH	sarcosine dehydrogenase	-1.4029402	1.4982945	1.7475973
SARS2	seryl-tRNA synthetase 2, mitochondrial	-1.0424987	-1.0615722	-1.585172
SAT1	spermidine/spermine N1-acetyltransferase 1	-2.333165	-1.5490787	1.2688655
SAT1	spermidine/spermine N1-acetyltransferase 1	-2.7993772	-1.7788233	1.1068374
SAT2	spermidine/spermine N1-acetyltransferase family member 2	1.0843834	1.7530824	1.8644016
SAYSD1	SAYSVFN motif domain containing 1	1.1569116	1.4304446	1.8067988
SBK2	SH3 domain binding kinase family, member 2	1.6659274	1.8562969	2.1398804
SBNO2	strawberry notch homolog 2 (Drosophila)	-1.1301495	-1.4590106	-2.2008548
SCAF11	SR-related CTD-associated factor 11	-1.1657723	-1.3548294	1.6719221
SCAMP1-AS1	SCAMP1 antisense RNA 1	1.368174	2.24172	1.3019036
SCAND1	SCAN domain containing 1	1.2561704	1.5559521	1.2782427
SCARA3	scavenger receptor class A, member 3	1.3019818	-1.966615	-1.8376255
SCARNA17	small Cajal body-specific RNA 17	-2.4211843	-1.1535819	1.0596874
SCCPDH	saccharopine dehydrogenase (putative)	1.2028383	2.0555384	3.5400376
SCGB1D1	secretoglobin, family 1D, member 1	-1.3540711	14.256525	11.184318
SCGB1D2	secretoglobin, family 1D, member 2	-1.340194	13.9227495	10.687653
SCGB3A1	secretoglobin, family 3A, member 1	1.8683622	1.9445604	2.429202
SCGN	secretagogin, EF-hand calcium binding protein	-5.966817	7.6859894	15.089449
SCHIP1	schwannomin interacting protein 1	1.782727	-1.2801081	-1.3722914
SCMH1	sex comb on midleg homolog 1 (Drosophila)	-1.1059926	-1.1743333	-2.6518087
SCN8A	sodium channel, voltage gated, type VIII alpha subunit	2.6672845	1.072365	-1.827339
SCNM1	sodium channel modifier 1	1.5878569	1.3490305	1.4016495
SCNN1A	sodium channel, non voltage gated 1 alpha subunit	5.2190723	4.9833536	3.3448582
SCP2	sterol carrier protein 2	-1.2318599	2.4466763	14.375591
SCP2	sterol carrier protein 2	1.0034329	1.4630371	4.830843
SCYL2	SCY1-like 2 (S. cerevisiae)	-1.0122179	1.078552	1.6633894
SCYL3	SCY1-like 3 (S. cerevisiae)	1.2665836	1.7017015	2.2124376
SDAD1	SDA1 domain containing 1	-1.584363	-1.8081182	-1.7652634
SDCBP	syndecan binding protein (syntenin)	-1.6337233	-2.1322486	-1.6422839
SDCCAG3	serologically defined colon cancer antigen 3	-1.2414291	-1.3477827	-1.8316346
SDF2L1	stromal cell-derived factor 2-like 1	1.4202486	1.8639544	1.3098973
SDHA	succinate dehydrogenase complex, subunit A, flavoprotein (Fp)	1.3928795	1.7936364	1.5884221
SDHC	succinate dehydrogenase complex, subunit C, integral membrane protein, 15kDa	2.073758	2.4171147	2.9315348
SDHC	succinate dehydrogenase complex, subunit C, integral membrane protein, 15kDa	1.3326641	1.6766577	2.2697368
SDHD	succinate dehydrogenase complex, subunit D, integral membrane protein	1.5078344	2.3327966	2.4048269
SDHD	succinate dehydrogenase complex, subunit D, integral membrane protein	2.004587	3.1722105	3.2077792
SDHD	succinate dehydrogenase complex, subunit D, integral membrane protein	1.5450048	2.431643	2.3831308
SDR39U1	short chain dehydrogenase/reductase family 39U, member 1	1.0509577	1.2664856	1.7151083
SDS	serine dehydratase	-1.5800799	2.688627	13.122218
SEC11C	SEC11 homolog C (S. cerevisiae)	-1.1685698	1.3321428	1.8937966
SEC14L1	SEC14-like 1 (S. cerevisiae)	1.7768173	1.5671345	1.4327685
SEC14L1	SEC14-like 1 (S. cerevisiae)	1.0209522	-1.9621178	-3.5680726
SEC14L1	SEC14-like 1 (S. cerevisiae)	1.3426552	-1.4428271	-2.4894567
SEC14L2	SEC14-like 2 (S. cerevisiae)	1.0259622	1.653421	2.0570133
SEC14L4	SEC14-like 4 (S. cerevisiae)	-1.2144438	1.0926824	-1.7603724
SEC16A	SEC16 homolog A (S. cerevisiae)	-2.2249331	-1.9340076	-1.8584989
SEC16B	SEC16 homolog B (S. cerevisiae)	-2.0516322	1.5392278	2.2311244
SEC31A	SEC31 homolog A (S. cerevisiae)	-1.4622264	-1.6967157	-1.9009572
SEC31B	SEC31 homolog B (S. cerevisiae)	-1.7666785	-1.0601538	2.0015686
SEC61A1	Sec61 alpha 1 subunit (S. cerevisiae)	-1.636566	-1.7487177	-2.2291398
SEH1L	SEH1-like (S. cerevisiae)	1.7789382	1.2945741	1.0900472
SEL1L	sel-1 suppressor of lin-12-like (C. elegans)	1.0777029	1.6844486	2.1484072
SEL1L	sel-1 suppressor of lin-12-like (C. elegans)	-2.0962722	-1.3915112	-1.2147409
SEL1L3	sel-1 suppressor of lin-12-like 3 (C. elegans)	-1.2143165	1.0112603	-1.618091
SELENBP1	selenium binding protein 1	1.2995863	3.9003696	15.041047
SELT	selenoprotein T	1.281936	1.4481286	1.8399601
SEMA3B	sema domain, immunoglobulin domain (Ig), short basic domain, secreted, (semaphorin) 3B	-1.1437056	-1.2363489	-3.3548224
SEMA4F	sema domain, immunoglobulin domain (Ig), transmembrane domain (TM) and short cytoplas	-1.1279516	-2.359173	-1.2822438
SEMA4G	sema domain, immunoglobulin domain (Ig), transmembrane domain (TM) and short cytoplas	-1.2540522	2.295698	3.1143525
SENP1	SUMO1/sentrin specific peptidase 1	-1.0833523	-1.5451442	-1.5855622
SENP3	SUMO1/sentrin/SMT3 specific peptidase 3	-1.3444546	-1.7285073	-2.0054305
SENP5	SUMO1/sentrin specific peptidase 5	-1.2280889	-1.5686823	-1.5807279
SEPHS2	selenophosphate synthetase 2	-1.0919359	2.0734754	4.907774
SEPN1	selenoprotein N, 1	-1.3378028	-1.8158634	-3.215515
SEPP1	selenoprotein P, plasma, 1	2.6573992	4.2224703	5.518384
SEPP1	selenoprotein P, plasma, 1	2.2705407	4.536428	5.3421364
SEPT7-AS1	SEPT7 antisense RNA 1 (head to head)	1.6613826	1.9403127	2.0857484
SEPW1	selenoprotein W, 1	-1.5514303	-1.708166	-1.8809086
SEPW1	selenoprotein W, 1	-1.2953401	-1.5749769	-1.7193056
SERBP1	SERPINE1 mRNA binding protein 1	-1.167636	-1.4936723	-1.685728
SERF1B	small EDRK-rich factor 1B (centromeric)	-1.0657883	-1.7446833	-2.3130152
SERF2	small EDRK-rich factor 2	1.3269588	2.2348754	-1.0140858
SERF2	small EDRK-rich factor 2	1.5628386	1.6337278	1.3934432
SERGEF	secretion regulating guanine nucleotide exchange factor	1.3215597	1.4511266	1.72647

SERINC1	serine incorporator 1	1.0286688	1.408486	1.9917631
SERINC3	serine incorporator 3	1.2947614	1.0850924	1.6122019
SERP1	stress-associated endoplasmic reticulum protein 1	1.1949985	1.7676556	1.8382192
SERPINA1	serpin peptidase inhibitor, clade A (alpha-1 antiproteinase, antitrypsin), member 1	-4.350659	1.3355529	1.70012
SERPINA10	serpin peptidase inhibitor, clade A (alpha-1 antiproteinase, antitrypsin), member 10	-7.4255114	6.341835	13.993089
SERPINA2	serpin peptidase inhibitor, clade A (alpha-1 antiproteinase, antitrypsin), member 2 (gene/pseu	-1.6164888	4.842818	6.611801
SERPINA3	serpin peptidase inhibitor, clade A (alpha-1 antiproteinase, antitrypsin), member 3	-3.3086915	1.9109617	1.5593424
SERPINA3	serpin peptidase inhibitor, clade A (alpha-1 antiproteinase, antitrypsin), member 3	-2.804409	2.1409488	1.8491609
SERPINA3	serpin peptidase inhibitor, clade A (alpha-1 antiproteinase, antitrypsin), member 3	-1.6757919	3.2141745	2.9421172
SERPINA4	serpin peptidase inhibitor, clade A (alpha-1 antiproteinase, antitrypsin), member 4	-1.7841222	8.735052	9.415482
SERPINA5	serpin peptidase inhibitor, clade A (alpha-1 antiproteinase, antitrypsin), member 5	-1.1483749	2.0974655	1.6097934
SERPINA6	serpin peptidase inhibitor, clade A (alpha-1 antiproteinase, antitrypsin), member 6	-4.2888327	1.8217833	4.106047
SERPINA7	serpin peptidase inhibitor, clade A (alpha-1 antiproteinase, antitrypsin), member 7	-8.416345	4.8809857	4.057233
SERPINB6	serpin peptidase inhibitor, clade B (ovalbumin), member 6	-1.3520797	-1.3141068	-2.1001353
SERPINB6	serpin peptidase inhibitor, clade B (ovalbumin), member 6	1.0067782	1.0170722	-1.5122749
SERPINC1	serpin peptidase inhibitor, clade C (antithrombin), member 1	-1.7322402	50.712063	194.83199
SERPIND1	serpin peptidase inhibitor, clade D (heparin cofactor), member 1	-23.770702	2.5594432	18.113253
SERPINE1	serpin peptidase inhibitor, clade E (nexin, plasminogen activator inhibitor type 1), member 1	-2.0570714	-16.971106	-14.904331
SERPINF1	serpin peptidase inhibitor, clade F (alpha-2 antiplasmin, pigment epithelium derived factor), m	-2.0025637	1.2999365	2.9638948
SERPINF2	serpin peptidase inhibitor, clade F (alpha-2 antiplasmin, pigment epithelium derived factor), m	-2.1087828	9.511162	29.385962
SERPING1	serpin peptidase inhibitor, clade G (C1 inhibitor), member 1	-2.7008896	2.952823	5.082266
SERPING1	serpin peptidase inhibitor, clade G (C1 inhibitor), member 1	-2.7870126	2.7580404	5.620823
SERPING1	serpin peptidase inhibitor, clade G (C1 inhibitor), member 1	-3.2920523	2.1656408	3.959912
SERPINH1	serpin peptidase inhibitor, clade H (heat shock protein 47), member 1, (collagen binding prote	1.069721	-1.6952834	-3.7564685
SERTAD4-AS1	SERTAD4 antisense RNA 1	2.4968417	1.1520106	-1.3439883
SET	SET nuclear proto-oncogene	2.3255785	1.5173889	1.466776
SETBP1	SET binding protein 1	-1.5257871	1.1460162	1.1839529
SETD8	SET domain containing (lysine methyltransferase) 8	-1.0075109	-1.3253651	-1.516302
SF3A1	splicing factor 3a, subunit 1, 120kDa	1.8635402	1.8416425	1.7747486
SF3B2	splicing factor 3b, subunit 2, 145kDa	-1.5013283	-1.6184783	-1.6172334
SFI1	Sfi1 homolog, spindle assembly associated (yeast)	-1.4996228	-1.9532106	-1.7233063
SFMBT1	Scm-like with four mbt domains 1	1.5374783	1.204099	1.8195246
SFN	stratifin	1.0686688	-2.4298713	-7.982024
SFPQ	splicing factor proline/glutamine-rich	1.6745341	-1.187472	-1.4354413
SFR1	SWI5-dependent recombination repair 1	-1.6007947	-1.8982062	-2.3792908
SFTPA2	surfactant protein A2	1.7780459	1.8912358	1.9399841
SFXN2	sideroflexin 2	1.3636367	2.0222087	2.8504171
SFXN4	sideroflexin 4	1.191699	1.5613828	-1.2574115
SFXN5	sideroflexin 5	2.6255639	2.38002	2.9495208
SFXN5	sideroflexin 5	-1.3851964	1.3488628	1.5881041
SGK1	serum/glucocorticoid regulated kinase 1	4.299962	2.816461	2.0910685
SGK2	serum/glucocorticoid regulated kinase 2	-2.7886167	1.1646919	1.2069113
SGK223	homolog of rat pragma of Rnd2	-1.479833	-3.1342216	-2.3551295
SGSH	N-sulfoglucosamine sulfohydrolase	-1.0494225	-1.1901762	-1.8070915
SGSM2	small G protein signaling modulator 2	-1.7521582	-1.6502855	-1.9651779
SGSM3	small G protein signaling modulator 3	-1.5652113	-1.1079704	1.3265873
SH2B2	SH2B adaptor protein 2	-1.0781249	-1.5029685	-3.0138175
SH2D3A	SH2 domain containing 3A	-1.2379444	-1.3467437	-2.3406181
SH2D5	SH2 domain containing 5	1.3323708	-1.1228523	-1.732131
SH3BGR	SH3 domain binding glutamate-rich protein	1.1371249	2.2663581	2.0390522
SH3BGRL3	SH3 domain binding glutamate-rich protein like 3	-1.3411041	-1.2559781	-2.1208353
SH3D19	SH3 domain containing 19	1.1682489	1.9775196	2.294974
SH3KBP1	SH3-domain kinase binding protein 1	-3.0840328	-5.047166	-4.457949
SH3KBP1	SH3-domain kinase binding protein 1	-2.753143	-5.1268	-4.2557254
SH3PXD2A	SH3 and PX domains 2A	1.1257074	-1.1465868	3.5231254
SH3RF1	SH3 domain containing ring finger 1	-1.0916264	-1.371218	-1.974265
SH3TC1	SH3 domain and tetratricopeptide repeats 1	-1.1240052	-1.3199602	-2.923575
SH3TC1	SH3 domain and tetratricopeptide repeats 1	3.2902536	2.2312756	2.1188474
SHANK2-AS3	SHANK2 antisense RNA 3	2.1118789	2.5070968	2.2046416
SHC1	SHC (Src homology 2 domain containing) transforming protein 1	-2.0384252	-1.6816298	-2.1036308
SHC2	SHC (Src homology 2 domain containing) transforming protein 2	-2.3524122	-1.2353162	-1.4224058
SHD	Src homology 2 domain containing transforming protein D	2.7196653	2.4368598	4.1170726
SHFM1	split hand/foot malformation (ectrodactyly) type 1	1.6967489	1.4161859	1.6517755
SHISA4	shisa family member 4	1.6795775	1.9465764	2.3667488
SHISA5	shisa family member 5	-1.8053123	-1.7868973	-1.6153424
SHISA5	shisa family member 5	2.4142697	2.1341178	1.9740434
SHISA7	shisa family member 7	1.9522396	1.7408799	1.8822857
SHISA9	shisa family member 9	4.7082934	2.7040942	1.4835128
SHMT1	serine hydroxymethyltransferase 1 (soluble)	-1.0321425	3.4145372	7.42678
SHMT1	serine hydroxymethyltransferase 1 (soluble)	-1.0631956	2.9913962	5.291749
SHMT2	serine hydroxymethyltransferase 2 (mitochondrial)	-1.2920119	1.1373566	1.9280696
SHPK	sedoheptulokinase	-1.1239029	1.6814123	3.0567298
SHROOM3	shroom family member 3	2.0346186	2.0283065	1.0214812
SIAE	sialic acid acetylesterase	-1.6044852	1.5711254	1.6405004
SIAH1	siah E3 ubiquitin protein ligase 1	-1.7832675	-1.5036122	-1.570928
SIAH2	siah E3 ubiquitin protein ligase 2	1.2929587	1.3108661	1.7487199
SIDT2	SID1 transmembrane family, member 2	1.3654399	1.8958683	3.6872268
SIGLEC14	sialic acid binding Ig-like lectin 14	1.5098627	1.57792	1.8593907
SIGLEC15	sialic acid binding Ig-like lectin 15	2.2713943	2.437573	2.782116
SIK3	SIK family kinase 3	1.2506907	1.317142	1.7135949
SIKE1	suppressor of IKBKE 1	1.5594324	1.2145708	1.1186177
SIL1	SIL1 nucleotide exchange factor	1.0137647	-1.6150259	-1.6338707
SIMC1	SUMO-interacting motifs containing 1	1.6692961	1.7641859	1.7540689
SIPA1L1	signal-induced proliferation-associated 1 like 1	-1.5173755	-1.4235659	1.4769307
SIPA1L2	signal-induced proliferation-associated 1 like 2	-1.5758629	-2.010887	-1.597765
SIRPB1	signal-regulatory protein beta 1	7.4149523	9.062489	10.713554
SIRT3	sirtuin 3	1.2089034	2.2849314	2.7860196
SIRT5	sirtuin 5	1.0992943	1.5697477	1.0295984
SIRT5	sirtuin 5	1.5049508	2.0777628	2.1689723
SIRT7	sirtuin 7	-1.7597258	-1.6362109	-1.1271782
SIRT7	sirtuin 7	-2.196638	-1.8555094	-1.3268952
SKA2	spindle and kinetochore associated complex subunit 2	2.158664	-1.3754581	-1.3824775
SKIV2L	superkiller viralicidic activity 2-like (S. cerevisiae)	-1.5558251	-1.4175187	-1.0508183
SLC10A1	solute carrier family 10 (sodium/bile acid cotransporter), member 1	-1.808789	19.261826	32.714806
SLC10A3	solute carrier family 10, member 3	1.2221264	-1.0252426	-1.9552364
SLC12A4	solute carrier family 12 (potassium/chloride transporter), member 4	-1.1838475	-1.2229773	-1.8788512
SLC12A6	solute carrier family 12 (potassium/chloride transporter), member 6	2.6431832	2.9170954	3.2122757
SLC16A12	solute carrier family 16, member 12	4.650394	7.853331	-1.0809457
SLC16A2	solute carrier family 16, member 2 (thyroid hormone transporter)	-1.2999253	1.1122873	2.207409
SLC16A3	solute carrier family 16 (monocarboxylate transporter), member 3	2.3649838	1.3522269	-1.6159395
SLC17A4	solute carrier family 17, member 4	-10.89484	9.976973	13.545348
SLC17A9	solute carrier family 17 (vesicular nucleotide transporter), member 9	-1.3928481	1.3855517	-1.6235474
SLC18A3	solute carrier family 18 (vesicular acetylcholine transporter), member 3	1.7142228	1.9450982	1.936722
SLC19A1	solute carrier family 19 (folate transporter), member 1	-1.0174707	-1.7885848	-3.3400965
SLC1A1	solute carrier family 1 (neuronal/epithelial high affinity glutamate transporter, system Xag), m	1.8188398	3.1539595	3.378134
SLC1A3	solute carrier family 1 (glial high affinity glutamate transporter), member 3	-1.0917277	-1.1988814	-2.0619175
SLC20A2	solute carrier family 20 (phosphate transporter), member 2	-1.0536364	1.0642264	1.8302293
SLC22A1	solute carrier family 22 (organic cation transporter), member 1	-1.3053917	11.481134	98.256546
SLC22A18	solute carrier family 22, member 18	-1.4269361	2.1765246	3.4180856

SLC22A18AS	solute carrier family 22 (organic cation transporter), member 18 antisense	-1.101032	2.8828135	3.4892247
SLC22A23	solute carrier family 22, member 23	2.6294472	1.7995074	2.337593
SLC22A23	solute carrier family 22, member 23	-1.8732666	-1.0928704	2.5267649
SLC22A31	solute carrier family 22, member 31	2.560413	2.4158578	2.7819543
SLC22A7	solute carrier family 22 (organic anion transporter), member 7	-2.0218892	10.7700405	36.452766
SLC22A7	solute carrier family 22 (organic anion transporter), member 7	1.1747532	3.7302845	9.881121
SLC23A3	solute carrier family 23, member 3	1.3743241	7.7586327	4.34903
SLC25A1	solute carrier family 25 (mitochondrial carrier; citrate transporter), member 1	1.095794	1.9624907	2.8709753
SLC25A10	solute carrier family 25 (mitochondrial carrier; dicarboxylate transporter), member 10	1.338937	1.8450674	2.7171407
SLC25A11	solute carrier family 25 (mitochondrial carrier; oxoglutarate carrier), member 11	1.6438897	2.4242136	2.2027435
SLC25A13	solute carrier family 25 (aspartate/glutamate carrier), member 13	1.0608811	1.1282179	1.9794168
SLC25A15	solute carrier family 25 (mitochondrial carrier; ornithine transporter) member 15	1.3153911	1.8070468	1.8549193
SLC25A16	solute carrier family 25 (mitochondrial carrier), member 16	-3.1335154	-2.8088195	-1.905514
SLC25A18	solute carrier family 25 (glutamate carrier), member 18	-1.902194	1.1756053	-1.3288044
SLC25A20	solute carrier family 25 (carnitine/acylcarnitine translocase), member 20	-1.0549455	1.8319917	2.2890089
SLC25A22	solute carrier family 25 (mitochondrial carrier: glutamate), member 22	1.5515262	2.1540234	1.8134736
SLC25A24	solute carrier family 25 (mitochondrial carrier; phosphate carrier), member 24	1.873731	1.4418555	1.2976379
SLC25A25	solute carrier family 25 (mitochondrial carrier; phosphate carrier), member 25	-1.0948589	-1.1579698	3.0931213
SLC25A29	solute carrier family 25 (mitochondrial carnitine/acylcarnitine carrier), member 29	-1.6018707	-1.4230505	-1.3405741
SLC25A3	solute carrier family 25 (mitochondrial carrier; phosphate carrier), member 3	1.6110805	1.7816254	1.3269194
SLC25A30	solute carrier family 25, member 30	1.4049884	1.4978731	2.1808097
SLC25A32	solute carrier family 25 (mitochondrial folate carrier), member 32	1.7337888	1.2187566	1.1267829
SLC25A33	solute carrier family 25 (pyrimidine nucleotide carrier), member 33	-1.0743562	1.3228137	4.1655974
SLC25A38	solute carrier family 25, member 38	1.4420902	1.574045	2.620011
SLC25A39	solute carrier family 25, member 39	-1.9195677	-1.545797	-1.6535798
SLC25A3P1	solute carrier family 25 (mitochondrial carrier; phosphate carrier), member 3 pseudogene 1	3.2614288	3.4339285	3.9853888
SLC25A4	solute carrier family 25 (mitochondrial carrier; adenine nucleotide translocator), member 4	1.2645558	2.942831	2.6497784
SLC25A42	solute carrier family 25, member 42	-1.2649488	2.0393999	4.0083995
SLC25A44	solute carrier family 25, member 44	1.4260623	1.1962836	2.5178235
SLC25A5	solute carrier family 25 (mitochondrial carrier; adenine nucleotide translocator), member 5	2.148518	2.3551426	1.5318265
SLC25A51	solute carrier family 25, member 51	-3.2186522	-2.9743102	-2.2430613
SLC25A6	solute carrier family 25 (mitochondrial carrier; adenine nucleotide translocator), member 6	1.6541319	1.5063533	-1.4465446
SLC25A6	solute carrier family 25 (mitochondrial carrier; adenine nucleotide translocator), member 6	1.5998741	1.468418	-1.5785735
SLC26A2	solute carrier family 26 (anion exchanger), member 2	3.4318497	1.161998	-2.1289167
SLC26A6	solute carrier family 26 (anion exchanger), member 6	-1.8660382	-1.3752201	-1.1102333
SLC27A1	solute carrier family 27 (fatty acid transporter), member 1	1.3268179	1.3758557	1.5422219
SLC27A2	solute carrier family 27 (fatty acid transporter), member 2	1.6437849	2.6282804	9.706769
SLC27A3	solute carrier family 27 (fatty acid transporter), member 3	1.0505366	3.3637047	1.8421309
SLC27A5	solute carrier family 27 (fatty acid transporter), member 5	-2.1967864	38.845703	204.32051
SLC28A1	solute carrier family 28 (concentrative nucleoside transporter), member 1	-8.133792	9.008162	24.013035
SLC29A1	solute carrier family 29 (equilibrative nucleoside transporter), member 1	-2.8064313	-4.428244	-14.703032
SLC29A3	solute carrier family 29 (equilibrative nucleoside transporter), member 3	1.5901564	-1.084072	1.0477352
SLC2A10	solute carrier family 2 (facilitated glucose transporter), member 10	-1.6012208	-1.08441	-1.1559803
SLC2A2	solute carrier family 2 (facilitated glucose transporter), member 2	-1.1235527	8.500055	12.809347
SLC2A4RG	SLC2A4 regulator	1.2589499	1.3161837	2.1073575
SLC2A6	solute carrier family 2 (facilitated glucose transporter), member 6	-1.4213092	-2.2327456	-4.169383
SLC2A6	solute carrier family 2 (facilitated glucose transporter), member 6	-2.8512294	-2.4531095	-2.1944606
SLC2A8	solute carrier family 2 (facilitated glucose transporter), member 8	1.1040472	1.3494704	1.9546982
SLC2A8	solute carrier family 2 (facilitated glucose transporter), member 8	-1.3629448	1.0147482	1.5230616
SLC30A10	solute carrier family 30, member 10	-3.6508377	3.2665973	10.494939
SLC30A3	solute carrier family 30 (zinc transporter), member 3	1.0374101	-1.6820804	-3.0831063
SLC30A7	solute carrier family 30 (zinc transporter), member 7	-1.6851166	-1.3377929	-1.5023929
SLC31A1	solute carrier family 31 (copper transporter), member 1	-1.3760784	1.068311	2.5066476
SLC31A1	solute carrier family 31 (copper transporter), member 1	-1.5289413	-1.1347662	2.1656764
SLC31A2	solute carrier family 31 (copper transporter), member 2	1.1410582	1.6056539	2.3589497
SLC35A3	solute carrier family 35 (UDP-N-acetylglucosamine (UDP-GlcNAc) transporter), member A3	1.0374733	1.2710024	2.5337033
SLC35B2	solute carrier family 35 (adenosine 3'-phospho 5'-phosphosulfate transporter), member B2	1.0616415	-1.1966561	-1.8056769
SLC35D1	solute carrier family 35 (UDP-GlcA/UDP-GalNAc transporter), member D1	1.5314987	1.7636696	3.36645
SLC35E1	solute carrier family 35, member E1	1.389095	1.4378419	1.6679814
SLC35E2	solute carrier family 35, member E2	-1.5435728	-1.1085297	-1.0239872
SLC35E2	solute carrier family 35, member E2	-1.6872557	-1.1156814	-1.4425288
SLC35E2B	solute carrier family 35, member E2B	1.0609909	1.6645412	2.9753926
SLC35E4	solute carrier family 35, member E4	-2.463433	-1.8938909	-1.7326406
SLC35F2	solute carrier family 35, member F2	1.432859	1.0919793	-1.6889794
SLC36A1	solute carrier family 36 (proton/amino acid symporter), member 1	-1.2080224	-1.8011956	-1.3696996
SLC37A2	solute carrier family 37 (glucose-6-phosphate transporter), member 2	-3.1087747	-11.772882	-34.778065
SLC37A4	solute carrier family 37 (glucose-6-phosphate transporter), member 4	1.1045938	3.3282654	5.8757505
SLC38A1	solute carrier family 38, member 1	-1.1745499	-1.5835779	-2.3986397
SLC38A1	solute carrier family 38, member 1	1.0555546	-1.2592753	-2.1197093
SLC38A2	solute carrier family 38, member 2	-1.167556	-2.067282	-2.2439182
SLC38A3	solute carrier family 38, member 3	-1.5054847	4.256264	21.038631
SLC38A7	solute carrier family 38, member 7	-1.3732321	-1.7741995	-1.9973671
SLC38A9	solute carrier family 38, member 9	-1.312122	-1.1662326	2.0694928
SLC39A1	solute carrier family 39 (zinc transporter), member 1	-1.162745	-1.3921567	-3.4290328
SLC39A11	solute carrier family 39, member 11	-1.0034019	1.2204446	3.6094453
SLC39A13	solute carrier family 39 (zinc transporter), member 13	1.3261465	-1.2200863	-1.637536
SLC39A14	solute carrier family 39 (zinc transporter), member 14	-1.4256858	1.277858	1.657299
SLC39A4	solute carrier family 39 (zinc transporter), member 4	1.5489049	1.5874053	1.9835525
SLC39A5	solute carrier family 39 (zinc transporter), member 5	-2.8900058	13.805403	40.023987
SLC39A8	solute carrier family 39 (zinc transporter), member 8	1.6997526	1.3673633	1.3511823
SLC3A2	solute carrier family 3 (amino acid transporter heavy chain), member 2	-2.0186028	-2.9715235	-2.6482844
SLC40A1	solute carrier family 40 (iron-regulated transporter), member 1	-1.3248483	2.086677	3.6915379
SLC41A1	solute carrier family 41 (magnesium transporter), member 1	1.6424451	1.55844	1.469026
SLC41A2	solute carrier family 41 (magnesium transporter), member 2	-1.33813	1.1325425	2.4564257
SLC41A3	solute carrier family 41, member 3	1.9949508	2.225788	1.4477366
SLC43A1	solute carrier family 43 (amino acid system L transporter), member 1	-1.6506951	1.465451	1.8266803
SLC43A3	solute carrier family 43, member 3	-1.6370691	-1.8801811	-4.533502
SLC44A1	solute carrier family 44 (choline transporter), member 1	-1.7178103	1.3015429	2.9603193
SLC44A2	solute carrier family 44 (choline transporter), member 2	-1.0885009	-1.93424	-2.3197687
SLC46A1	solute carrier family 46 (folate transporter), member 1	1.6816125	2.9240093	8.546611
SLC47A1	solute carrier family 47 (multidrug and toxin extrusion), member 1	1.1523201	1.3919597	10.010812
SLC4A2	solute carrier family 4 (anion exchanger), member 2	-1.8683698	-1.9934894	-3.3929918
SLC50A1	solute carrier family 50 (sugar efflux transporter), member 1	-1.899726	-1.4050866	1.0122759
SLC52A2	solute carrier family 52 (riboflavin transporter), member 2	1.175932	1.0120589	-2.1705809
SLC5A3	solute carrier family 5 (sodium/myo-inositol cotransporter), member 3	-2.4060047	-3.6832335	-2.5376537
SLC5A6	solute carrier family 5 (sodium/multivitamin and iodide cotransporter), member 6	1.7270492	1.6034198	1.6515021
SLC6A16	solute carrier family 6, member 16	1.2732795	2.7656279	2.265109
SLC6A3	solute carrier family 6 (neurotransmitter transporter), member 3	1.7669775	3.122406	5.2100687
SLC6A6	solute carrier family 6 (neurotransmitter transporter), member 6	-1.4560887	-4.1777663	-4.0904207
SLC6A6	solute carrier family 6 (neurotransmitter transporter), member 6	3.0360563	3.3813798	3.8821108
SLC7A11	solute carrier family 7 (anionic amino acid transporter light chain, xc- system), member 11	-3.9302382	-13.179388	-10.788265
SLC7A5P1	solute carrier family 7 (amino acid transporter light chain, L system), member 5 pseudogene 1	1.7403421	1.8971	2.1868942
SLC7A7	solute carrier family 7 (amino acid transporter light chain, y+L system), member 7	2.803408	2.0409176	-1.5330316
SLC9A3R2	solute carrier family 9, subfamily A (NHE3, cation proton antiporter 3), member 3 regulator 2	-1.9766527	-1.5319843	-1.359068
SLCO1B1	solute carrier organic anion transporter family, member 1B1	-7.5478034	2.7286785	12.508953
SLCO2B1	solute carrier organic anion transporter family, member 2B1	-6.6111608	6.6727624	17.888638
SLCO4A1-AS1	SLCO4A1 antisense RNA 1	1.8496791	1.9817048	2.111247
SLFN5	schlafen family member 5	-3.7049403	-3.0669003	-2.5793955
SLIRP	SRA stem-loop interacting RNA binding protein	1.630568	1.575648	1.3051294

SLPI	secretory leukocyte peptidase inhibitor	1.9389421	9.043225	2.3345978
SLX4IP	SLX4 interacting protein	-1.8905771	-1.3442557	-1.6468722
SMA4	glucuronidase, beta pseudogene	-2.2182956	-1.5755607	-1.4717109
SMAD3	SMAD family member 3	-1.5971657	-2.1791492	-2.3414042
SMAP1	small ArfGAP 1	-1.1637366	-1.0374907	-1.6641269
SMAP2	small ArfGAP2	-1.117631	1.3710387	2.4417903
SMARCA4	SWI/SNF related, matrix associated, actin dependent regulator of chromatin, subfamily a, mer	-1.0366464	-1.6034216	-2.4276907
SMARCA5	SWI/SNF related, matrix associated, actin dependent regulator of chromatin, subfamily a, mer	1.0520191	-1.3513302	-1.5367419
SMARCC1	SWI/SNF related, matrix associated, actin dependent regulator of chromatin, subfamily c, mer	-2.0011706	-3.058976	-3.6731095
SMARCD2	SWI/SNF related, matrix associated, actin dependent regulator of chromatin, subfamily d, mer	1.4872522	2.0546424	1.718029
SMARCD3	SWI/SNF related, matrix associated, actin dependent regulator of chromatin, subfamily d, mer	1.4040523	1.5692666	1.0870802
SMC1A	structural maintenance of chromosomes 1A	-1.919826	-2.6558046	-1.9856088
SMC4	structural maintenance of chromosomes 4	1.3193622	-1.3761228	-3.9106894
SMC4	structural maintenance of chromosomes 4	1.1339977	-1.3068424	-3.1362405
SMC6	structural maintenance of chromosomes 6	-1.4846038	-2.161162	-1.8103569
SMCR2	Smith-Magenis syndrome chromosome region, candidate 2 (non-protein coding)	1.6109823	1.8829306	3.3845751
SMG1	SMG1 phosphatidylinositol 3-kinase-related kinase	-1.1905327	-1.3963404	-2.0962443
SMG5	SMG5 nonsense mediated mRNA decay factor	-1.4670689	-1.5915753	-1.7495811
SMIM1	small integral membrane protein 1 (Vel blood group)	1.0394793	1.8660136	-1.0602834
SMIM12	small integral membrane protein 12	1.9818746	2.006799	1.3364757
SMIM14	small integral membrane protein 14	-1.543154	1.5338691	2.5357888
SMIM19	small integral membrane protein 19	-1.2391104	1.6413269	2.2158859
SMIM2-AS1	SMIM2 antisense RNA 1	-1.3773283	2.214496	2.0741563
SMIM2-AS1	SMIM2 antisense RNA 1	-1.6757507	2.1834512	1.7841811
SMIM4	small integral membrane protein 4	2.0950696	2.2171996	1.6133537
SMIM7	small integral membrane protein 7	1.5526754	1.2529967	1.4785098
SMIM7	small integral membrane protein 7	1.11897	1.0946753	1.5101703
SMLR1	small leucine-rich protein 1	-1.7305151	8.208442	15.157807
SMOC1	SPARC related modular calcium binding 1	-3.1856263	1.9618473	2.3964856
SMOC1	SPARC related modular calcium binding 1	-2.6445448	1.2417889	2.4793272
SMOX	spermine oxidase	-2.293305	-2.5403297	-2.5967128
SMPD4	sphingomyelin phosphodiesterase 4, neutral membrane (neutral sphingomyelinase-3)	-1.1056565	-1.7699097	-1.4099935
SMPDL3A	sphingomyelin phosphodiesterase, acid-like 3A	-1.0904276	3.191249	5.238718
SMPX	small muscle protein, X-linked	-3.002003	-1.1973898	1.8039954
SMS	spermine synthase	-1.3017191	-2.458684	-2.328596
SMYD3	SET and MYND domain containing 3	2.0485172	1.1536632	-1.6968439
SMYD4	SET and MYND domain containing 4	1.3974257	1.5031803	1.7561928
SMYD5	SMYD family member 5	-1.2750874	-1.2766433	-1.6541448
SNAI3-AS1	SNAI3 antisense RNA 1	1.3547846	1.4074706	3.1531935
SNAP47	synaptosomal-associated protein, 47kDa	1.4286329	1.5937598	1.7893986
SNAPC1	small nuclear RNA activating complex, polypeptide 1, 43kDa	1.703814	-1.4769742	-2.1035414
SNAPC5	small nuclear RNA activating complex, polypeptide 5, 19kDa	-1.0434134	1.071855	1.7246356
SNAPC5	small nuclear RNA activating complex, polypeptide 5, 19kDa	1.0082256	1.1617014	1.9085529
SNAPIN	SNAP-associated protein	1.471214	1.4353739	1.6066891
SNF8	SNF8, ESCRT-II complex subunit	1.5095466	1.2457062	1.3549796
SNHG11	small nucleolar RNA host gene 11 (non-protein coding)	1.1420289	1.5484415	1.2069713
SNHG11	small nucleolar RNA host gene 11 (non-protein coding)	1.3973335	1.8676507	1.5494525
SNHG12	small nucleolar RNA host gene 12 (non-protein coding)	-2.2418633	-2.949431	-2.6267815
SNHG15	small nucleolar RNA host gene 15 (non-protein coding)	-1.0349617	-1.5939758	-1.8119353
SNHG17	small nucleolar RNA host gene 17 (non-protein coding)	1.53434	1.3346313	-1.3125916
SNHG6	small nucleolar RNA host gene 6 (non-protein coding)	1.2447852	-1.0218625	-1.60113
SNHG7	small nucleolar RNA host gene 7 (non-protein coding)	1.1832764	1.5745972	1.3567884
SNHG8	small nucleolar RNA host gene 8 (non-protein coding)	1.1319906	1.5974228	1.2732801
SNORA10	small nucleolar RNA, H/ACA box 10	-1.1135262	-2.260773	-2.3791447
SNORA71A	small nucleolar RNA, H/ACA box 71A	2.7988958	2.7507863	3.196467
SNORA73A	small nucleolar RNA, H/ACA box 73A	1.7068484	3.8587108	3.9755485
SNORA73B	small nucleolar RNA, H/ACA box 73B	1.3083968	2.5338326	2.7933218
SNORA73B	small nucleolar RNA, H/ACA box 73B	1.8242474	4.2522273	4.3181963
SNORD116-19	small nucleolar RNA, C/D box 116-19	1.3950301	1.7962445	-1.2947695
SNORD15A	small nucleolar RNA, C/D box 15A	3.0081499	7.683933	8.327596
SNRNP70	small nuclear ribonucleoprotein 70kDa (U1)	-1.1013277	-1.298006	-1.663765
SNRPA	small nuclear ribonucleoprotein polypeptide A	1.1834499	-1.4764081	-1.6935905
SNRPA1	small nuclear ribonucleoprotein polypeptide A'	-1.0769246	-1.7942895	-1.6741272
SNRPB	small nuclear ribonucleoprotein polypeptides B and B1	1.2338811	-1.3203876	-1.5916994
SNRPB2	small nuclear ribonucleoprotein polypeptide B	1.8340122	1.3545281	1.1095315
SNRPC	small nuclear ribonucleoprotein polypeptide C	2.2389388	1.848088	1.578266
SNRPD1	small nuclear ribonucleoprotein D1 polypeptide 16kDa	1.7241516	-1.0390321	-1.4341822
SNRPD1	small nuclear ribonucleoprotein D1 polypeptide 16kDa	1.5573074	-1.1441429	-1.6547384
SNRPD2P2	small nuclear ribonucleoprotein D2 pseudogene 2	4.3171186	2.760395	2.4074836
SNRPD3	small nuclear ribonucleoprotein D3 polypeptide 18kDa	1.7216176	1.2412219	1.5125538
SNRPE	small nuclear ribonucleoprotein polypeptide E	2.2191734	1.8597981	1.4182901
SNRPE	small nuclear ribonucleoprotein polypeptide E	2.0963392	1.6278296	1.3992276
SNRPE	small nuclear ribonucleoprotein polypeptide E	1.823418	1.4522648	1.169904
SNRPF	small nuclear ribonucleoprotein polypeptide F	2.1569805	1.241266	1.3179556
SNTB1	syntrophin, beta 1 (dystrophin-associated protein A1, 59kDa, basic component 1)	-1.701853	1.3098835	3.279155
SNTB2	syntrophin, beta 2 (dystrophin-associated protein A1, 59kDa, basic component 2)	1.7614442	1.1025875	-1.0332612
SNTB2	syntrophin, beta 2 (dystrophin-associated protein A1, 59kDa, basic component 2)	1.152457	-1.6347209	-1.7691736
SNX1	sorting nexin 1	1.217458	1.5706129	1.1357956
SNX2	sorting nexin 2	1.5872076	1.2866852	1.3243016
SNX24	sorting nexin 24	1.7837611	1.480506	1.5360043
SNX4	sorting nexin 4	1.5096503	1.884063	2.7799525
SNX6	sorting nexin 6	2.0203063	1.8285637	1.4516182
SNX8	sorting nexin 8	-1.1011931	-1.5623313	-2.6198041
SNX8	sorting nexin 8	1.0866797	-1.2013723	-1.7190073
SOBP	sine oculis binding protein homolog (Drosophila)	1.1186621	1.3232081	2.06355
SOCS2	suppressor of cytokine signaling 2	2.133756	-1.0664314	-1.3201275
SOD1	superoxide dismutase 1, soluble	1.1726093	1.2274631	1.659405
SOD2	superoxide dismutase 2, mitochondrial	-1.9937556	1.0370243	-2.9731884
SOD3	superoxide dismutase 3, extracellular	1.0915691	2.3867073	1.3715254
SOHLH2	spermatogenesis and oogenesis specific basic helix-loop-helix 2	1.6019713	1.0751418	-1.776299
SOHLH2	spermatogenesis and oogenesis specific basic helix-loop-helix 2	1.4263489	1.0600528	-1.7491736
SORBS1	sorbin and SH3 domain containing 1	-1.0258362	4.5616875	7.1283083
SORBS2	sorbin and SH3 domain containing 2	-4.017296	1.8076246	2.1939662
SORBS3	sorbin and SH3 domain containing 3	1.580346	1.7888482	2.0045733
SORD	sorbitol dehydrogenase	-1.34676	1.1670227	3.4715168
SORL1	sortilin-related receptor, L(DLR class) A repeats containing	-2.8288624	1.2964125	1.8979291
SORT1	sortilin 1	2.4480584	2.1477494	1.804994
SOWAHA	sosondowah ankyrin repeat domain family member A	-1.1032486	5.2524953	13.975751
SOWAHD	sosondowah ankyrin repeat domain family member D	7.1870484	4.4639134	2.4328651
SP5	Sp5 transcription factor	2.121524	2.1133378	1.7304771
SP7	Sp7 transcription factor	1.3105341	1.3897233	1.6744756
SP8	Sp8 transcription factor	2.419778	2.6256182	3.3360026
SP9	Sp9 transcription factor	-1.1518658	1.4703492	2.130009
SPA17	sperm autoantigenic protein 17	-1.3740991	-2.489396	-2.0798376
SPAG5	sperm associated antigen 5	1.8310548	-2.982464	-2.6462069
SPANXA1	sperm protein associated with the nucleus, X-linked, family member A1	2.2456856	-1.4530123	-5.838204
SPANXB1	SPANX family, member B1	1.4341886	-1.6979777	-4.6439734
SPANXC	SPANX family, member C	1.6054047	-1.6235554	-4.5233154
SPARC	secreted protein, acidic, cysteine-rich (osteonectin)	1.8497189	-1.759863	-3.7379744

SPATA13	spermatogenesis associated 13	-4.1200147	-2.2378654	-2.3889031
SPATA18	spermatogenesis associated 18	-1.0966346	-1.3011426	-2.3865635
SPATA21	spermatogenesis associated 21	1.3205131	2.031153	1.773841
SPATA2L	spermatogenesis associated 2-like	-1.1199892	1.4588728	1.9454882
SPATA5L1	spermatogenesis associated 5-like 1	1.1716942	1.2332907	1.5690659
SPATS2	spermatogenesis associated, serine-rich 2	-1.0659769	-1.4331075	-1.9639453
SPATS2L	spermatogenesis associated, serine-rich 2-like	-2.3760705	-3.340594	-5.719063
SPC24	SPC24, NDC80 kinetochore complex component	1.9372025	-3.058635	-2.638184
SPC25	SPC25, NDC80 kinetochore complex component	3.0481286	-3.26463	-4.2064667
SPCS3	signal peptidase complex subunit 3 homolog (S. cerevisiae)	1.111231	-1.5407048	-1.3812397
SPDL1	spindle apparatus coiled-coil protein 1	-1.038788	-3.028336	-2.5925221
SPECC1	sperm antigen with calponin homology and coiled-coil domains 1	1.7657126	-1.2822666	-2.0049732
SPEG	SPEG complex locus	1.17459	-1.3636352	-1.798142
SPG11	spastic paraplegia 11 (autosomal recessive)	-2.1157386	-1.5614396	-1.2798105
SPG7	spastic paraplegia 7 (pure and complicated autosomal recessive)	-1.8827215	-1.9182078	-1.3867742
SPHAR	S-phase response (cyclin related)	1.8286676	2.4709136	3.0472612
SPHK1	sphingosine kinase 1	1.5954267	1.6639818	1.9198645
SPHK2	sphingosine kinase 2	1.363873	1.5645866	1.3639575
SPIN4	spindlin family, member 4	2.189632	-1.0586516	-1.9947528
SPINK13	serine peptidase inhibitor, Kazal type 13 (putative)	-1.0118114	4.342994	-2.5463407
SPINK7	serine peptidase inhibitor, Kazal type 7 (putative)	1.6886501	1.6448749	1.8355583
SPINT3	serine peptidase inhibitor, Kunitz type, 3	2.006382	24.25346	2.5855305
SPIRE2	spire-type actin nucleation factor 2	-1.1103905	-1.1517566	-1.8099381
SPNS1	spinster homolog 1 (Drosophila)	-1.5324687	-1.9649009	-1.7066847
SPNS2	spinster homolog 2 (Drosophila)	1.5895267	3.190424	5.834421
SPOCD1	SPOC domain containing 1	1.1125206	-16.554312	-1004.7295
SPOCK2	sparc/osteonectin, cwcv and kazal-like domains proteoglycan (testican) 2	2.5504377	2.5372148	2.8722453
SPP1	secreted phosphoprotein 1	-20.378233	-6.3879848	-22.398846
SPP2	secreted phosphoprotein 2, 24kDa	-4.593771	27.285614	58.4454
SPRNP1	shadow of prion protein homolog (zebrafish) pseudogene 1	2.0593264	1.9352475	1.8369548
SPRR1A	small proline-rich protein 1A	1.942729	1.9854966	1.9536252
SPRR2D	small proline-rich protein 2D	1.6555679	1.716242	1.9349966
SPRY2	sprouty homolog 2 (Drosophila)	-3.467053	-2.5513446	-2.8163273
SPRYD4	SPRY domain containing 4	1.0111649	1.4822377	1.7838074
SPRYD7	SPRY domain containing 7	2.5561993	3.1512074	2.5822172
SPSB1	splA/ryanodine receptor domain and SOCS box containing 1	-1.0144353	1.6041619	1.9098102
SPSB1	splA/ryanodine receptor domain and SOCS box containing 1	-1.647773	1.003688	1.2201207
SPSB2	splA/ryanodine receptor domain and SOCS box containing 2	1.5965359	2.0269961	2.003322
SPSB3	splA/ryanodine receptor domain and SOCS box containing 3	-1.1227825	1.36715	1.7072263
SPSB4	splA/ryanodine receptor domain and SOCS box containing 4	1.7757951	1.6681544	1.7652024
SPTBN1	spectrin, beta, non-erythrocytic 1	-1.4496118	-2.4179175	-4.979015
SPTLC3	serine palmitoyltransferase, long chain base subunit 3	-1.1219158	1.030837	2.3832026
SPTSSA	serine palmitoyltransferase, small subunit A	1.1250803	1.343669	2.6223829
SPTSSA	serine palmitoyltransferase, small subunit A	1.0289475	2.094321	3.979243
SQLE	squalene epoxidase	2.1201854	1.7013675	2.0997472
SQRDL	sulfide quinone reductase-like (yeast)	-1.2970825	1.5150857	1.1195068
SREBF1	sterol regulatory element binding transcription factor 1	2.324827	3.002283	4.2048006
SREBF1	sterol regulatory element binding transcription factor 1	1.8701361	1.8715407	2.1230595
SREK1	splicing regulatory glutamine/lysine-rich protein 1	-1.5784943	-1.7061552	-1.7849573
SRGAP1	SLIT-ROBO Rho GTPase activating protein 1	-1.480463	-1.3918879	-2.368534
SRGAP2	SLIT-ROBO Rho GTPase activating protein 2	-1.5148708	-1.9965576	-1.2121831
SRGAP2C	SLIT-ROBO Rho GTPase activating protein 2C	-1.2316146	-1.562779	-1.151537
SRGN	serglycin	1.9996177	1.18228	-2.1012805
SRM	spermidine synthase	1.1705662	-1.0465056	-1.7425169
SRP72	signal recognition particle 72kDa	1.5492269	1.5010701	1.1602569
SRPRB	signal recognition particle receptor, B subunit	1.0182475	1.3755159	1.5167617
SRPX2	sushi-repeat containing protein, X-linked 2	-2.6737344	-6.87137	-25.19331
SRRM3	serine/arginine repetitive matrix 3	1.2204133	4.6077957	6.7726965
SRRM3	serine/arginine repetitive matrix 3	1.1079338	1.1712215	-8.3530445
SRRT	serrate, RNA effector molecule	-1.0435176	-1.7078086	-1.6527433
SRRT	serrate, RNA effector molecule	-1.8335623	-2.9572468	-3.0882413
SRSF10	serine/arginine-rich splicing factor 10	-1.1428986	-1.8629682	-1.9312867
SRSF11	serine/arginine-rich splicing factor 11	-1.2619545	-1.5601952	-1.4741279
SRSF2	serine/arginine-rich splicing factor 2	-1.0299962	-1.9227448	-2.2655559
SRSF3	serine/arginine-rich splicing factor 3	1.9661336	-1.0820541	-1.0316765
SRSF5	serine/arginine-rich splicing factor 5	-1.1816095	-1.6211687	1.2826374
SRSF5	serine/arginine-rich splicing factor 5	-1.1085434	-1.5652981	1.3757917
SRSF7	serine/arginine-rich splicing factor 7	2.257375	1.0103167	1.1037792
SRSF7	serine/arginine-rich splicing factor 7	1.8491849	1.1631792	1.4463726
SSB	Sjogren syndrome antigen B (autoantigen La)	1.5261127	1.1013716	1.3077947
SSB	Sjogren syndrome antigen B (autoantigen La)	1.5696571	1.0815597	1.2664919
SSBP1	single-stranded DNA binding protein 1, mitochondrial	1.6503032	1.3250656	1.2136061
SSBP4	single stranded DNA binding protein 4	1.1261115	-1.2335889	-1.7940437
SSC4D	scavenger receptor cysteine rich family, 4 domains	1.1450413	1.3731871	1.5036435
SSH1	slingshot protein phosphatase 1	-1.7801377	-3.2371893	-3.0889983
SSR1	signal sequence receptor, alpha	-1.1520461	1.2421026	1.8433915
SSR1	signal sequence receptor, alpha	-1.1551639	-1.5371554	-1.4191508
SSTR3	somatostatin receptor 3	2.385569	2.5836391	3.0814447
SSX2	synovial sarcoma, X breakpoint 2	1.6364591	1.748419	3.2053099
ST13	suppression of tumorigenicity 13 (colon carcinoma) (Hsp70 interacting protein)	1.283831	1.6908919	1.705391
ST14	suppression of tumorigenicity 14 (colon carcinoma)	1.5153235	1.8548943	1.9836534
ST3GAL1	ST3 beta-galactoside alpha-2,3-sialyltransferase 1	-1.1181448	1.7676034	1.6061565
ST3GAL2	ST3 beta-galactoside alpha-2,3-sialyltransferase 2	-1.642553	-1.7425333	-1.4257985
ST3GAL3	ST3 beta-galactoside alpha-2,3-sialyltransferase 3	1.400011	1.3831003	2.62141
ST3GAL6	ST3 beta-galactoside alpha-2,3-sialyltransferase 6	1.1466918	2.5730839	3.6508713
ST5	suppression of tumorigenicity 5	-1.5840553	-1.2329996	-1.5993301
ST6GAL1	ST6 beta-galactosamide alpha-2,6-sialyltranferase 1	-6.2614427	5.180803	8.553307
ST6GALNAC6	ST6 (alpha-N-acetyl-neuraminyl-2,3-beta-galactosyl-1,3)-N-acetylgalactosaminide alpha-2,6-s	-1.2437434	1.59013	1.4186994
STAMBP	STAM binding protein	-2.1920218	-2.570282	-2.0718277
STAP2	signal transducing adaptor family member 2	-1.942918	1.2595867	1.0392928
STARD10	StAR-related lipid transfer (START) domain containing 10	-3.2331858	1.2535805	3.484791
STARD3	StAR-related lipid transfer (START) domain containing 3	-1.6272187	-1.9834623	-1.4905329
STARD3NL	STARD3 N-terminal like	-1.0134928	-1.8383341	-1.8578993
STARD5	StAR-related lipid transfer (START) domain containing 5	2.5843592	2.482603	2.9300504
STARD8	StAR-related lipid transfer (START) domain containing 8	-1.7358139	1.8089311	1.4182249
STAT1	signal transducer and activator of transcription 1, 91kDa	-1.4171572	-1.875362	-1.7153803
STAT2	signal transducer and activator of transcription 2, 113kDa	1.5112374	1.1743695	1.1758454
STAT5B	signal transducer and activator of transcription 5B	1.4874576	1.3983985	1.6903147
STAT6	signal transducer and activator of transcription 6, interleukin-4 induced	1.0778958	1.021316	-1.7717445
STAU2	staufen double-stranded RNA binding protein 2	1.6620338	1.8883431	2.6519358
STBD1	starch binding domain 1	-1.7244278	1.94383	2.4696171
STK11IP	serine/threonine kinase 11 interacting protein	-1.0399064	-1.1027001	-1.9091086
STK16	serine/threonine kinase 16	1.4851376	2.7528162	2.5670946
STK26	serine/threonine protein kinase 26	1.5979449	1.2295483	1.2054483
STK3	serine/threonine kinase 3	1.400026	1.3844198	1.5307549
STK39	serine threonine kinase 39	2.5073342	3.2362134	-1.9259167
STK4	serine/threonine kinase 4	1.51065	2.7501516	3.1165268
STK4	serine/threonine kinase 4	-1.9681151	-2.6010997	-2.004573
STMN1	stathmin 1	1.890737	-2.6210797	-2.3660967

STRADA	STE20-related kinase adaptor alpha	1.0731244	1.0018995	1.5448377
STRAP	serine/threonine kinase receptor associated protein	1.5832808	1.2935299	1.4122312
STRC	stereocilin	1.8906325	1.8671436	1.6899285
STRIP1	striatin interacting protein 1	-1.4568931	-1.6843597	-1.712978
STT3A	STT3A, subunit of the oligosaccharyltransferase complex (catalytic)	-1.3048984	-1.8820912	-2.7712438
STT3B	STT3B, subunit of the oligosaccharyltransferase complex (catalytic)	-1.2549639	-1.8499234	-1.5196068
STX10	syntaxin 10	-1.5982271	-1.7928278	-1.9239105
STX1A	syntaxin 1A (brain)	-2.6443837	-3.3758883	-2.586413
STX2	syntaxin 2	-1.1430994	-1.9310428	-1.8669915
STXBP6	syntaxin binding protein 6 (amisyn)	1.977121	1.0282365	-1.02972
STYX	serine/threonine/tyrosine interacting protein	1.4931118	1.538196	1.9646984
SUB1	SUB1 homolog (S. cerevisiae)	2.3315413	1.8318934	2.045633
SUCLG1	succinate-CoA ligase, alpha subunit	1.6955075	2.39181	1.9637502
SUCLG2	succinate-CoA ligase, GDP-forming, beta subunit	1.075885	1.4437623	2.043151
SUCO	SUN domain containing ossification factor	-1.4963312	-1.9642141	-1.2195853
SUGP2	SURP and G patch domain containing 2	-1.4383347	-2.2627928	-2.1250832
SUGP2	SURP and G patch domain containing 2	-1.7303178	-2.3699117	-2.1433654
SUGT1	SGT1, suppressor of G2 allele of SKP1 (S. cerevisiae)	-1.4786199	-1.5866174	-1.5484082
SULF1	sulfatase 1	-2.864659	-1.4700261	-2.442881
SULT1A2	sulfotransferase family, cytosolic, 1A, phenol-preferring, member 2	1.2681526	2.3654237	2.8085282
SULT1A4	sulfotransferase family, cytosolic, 1A, phenol-preferring, member 4	1.0177813	1.760682	1.8297224
SULT1C2	sulfotransferase family, cytosolic, 1C, member 2	-6.3750744	1.3281702	-2.0257223
SULT1C2	sulfotransferase family, cytosolic, 1C, member 2	-5.6900334	1.4306496	1.0267562
SULT2A1	sulfotransferase family, cytosolic, 2A, dehydroepiandrosterone (DHEA)-preferring, member 1	-3.8089952	14.894657	71.86473
SUMO1	small ubiquitin-like modifier 1	1.4663832	1.2300682	1.6244743
SUN2	Sad1 and UNC84 domain containing 2	-1.4387869	1.0108155	1.56506
SUOX	sulfite oxidase	-1.2127761	1.6532371	2.4819636
SUPT4H1	suppressor of Ty 4 homolog 1 (S. cerevisiae)	1.705671	1.8583897	2.322184
SUPT5H	suppressor of Ty 5 homolog (S. cerevisiae)	-1.5089526	-1.5973849	-1.6778232
SUPT6H	suppressor of Ty 6 homolog (S. cerevisiae)	-1.9881774	-1.8301605	-1.755272
SURF1	surfeit 1	1.286019	2.0560215	2.9623673
SURF4	surfeit 4	1.3802071	1.360347	1.8532791
SUSD2	sushi domain containing 2	3.9858592	6.691944	1.7376752
SUSD3	sushi domain containing 3	2.4271486	5.9545984	7.399642
SUSD4	sushi domain containing 4	2.5729895	2.8465197	3.1662486
SUV39H2	suppressor of variegation 3-9 homolog 2 (Drosophila)	1.4541098	-1.8553524	-1.4406016
SWAP70	SWAP switching B-cell complex 70kDa subunit	1.299924	-1.1985642	-1.7208929
SWI5	SWI5 recombination repair homolog (yeast)	-1.0060159	1.1097131	1.7519114
SYBU	syntabulin (syntaxin-interacting)	1.2587949	3.4610367	3.2372684
SYCE3	synaptonemal complex central element protein 3	-1.1923728	2.0907366	2.0093493
SYCE3	synaptonemal complex central element protein 3	-1.0719717	2.3159032	1.975362
SYK	spleen tyrosine kinase	-1.1691829	-1.6927692	-2.681666
SYNC	syncoilin, intermediate filament protein	1.3037512	-1.9168406	-7.691791
SYNCRIP	synaptotagmin binding, cytoplasmic RNA interacting protein	1.3286914	-1.1929227	-1.5025804
SYNCRIP	synaptotagmin binding, cytoplasmic RNA interacting protein	-1.0771426	-1.3968469	-1.7606964
SYNE1	spectrin repeat containing, nuclear envelope 1	-2.7953045	-1.3304312	-1.4801763
SYNE1	spectrin repeat containing, nuclear envelope 1	2.9592874	3.583591	3.270287
SYNGR1	synaptogyrin 1	1.5433127	1.4401647	-2.3198824
SYNGR1	synaptogyrin 1	1.5092368	1.3273932	-2.448257
SYNGR2	synaptogyrin 2	-1.0331993	1.345247	1.6894516
SYNGR4	synaptogyrin 4	2.643746	3.194789	3.2237186
SYNPO	synaptopodin	1.0560495	1.8597676	-2.5864513
SYNPO	synaptopodin	-1.0517522	1.0865989	-1.9252353
SYPL2	synaptophysin-like 2	1.1896402	1.5518813	1.6329671
SYT12	synaptotagmin XII	-1.6509771	1.3421211	-3.671461
SYT13	synaptotagmin XIII	-1.9446095	-1.5416192	-11.412266
SYT7	synaptotagmin VII	-1.7792071	3.2283337	3.7013643
SYTL1	synaptotagmin-like 1	-1.2194417	-1.8105282	-5.332188
SYTL2	synaptotagmin-like 2	-1.1701313	-2.02092	-8.751412
SYVN1	synovial apoptosis inhibitor 1, synoviolin	-1.6263726	1.1437958	1.5366815
TAAR5	trace amine associated receptor 5	2.1049066	2.194616	2.4481826
TACC3	transforming, acidic coiled-coil containing protein 3	-1.0224518	-6.508196	-7.8135343
TACO1	translational activator of mitochondrially encoded cytochrome c oxidase I	1.2644676	2.1019835	2.5509818
TAF10	TAF10 RNA polymerase II, TATA box binding protein (TBP)-associated factor, 30kDa	1.5620407	1.565375	1.9003882
TAF12	TAF12 RNA polymerase II, TATA box binding protein (TBP)-associated factor, 20kDa	1.330169	1.6050593	1.6114156
TAF1B	TATA box binding protein (TBP)-associated factor, RNA polymerase I, B, 63kDa	-1.2912341	-2.044501	-1.8553804
TAF1C	TATA box binding protein (TBP)-associated factor, RNA polymerase I, C, 110kDa	-1.375455	-1.3809338	-1.5229517
TAGLN	transgelin	3.0887997	-1.5316217	-1.4213132
TAGLN2	transgelin 2	1.1523675	-1.6056871	-2.8327808
TAMM41	TAM41, mitochondrial translocator assembly and maintenance protein, homolog (S. cerevisia	1.5015438	1.1468943	1.34518
TANC2	tetratricopeptide repeat, ankyrin repeat and coiled-coil containing 2	-1.2160119	-1.7524011	-3.8076596
TANGO2	transport and golgi organization 2 homolog (Drosophila)	-1.462496	-1.3811263	-2.060506
TAOK1	TAO kinase 1	-1.7465806	-1.6513689	-1.7735974
TAOK3	TAO kinase 3	1.0204197	1.480108	1.6150413
TAP1	transporter 1, ATP-binding cassette, sub-family B (MDR/TAP)	-1.1147197	-2.01935	-2.7573135
TAP2	transporter 2, ATP-binding cassette, sub-family B (MDR/TAP)	1.0474875	-1.6268176	-2.2586536
TAPBP	TAP binding protein (tapasin)	-1.7908013	-1.7885695	-2.3479419
TAPBP	TAP binding protein (tapasin)	-1.3819709	-1.3412261	-1.7492526
TAPT1	transmembrane anterior posterior transformation 1	-1.0013114	1.2278532	2.354944
TAPT1-AS1	TAPT1 antisense RNA 1 (head to head)	2.5585644	1.996123	2.0969617
TARP	TCR gamma alternate reading frame protein	2.3965333	2.0047178	1.8898406
TARS	threonyl-tRNA synthetase	-1.0933973	-1.6543747	-1.4197326
TAS1R3	taste receptor, type 1, member 3	2.9173923	3.7728317	4.095854
TAT-AS1	TAT antisense RNA 1	-2.2257369	1.3557439	1.9200476
TATDN3	TatD DNase domain containing 3	2.1080937	1.9188722	2.2727964
TAX1BP3	Tax1 (human T-cell leukemia virus type I) binding protein 3	-1.1875472	-1.5424379	-2.731571
TBC1D1	TBC1 (tre-2/USP6, BUB2, cdc16) domain family, member 1	-1.2794998	-1.5376961	-1.535678
TBC1D10B	TBC1 domain family, member 10B	1.7906916	1.6238729	1.7394372
TBC1D15	TBC1 domain family, member 15	-1.5653245	-1.638926	-2.0316308
TBC1D16	TBC1 domain family, member 16	1.141793	1.2340009	2.0993905
TBC1D17	TBC1 domain family, member 17	-1.7287309	-1.185378	-1.037319
TBC1D19	TBC1 domain family, member 19	-1.2831686	-1.5424885	-1.3661429
TBC1D2	TBC1 domain family, member 2	2.6462595	1.4506838	-1.6869977
TBC1D3B	TBC1 domain family, member 3B	-2.930449	-3.604197	-3.1740289
TBC1D3L	TBC1 domain family, member 3L	1.5259483	1.9263092	2.1204917
TBC1D8	TBC1 domain family, member 8 (with GRAM domain)	1.1655017	1.6313177	1.8822027
TBC1D9B	TBC1 domain family, member 9B (with GRAM domain)	1.1047865	-1.0847625	1.5451933
TBCA	tubulin folding cofactor A	1.6650475	1.785094	1.5928022
TBCD	tubulin folding cofactor D	-1.6389956	-2.1760128	-1.5288321
TBCD	tubulin folding cofactor D	-1.2913994	-1.6621938	-1.15296
TBCE	tubulin folding cofactor E	1.5410113	1.1417153	1.6850233
TBCEL	tubulin folding cofactor E-like	1.5849727	1.9744307	2.6185474
TBKBP1	TBK1 binding protein 1	2.3643353	2.2682464	2.34277
TBL1XR1	transducin (beta)-like 1 X-linked receptor 1	1.0805273	-1.5189704	-2.1310263
TBL1XR1	transducin (beta)-like 1 X-linked receptor 1	1.1150041	-1.4899598	-1.9131664
TBL2	transducin (beta)-like 2	1.5365489	1.4405522	1.8252802
TBL3	transducin (beta)-like 3	1.6288673	1.5769247	1.8424069
TBRG1	transforming growth factor beta regulator 1	-1.565388	-1.731641	-1.167304
TBXA2R	thromboxane A2 receptor	2.3694546	2.5533495	2.5918791

TCAF1	TRPM8 channel-associated factor 1	-1.2198575	1.3638372	1.6637216
TCAIM	T cell activation inhibitor, mitochondrial	1.1600132	1.4353244	1.8324195
TCEA1	transcription elongation factor A (SII), 1	-1.8712993	-2.1037526	-1.8560936
TCEAL2	transcription elongation factor A (SII)-like 2	4.333191	2.9812713	2.5768049
TCEAL4	transcription elongation factor A (SII)-like 4	2.5011644	2.054179	1.6748497
TCEAL5	transcription elongation factor A (SII)-like 5	2.1330335	1.0187439	-2.7531784
TCEAL6	transcription elongation factor A (SII)-like 6	1.8209939	-1.0996492	-2.8900583
TCEAL6	transcription elongation factor A (SII)-like 6	1.8427103	-1.0833857	-3.1051524
TCEB1	transcription elongation factor B (SIII), polypeptide 1 (15kDa, elongin C)	1.5791067	1.222483	1.0844692
TCF3	transcription factor 3	1.0486133	-1.4736408	-2.5090873
TCF4	transcription factor 4	-1.3720851	-1.9250602	-2.1586926
TCIRG1	T-cell, immune regulator 1, ATPase, H+ transporting, lysosomal V0 subunit A3	-1.5440426	-1.7063305	-2.5214162
TCN2	transcobalamin II	-1.9411846	1.2001244	-6.7534113
TCOF1	Treacher Collins-Franceschetti syndrome 1	-1.2396181	-1.7371356	-3.07137
TCP11L1	t-complex 11, testis-specific-like 1	1.1266605	-1.7050128	-1.4647647
TCTA	T-cell leukemia translocation altered	1.067176	1.2117137	-1.8653857
TCTEX1D4	Tctex1 domain containing 4	1.276443	1.2491515	2.4662962
TDG	thymine-DNA glycosylase	1.2105427	-1.6853359	-1.4336674
TDO2	tryptophan 2,3-dioxygenase	-20.255114	2.499957	2.9508243
TDP1	tyrosyl-DNA phosphodiesterase 1	1.26489	-2.1166523	-2.1906505
TDRG1	testis development related 1 (non-protein coding)	-2.1091568	2.5884547	4.00731
TECPR1	tectonin beta-propeller repeat containing 1	-1.5462251	-1.6209317	-1.0372435
TEF	thyrotrophic embryonic factor	-1.8720527	1.5748028	3.661378
TENM2	teneurin transmembrane protein 2	1.3916177	-3.7677617	-9.478984
TENM2	teneurin transmembrane protein 2	1.7706836	-2.9332566	-5.9300017
TEP1	telomerase-associated protein 1	-1.6257143	-1.5402673	-1.4216847
TERF1	telomeric repeat binding factor (NIMA-interacting) 1	1.3621945	1.127689	1.5198143
TERF1	telomeric repeat binding factor (NIMA-interacting) 1	2.0940292	1.8366827	2.3369963
TERF1	telomeric repeat binding factor (NIMA-interacting) 1	2.397505	1.9327683	2.357594
TES	testis derived transcript (3 LIM domains)	-1.05138	-1.7743645	-4.3728557
TESC	tescalcin	-3.0358667	-1.2838995	-3.3836038
TEX10	testis expressed 10	1.1235797	-1.217285	-1.9060278
TEX2	testis expressed 2	1.2521876	1.3302484	2.2937279
TEX264	testis expressed 264	1.1624662	1.5449388	1.5702224
TEX30	testis expressed 30	-1.5228186	-2.5066543	-1.6097971
TF	transferrin	-2.3612158	14.018463	36.56142
TFDP1	transcription factor Dp-1	1.9562992	-1.252821	-1.2132611
TFDP2	transcription factor Dp-2 (E2F dimerization partner 2)	1.5289384	1.3418883	1.6041408
TFE3	transcription factor binding to IGHM enhancer 3	2.196413	1.9630679	2.2263796
TFF3	trefoil factor 3 (intestinal)	-1.4958143	1.6247538	-1.0537617
TFPI2	tissue factor pathway inhibitor 2	-5.8892922	-11.08909	-9.656313
TG	thyroglobulin	3.1938858	6.688488	1.9232856
TGFB1	transforming growth factor, beta 1	-1.8980694	-2.264972	-4.091801
TGFB1I1	transforming growth factor beta 1 induced transcript 1	1.6341335	1.3931694	1.4146159
TGFB2	transforming growth factor, beta 2	-1.1736108	-4.475854	-26.531841
TGFBI	transforming growth factor, beta-induced, 68kDa	-1.3573024	-2.0394475	-4.4652185
TGFBR2	transforming growth factor, beta receptor II (70/80kDa)	1.1919274	1.7202139	2.27409
TGFBRAP1	transforming growth factor, beta receptor associated protein 1	1.0481079	-1.3884667	-1.9293972
TGIF1	TGFB-induced factor homeobox 1	-1.1074691	-2.086661	-2.6840563
TGM2	transglutaminase 2	-1.2155795	-2.1210048	-5.9692373
TGS1	trimethylguanosine synthase 1	1.7732531	1.3032697	1.1399078
THAP3	THAP domain containing, apoptosis associated protein 3	1.2277598	1.4776868	1.6413982
THAP6	THAP domain containing 6	1.021101	1.2426757	1.5159875
THBS1	thrombospondin 1	-1.3318387	-3.025964	-2.7347949
THBS3	thrombospondin 3	-1.5134302	-1.2337664	-1.6018466
THEM6	thioesterase superfamily member 6	2.523521	3.5951998	3.4016852
THNSL2	threonine synthase-like 2 (S. cerevisiae)	-1.2246006	1.4720919	2.019656
THOC2	THO complex 2	-1.206726	-1.1495279	-1.5658332
THRA	thyroid hormone receptor, alpha	2.658865	3.043652	3.3134468
THSD1	thrombospondin, type I, domain containing 1	-1.1595623	-3.443338	-6.104186
THTPA	thiamine triphosphatase	1.3474087	1.7500768	1.3952657
TIA1	TIA1 cytotoxic granule-associated RNA binding protein	-1.4601588	-2.067703	-2.1378322
TIGD2	tigger transposable element derived 2	2.875189	2.127431	2.3989623
TIGD5	tigger transposable element derived 5	2.6148648	2.798267	2.2043288
TIMELESS	timeless circadian clock	1.0779704	-2.6878407	-1.8108581
TIMM10	translocase of inner mitochondrial membrane 10 homolog (yeast)	1.5375017	1.5052788	1.4262116
TIMM10B	translocase of inner mitochondrial membrane 10 homolog B (yeast)	1.5614706	1.972842	1.7290602
TIMM17A	translocase of inner mitochondrial membrane 17 homolog A (yeast)	1.6649698	1.2015102	1.1473289
TIMM8A	translocase of inner mitochondrial membrane 8 homolog A (yeast)	1.6513838	1.6863447	1.3872441
TIMM8B	translocase of inner mitochondrial membrane 8 homolog B (yeast)	1.7417028	1.6664343	2.077398
TIPARP	TCDD-inducible poly(ADP-ribose) polymerase	-3.1308556	-2.0737097	-1.6815354
TIPIN	TIMELESS interacting protein	1.478744	-1.6590103	-1.8460293
TJAP1	tight junction associated protein 1 (peripheral)	-1.274603	-1.5530258	-1.671914
TJP1	tight junction protein 1	-1.2027699	-1.4024166	-1.7494525
TK1	thymidine kinase 1, soluble	3.0097306	-2.644096	-3.1125164
TK2	thymidine kinase 2, mitochondrial	1.1219751	1.764835	2.7438993
TKT	transketolase	1.2947712	-1.5180513	-2.1445913
TLCD1	TLC domain containing 1	1.3659933	2.1785688	1.7614608
TLDC1	TBC/LysM-associated domain containing 1	-1.1928085	-1.6182976	-2.6026862
TLE1	transducin-like enhancer of split 1 (E(sp1) homolog, Drosophila)	-1.0899453	1.3761504	1.8412241
TLE1	transducin-like enhancer of split 1 (E(sp1) homolog, Drosophila)	1.8234122	1.6897625	1.6720337
TLE1	transducin-like enhancer of split 1 (E(sp1) homolog, Drosophila)	-1.1396118	1.4191114	1.9279516
TLE2	transducin-like enhancer of split 2	-1.1479008	1.3765517	-3.7993293
TLN1	talin 1	-2.2306838	-2.2137268	-4.992391
TLN2	talin 2	3.077702	2.5385096	1.9448613
TLR2	toll-like receptor 2	-2.0660095	-2.0379617	-2.122687
TLR3	toll-like receptor 3	-1.5101697	-1.9810708	-2.2948031
TM2D2	TM2 domain containing 2	-1.1762328	-1.6938051	1.1871629
TM4SF1	transmembrane 4 L six family member 1	-1.047407	-1.4799893	-4.394585
TM4SF1	transmembrane 4 L six family member 1	-1.3444914	-2.203387	-6.721186
TM4SF4	transmembrane 4 L six family member 4	-3.1231487	1.9618998	1.098228
TM4SF5	transmembrane 4 L six family member 5	-1.8724648	3.1089215	3.4709537
TM7SF2	transmembrane 7 superfamily member 2	1.0806673	3.7673035	10.450668
TM7SF3	transmembrane 7 superfamily member 3	-1.0141773	-1.7709491	-2.1969693
TM7SF3	transmembrane 7 superfamily member 3	1.1784198	-1.4926856	-1.8555712
TMA7	translation machinery associated 7 homolog (S. cerevisiae)	1.9605532	1.7273334	1.7020179
TMBIM4	transmembrane BAX inhibitor motif containing 4	-1.1569346	1.5183138	1.9205325
TMBIM6	transmembrane BAX inhibitor motif containing 6	-1.4768589	1.1335995	2.0700803
TMCO4	transmembrane and coiled-coil domains 4	1.0426949	1.5441761	-1.1528194
TMED5	transmembrane emp24 protein transport domain containing 5	-1.8648975	-1.8752341	-1.9263356
TMEM106B	transmembrane protein 106B	1.0022272	1.3687644	1.8533125
TMEM107	transmembrane protein 107	1.6207541	-1.4134288	-1.3891022
TMEM109	transmembrane protein 109	1.5053858	1.1682901	1.3964859
TMEM11	transmembrane protein 11	1.5232745	1.3769515	1.4131042
TMEM110	transmembrane protein 110	1.0984405	1.0633795	2.5555737
TMEM120A	transmembrane protein 120A	1.0235637	2.0677838	3.1323586
TMEM123	transmembrane protein 123	1.1206981	-1.033512	-1.566145
TMEM129	transmembrane protein 129, E3 ubiquitin protein ligase	1.7058785	2.3922153	2.4912262
TMEM132A	transmembrane protein 132A	-1.1309694	-2.2668796	-4.379152

TMEM135	transmembrane protein 135	1.2832193	1.4322788	2.2174442
TMEM136	transmembrane protein 136	1.5910801	1.0467218	-1.6097499
TMEM136	transmembrane protein 136	2.0141294	1.3978572	1.0227627
TMEM140	transmembrane protein 140	-2.0006738	1.1173649	1.796273
TMEM141	transmembrane protein 141	1.1642613	1.6801796	2.0093021
TMEM143	transmembrane protein 143	1.2095077	1.7041489	1.7393662
TMEM145	transmembrane protein 145	-1.1565067	1.2260715	-1.7748803
TMEM145	transmembrane protein 145	1.2005582	1.8606694	1.3586837
TMEM147	transmembrane protein 147	1.1855991	1.0191337	-1.5392992
TMEM14A	transmembrane protein 14A	1.4352086	1.6757348	3.4401069
TMEM14C	transmembrane protein 14C	1.6445385	1.2776144	1.1359329
TMEM158	transmembrane protein 158 (gene/pseudogene)	-2.340507	-3.6813543	-1.9805537
TMEM159	transmembrane protein 159	1.2576292	1.0667347	-1.5304046
TMEM161A	transmembrane protein 161A	1.0544244	1.1214206	1.5376159
TMEM165	transmembrane protein 165	1.2651495	-1.3419688	-1.8531892
TMEM170B	transmembrane protein 170B	-1.1060406	1.6235651	2.432069
TMEM173	transmembrane protein 173	2.5078707	1.6371199	-1.3705677
TMEM175	transmembrane protein 175	-1.1937809	1.0206697	1.5908381
TMEM176A	transmembrane protein 176A	-2.9704382	1.7833825	1.1989825
TMEM176B	transmembrane protein 176B	-3.727904	1.1353805	-1.1965723
TMEM177	transmembrane protein 177	1.3818159	1.7430358	2.0169358
TMEM178A	transmembrane protein 178A	4.448873	3.7548873	3.6080763
TMEM180	transmembrane protein 180	1.0010077	-1.031201	1.7187587
TMEM184C	transmembrane protein 184C	1.413348	1.7855682	2.044198
TMEM185B	transmembrane protein 185B	-1.0156353	-1.440889	-1.9825962
TMEM185B	transmembrane protein 185B	-1.049081	-1.4145924	-2.8471317
TMEM186	transmembrane protein 186	1.2540625	1.7719692	1.574505
TMEM187	transmembrane protein 187	1.451191	1.8595724	2.1581786
TMEM191A	transmembrane protein 191A (pseudogene)	1.2568326	1.831263	1.8444847
TMEM191B	transmembrane protein 191B	1.1077526	1.5394768	1.5931087
TMEM201	transmembrane protein 201	1.7453946	1.1628616	-1.767987
TMEM203	transmembrane protein 203	1.1869558	1.5239904	1.7630721
TMEM205	transmembrane protein 205	-1.5859451	1.683656	2.2025368
TMEM208	transmembrane protein 208	1.074613	1.0964428	1.6557958
TMEM208	transmembrane protein 208	1.0272512	1.07699	1.6697025
TMEM234	transmembrane protein 234	1.0543355	-1.5334599	-2.080642
TMEM237	transmembrane protein 237	1.3492326	-1.7756046	-1.7118475
TMEM238	transmembrane protein 238	-1.1261982	1.9821578	2.5667756
TMEM242	transmembrane protein 242	1.1919187	1.6797503	3.0422401
TMEM245	transmembrane protein 245	-1.1520454	1.1408615	1.6865102
TMEM246	transmembrane protein 246	1.8383079	1.6379724	1.3971921
TMEM25	transmembrane protein 25	2.0107272	1.176472	2.1162004
TMEM254	transmembrane protein 254	-1.3514857	1.2942127	1.7029672
TMEM256	transmembrane protein 256	1.3871837	1.5671755	2.329544
TMEM27	transmembrane protein 27	-1.6285174	1.4602426	1.3857986
TMEM30B	transmembrane protein 30B	1.1702126	4.265281	9.357155
TMEM37	transmembrane protein 37	1.0359462	2.5282006	3.3135476
TMEM38A	transmembrane protein 38A	1.4271126	2.667002	2.098248
TMEM41A	transmembrane protein 41A	-1.3744893	1.1147643	2.0520844
TMEM42	transmembrane protein 42	1.3198798	1.4576979	1.9988952
TMEM44	transmembrane protein 44	-2.8561282	-5.7615323	-8.549197
TMEM45A	transmembrane protein 45A	1.6382452	2.204066	1.2963936
TMEM5	transmembrane protein 5	-1.1192155	-1.4014252	-1.5795571
TMEM50B	transmembrane protein 50B	1.3111293	1.705816	1.72339
TMEM51	transmembrane protein 51	-2.315915	-3.428572	-4.526716
TMEM53	transmembrane protein 53	-1.6484083	1.1943387	1.180782
TMEM54	transmembrane protein 54	-1.0649086	-1.6584711	-1.8086013
TMEM55B	transmembrane protein 55B	1.0855104	-1.0205253	1.7496853
TMEM56	transmembrane protein 56	1.197624	1.1296765	3.1862216
TMEM57	transmembrane protein 57	-1.6099745	-1.2084727	1.1448516
TMEM59	transmembrane protein 59	1.0368273	1.3173515	1.7848772
TMEM80	transmembrane protein 80	-1.2350583	1.226422	2.1749256
TMEM82	transmembrane protein 82	3.8269706	3.3788598	3.3692899
TMEM86B	transmembrane protein 86B	-1.3256142	1.9956683	1.8122034
TMEM87A	transmembrane protein 87A	-1.4597113	-1.5313876	-1.603582
TMEM8B	transmembrane protein 8B	-1.3318402	-1.4384041	1.7598038
TMEM8C	transmembrane protein 8C	1.453567	1.4812768	1.8845657
TMEM8C	transmembrane protein 8C	2.6611948	2.6986804	2.5894349
TMEM98	transmembrane protein 98	2.1370552	1.186538	1.3231525
TMIGD2	transmembrane and immunoglobulin domain containing 2	2.7984421	2.4332578	2.590163
TMOD3	tropomodulin 3 (ubiquitous)	-1.4845455	-2.285907	-1.7244924
TMPRSS2	transmembrane protease, serine 2	-1.323165	2.2855906	4.2187133
TMPRSS6	transmembrane protease, serine 6	1.4585954	4.6096025	13.278449
TMSB10	thymosin beta 10	1.1642293	-1.5310091	-2.3403351
TMSB15A	thymosin beta 15a	2.2665722	-3.4837594	-3.0422864
TMSB15B	thymosin beta 15B	1.1940527	-2.4672515	-3.119633
TMSB4X	thymosin beta 4, X-linked	-1.3287146	-2.1632833	-2.8611097
TMSB4X	thymosin beta 4, X-linked	-1.2324375	-2.102501	-2.7680154
TMTC1	transmembrane and tetratricopeptide repeat containing 1	2.1062772	-1.21737	1.6813143
TMTC1	transmembrane and tetratricopeptide repeat containing 1	1.4452224	-1.0733862	1.7314636
TMUB2	transmembrane and ubiquitin-like domain containing 2	-1.7437913	-1.979364	-2.0903015
TMX3	thioredoxin-related transmembrane protein 3	1.3303862	-1.2514312	-2.2332563
TNC	tenascin C	-7.5417924	-13.095004	-94.87672
TNFAIP2	tumor necrosis factor, alpha-induced protein 2	-2.2358258	-2.97704	-6.4786553
TNFAIP3	tumor necrosis factor, alpha-induced protein 3	-4.833983	-4.269881	-11.941919
TNFAIP8	tumor necrosis factor, alpha-induced protein 8	-2.5422335	-2.353779	-3.1508398
TNFRSF10A	tumor necrosis factor receptor superfamily, member 10a	-1.4787765	-2.1727958	-3.1446402
TNFRSF10B	tumor necrosis factor receptor superfamily, member 10b	-1.3681719	-2.3372395	-2.6330018
TNFRSF10D	tumor necrosis factor receptor superfamily, member 10d, decoy with truncated death domain	-1.5171701	-2.288208	-2.5469296
TNFRSF12A	tumor necrosis factor receptor superfamily, member 12A	-1.7257025	-2.812231	-2.6182199
TNFRSF14	tumor necrosis factor receptor superfamily, member 14	-1.0491128	1.182098	-1.665354
TNFRSF14	tumor necrosis factor receptor superfamily, member 14	1.5835705	1.4522533	1.6263287
TNFRSF21	tumor necrosis factor receptor superfamily, member 21	-2.1135511	-3.7573721	-3.146077
TNFRSF25	tumor necrosis factor receptor superfamily, member 25	-1.6716586	-1.9118252	-3.6395588
TNFSF10	tumor necrosis factor (ligand) superfamily, member 10	-4.673061	1.7855158	2.3475802
TNFSF10	tumor necrosis factor (ligand) superfamily, member 10	-6.952199	1.3277048	1.7060571
TNFSF9	tumor necrosis factor (ligand) superfamily, member 9	1.5670786	-2.0809615	-4.6262794
TNNT1	troponin T type 1 (skeletal, slow)	-1.4067078	-3.7811296	-13.11185
TNPO3	transportin 3	-1.4469787	-1.7319409	-1.5287241
TNRC18	trinucleotide repeat containing 18	1.7697973	2.2546036	2.8128328
TNRC18P1	TNRC18P1	1.6628606	1.8481262	1.763145
TNRC6A	trinucleotide repeat containing 6A	-1.5444399	-1.4150503	-1.205787
TNS3	tensin 3	-1.2604852	-1.3056215	-2.849274
TNS4	tensin 4	9.803647	1.0841337	-4.3885427
TNXB	tenascin XB	1.8827587	2.5648797	3.4481933
TOB2	transducer of ERBB2, 2	1.7094262	3.6392329	6.124981
TOLLIP	toll interacting protein	1.0366006	1.2654932	1.8629466
TOM1	target of myb1 (chicken)	-1.2888314	1.0321338	1.5242815
TOM1L1	target of myb1 (chicken)-like 1	1.083413	1.7556055	2.0771995

TOM1L2	target of myb1-like 2 (chicken)	1.0864019	1.3039786	1.8159177
TOMM20	translocase of outer mitochondrial membrane 20 homolog (yeast)	1.7545451	1.6409429	1.0399485
TOMM22	translocase of outer mitochondrial membrane 22 homolog (yeast)	1.6695602	1.342031	1.1060607
TOMM7	translocase of outer mitochondrial membrane 7 homolog (yeast)	1.2491125	1.3653439	1.5561571
TOMM7	translocase of outer mitochondrial membrane 7 homolog (yeast)	1.486755	1.5816338	1.845946
TONSL	tonsoku-like, DNA repair protein	1.621404	1.6948	1.8748091
TOP1	topoisomerase (DNA) I	-1.3552762	-1.6858242	-1.6854372
TOPBP1	topoisomerase (DNA) II binding protein 1	-1.1810086	-1.8708726	-2.1572924
TOPORS-AS1	TOPORS antisense RNA 1	1.4096272	1.7254361	1.7049854
TOR1AIP2	torsin A interacting protein 2	1.1741035	1.6883199	1.5712103
TOR1AIP2	torsin A interacting protein 2	-1.2884504	1.4671719	2.3630738
TOR2A	torsin family 2, member A	2.370988	2.3569193	2.7730842
TP53BP1	tumor protein p53 binding protein 1	-2.0234852	-2.9371543	-4.309627
TP53BP2	tumor protein p53 binding protein 2	-1.5380149	-1.9112757	-1.9892637
TP53I11	tumor protein p53 inducible protein 11	1.520799	-1.7353293	-3.4657912
TP53I13	tumor protein p53 inducible protein 13	-1.0391027	1.4964554	1.8667239
TP53I3	tumor protein p53 inducible protein 3	-1.2430416	-1.7327534	-2.11029
TP53I3	tumor protein p53 inducible protein 3	-1.2575425	-1.8119998	-2.1240957
TP53INP1	tumor protein p53 inducible nuclear protein 1	-1.7194221	-1.1985359	1.5191453
TPBG	trophoblast glycoprotein	-1.9836743	-2.7402332	-2.5329547
TPGS2	tubulin polyglutamylase complex subunit 2	2.6339545	4.4782815	7.65914
TPGS2	tubulin polyglutamylase complex subunit 2	1.5938638	-1.1216993	-1.1840922
TPGS2	tubulin polyglutamylase complex subunit 2	1.5195564	1.0844843	1.2292128
TPK1	thiamin pyrophosphokinase 1	-1.1177229	1.0867165	2.1448574
TPM1	tropomyosin 1 (alpha)	-1.1400719	-3.040107	-4.8973045
TPM1	tropomyosin 1 (alpha)	1.0690198	-2.5807464	-3.9498525
TPM1	tropomyosin 1 (alpha)	1.0993998	-2.4487731	-4.2675114
TPM2	tropomyosin 2 (beta)	-2.2110221	-2.6180768	-9.40815
TPM2	tropomyosin 2 (beta)	-1.8022143	-2.0391982	-6.6330075
TPM3	tropomyosin 3	-1.6131665	-1.9456362	-2.520708
TPM3	tropomyosin 3	-1.7502308	-2.051911	-2.4804535
TPM4	tropomyosin 4	-1.703221	-8.696246	-28.283438
TPM4	tropomyosin 4	-1.0071032	-5.838037	-17.007088
TPMT	thiopurine S-methyltransferase	1.2327058	2.336882	2.080252
TPP1	tripeptidyl peptidase I	3.417966	3.9849586	4.6334233
TPP1	tripeptidyl peptidase I	-2.0618618	-1.7593062	-1.8723537
TPR	translocated promoter region, nuclear basket protein	-2.0655816	-1.7428529	-2.2229311
TPRG1L	tumor protein p63 regulated 1-like	-1.2686318	1.5711764	2.5623827
TPRN	taperin	1.4564946	2.4200106	2.0110445
TPSG1	tryptase gamma 1	1.3140346	1.6342574	1.7823706
TPX2	TPX2, microtubule-associated	1.852386	-3.8607557	-5.16599
TRA2B	transformer 2 beta homolog (Drosophila)	-1.2654624	-2.0422382	-1.7597061
TRA2B	transformer 2 beta homolog (Drosophila)	-1.5976818	-2.4456475	-2.112548
TRABD2A	TraB domain containing 2A	-1.2635707	-1.3083596	-4.6338954
TRAF2	TNF receptor-associated factor 2	1.2635986	1.0857362	-1.8548822
TRAF3IP2	TRAF3 interacting protein 2	-1.1511269	-1.7106718	-3.0261102
TRAF3IP2	TRAF3 interacting protein 2	-1.2033355	-1.7081621	-2.6729236
TRAF7	TNF receptor-associated factor 7, E3 ubiquitin protein ligase	1.6669213	1.5373981	1.2147495
TRAIP	TRAF interacting protein	1.1850498	-2.9769557	-3.8422613
TRAM1	translocation associated membrane protein 1	2.4163067	1.1855302	-1.1054146
TRAM2	translocation associated membrane protein 2	1.7850175	-1.1640996	-1.0769291
TRAM2-AS1	TRAM2 antisense RNA 1 (head to head)	1.3810388	2.1805973	1.9650903
TRANK1	tetratricopeptide repeat and ankyrin repeat containing 1	-2.7721221	-2.5231223	-3.3723726
TRAP1	TNF receptor-associated protein 1	-1.7096871	-1.2900803	-1.493424
TRAPPC4	trafficking protein particle complex 4	1.5253427	1.7560779	1.6192456
TRAPPC6A	trafficking protein particle complex 6A	1.4967141	2.3898644	2.6406553
TRAPPC9	trafficking protein particle complex 9	-1.2677596	-1.2148467	1.5262036
TRIAP1	TP53 regulated inhibitor of apoptosis 1	1.5705662	1.0743071	1.0067964
TRIB1	tribbles pseudokinase 1	-2.4665031	-1.8958247	-1.995325
TRIM10	tripartite motif containing 10	-2.181682	2.163499	3.3453264
TRIM10	tripartite motif containing 10	3.9007742	3.5440888	1.5462743
TRIM14	tripartite motif containing 14	1.6825132	2.202989	1.3361623
TRIM15	tripartite motif containing 15	-2.3535738	1.6924843	1.5398529
TRIM16L	tripartite motif containing 16-like	-1.4372381	-3.2786117	-2.634134
TRIM22	tripartite motif containing 22	1.1203035	-1.631639	-3.0548568
TRIM24	tripartite motif containing 24	1.074792	1.01666	1.5655086
TRIM28	tripartite motif containing 28	-1.1030618	-1.7379225	-2.0522923
TRIM4	tripartite motif containing 4	1.3193088	1.7775323	2.0316865
TRIM41	tripartite motif containing 41	1.1569475	1.3061357	1.8778812
TRIM44	tripartite motif containing 44	-1.3439143	-1.5360113	-1.8738918
TRIM44	tripartite motif containing 44	-2.0174477	-2.2651029	-2.1431992
TRIM45	tripartite motif containing 45	-1.2703003	-1.0124494	-2.5938823
TRIM47	tripartite motif containing 47	1.0073357	1.0067877	-3.7244022
TRIM54	tripartite motif containing 54	-1.3637989	-1.5911206	-5.985904
TRIM56	tripartite motif containing 56	-1.309215	-1.2728599	-1.6309468
TRIM6	tripartite motif containing 6	1.2751793	-1.7319006	-1.9423381
TRIM65	tripartite motif containing 65	1.3856196	-1.0588316	-1.5668441
TRIM69	tripartite motif containing 69	1.6503958	1.5592885	1.7239017
TRIM8	tripartite motif containing 8	-1.6904485	-1.7869618	-2.6232944
TRIO	trio Rho guanine nucleotide exchange factor	-2.4892206	-4.1735387	-5.5827036
TRIO	trio Rho guanine nucleotide exchange factor	-1.3523251	-2.1994076	-2.974701
TRIOBP	TRIO and F-actin binding protein	-1.5914657	-1.5852255	-2.8145447
TRIP10	thyroid hormone receptor interactor 10	-1.5006838	-2.236068	-4.768687
TRIP13	thyroid hormone receptor interactor 13	1.9322214	-2.848402	-7.2295933
TRIP6	thyroid hormone receptor interactor 6	1.0653286	-1.5964916	-5.3332844
TRIQK	triple QxxK/R motif containing	1.201914	1.5758308	1.5328878
TRMT1	tRNA methyltransferase 1 homolog (S. cerevisiae)	-1.2859893	-1.5706967	-2.175881
TRMT10C	tRNA methyltransferase 10 homolog C (S. cerevisiae)	1.8484453	1.636338	1.5708902
TRMT44	tRNA methyltransferase 44 homolog (S. cerevisiae)	-1.5367756	1.276041	1.6415229
TRMT61A	tRNA methyltransferase 61A	1.0536615	1.0830206	-1.8587278
TROAP	trophinin associated protein	2.4315503	-2.2111235	-1.8118086
TROVE2	TROVE domain family, member 2	-1.501672	-1.3275456	-1.3797777
TRPT1	tRNA phosphotransferase 1	1.1577715	1.665624	1.3176801
TRPV1	transient receptor potential cation channel, subfamily V, member 1	-1.5980519	1.1773429	2.7967362
TRUB1	TruB pseudouridine (psi) synthase family member 1	1.7720789	1.428565	1.4133526
TSC2	tuberous sclerosis 2	-1.6165603	-1.3746939	-1.783657
TSC22D3	TSC22 domain family, member 3	3.8147724	7.170591	7.28412
TSEN54	TSEN54 tRNA splicing endonuclease subunit	1.7318962	1.8451749	2.3172157
TSFM	Ts translation elongation factor, mitochondrial	1.3074026	1.3095062	1.658216
TSKU	tsukushi, small leucine rich proteoglycan	-1.3784093	1.5456697	5.241734
TSNAX	translin-associated factor X	2.1748533	2.1069448	2.2968774
TSPAN1	tetraspanin 1	1.4584098	1.6157932	1.2083462
TSPAN10	tetraspanin 10	1.3454373	-1.3146092	-2.325233
TSPAN11	tetraspanin 11	2.7261584	2.2640345	2.323112
TSPAN12	tetraspanin 12	1.239482	1.93913	2.5979521
TSPAN14	tetraspanin 14	-1.1237658	-2.0705373	-2.3784945
TSPAN14	tetraspanin 14	-1.0526654	-1.649831	-1.8485034
TSPAN31	tetraspanin 31	-1.1768619	1.1240653	1.8849607
TSPAN33	tetraspanin 33	-1.5771977	1.8372189	2.968955

TSPAN4	tetraspanin 4	1.3704219	1.1921705	-1.5577847
TSPAN5	tetraspanin 5	1.604078	-1.6191281	-1.5732467
TSPO	translocator protein (18kDa)	1.3131983	1.0191799	-1.6998637
TSPYL1	TSPY-like 1	1.5377923	1.1377084	1.8732729
TSR2	TSR2, 20S rRNA accumulation, homolog (S. cerevisiae)	1.1214709	1.2413437	1.9160956
TST	thiosulfate sulfurtransferase (rhodanese)	-1.234491	2.9069338	5.6714306
TTC16	tetratricopeptide repeat domain 16	1.3826516	3.160437	4.651257
TTC17	tetratricopeptide repeat domain 17	-2.6129622	-1.9093969	-1.6973591
TTC19	tetratricopeptide repeat domain 19	1.0887161	1.2503074	1.9440941
TTC28	tetratricopeptide repeat domain 28	2.2104867	1.5452828	1.651664
TTC28	tetratricopeptide repeat domain 28	2.7971735	2.9395483	2.7369485
TTC3	tetratricopeptide repeat domain 3	-1.6013064	-2.29428	-1.8489182
TTC38	tetratricopeptide repeat domain 38	-1.298688	2.2549686	3.126163
TTC39C	tetratricopeptide repeat domain 39C	-1.0354228	2.040943	4.448155
TTC39C	tetratricopeptide repeat domain 39C	1.1139202	-1.2388705	-1.725126
TTC39C	tetratricopeptide repeat domain 39C	-1.5231124	2.628032	9.127344
TTC4	tetratricopeptide repeat domain 4	-1.8425276	-1.9347804	-1.8913156
TTC5	tetratricopeptide repeat domain 5	1.6708301	1.3024384	1.2400188
TTC7A	tetratricopeptide repeat domain 7A	1.461625	1.0658747	2.722108
TTC7B	tetratricopeptide repeat domain 7B	1.5812109	1.0689608	-1.2249709
TTC8	tetratricopeptide repeat domain 8	-1.3912753	-2.0979073	-2.2840905
TTF1	transcription termination factor, RNA polymerase I	-1.110818	-1.3802712	-1.5603197
TTF2	transcription termination factor, RNA polymerase II	1.1323038	-1.9548597	-2.4794507
TTPA	tocopherol (alpha) transfer protein	1.2649972	7.8621783	42.71024
TTPAL	tocopherol (alpha) transfer protein-like	1.9089388	1.9722452	1.5580982
TTR	transthyretin	-1.844131	9.79993	16.395964
TTYH1	tweety family member 1	2.3509786	2.4451468	2.8689623
TTYH3	tweety family member 3	-1.454623	-3.5172346	-4.472554
TUBA1A	tubulin, alpha 1a	1.2820867	-1.7138852	-2.0218437
TUBA1B	tubulin, alpha 1b	1.340098	-1.8379178	-1.9243131
TUBA1C	tubulin, alpha 1c	1.6016548	-1.0754342	-1.3124907
TUBA1C	tubulin, alpha 1c	1.2876034	-1.7246546	-2.0140345
TUBA3C	tubulin, alpha 3c	1.1026455	-1.7813298	-2.6052706
TUBA3D	tubulin, alpha 3d	4.3299055	4.982668	3.505364
TUBA3FP	tubulin, alpha 3f, pseudogene	3.479191	1.8813043	1.2146705
TUBA4A	tubulin, alpha 4a	-1.0103264	-2.393646	-2.8332038
TUBA4A	tubulin, alpha 4a	1.099152	-2.2282586	-2.5771382
TUBA4A	tubulin, alpha 4a	-1.0042201	-2.548149	-2.8623388
TUBA8	tubulin, alpha 8	2.7871146	1.5152625	1.065819
TUBB	tubulin, beta class I	1.2152779	-1.8058918	-1.8141431
TUBB	tubulin, beta class I	-1.3974179	-3.092624	-3.6468885
TUBB	tubulin, beta class I	-1.0249331	-2.353948	-2.6628742
TUBB2A	tubulin, beta 2A class IIa	1.377666	1.7857834	2.8382883
TUBB2A	tubulin, beta 2A class IIa	1.1387821	1.4155594	2.1701515
TUBB3	tubulin, beta 3 class III	-1.0078712	-2.667837	-1.269418
TUBB8	tubulin, beta 8 class VIII	1.669093	1.3216276	1.9281083
TUBBP5	tubulin, beta pseudogene 5	2.6771076	2.8774972	2.5352757
TUBE1	tubulin, epsilon 1	-1.7851696	-1.4887947	1.1837208
TUBGCP3	tubulin, gamma complex associated protein 3	-1.3938998	-1.9720509	-2.044
TUFT1	tuftelin 1	1.2924197	-3.8976843	-2.1352117
TUG1	taurine up-regulated 1 (non-protein coding)	-1.6014684	-2.3776464	-2.455516
TUG1	taurine up-regulated 1 (non-protein coding)	-1.566867	-1.7989405	-1.423432
TUG1	taurine up-regulated 1 (non-protein coding)	1.1847916	1.0025276	-3.2062192
TUG1	taurine up-regulated 1 (non-protein coding)	-1.8392833	-2.1373405	-2.158254
TULP3	tubby like protein 3	-1.0595886	-1.5292869	-1.3128108
TUSC1	tumor suppressor candidate 1	2.2111402	2.338347	9.077772
TUSC1	tumor suppressor candidate 1	1.7145618	1.5120256	1.3174974
TUSC2	tumor suppressor candidate 2	1.2072189	1.3302509	2.6350894
TVP23C	trans-golgi network vesicle protein 23 homolog C (S. cerevisiae)	-1.6866091	-2.105342	-1.9438887
TWF2	twinfilin actin-binding protein 2	-1.0080278	1.5543175	-1.4577645
TWIST1	twist family bHLH transcription factor 1	1.8248742	-1.0187727	-1.7972581
TWIST2	twist family bHLH transcription factor 2	1.9915963	-1.1440732	-1.2006029
TWIST2	twist family bHLH transcription factor 2	2.04418	-1.1433882	-1.1744608
TWSG1	twisted gastrulation BMP signaling modulator 1	2.2713113	1.1101911	1.3866208
TXN	thioredoxin	1.4200445	-1.2160598	-1.5787983
TXNDC17	thioredoxin domain containing 17	1.5748186	2.627646	2.4188788
TXNDC5	thioredoxin domain containing 5 (endoplasmic reticulum)	-1.4791574	-2.3401487	-1.758228
TXNDC9	thioredoxin domain containing 9	-1.0834126	-1.4663643	-1.9016052
TXNIP	thioredoxin interacting protein	-1.7501258	1.2271597	1.9373931
TXNL4A	thioredoxin-like 4A	1.872261	1.5387684	1.8249425
TXNRD1	thioredoxin reductase 1	1.7876393	1.0131028	1.0549473
TYMP	thymidine phosphorylase	1.2873331	1.7312553	1.8464438
TYMS	thymidylate synthetase	1.5092738	-2.0457098	-3.8565488
TYMSOS	TYMS opposite strand	1.8848329	1.6786672	-1.4957443
TYRO3	TYRO3 protein tyrosine kinase	-1.2841785	-1.7106692	-4.3519244
UBA1	ubiquitin-like modifier activating enzyme 1	-1.8316683	-2.121717	-2.01686
UBA5	ubiquitin-like modifier activating enzyme 5	1.0863166	1.3502938	2.000863
UBA6-AS1	UBA6 antisense RNA 1 (head to head)	1.2046803	1.8985851	-1.2748983
UBALD2	UBA-like domain containing 2	-1.4379098	2.3483965	1.1537535
UBAP2	ubiquitin associated protein 2	-1.1848397	-1.4328179	-1.5438551
UBAP2L	ubiquitin associated protein 2-like	-1.2331461	-1.4807771	-1.8343257
UBD	ubiquitin D	-2.9938493	-1.3830299	-4.3534927
UBE2B	ubiquitin-conjugating enzyme E2B	1.3961169	1.4053673	2.2360475
UBE2D2	ubiquitin-conjugating enzyme E2D 2	1.7861425	1.5725338	1.4492784
UBE2D4	ubiquitin-conjugating enzyme E2D 4 (putative)	1.1533546	-1.0659378	1.7727066
UBE2D4	ubiquitin-conjugating enzyme E2D 4 (putative)	-1.4361626	-1.3858329	1.5707061
UBE2E3	ubiquitin-conjugating enzyme E2E 3	-1.8451437	-1.9814858	-1.703284
UBE2H	ubiquitin-conjugating enzyme E2H	-1.2891237	-1.8130538	-1.3307247
UBE2H	ubiquitin-conjugating enzyme E2H	-1.1732703	-2.0712988	-2.057962
UBE2J1	ubiquitin-conjugating enzyme E2, J1	1.1038879	1.5132581	1.5147855
UBE2MP1	ubiquitin-conjugating enzyme E2M pseudogene 1	1.6124462	1.3998451	-1.198794
UBE2NL	ubiquitin-conjugating enzyme E2N-like (gene/pseudogene)	-1.2455419	-2.4778612	-2.3842068
UBE2Q2P1	ubiquitin-conjugating enzyme E2Q family member 2 pseudogene 1	-2.409527	-2.2906625	-2.4843776
UBE2R2	ubiquitin-conjugating enzyme E2R 2	1.4316543	1.6830806	1.8420658
UBE2S	ubiquitin-conjugating enzyme E2S	2.4257715	-1.7193694	-1.8328547
UBE2T	ubiquitin-conjugating enzyme E2T	1.814047	-2.2264695	-2.2870283
UBL3	ubiquitin-like 3	-1.9139675	-1.2393385	1.1657172
UBP1	upstream binding protein 1 (LBP-1a)	1.5516324	1.1847689	1.282048
UBR2	ubiquitin protein ligase E3 component n-recognin 2	-1.5268648	-1.2192835	1.0132053
UBR4	ubiquitin protein ligase E3 component n-recognin 4	-1.9378046	-1.4082363	-2.800067
UBTD2	ubiquitin domain containing 2	1.2191141	1.1550066	1.8682739
UBXN2B	UBX domain protein 2B	1.1501069	1.442161	2.171455
UBXN4	UBX domain protein 4	-1.8541377	-1.8056283	-1.6308675
UCHL3	ubiquitin carboxyl-terminal esterase L3 (ubiquitin thiolesterase)	1.0900831	-1.5120174	-1.3109522
UCK2	uridine-cytidine kinase 2	1.099096	-1.7086431	-2.1207526
UCP3	uncoupling protein 3 (mitochondrial, proton carrier)	3.061019	3.4800334	4.126696
UFL1	UFM1-specific ligase 1	1.2330828	1.5908277	1.9236139
UFSP1	UFM1-specific peptidase 1 (non-functional)	1.5937632	1.9842337	2.0048237
UFSP1	UFM1-specific peptidase 1 (non-functional)	1.794907	2.5810735	2.2529085

UGCG	UDP-glucose ceramide glucosyltransferase	-1.5144477	-2.8551927	-2.296258
UGGT1	UDP-glucose glycoprotein glucosyltransferase 1	-2.7138498	-2.300374	-2.55783
UGGT2	UDP-glucose glycoprotein glucosyltransferase 2	-1.2552103	-1.671352	-1.324174
UGT1A6	UDP glucuronosyltransferase 1 family, polypeptide A6	-2.2078876	-1.6208925	1.9604409
UGT1A6	UDP glucuronosyltransferase 1 family, polypeptide A6	-2.931282	-2.6702816	-1.0805223
UGT1A8	UDP glucuronosyltransferase 1 family, polypeptide A8	-2.3891256	-1.7592556	1.6907799
UGT2B10	UDP glucuronosyltransferase 2 family, polypeptide B10	-4.7804017	3.1380432	5.8686266
UGT2B11	UDP glucuronosyltransferase 2 family, polypeptide B11	-7.4860606	2.5468407	5.077058
UGT2B15	UDP glucuronosyltransferase 2 family, polypeptide B15	-10.083585	3.2052414	13.672687
UGT2B4	UDP glucuronosyltransferase 2 family, polypeptide B4	-9.690855	6.1332164	18.247618
UGT2B7	UDP glucuronosyltransferase 2 family, polypeptide B7	-3.8165128	3.1128411	2.2278905
UHRF1	ubiquitin-like with PHD and ring finger domains 1	2.2733889	-5.1836185	-13.19537
ULK1	unc-51 like autophagy activating kinase 1	-1.9703156	-1.6336542	-1.2570094
ULK3	unc-51 like kinase 3	-2.162189	-1.4332218	-1.1338156
ULK4	unc-51 like kinase 4	1.4404542	3.820628	1.5918238
UMAD1	UBAP1-MVB12-associated (UMA) domain containing 1	1.3782643	1.3634343	1.7904013
UNC80	unc-80 homolog (C. elegans)	-1.4379556	-2.0602722	-2.387763
UNC93B1	unc-93 homolog B1 (C. elegans)	-1.0527751	-1.0182719	-1.5689387
UNK	unkempt family zinc finger	-1.0645567	-1.5321409	-1.3190084
UNKL	unkempt family zinc finger-like	-1.3826602	-2.160972	-2.613533
UPB1	ureidopropionase, beta	-1.2247928	3.373852	9.15406
UPB1	ureidopropionase, beta	-1.2338521	2.7410476	6.261212
UPF3A	UPF3 regulator of nonsense transcripts homolog A (yeast)	-1.3360355	-1.5188196	-1.3022343
UPP1	uridine phosphorylase 1	-1.1075424	-1.5937316	-2.8083172
UPP1	uridine phosphorylase 1	-1.2840157	-1.478443	-3.3601005
UPRT	uracil phosphoribosyltransferase (FUR1) homolog (S. cerevisiae)	1.7142034	1.4218341	1.5472398
UQCC2	ubiquinol-cytochrome c reductase complex assembly factor 2	-1.21585	-1.6624464	-1.4304681
UQCR10	ubiquinol-cytochrome c reductase, complex III subunit X	1.4931631	2.2139013	1.2839375
UQCR10	ubiquinol-cytochrome c reductase, complex III subunit X	1.8730658	2.0853195	2.0776546
UQCR10	ubiquinol-cytochrome c reductase, complex III subunit X	1.877099	2.0684128	1.8632398
UQCR11	ubiquinol-cytochrome c reductase, complex III subunit XI	1.4486309	1.7433391	1.7611789
UQCRB	ubiquinol-cytochrome c reductase binding protein	2.0569725	2.371686	1.8540581
UQCRBP1	ubiquinol-cytochrome c reductase binding protein pseudogene 1	1.8676947	2.0637965	1.656246
UQCRC2	ubiquinol-cytochrome c reductase core protein II	1.8415837	2.4602137	1.5054017
UQCRFS1	ubiquinol-cytochrome c reductase, Rieske iron-sulfur polypeptide 1	2.2911599	2.5149357	2.234667
UQCRH	ubiquinol-cytochrome c reductase hinge protein	1.9395518	1.9834002	1.4008876
UQCRH	ubiquinol-cytochrome c reductase hinge protein	1.8137017	1.7619932	1.1821145
UQCRQ	ubiquinol-cytochrome c reductase, complex III subunit VII, 9.5kDa	1.3537815	1.844973	2.2014663
URB1	URB1 ribosome biogenesis 1 homolog (S. cerevisiae)	-1.1132623	-1.0897733	-1.555177
URI1	URI1, prefoldin-like chaperone	-1.3223908	-1.6781137	-1.4748297
URM1	ubiquitin related modifier 1	1.4713981	1.5070088	1.1527643
USE1	unconventional SNARE in the ER 1 homolog (S. cerevisiae)	1.2767277	1.6488404	1.4740711
USP1	ubiquitin specific peptidase 1	1.5147138	-1.2678192	-1.2421342
USP10	ubiquitin specific peptidase 10	-1.36322	-1.6498172	-1.3952606
USP11	ubiquitin specific peptidase 11	-1.7835416	-2.3011131	-2.2871666
USP30-AS1	USP30 antisense RNA 1	-1.7685281	2.7628825	6.189751
USP32	ubiquitin specific peptidase 32	-1.7569594	-1.6428313	-1.3160051
USP38	ubiquitin specific peptidase 38	1.034494	1.1581612	1.9880344
USP42	ubiquitin specific peptidase 42	-1.769688	-1.6890303	-1.4367356
USP46-AS1	USP46 antisense RNA 1	2.255201	2.498888	2.9786096
USP47	ubiquitin specific peptidase 47	-1.7184014	-1.721964	-1.0916201
USP53	ubiquitin specific peptidase 53	1.2596124	1.9932954	2.5425515
USP53	ubiquitin specific peptidase 53	1.1895672	2.7154324	3.1920142
USP54	ubiquitin specific peptidase 54	-1.4815493	-1.5892022	-1.407559
UTF1	undifferentiated embryonic cell transcription factor 1	1.8291652	2.2480845	2.1487076
UTP14C	UTP14, U3 small nucleolar ribonucleoprotein, homolog C (yeast)	-1.6333292	-1.5845855	-1.1689303
UTP20	UTP20, small subunit (SSU) processome component, homolog (yeast)	-1.543942	-1.7730811	-2.8401637
UVSSA	UV-stimulated scaffold protein A	-1.7564945	-1.3315622	-1.5408217
UVSSA	UV-stimulated scaffold protein A	3.692285	4.098132	5.4669423
UXS1	UDP-glucuronate decarboxylase 1	-1.2516806	-2.4129808	-2.272415
VAMP2	vesicle-associated membrane protein 2 (synaptobrevin 2)	2.0835135	2.3228822	2.715669
VAMP2	vesicle-associated membrane protein 2 (synaptobrevin 2)	1.3703536	1.7017906	3.0775182
VARS	valyl-tRNA synthetase	-1.4695287	-1.7637106	-2.567299
VARS2	valyl-tRNA synthetase 2, mitochondrial	1.0383168	1.6303085	1.6026074
VASN	vasorin	1.2334415	1.6243594	2.5721579
VASP	vasodilator-stimulated phosphoprotein	1.3534473	-1.2495553	-2.3828568
VAX2	ventral anterior homeobox 2	1.5106289	1.1482983	1.234001
VCAN	versican	-6.7809415	-16.657366	-28.927488
VCL	vinculin	-1.1154671	-2.283949	-3.1155615
VCX2	variable charge, X-linked 2	-1.577596	-1.2132796	-1.9779264
VDAC2	voltage-dependent anion channel 2	1.5887083	1.4953209	1.0976368
VDAC2	voltage-dependent anion channel 2	1.6186129	1.5378746	1.1140548
VDR	vitamin D (1,25- dihydroxyvitamin D3) receptor	-1.1259606	-3.2375834	-8.583275
VEGFA	vascular endothelial growth factor A	-5.0256066	-3.4137156	-2.4879882
VEGFA	vascular endothelial growth factor A	-4.687705	-2.7314382	-1.8753235
VEGFC	vascular endothelial growth factor C	-1.5253464	-4.4232845	-9.31497
VGF	VGF nerve growth factor inducible	-2.0238688	-2.066892	-3.8007636
VGLL3	vestigial-like family member 3	4.1210294	1.2642033	-2.0478609
VGLL4	vestigial-like family member 4	-1.7922369	-1.5233836	-1.5034763
VIL1	villin 1	-1.2515653	4.118247	11.176674
VKORC1	vitamin K epoxide reductase complex, subunit 1	-1.0200517	1.4474317	1.5952703
VMA21	VMA21 vacuolar H+-ATPase homolog (S. cerevisiae)	1.8601265	1.7253783	1.076579
VMAC	vimentin-type intermediate filament associated coiled-coil protein	1.6494422	1.9375985	2.5520895
VMP1	vacuole membrane protein 1	-1.6531397	-1.7763114	-1.5288463
VNN1	vanin 1	-31.623087	-2.1883047	-5.311928
VOPP1	vesicular, overexpressed in cancer, prosurvival protein 1	-1.3241867	-2.2403882	-3.1420372
VPREB3	pre-B lymphocyte 3	1.7500635	1.7926913	2.882882
VPS13A	vacuolar protein sorting 13 homolog A (S. cerevisiae)	-2.034528	-1.4321343	-1.3488082
VPS13B	vacuolar protein sorting 13 homolog B (yeast)	-1.808481	1.0024797	-1.0452476
VPS13C	vacuolar protein sorting 13 homolog C (S. cerevisiae)	-1.8848256	-1.1762155	-1.6910942
VPS16	vacuolar protein sorting 16 homolog (S. cerevisiae)	-1.5771972	-1.3286505	-1.0386307
VPS36	vacuolar protein sorting 36 homolog (S. cerevisiae)	1.3814867	1.6186861	2.217927
VPS37B	vacuolar protein sorting 37 homolog B (S. cerevisiae)	-1.4481181	-2.7024617	-3.7084706
VPS37D	vacuolar protein sorting 37 homolog D (S. cerevisiae)	1.1252285	2.9745052	3.696798
VPS41	vacuolar protein sorting 41 homolog (S. cerevisiae)	-1.2618883	-1.1151402	-1.5603732
VPS51	vacuolar protein sorting 51 homolog (S. cerevisiae)	-1.1356075	-1.1241566	-2.3758793
VPS53	vacuolar protein sorting 53 homolog (S. cerevisiae)	1.8613588	1.8011341	1.7719436
VRK1	vaccinia related kinase 1	1.5771396	-2.3584747	-2.0803306
VSIG10	V-set and immunoglobulin domain containing 10	-1.3162206	-1.0400493	2.9549787
VSIG10	V-set and immunoglobulin domain containing 10	-1.6311637	-1.1344343	2.6871784
VSIG10L	V-set and immunoglobulin domain containing 10 like	-1.787839	-1.5845871	-1.2364978
VSTM2L	V-set and transmembrane domain containing 2 like	5.801	3.7387247	2.2269776
VSX1	visual system homeobox 1	4.3537836	7.519342	4.8892245
VTI1B	vesicle transport through interaction with t-SNAREs 1B	1.2238232	1.0592369	1.5419394
VTN	vitronectin	-2.7157614	5.0607	16.277094
VWA1	von Willebrand factor A domain containing 1	-1.3724569	1.580586	3.3664954
VWCE	von Willebrand factor C and EGF domains	-1.3132699	-2.9767122	-12.683926
WARS	tryptophanyl-tRNA synthetase	-2.1608236	-2.5825498	-3.2448244
WASH1	WAS protein family homolog 1	1.7047675	2.1372337	2.2395203

WASH5P	WAS protein family homolog 5 pseudogene	2.6391797	2.0102808	2.715292
WASL	Wiskott-Aldrich syndrome-like	-1.1216933	1.3169632	1.6095661
WBP1	WW domain binding protein 1	-1.0285832	1.0668042	1.674522
WBSCR22	Williams Beuren syndrome chromosome region 22	-1.2029151	-1.6108407	-1.6632884
WBSCR27	Williams Beuren syndrome chromosome region 27	-1.5845311	-1.8252472	-1.5606453
WDFY3-AS2	WDFY3 antisense RNA 2	1.2207475	1.538643	-1.0271889
WDHD1	WD repeat and HMG-box DNA binding protein 1	1.0749838	-4.8133044	-6.351327
WDR1	WD repeat domain 1	-1.2328026	-2.1379285	-2.758376
WDR1	WD repeat domain 1	-1.276608	-2.7570174	-3.9639375
WDR13	WD repeat domain 13	-1.1773632	1.041701	-1.5509453
WDR18	WD repeat domain 18	1.7250761	1.502528	1.3688248
WDR19	WD repeat domain 19	-1.6620655	-1.2555171	1.3337766
WDR24	WD repeat domain 24	-1.5658028	-1.3033152	-1.1512271
WDR3	WD repeat domain 3	1.7045109	1.7061553	1.2112489
WDR33	WD repeat domain 33	1.2289716	1.5695804	2.1729956
WDR43	WD repeat domain 43	1.5830164	1.1778663	-1.1920475
WDR45	WD repeat domain 45	-1.4464709	1.0109754	-1.5432528
WDR59	WD repeat domain 59	-1.5403752	-1.4609135	-1.2260917
WDR6	WD repeat domain 6	-1.4543763	-1.6332687	-1.7407097
WDR62	WD repeat domain 62	1.2370036	-3.9075794	-4.3323307
WDR7	WD repeat domain 7	-1.0855706	-1.0891976	1.5158329
WDR75	WD repeat domain 75	1.0967878	-1.2804081	-1.5369301
WDR77	WD repeat domain 77	1.033947	-1.3340023	-2.1312344
WDR82	WD repeat domain 82	1.7268914	1.4510019	1.382684
WDR83	WD repeat domain 83	1.1299239	1.3535681	1.5627706
WDR90	WD repeat domain 90	3.110055	3.092456	3.8415458
WDR90	WD repeat domain 90	-1.5463607	-2.1948185	-2.0783162
WDR93	WD repeat domain 93	1.7017646	1.5758783	1.4300588
WDTC1	WD and tetratricopeptide repeats 1	2.3701956	2.4245741	2.5911436
WEE1	WEE1 G2 checkpoint kinase	-1.2107095	-2.1743138	-1.9841433
WFDC21P	WAP four-disulfide core domain 21, pseudogene	4.7812896	1.355199	1.9372457
WFDC3	WAP four-disulfide core domain 3	2.2508106	2.5833673	1.7688813
WFS1	Wolfram syndrome 1 (wolframin)	-1.0604799	-1.6619662	-2.1564667
WHAMM	WAS protein homolog associated with actin, golgi membranes and microtubules	-2.0350964	-2.0132694	-1.497598
WHAMMP1	WAS protein homolog associated with actin, golgi membranes and microtubules pseudogene	-2.140555	-2.248876	-1.5559392
WIPI2	WD repeat domain, phosphoinositide interacting 2	1.1129981	1.1165361	1.6073555
WISP2	WNT1 inducible signaling pathway protein 2	7.319257	7.83261	4.0856004
WNT3A	wingless-type MMTV integration site family, member 3A	2.1667528	2.7853305	3.655122
WNT5A	wingless-type MMTV integration site family, member 5A	2.5365124	1.3044631	-1.2963457
WRN	Werner syndrome, RecQ helicase-like	-1.4822738	-1.6442382	-1.7970146
WSB1	WD repeat and SOCS box containing 1	-1.68079	-2.6016855	-1.9582819
WTAP	Wilms tumor 1 associated protein	-1.8537847	-1.6255047	-2.2693293
WWC3	WWC family member 3	-1.5808148	-1.7414521	-1.4211206
XAGE1B	X antigen family, member 1B	1.0262411	-1.4654434	-1.612464
XBP1	X-box binding protein 1	-1.6037014	2.0213575	2.461777
XCR1	chemokine (C motif) receptor 1	2.3704667	2.6217961	3.1727984
XDH	xanthine dehydrogenase	-2.5873218	6.5190773	11.871235
XIST	X inactive specific transcript (non-protein coding)	-2.762974	-1.9021353	-1.3116395
XIST	X inactive specific transcript (non-protein coding)	-3.2956486	-2.2328048	-1.5014542
XIST	X inactive specific transcript (non-protein coding)	-4.0983014	-2.394006	-1.631471
XLOC_l2_001324		1.3862603	1.5310457	1.8221476
XLOC_l2_003882		1.2321398	-1.0060256	-1.6511004
XLOC_l2_006578		2.9755216	2.5459433	3.576737
XLOC_l2_008285		1.0607778	1.2098981	1.7078657
XLOC_l2_009136		-1.0497085	-1.2010036	-2.3223562
XLOC_l2_010751		-1.5780747	-1.2229894	-3.3274379
XLOC_l2_013383		2.9113889	3.7998931	4.2795386
XLOC_l2_013410		-3.7698119	-3.4023461	-3.2511659
XLOC_l2_013837		1.825868	3.0531468	3.1112926
XLOC_l2_015203		1.3911805	1.2349669	1.7102096
XLOC_l2_015464		1.259627	-1.6149414	-1.865257
XPA	xeroderma pigmentosum, complementation group A	1.664114	1.9590639	1.9887136
XPO1	exportin 1	-1.2391106	-2.1513255	-1.415942
XPO6	exportin 6	-1.3891972	-1.6147358	-1.9353722
XPOT	exportin, tRNA	-2.0773613	-4.3080378	-6.226128
XRCC1	X-ray repair complementing defective repair in Chinese hamster cells 1	-1.1662495	-1.8452785	-2.1986299
XRCC3	X-ray repair complementing defective repair in Chinese hamster cells 3	1.5611719	-1.8416846	-2.4778655
XRCC3	X-ray repair complementing defective repair in Chinese hamster cells 3	-2.2875752	-2.0549426	-1.755652
XRCC5	X-ray repair complementing defective repair in Chinese hamster cells 5 (double-strand-break	-1.1039605	-1.5231419	-2.003157
XXYLT1	xyloside xylosyltransferase 1	1.2651132	-1.5754465	-1.3015803
YAP1	Yes-associated protein 1	-1.003007	-1.4842753	-2.0163326
YARS	tyrosyl-tRNA synthetase	-2.0882668	-2.9920268	-2.2954688
YBEY	ybeY metallopeptidase (putative)	1.0424179	1.5247239	1.4866661
YBEY	ybeY metallopeptidase (putative)	1.070574	1.621406	1.67693
YBX1	Y box binding protein 1	-1.4268713	-2.010268	-2.078887
YBX3	Y box binding protein 3	-1.2962346	-1.817404	-3.7066708
YBX3	Y box binding protein 3	1.1103079	-1.3452299	-2.5119038
YDJC	YdjC homolog (bacterial)	1.6162155	1.2066399	-1.0962423
YEATS2	YEATS domain containing 2	1.5269393	1.1786711	-1.1568997
YIF1A	Yip1 interacting factor homolog A (S. cerevisiae)	1.2484401	1.5266407	1.628007
YIF1B	Yip1 interacting factor homolog B (S. cerevisiae)	-1.3818276	-1.7225075	-2.265475
YIPF5	Yip1 domain family, member 5	-1.8324828	-1.9473635	-2.0459666
YPEL3	yippee-like 3 (Drosophila)	-1.5610712	-1.0227582	1.8744646
YPEL4	yippee-like 4 (Drosophila)	3.0826159	3.191976	4.1137495
YTHDF3	YTH N(6)-methyladenosine RNA binding protein 3	1.2807937	1.4393606	1.677553
YWHAQ	tyrosine 3-monooxygenase/tryptophan 5-monooxygenase activation protein, theta	-1.0542235	-1.7000359	-1.9667122
YWHAQ	tyrosine 3-monooxygenase/tryptophan 5-monooxygenase activation protein, theta	-1.3492262	-2.2124534	-2.6505215
YWHAZ	tyrosine 3-monooxygenase/tryptophan 5-monooxygenase activation protein, zeta	-1.2793742	-1.9635524	-2.2266395
YWHAZ	tyrosine 3-monooxygenase/tryptophan 5-monooxygenase activation protein, zeta	-1.5319822	-2.5852392	-3.1985729
YY2	YY2 transcription factor	4.0005784	2.816456	1.7838954
YY2	YY2 transcription factor	-1.3739944	-1.6339532	-1.9827458
ZAK	sterile alpha motif and leucine zipper containing kinase AZK	1.7773058	-1.2094699	-1.3098646
ZASP	ZO-2 associated speckle protein	1.6501359	1.3013995	1.7198147
ZBED3	zinc finger, BED-type containing 3	1.1093074	3.5453165	3.6699934
ZBED3-AS1	ZBED3 antisense RNA 1	2.4148088	10.871209	7.3092895
ZBED5-AS1	ZBED5 antisense RNA 1	1.0545892	1.7515529	2.264988
ZBTB17	zinc finger and BTB domain containing 17	-1.1769756	-1.3901227	-1.6107696
ZBTB20	zinc finger and BTB domain containing 20	-2.479777	-1.124156	-1.1255593
ZBTB21	zinc finger and BTB domain containing 21	-1.9037334	-1.8604015	-1.2551214
ZBTB21	zinc finger and BTB domain containing 21	-1.7068009	-1.3593075	1.0591966
ZBTB3	zinc finger and BTB domain containing 3	1.8433654	1.9861863	3.6230097
ZBTB40	zinc finger and BTB domain containing 40	-1.643443	-1.6593828	-1.6556766
ZBTB42	zinc finger and BTB domain containing 42	1.243208	1.9251584	2.8215756
ZBTB43	zinc finger and BTB domain containing 43	-1.3516791	-1.5335386	-1.6112036
ZBTB45	zinc finger and BTB domain containing 45	1.7892581	1.5888093	1.4089756
ZC3H11A	zinc finger CCCH-type containing 11A	-2.0931923	-2.1754808	-2.3157947
ZC3H11A	zinc finger CCCH-type containing 11A	-1.4995456	-1.5844935	-1.578702
ZC3H12A	zinc finger CCCH-type containing 12A	-2.6558983	-3.383549	-3.8200243
ZC3H14	zinc finger CCCH-type containing 14	-1.5217742	-1.8578426	-1.6967531

ZC3H14	zinc finger CCCH-type containing 14	-1.6409074	-1.3817552	-1.3655176
ZC3H3	zinc finger CCCH-type containing 3	1.206067	1.8821334	3.3401499
ZC3H7B	zinc finger CCCH-type containing 7B	2.3552732	2.46705	2.4131799
ZC3HAV1	zinc finger CCCH-type, antiviral 1	-1.512397	-1.4046838	-1.4507595
ZC3HAV1L	zinc finger CCCH-type, antiviral 1-like	1.5373932	1.6226082	1.7042302
ZCCHC18	zinc finger, CCHC domain containing 18	1.7018132	1.6352814	1.8181584
ZCCHC6	zinc finger, CCHC domain containing 6	-1.4672629	-1.0267806	1.7099221
ZCCHC9	zinc finger, CCHC domain containing 9	-1.1633221	1.3146626	1.6954622
ZDHHC13	zinc finger, DHHC-type containing 13	-1.1206721	-1.790932	-1.7160473
ZDHHC14	zinc finger, DHHC-type containing 14	-1.7346967	1.3542634	1.8671788
ZDHHC20	zinc finger, DHHC-type containing 20	3.2872486	3.6341827	3.8227825
ZDHHC23	zinc finger, DHHC-type containing 23	1.2581953	2.156439	1.4249778
ZDHHC3	zinc finger, DHHC-type containing 3	1.5240157	1.8419687	3.0664968
ZDHHC3	zinc finger, DHHC-type containing 3	1.4677196	1.4395779	1.8952031
ZDHHC7	zinc finger, DHHC-type containing 7	1.0967426	-1.3559935	-1.7316997
ZDHHC8	zinc finger, DHHC-type containing 8	1.5143641	1.1559919	-1.279134
ZFAND2A	zinc finger, AN1-type domain 2A	-1.4335252	1.2281417	1.5718293
ZFAND5	zinc finger, AN1-type domain 5	-2.4293857	-2.6907837	-2.7952542
ZFAND5	zinc finger, AN1-type domain 5	-1.8206513	-1.9918053	-2.081536
ZFC3H1	zinc finger, C3H1-type containing	-1.9430559	-1.6495564	-2.0137079
ZFC3H1	zinc finger, C3H1-type containing	-1.8056629	-1.4403992	-2.0068595
ZFP30	ZFP30 zinc finger protein	1.8418658	1.9973589	1.9964744
ZFP36	ZFP36 ring finger protein	-1.39396	1.3826487	2.6079633
ZFP36L1	ZFP36 ring finger protein-like 1	-2.0987895	-2.0354486	-1.9625725
ZFP36L2	ZFP36 ring finger protein-like 2	2.0812209	2.2359447	1.4411701
ZFP62	ZFP62 zinc finger protein	1.3409096	1.8215029	1.1842138
ZFP91	ZFP91 zinc finger protein	-1.928	-2.5268414	-2.2694616
ZFP91	ZFP91 zinc finger protein	-1.6901438	-2.3525274	-2.195241
ZFYVE26	zinc finger, FYVE domain containing 26	-1.6659677	-1.6131965	-1.0434268
ZFYVE28	zinc finger, FYVE domain containing 28	-1.3225607	1.3694633	1.955949
ZG16B	zymogen granule protein 16B	-2.3113472	1.0306636	-3.9278286
ZHX1-C8orf76	ZHX1-C8orf76 readthrough	-1.0137434	-1.701197	1.0653046
ZHX3	zinc fingers and homeoboxes 3	1.3180299	1.7311724	1.9126835
ZMAT2	zinc finger, matrin-type 2	1.1393399	1.0480599	1.962787
ZMAT5	zinc finger, matrin-type 5	-1.0441302	1.6136581	1.9755801
ZMIZ1-AS1	ZMIZ1 antisense RNA 1	-1.5754629	-1.698797	-3.5265827
ZMYND15	zinc finger, MYND-type containing 15	1.059582	1.9348084	1.4040154
ZNF143	zinc finger protein 143	-1.3031906	-1.7636862	-2.024947
ZNF18	zinc finger protein 18	1.0155375	1.2880844	1.5574834
ZNF189	zinc finger protein 189	1.3386766	2.5528538	3.4536955
ZNF195	zinc finger protein 195	-1.8768629	-2.0351963	-2.2293177
ZNF2	zinc finger protein 2	1.9543439	2.0698903	1.962624
ZNF205	zinc finger protein 205	2.8392599	2.8932452	3.4663785
ZNF212	zinc finger protein 212	-1.3961939	-1.6571633	-1.269183
ZNF215	zinc finger protein 215	1.4434794	1.0249306	-1.7269171
ZNF217	zinc finger protein 217	-2.1673973	-2.8569632	-3.0972981
ZNF219	zinc finger protein 219	1.8862832	1.4925563	-1.9070274
ZNF226	zinc finger protein 226	1.0328234	1.7155492	2.2016811
ZNF24	zinc finger protein 24	-1.4462026	-1.7305652	-1.3641822
ZNF252P	zinc finger protein 252, pseudogene	1.4184002	2.0340285	1.7808827
ZNF268	zinc finger protein 268	1.4203526	1.1397223	1.750995
ZNF281	zinc finger protein 281	-2.2745702	-3.1351182	-2.60612
ZNF283	zinc finger protein 283	1.5645388	1.3364209	-1.2394936
ZNF284	zinc finger protein 284	1.7263883	2.1747227	1.7212598
ZNF30	zinc finger protein 30	1.7055167	1.8717529	3.0110915
ZNF322	zinc finger protein 322	1.7733723	1.8562324	1.9555577
ZNF326	zinc finger protein 326	-1.6724501	-1.7504897	-1.8483398
ZNF329	zinc finger protein 329	2.4365716	2.4009712	3.032729
ZNF335	zinc finger protein 335	-1.4964668	-1.6758835	-1.773258
ZNF33B	zinc finger protein 33B	1.1829536	1.6680572	1.1263449
ZNF350	zinc finger protein 350	1.5767412	1.8887085	1.7317848
ZNF362	zinc finger protein 362	1.5255007	1.5702773	2.0507116
ZNF37A	zinc finger protein 37A	-1.1897622	-1.1899493	-1.8392088
ZNF385A	zinc finger protein 385A	1.5091757	1.4838315	1.5375831
ZNF395	zinc finger protein 395	1.6635007	1.2781945	-1.0545212
ZNF418	zinc finger protein 418	1.4015678	1.8531255	2.3337126
ZNF420	zinc finger protein 420	1.3352277	1.7161976	2.4689424
ZNF425	zinc finger protein 425	1.5588346	1.5355892	2.0597508
ZNF429	zinc finger protein 429	-1.1641903	-1.6491334	-1.6543179
ZNF444	zinc finger protein 444	1.0841104	1.7741569	1.8758179
ZNF445	zinc finger protein 445	-1.9633259	-1.7270142	-1.9798914
ZNF446	zinc finger protein 446	1.1583451	1.5070162	1.3701195
ZNF462	zinc finger protein 462	-1.0697919	-1.4252129	-2.0957885
ZNF48	zinc finger protein 48	2.221404	2.4062655	2.358913
ZNF497	zinc finger protein 497	2.6375437	3.1063678	3.4766924
ZNF511	zinc finger protein 511	-1.2948633	-1.4354093	-1.515212
ZNF526	zinc finger protein 526	-2.2242713	-1.898564	-1.6680676
ZNF532	zinc finger protein 532	1.3044682	-1.1480508	-2.5516815
ZNF541	zinc finger protein 541	6.3285155	31.227673	21.972742
ZNF542P	zinc finger protein 542, pseudogene	1.5070127	1.6805438	1.3266761
ZNF557	zinc finger protein 557	1.7332152	1.2195169	1.7428865
ZNF561	zinc finger protein 561	1.5186272	1.3479857	1.0065918
ZNF573	zinc finger protein 573	1.1011659	1.6052389	1.0801382
ZNF579	zinc finger protein 579	1.1033472	1.135538	-1.6884155
ZNF584	zinc finger protein 584	1.6148505	1.8526815	1.6564004
ZNF600	zinc finger protein 600	-1.5673976	-1.4810556	-1.9103601
ZNF605	zinc finger protein 605	1.1033033	1.40703	-1.6310462
ZNF618	zinc finger protein 618	-1.1917874	-1.7090963	-4.319825
ZNF628	zinc finger protein 628	-1.278431	-1.6578689	-1.8392162
ZNF629	zinc finger protein 629	1.6383258	1.3737224	-1.3165779
ZNF652	zinc finger protein 652	1.8154672	2.5208995	3.2565823
ZNF658	zinc finger protein 658	1.356484	1.9687937	2.5240457
ZNF658	zinc finger protein 658	1.4094899	2.0217748	2.358998
ZNF713	zinc finger protein 713	1.8604647	1.8585167	1.8313931
ZNF720	zinc finger protein 720	1.0644736	1.4006385	1.6516117
ZNF729	zinc finger protein 729	-1.2320318	-1.6576315	-1.7648829
ZNF746	zinc finger protein 746	2.2956011	2.5112374	2.9436548
ZNF768	zinc finger protein 768	1.8578831	1.9949427	1.3090285
ZNF771	zinc finger protein 771	1.4641546	1.7395664	2.5267541
ZNF777	zinc finger protein 777	2.0131178	1.758489	2.0650742
ZNF777	zinc finger protein 777	1.6589866	1.4539349	1.6737647
ZNF780A	zinc finger protein 780A	1.0434594	2.0426497	3.5023975
ZNF784	zinc finger protein 784	-1.0498143	1.1837058	1.8147303
ZNF83	zinc finger protein 83	-1.9004902	-3.5582302	-4.3340507
ZNF83	zinc finger protein 83	-1.8593946	-3.224995	-3.9916203
ZNF837	zinc finger protein 837	-1.1098477	1.3884436	2.2765496
ZNF843	zinc finger protein 843	2.0744638	2.0032008	2.2470727
ZNF862	zinc finger protein 862	-1.1642323	1.4825983	1.8139694
ZNF90	zinc finger protein 90	2.1628215	1.8959907	1.7289205

ZNHIT2	zinc finger, HIT-type containing 2	1.2398162	1.4508955	1.5888797
ZNRF2	zinc and ring finger 2, E3 ubiquitin protein ligase	-1.2956488	1.3313454	2.8134732
ZNRF3	zinc and ring finger 3	-1.3378292	-1.5773851	-1.6439049
ZP1	zona pellucida glycoprotein 1 (sperm receptor)	2.8963337	-3.4112167	-51.73576
ZSCAN12P1	zinc finger and SCAN domain containing 12 pseudogene 1	1.4201266	1.5886542	1.8310925
ZSCAN2	zinc finger and SCAN domain containing 2	2.110141	2.2722633	2.2873394
ZSCAN5A	zinc finger and SCAN domain containing 5A	1.6938697	1.8584028	2.1531458
ZSWIM6	zinc finger, SWIM-type containing 6	-1.7095523	-2.5908463	-1.5791215
ZWILCH	zwilch kinetochore protein	1.3568758	-2.1378157	-3.2831767
ZWINT	ZW10 interacting kinetochore protein	1.804749	-3.769868	-4.348347
ZWINT	ZW10 interacting kinetochore protein	2.8420105	-2.1421366	-1.9422646
ZXDB	zinc finger, X-linked, duplicated B	1.8380953	2.262978	2.25178
ZYG11B	zyg-11 family member B, cell cycle regulator	1.5978808	1.9062023	2.4991193
ZYX	zyxin	-1.122071	-1.7072753	-4.0050206

Supplementary Table 3: List of 471 mitocarta 3.0 genes significantly deregulated between HepaRG-CSCs and HepaRG differentiated cells

Gene Symbol	GeneName	FC ([D4] vs [STEM])	FC ([D15] vs [STEM])	FC ([D30] vs [STEM])
AARS2	alanyl-tRNA synthetase 2, mitochondrial	-1,58	-2,04	-2,42
AASS	aminoadipate-semialdehyde synthase	1,66	4,08	13,74
ABAT	4-aminobutyrate aminotransferase	1,33	9,19	22,99
ABCA9	ATP-binding cassette, sub-family A (ABC1), member 9	-3,46	-2,8	-2,76
ABCB10	ATP-binding cassette, sub-family B (MDR/TAP), member 10	1,24	1,25	1,68
ACAA1	acetyl-CoA acyltransferase 1	1,27	3,2	8
ACAA2	acetyl-CoA acyltransferase 2	-1,43	1,48	2,33
ACACB	acetyl-CoA carboxylase beta	-1,11	3,33	4,7
ACAD10	acyl-CoA dehydrogenase family, member 10	1,94	2,45	2,95
ACAD11	acyl-CoA dehydrogenase family, member 11	-1,73	2,4	2,9
ACADL	acyl-CoA dehydrogenase, long chain	2,4	6,68	4,63
ACADM	acyl-CoA dehydrogenase, C-4 to C-12 straight chain	-1,01	1,69	1,75
ACADSB	acyl-CoA dehydrogenase, short/branched chain	1,65	4,05	4,23
ACADVL	acyl-CoA dehydrogenase, very long chain	-2,06	-1,3	-1,27
ACAT1	acetyl-CoA acetyltransferase 1	-1,04	1,67	2,52
ACLY	ATP citrate lyase	-1,51	-2,36	-2,26
ACO2	aconitase 2, mitochondrial	1,72	2,47	1,82
ACOT13	acyl-CoA thioesterase 13	1,45	1,63	2,01
ACOT9	acyl-CoA thioesterase 9	1,14	-1,42	-1,93
ACSF2	acyl-CoA synthetase family member 2	1,08	3,08	1,53
ACSM2B	acyl-CoA synthetase medium-chain family member 2B	-1,31	24,23	398,07
ACSM3	acyl-CoA synthetase medium-chain family member 3	-1,43	4,3	4,18
ACSM5	acyl-CoA synthetase medium-chain family member 5	1,33	3,88	5,89
ACSS1	acyl-CoA synthetase short-chain family member 1	3,9	29,42	11,23
ADHFE1	alcohol dehydrogenase, iron containing, 1	-1,3	3,77	4,6
AGMAT	agmatine ureohydrolase (agmatinase)	-1,7	2,12	2,38
AGPAT4	1-acylglycerol-3-phosphate O-acyltransferase 4	-1,26	-3,17	-5,97
AGXT	alanine-glyoxylate aminotransferase	-3,72	16,74	128,5
AGXT2	alanine--glyoxylate aminotransferase 2	-1,84	40,46	121,84
AIFM1	apoptosis-inducing factor, mitochondrion-associated, 1	1,33	2,19	2,47
AIFM2	apoptosis-inducing factor, mitochondrion-associated, 2	1,49	1,63	1,15
AK2	adenylate kinase 2	1,2	1,58	3,03
AK3	adenylate kinase 3	1,22	1,86	1,98
AK4	adenylate kinase 4	-1	1,2	2,22
AKR1B10	aldo-keto reductase family 1, member B10 (aldose reductase)	-3,25	-1,79	-1,14
AKR7A2	aldo-keto reductase family 7, member A2 (aflatoxin aldehyde reductase)	1,51	1,65	2,72
ALDH1L1	aldehyde dehydrogenase 1 family, member L1	1,75	8,02	23
ALDH2	aldehyde dehydrogenase 2 family (mitochondrial)	-2,44	2,01	6,03
ALDH3A2	aldehyde dehydrogenase 3 family, member A2	-1,36	-1,27	1,71
ALDH4A1	aldehyde dehydrogenase 4 family, member A1	1,48	3,77	4,36
ALDH5A1	aldehyde dehydrogenase 5 family, member A1	1,19	2,89	5,88
ALDH6A1	aldehyde dehydrogenase 6 family, member A1	-1,02	4,88	9,43
ALDH7A1	aldehyde dehydrogenase 7 family, member A1	3,88	3,94	5,73
ALDH9A1	aldehyde dehydrogenase 9 family, member A1	1,27	1,71	2,22
ALKBH7	alkB, alkylation repair homolog 7 (E. coli)	1,63	1,78	1,57
AMACR	alpha-methylacyl-CoA racemase	-1,08	1,24	2,29
APEX1	APEX nuclease (multifunctional DNA repair enzyme) 1	-1,1	-1,11	-1,75
APOO	apolipoprotein O	1,48	1,57	1,13
APOOL	apolipoprotein O-like	1,52	1,46	1,9
ARF5	ADP-ribosylation factor 5	1,36	1,3	1,71
ATAD3B	ATPase family, AAA domain containing 3B	-2	-2,2	-2,93
ATPAF2	ATP synthase mitochondrial F1 complex assembly factor 2	-1,09	1,1	1,77
AUH	AU RNA binding protein/enoyl-CoA hydratase	1,24	2,27	2,91
BAK1	BCL2-antagonist/killer 1	1,29	1,14	-1,71
BAX	BCL2-associated X protein	1,24	-1,49	-2,93
BBC3	BCL2 binding component 3	-1,07	-1,26	-1,9
BCAT2	branched chain amino-acid transaminase 2, mitochondrial	1,31	2,2	3,03
BCKDHA	branched chain keto acid dehydrogenase E1, alpha polypeptide	1,12	1,88	1,74
BCKDHB	branched chain keto acid dehydrogenase E1, beta polypeptide	-1,07	1,81	2,8
BCL2L1	BCL2-like 1	-1,09	-1,56	-2,45
BCL2L11	BCL2-like 11 (apoptosis facilitator)	-1,34	1,44	2,25
BCL2L2	BCL2-like 2	-1,45	-1,63	-1,73
BCO2	beta-carotene oxygenase 2	-1,36	5,91	3,6
BDH1	3-hydroxybutyrate dehydrogenase, type 1	-13,91	13,61	43,51
BID	BH3 interacting domain death agonist	-1,01	-1,3	-2,5
BNIP3	BCL2/adenovirus E1B 19kDa interacting protein 3	1,37	1,98	2,71
BNIP3L	BCL2/adenovirus E1B 19kDa interacting protein 3-like	-1,35	-1,71	-1,22
BOLA1	bolA family member 1	1,27	1,79	1,69
BPHL	biphenyl hydrolase-like (serine hydrolase)	-1,47	1,55	1,78
C15orf61	chromosome 15 open reading frame 61	2,22	2,34	2,29
C2orf69	chromosome 2 open reading frame 69	1,26	1,25	2,19
C5orf63	chromosome 5 open reading frame 63	-1,79	-2,23	1,15
C6orf136	chromosome 6 open reading frame 136	1,2	1,78	1,56
C8orf82	chromosome 8 open reading frame 82	1,33	1,53	-1,06

CARS2	cysteinyl-tRNA synthetase 2, mitochondrial (putative)	1,55	1,3	1
CAT	catalase	-1,93	1,17	3,27
CBR4	carbonyl reductase 4	1,02	2,01	2,65
CCDC51	coiled-coil domain containing 51	1,59	1,37	-1,31
CHCHD10	coiled-coil-helix-coiled-coil-helix domain containing 10	1,36	2,12	1,3
CHCHD4	coiled-coil-helix-coiled-coil-helix domain containing 4	1,81	1,88	1,43
CHCHD7	coiled-coil-helix-coiled-coil-helix domain containing 7	2,01	1,48	2,69
CHDH	choline dehydrogenase	-2,66	-1,26	1,27
CHPT1	choline phosphotransferase 1	1,57	1,96	4,65
CISD1	CDGSH iron sulfur domain 1	1,7	1,89	1,5
CKMT1A	creatine kinase, mitochondrial 1A	1,6	1,81	-2,94
CLPB	ClpB caseinolytic peptidase B homolog (E. coli)	-1,56	-1,45	1,04
CLYBL	citrate lyase beta like	1,05	2,13	3,02
CMC1	C-x(9)-C motif containing 1	1,75	2,07	1,45
CMC4	C-x(9)-C motif containing 4	1,09	1,69	1,81
COA3	cytochrome c oxidase assembly factor 3	1,09	1,81	1,76
COA4	cytochrome c oxidase assembly factor 4 homolog (S. cerevisiae)	1,23	1,8	1,64
COA5	cytochrome c oxidase assembly factor 5	-1,06	1,06	1,65
COMTD1	catechol-O-methyltransferase domain containing 1	1,3	1,35	-1,9
COQ10A	coenzyme Q10 homolog A (S. cerevisiae)	1,6	1,93	1,49
COQ5	coenzyme Q5 homolog, methyltransferase (S. cerevisiae)	-1,13	1,18	1,8
COQ9	coenzyme Q9	1,07	1,76	1,49
COX11	COX11 cytochrome c oxidase copper chaperone	1,41	1,3	1,76
COX14	COX14 cytochrome c oxidase assembly factor	1,47	2,04	2,46
COX20	COX20 cytochrome c oxidase assembly factor	1,85	2,07	1,47
COX5A	cytochrome c oxidase subunit Va	2,67	2,94	2,03
COX5B	cytochrome c oxidase subunit Vb	1,98	2,62	1,83
COX6A1	cytochrome c oxidase subunit VIa polypeptide 1	1,61	1,97	2,04
COX6A2	cytochrome c oxidase subunit VIa polypeptide 2	-1,29	2,83	4,06
COX6B1	cytochrome c oxidase subunit VIb polypeptide 1 (ubiquitous)	1,72	2,07	1,42
COX6B2	cytochrome c oxidase subunit VIb polypeptide 2 (testis)	2,25	2,43	2,48
COX6C	cytochrome c oxidase subunit VIc	1,76	2,35	2,09
COX7A2	cytochrome c oxidase subunit VIIa polypeptide 2 (liver)	1,59	1,9	1,65
COX7C	cytochrome c oxidase subunit VIIc	1,96	2,31	2,04
COX8A	cytochrome c oxidase subunit VIIIA (ubiquitous)	1,62	1,85	1,56
CPS1	carbamoyl-phosphate synthase 1, mitochondrial	-1,79	9,96	5,34
CPT1A	carnitine palmitoyltransferase 1A (liver)	-2,45	-1,76	-1,77
CPT2	carnitine palmitoyltransferase 2	-1,22	1,59	2,13
CRAT	carnitine O-acetyltransferase	1,25	2,26	2,79
CRLS1	cardiolipin synthase 1	1,15	1,76	2,42
CRYZ	crystallin, zeta (quinone reductase)	-1,75	1,79	2
CS	citrate synthase	2,14	2,38	1,18
CYB5R3	cytochrome b5 reductase 3	1,55	1,64	1,79
CYC1	cytochrome c-1	1,73	2,2	1,72
CYCS	cytochrome c, somatic	2,01	1,56	1,14
CYP27A1	cytochrome P450, family 27, subfamily A, polypeptide 1	1,8	2,9	4,52
D2HGDH	D-2-hydroxyglutarate dehydrogenase	-1,58	-1,23	1,03
DBI	diazepam binding inhibitor (GABA receptor modulator, acyl-CoA binding protein)	2,02	2,48	2,93
DBT	dihydrolipoamide branched chain transacylase E2	1,01	2,37	3,06
DCXR	dicarbonyl/L-xylulose reductase	1,37	4,34	17,84
DECR1	2,4-dienoyl CoA reductase 1, mitochondrial	1,33	2,5	3,26
DHODH	dihydroorotate dehydrogenase (quinone)	-1,51	1,47	1,18
DHRS1	dehydrogenase/reductase (SDR family) member 1	-1,31	1,09	1,76
DHRS4	dehydrogenase/reductase (SDR family) member 4	1,22	1,51	2,43
DHTKD1	dehydrogenase E1 and transketolase domain containing 1	1,16	3,01	7,4
DIABLO	diablo, IAP-binding mitochondrial protein	1,36	1,68	1,99
DLAT	dihydrolipoamide S-acetyltransferase	1,75	2,1	1,52
DMGDH	dimethylglycine dehydrogenase	-1,45	3,83	9,35
DNAJC19	DnaJ (Hsp40) homolog, subfamily C, member 19	1,41	2,4	2,49
DNAJC30	DnaJ (Hsp40) homolog, subfamily C, member 30	1,17	1,6	1,72
DNAJC4	DnaJ (Hsp40) homolog, subfamily C, member 4	1,63	2,07	1,6
DNLZ	DNL-type zinc finger	1,01	-1,16	-1,68
DTYMK	deoxythymidylate kinase (thymidylate kinase)	1,64	-1,5	-1,44
DUT	deoxyuridine triphosphatase	-1	-1,65	-1,81
ECHDC1	ethylmalonyl-CoA decarboxylase 1	1,71	2,14	1,79
ECHDC2	enoyl CoA hydratase domain containing 2	-1,12	3,05	9
ECHDC3	enoyl CoA hydratase domain containing 3	1,03	2,35	2,97
ECHS1	enoyl CoA hydratase, short chain, 1, mitochondrial	1,14	1,76	3,13
ECI1	enoyl-CoA delta isomerase 1	1,75	2,76	3,24
ECI2	enoyl-CoA delta isomerase 2	1,31	1,75	2,24
EHHADH	enoyl-CoA, hydratase/3-hydroxyacyl CoA dehydrogenase	-1,61	2,3	6,51
ENDOG	endonuclease G	1,6	1,95	1,77
EPHX2	epoxide hydrolase 2, cytoplasmic	-1,07	4,66	8,36
ETFA	electron-transfer-flavoprotein, alpha polypeptide	-1,83	-1,35	1,18
ETFB	electron-transfer-flavoprotein, beta polypeptide	-1,03	1,6	2,03
ETFDH	electron-transferring-flavoprotein dehydrogenase	-1,3	1,36	2,41
ETHE1	ethylmalonic encephalopathy 1	1,17	1,4	1,83

FABP1	fatty acid binding protein 1, liver	-14,94	8,49	37,85
FAHD1	fumarylacetoacetate hydrolase domain containing 1	1,43	1,66	2,31
FAHD2A	fumarylacetoacetate hydrolase domain containing 2A	1,12	1,46	1,7
FAM136A	family with sequence similarity 136, member A	1,57	1,49	-1,16
FAM162A	family with sequence similarity 162, member A	1,67	2,18	2,39
FASN	fatty acid synthase	2,29	2,71	4,1
FASTKD1	FAST kinase domains 1	1,42	1,1	-1,5
FDPS	farnesyl diphosphate synthase	1,7	1,7	2,53
FDX1	ferredoxin 1	1,42	2,3	2,38
FDXR	ferredoxin reductase	1,63	1,32	-2,01
FH	fumarate hydratase	1,22	1,59	1,58
FPGS	folylpolyglutamate synthase	-1,24	-1,43	-1,88
FUNDC2	FUN14 domain containing 2	-1,03	-1,13	-1,73
FXN	frataxin	1,77	1,69	1,52
GATM	glycine amidinotransferase (L-arginine:glycine amidinotransferase)	-1,55	11,78	21,28
GCDH	glutaryl-CoA dehydrogenase	-1,36	1,25	4,85
GCSH	glycine cleavage system protein H (aminomethyl carrier)	1,69	1,79	2,02
GFER	growth factor, augmenter of liver regeneration	1,7	1,6	1,86
GFM2	G elongation factor, mitochondrial 2	1,51	1,44	1,65
GHITM	growth hormone inducible transmembrane protein	1,21	1,47	1,62
GLDC	glycine dehydrogenase (decarboxylating)	1,22	6,36	17,25
GLOD4	glyoxalase domain containing 4	-1,03	1,18	1,57
GLRX5	glutaredoxin 5	1,74	2,4	2,57
GLS	glutaminase	-1,16	-1,55	-1,8
GLS2	glutaminase 2 (liver, mitochondrial)	-1,29	1,21	10,65
GLUD1	glutamate dehydrogenase 1	-1,29	1,24	2,78
GLUD2	glutamate dehydrogenase 2	-1,14	1,34	3,18
GLYAT	glycine-N-acyltransferase	-1,48	50,84	227,32
GLYCTK	glycerate kinase	1,03	9,12	13,76
GPD2	glycerol-3-phosphate dehydrogenase 2 (mitochondrial)	1,47	-1,4	-2,17
GPX1	glutathione peroxidase 1	1,7	1,63	1,26
GPX4	glutathione peroxidase 4	-1,07	1,22	1,84
GRHPR	glyoxylate reductase/hydroxypyruvate reductase	1,52	3,49	4,6
GRPEL2	GrpE-like 2, mitochondrial (E. coli)	-1,03	-1,45	-1,96
GSTK1	glutathione S-transferase kappa 1	1,28	1,92	2,25
GSTZ1	glutathione S-transferase zeta 1	1,01	1,33	2,61
HADH	hydroxyacyl-CoA dehydrogenase	1,14	1,28	2,65
HAGH	hydroxyacylglutathione hydrolase	1,13	2,03	6,26
HAO2	hydroxyacid oxidase 2 (long chain)	-2,22	38,7	53,28
HCCS	holocytochrome c synthase	1,21	-1,05	-1,61
HEBP1	heme binding protein 1	1,82	2,54	2,55
HIBCH	3-hydroxyisobutyryl-CoA hydrolase	-1,11	1,12	1,65
HIGD1A	HIG1 hypoxia inducible domain family, member 1A	1,78	1,86	2,47
HINT2	histidine triad nucleotide binding protein 2	1,27	2,15	2,15
HMGCL	3-hydroxymethyl-3-methylglutaryl-CoA lyase	-1,5	1,89	3,16
HMGCS2	3-hydroxy-3-methylglutaryl-CoA synthase 2 (mitochondrial)	-19,08	4,52	5,2
HOGA1	4-hydroxy-2-oxoglutarate aldolase 1	11,54	36,94	24,51
HSD17B10	hydroxysteroid (17-beta) dehydrogenase 10	1,11	1,52	1,95
HSD17B4	hydroxysteroid (17-beta) dehydrogenase 4	-1,14	1,5	1,84
HSD17B8	hydroxysteroid (17-beta) dehydrogenase 8	1,22	2,42	2,23
HSPE1	heat shock 10kDa protein 1	1,45	1,53	1,38
IDE	insulin-degrading enzyme	-1,2	-1,55	-1,62
IDH2	isocitrate dehydrogenase 2 (NADP+), mitochondrial	1,14	1,28	-1,9
IFI27	interferon, alpha-inducible protein 27	1,57	-1,2	-2,27
ISCA2	iron-sulfur cluster assembly 2	1,84	2,17	2,26
ISCU	iron-sulfur cluster assembly enzyme	1,31	1,56	1,48
IVD	isovaleryl-CoA dehydrogenase	1,45	2,54	4,41
L2HGDH	L-2-hydroxyglutarate dehydrogenase	-3,76	-2,5	-1,82
LACTB2	lactamase, beta 2	1,87	1,7	1,36
LAP3	leucine aminopeptidase 3	1,35	1,45	1,91
LDHB	lactate dehydrogenase B	-2,06	1,07	-1,74
LDHD	lactate dehydrogenase D	1,57	6,7	7,9
LETM1	leucine zipper-EF-hand containing transmembrane protein 1	1,1	-1,24	-1,59
LETMD1	LETM1 domain containing 1	1,09	1,84	1,48
LIPT2	lipoyl(octanoyl) transferase 2 (putative)	1,35	1,11	2,31
LYPLAL1	lysophospholipase-like 1	-1,12	1,58	2,17
LYRM9	LYR motif containing 9	1,84	3,65	6,69
MAOA	monoamine oxidase A	5,45	10,38	9,6
MAOB	monoamine oxidase B	-1,28	1,93	2,64
MAVS	mitochondrial antiviral signaling protein	1,47	1,6	1,7
MCCC1	methylcrotonoyl-CoA carboxylase 1 (alpha)	1,12	2,35	1,68
MCCC2	methylcrotonoyl-CoA carboxylase 2 (beta)	-1,55	-1,02	1,35
MCEE	methylmalonyl CoA epimerase	-1,11	2,11	2,5
MCUR1	mitochondrial calcium uniporter regulator 1	1,38	1,39	1,63
ME3	malic enzyme 3, NADP(+)-dependent, mitochondrial	1,16	-1,05	-1,59
METTL15	methyltransferase like 15	1,24	1,46	1,92
METTL8	methyltransferase like 8	1,18	-1,06	-1,71

MFF	mitochondrial fission factor	1,74	1,73	1,53
MFN1	mitofusin 1	1,2	1,35	1,62
MFN2	mitofusin 2	1,57	1,59	1,48
MGME1	mitochondrial genome maintenance exonuclease 1	1,23	-1,51	-2,16
MGST1	microsomal glutathione S-transferase 1	1,06	1,39	2,46
MIEF1	mitochondrial elongation factor 1	1,51	1,72	2,02
MLYCD	malonyl-CoA decarboxylase	1,7	1,69	2,24
MMAA	methylmalonic aciduria (cobalamin deficiency) cblA type	-1,31	1,67	2,77
MMAB	methylmalonic aciduria (cobalamin deficiency) cblB type	1,54	1,98	4,66
MOCS1	molybdenum cofactor synthesis 1	1,16	2,86	3,6
MPC1	mitochondrial pyruvate carrier 1	2,52	3,69	3,63
MPC2	mitochondrial pyruvate carrier 2	1,32	2,11	2,88
MPST	mercaptopyruvate sulfurtransferase	-1,24	1,66	2,54
MPV17	MpV17 mitochondrial inner membrane protein	1,04	1,1	-1,51
MPV17L2	MPV17 mitochondrial membrane protein-like 2	1,39	1,12	1,6
MRPL15	mitochondrial ribosomal protein L15	1,96	2,14	1,23
MRPL16	mitochondrial ribosomal protein L16	1,59	2,15	2,36
MRPL17	mitochondrial ribosomal protein L17	1,56	1,27	-1,13
MRPL19	mitochondrial ribosomal protein L19	1,34	1,33	1,9
MRPL20	mitochondrial ribosomal protein L20	1,51	1,51	1,7
MRPL21	mitochondrial ribosomal protein L21	1,59	1,67	1,4
MRPL23	mitochondrial ribosomal protein L23	1,18	2,38	27,92
MRPL3	mitochondrial ribosomal protein L3	1,53	1,43	1,34
MRPL30	mitochondrial ribosomal protein L30	1,5	1,37	1,71
MRPL32	mitochondrial ribosomal protein L32	1,44	1,57	1,45
MRPL33	mitochondrial ribosomal protein L33	1,86	1,21	-1,17
MRPL34	mitochondrial ribosomal protein L34	1,29	1,61	1,58
MRPL4	mitochondrial ribosomal protein L4	1,71	1,97	1,58
MRPL40	mitochondrial ribosomal protein L40	1,5	1,97	1,86
MRPL41	mitochondrial ribosomal protein L41	1,29	1,97	1,93
MRPL47	mitochondrial ribosomal protein L47	1,64	1,52	1,03
MRPL52	mitochondrial ribosomal protein L52	-1,12	-1,43	-2,85
MRPL53	mitochondrial ribosomal protein L53	1,5	1,51	1,63
MRPL57	mitochondrial ribosomal protein L57	-1,59	-1,45	-2,19
MRPS12	mitochondrial ribosomal protein S12	1,97	2,6	1,6
MRPS14	mitochondrial ribosomal protein S14	1,43	1,23	1,72
MRPS18B	mitochondrial ribosomal protein S18B	1,32	1,91	1,55
MRPS18C	mitochondrial ribosomal protein S18C	1,51	1,41	1,71
MRPS21	mitochondrial ribosomal protein S21	1,53	1,48	1,07
MRPS22	mitochondrial ribosomal protein S22	1,56	1,84	1,78
MRPS24	mitochondrial ribosomal protein S24	1,86	1,83	1,29
MRPS26	mitochondrial ribosomal protein S26	1,45	1,73	1,84
MRPS28	mitochondrial ribosomal protein S28	1,29	1,28	1,53
MRPS31	mitochondrial ribosomal protein S31	1,11	1,14	1,55
MRPS36	mitochondrial ribosomal protein S36	2,55	3,04	2,74
MRPS9	mitochondrial ribosomal protein S9	1,41	1,6	1,42
MRS2	MRS2 magnesium transporter	2,37	2,2	1,34
MSRA	methionine sulfoxide reductase A	1,03	1,22	2,14
MSRB2	methionine sulfoxide reductase B2	1,08	1,65	2,37
MSRB3	methionine sulfoxide reductase B3	1,87	1,7	1,11
MTCH2	mitochondrial carrier 2	-1,14	1,23	1,76
MTERF4	mitochondrial transcription termination factor 4	1,23	1,28	1,58
MTFR1L	mitochondrial fission regulator 1-like	1,78	2,38	2
MTFR2	mitochondrial fission regulator 2	2,05	-2,84	-3,87
MTHFD1L	methylenetetrahydrofolate dehydrogenase (NADP+ dependent) 1-like	-1,45	-3,4	-10,15
MTHFS	5,10-methenyltetrahydrofolate synthetase (5-formyltetrahydrofolate cyclo-ligase)	-1,13	1,33	2,54
MTRF1L	mitochondrial translational release factor 1-like	1,03	-1,22	-1,58
MYO19	myosin XIX	1,27	-1,22	-3,82
NBR1	neighbor of BRCA1 gene 1	1,09	1,17	1,57
NDUFA1	NADH dehydrogenase (ubiquinone) 1 alpha subcomplex, 1, 7.5kDa	1,7	1,77	1,67
NDUFA11	NADH dehydrogenase (ubiquinone) 1 alpha subcomplex, 11, 14.7kDa	1,72	1,5	1,35
NDUFA12	NADH dehydrogenase (ubiquinone) 1 alpha subcomplex, 12	1,82	1,97	1,63
NDUFA2	NADH dehydrogenase (ubiquinone) 1 alpha subcomplex, 2, 8kDa	1,55	2,02	2,44
NDUFA3	NADH dehydrogenase (ubiquinone) 1 alpha subcomplex, 3, 9kDa	1,44	1,49	2,29
NDUFA5	NADH dehydrogenase (ubiquinone) 1 alpha subcomplex, 5	1,45	1,84	1,9
NDUFA6	NADH dehydrogenase (ubiquinone) 1 alpha subcomplex, 6, 14kDa	1,93	2,1	1,84
NDUFA7	NADH dehydrogenase (ubiquinone) 1 alpha subcomplex, 7, 14.5kDa	1,62	1,65	1,73
NDUFA8	NADH dehydrogenase (ubiquinone) 1 alpha subcomplex, 8, 19kDa	2,17	2,43	1,72
NDUFAB1	NADH dehydrogenase (ubiquinone) 1, alpha/beta subcomplex, 1, 8kDa	1,4	1,72	1,89
NDUFAF1	NADH dehydrogenase (ubiquinone) complex I, assembly factor 1	-1,09	1,24	1,96
NDUFAF2	NADH dehydrogenase (ubiquinone) complex I, assembly factor 2	-1,32	-1,7	-1,85
NDUFAF3	NADH dehydrogenase (ubiquinone) complex I, assembly factor 3	1,7	1,49	1,86
NDUFAF4	NADH dehydrogenase (ubiquinone) complex I, assembly factor 4	1,61	1,34	-1,21
NDUFAF6	NADH dehydrogenase (ubiquinone) complex I, assembly factor 6	1,64	2,08	1,71
NDUFB1	NADH dehydrogenase (ubiquinone) 1 beta subcomplex, 1, 7kDa	1,7	2,11	2,25
NDUFB10	NADH dehydrogenase (ubiquinone) 1 beta subcomplex, 10, 22kDa	2,04	2,51	2,19
NDUFB2	NADH dehydrogenase (ubiquinone) 1 beta subcomplex, 2, 8kDa	1,67	1,69	1,65

NDUFB3	NADH dehydrogenase (ubiquinone) 1 beta subcomplex, 3, 12kDa	1,79	1,94	1,64
NDUFB4	NADH dehydrogenase (ubiquinone) 1 beta subcomplex, 4, 15kDa	1,85	2,09	1,95
NDUFB5	NADH dehydrogenase (ubiquinone) 1 beta subcomplex, 5, 16kDa	1,61	2,33	1,63
NDUFB6	NADH dehydrogenase (ubiquinone) 1 beta subcomplex, 6, 17kDa	1,58	1,76	1,95
NDUFB7	NADH dehydrogenase (ubiquinone) 1 beta subcomplex, 7, 18kDa	1,41	1,65	1,51
NDUFB8	NADH dehydrogenase (ubiquinone) 1 beta subcomplex, 8, 19kDa	1,52	1,5	1,31
NDUFB9	NADH dehydrogenase (ubiquinone) 1 beta subcomplex, 9, 22kDa	1,22	1,68	1,14
NDUFC1	NADH dehydrogenase (ubiquinone) 1, subcomplex unknown, 1, 6kDa	1,27	1,55	1,51
NDUFC2	NADH dehydrogenase (ubiquinone) 1, subcomplex unknown, 2, 14.5kDa	1,45	2,08	2,05
NDUFS3	NADH dehydrogenase (ubiquinone) Fe-S protein 3, 30kDa (NADH-coenzyme Q reductase)	1,43	1,74	1,37
NDUFS5	NADH dehydrogenase (ubiquinone) Fe-S protein 5, 15kDa (NADH-coenzyme Q reductase)	1,69	1,33	1,32
NDUFS6	NADH dehydrogenase (ubiquinone) Fe-S protein 6, 13kDa (NADH-coenzyme Q reductase)	1,86	1,62	1,6
NDUFV2	NADH dehydrogenase (ubiquinone) flavoprotein 2, 24kDa	1,13	1,43	1,67
NDUFV3	NADH dehydrogenase (ubiquinone) flavoprotein 3, 10kDa	1,14	1,41	1,75
NFS1	NFS1 cysteine desulfurase	1,04	1,46	1,67
NGRN	neugrin, neurite outgrowth associated	-1,96	-1,83	-1,65
NIPSNAP1	nipsnap homolog 1 (C. elegans)	-1,06	1,73	2,78
NIT1	nitrilase 1	-1,3	1,36	1,96
NIT2	nitrilase family, member 2	1,31	1,77	2,61
NLRX1	NLR family member X1	1,05	1,21	1,78
NME3	NME/NM23 nucleoside diphosphate kinase 3	-1,07	1,41	1,54
NME4	NME/NM23 nucleoside diphosphate kinase 4	1,07	-1,37	-3
NSUN4	NOP2/Sun domain family, member 4	-1,61	-1,51	-1,24
NT5M	5',3'-nucleotidase, mitochondrial	2,02	3,02	1,75
NUDT8	nudix (nucleoside diphosphate linked moiety X)-type motif 8	1,36	2,34	2,89
NUDT9	nudix (nucleoside diphosphate linked moiety X)-type motif 9	1,13	1,36	2,1
OGDHL	oxoglutarate dehydrogenase-like	2,06	5,03	3,8
OMA1	OMA1 zinc metallopeptidase	-1,58	-1,17	1,07
OSBPL1A	oxysterol binding protein-like 1A	2,65	3,54	3,02
OSGEPL1	O-sialoglycoprotein endopeptidase-like 1	1,04	1,25	1,7
OTC	ornithine carbamoyltransferase	-5,49	13,7	102,1
OXLD1	oxidoreductase-like domain containing 1	1,42	1,79	1,77
OXSM	3-oxoacyl-ACP synthase, mitochondrial	1,81	2,32	2,34
PANK2	pantothenate kinase 2	-1,35	-1,65	-1,46
PARS2	prolyl-tRNA synthetase 2, mitochondrial (putative)	1,3	1,79	1,74
PC	pyruvate carboxylase	-1,06	2	2,69
PCBD2	pterin-4 alpha-carbinolamine dehydratase/dimerization cofactor of hepatocyte nuclear factor 1 alpha	1,02	1,08	1,78
PCCA	propionyl CoA carboxylase, alpha polypeptide	-1,7	1,83	3,07
PCCB	propionyl CoA carboxylase, beta polypeptide	-1,28	1,25	2,26
PCK2	phosphoenolpyruvate carboxykinase 2 (mitochondrial)	-2,48	2,64	9,72
PDE12	phosphodiesterase 12	1,58	1,4	1,69
PDHA1	pyruvate dehydrogenase (lipoamide) alpha 1	1,67	1,87	1,41
PDHB	pyruvate dehydrogenase (lipoamide) beta	1,67	1,84	2,14
PDK2	pyruvate dehydrogenase kinase, isozyme 2	-1,23	1,93	1,59
PDK4	pyruvate dehydrogenase kinase, isozyme 4	-11,04	-5,64	-2,75
PDP2	pyruvate dehyrogenase phosphatase catalytic subunit 2	1,23	1,25	1,88
PDPR	pyruvate dehydrogenase phosphatase regulatory subunit	-1,38	-1,29	-1,69
PDSS1	prenyl (decaprenyl) diphosphate synthase, subunit 1	1,73	1,42	-1,57
PDSS2	prenyl (decaprenyl) diphosphate synthase, subunit 2	-1,33	1,27	1,51
PEX11B	peroxisomal biogenesis factor 11 beta	1,65	1,77	2,05
PHYH	phytanoyl-CoA 2-hydroxylase	1,08	1,42	2,29
PIF1	PIF1 5'-to-3' DNA helicase	3,65	-2,1	-2,05
PINK1	PTEN induced putative kinase 1	-1,19	-1,25	1,87
PITRM1	pitrilysin metallopeptidase 1	-1,99	-1,91	-2,26
PMAIP1	phorbol-12-myristate-13-acetate-induced protein 1	-2,79	-10,12	-12,66
PNKD	paroxysmal nonkinesigenic dyskinesia	1,62	1,63	1,38
PNPO	pyridoxamine 5'-phosphate oxidase	1,5	1,71	2,44
PNPT1	polyribonucleotide nucleotidyltransferase 1	1,11	-1,35	-1,71
POLDIP2	polymerase (DNA-directed), delta interacting protein 2	1,18	1,65	2,14
PPA2	pyrophosphatase (inorganic) 2	1,41	1,62	1,87
PPIF	peptidylprolyl isomerase F	1,73	1,68	1,69
PRDX5	peroxiredoxin 5	-1,07	1,11	-1,56
PRDX6	peroxiredoxin 6	1,69	2,56	2,99
PRELID1	PRELI domain containing 1	1,31	1,58	1,64
PRODH	proline dehydrogenase (oxidase) 1	2,92	9,91	8,48
PRODH2	proline dehydrogenase (oxidase) 2	-10,5	2,55	4,93
PUSL1	pseudouridylate synthase-like 1	1,52	1,54	1,07
PXMP2	peroxisomal membrane protein 2, 22kDa	1,9	6,14	6,74
PXMP4	peroxisomal membrane protein 4, 24kDa	2,07	2,04	2,06
PYCR2	pyrroline-5-carboxylate reductase family, member 2	1,47	1,52	1,49
QDPR	quinoid dihydropteridine reductase	1,26	1,74	4,99
RARS2	arginyl-tRNA synthetase 2, mitochondrial	1,81	1,43	1,26
RBFA	ribosome binding factor A (putative)	1,56	1,28	1,19
RDH14	retinol dehydrogenase 14 (all-trans/9-cis/11-cis)	1,32	1,44	1,66
REXO2	RNA exonuclease 2	1,68	1,19	-1,05
RMDN1	regulator of microtubule dynamics 1	1,48	1,96	2,04
RMDN3	regulator of microtubule dynamics 3	-1,07	-1,07	1,69

RPIA	ribose 5-phosphate isomerase A	1,4	-1,1	-1,57
SAMM50	SAMM50 sorting and assembly machinery component	1,57	1,76	1,19
SARDH	sarcosine dehydrogenase	-1,69	1,33	1,47
SARS2	seryl-tRNA synthetase 2, mitochondrial	-1,04	-1,06	-1,59
SCP2	sterol carrier protein 2	-1,23	2,45	14,38
SDHA	succinate dehydrogenase complex, subunit A, flavoprotein (Fp)	1,39	1,79	1,59
SDHC	succinate dehydrogenase complex, subunit C, integral membrane protein, 15kDa	2,07	2,42	2,93
SDHD	succinate dehydrogenase complex, subunit D, integral membrane protein	1,51	2,33	2,4
SDR39U1	short chain dehydrogenase/reductase family 39U, member 1	1,05	1,27	1,72
SFXN2	sideroflexin 2	1,36	2,02	2,85
SFXN4	sideroflexin 4	1,19	1,56	-1,26
SFXN5	sideroflexin 5	2,63	2,38	2,95
SHMT2	serine hydroxymethyltransferase 2 (mitochondrial)	-1,29	1,14	1,93
SIRT3	sirtuin 3	1,21	2,28	2,79
SIRT5	sirtuin 5	1,1	1,57	1,03
SLC25A1	solute carrier family 25 (mitochondrial carrier; citrate transporter), member 1	1,1	1,96	2,87
SLC25A10	solute carrier family 25 (mitochondrial carrier; dicarboxylate transporter), member 10	1,34	1,85	2,72
SLC25A11	solute carrier family 25 (mitochondrial carrier; oxoglutarate carrier), member 11	1,64	2,42	2,2
SLC25A13	solute carrier family 25 (aspartate/glutamate carrier), member 13	1,06	1,13	1,98
SLC25A15	solute carrier family 25 (mitochondrial carrier; ornithine transporter) member 15	1,32	1,81	1,85
SLC25A16	solute carrier family 25 (mitochondrial carrier), member 16	-3,13	-2,81	-1,91
SLC25A18	solute carrier family 25 (glutamate carrier), member 18	-1,9	1,18	-1,33
SLC25A20	solute carrier family 25 (carnitine/acylcarnitine translocase), member 20	-1,05	1,83	2,29
SLC25A22	solute carrier family 25 (mitochondrial carrier: glutamate), member 22	1,55	2,15	1,81
SLC25A24	solute carrier family 25 (mitochondrial carrier; phosphate carrier), member 24	1,87	1,44	1,3
SLC25A25	solute carrier family 25 (mitochondrial carrier; phosphate carrier), member 25	-1,09	-1,16	3,09
SLC25A29	solute carrier family 25 (mitochondrial carnitine/acylcarnitine carrier), member 29	-1,6	-1,42	-1,34
SLC25A3	solute carrier family 25 (mitochondrial carrier; phosphate carrier), member 3	1,61	1,78	1,33
SLC25A30	solute carrier family 25, member 30	1,4	1,5	2,18
SLC25A32	solute carrier family 25 (mitochondrial folate carrier), member 32	1,73	1,22	1,13
SLC25A33	solute carrier family 25 (pyrimidine nucleotide carrier), member 33	-1,07	1,32	4,17
SLC25A38	solute carrier family 25, member 38	1,44	1,57	2,62
SLC25A39	solute carrier family 25, member 39	-1,92	-1,55	-1,65
SLC25A4	solute carrier family 25 (mitochondrial carrier; adenine nucleotide translocator), member 4	1,26	2,94	2,65
SLC25A42	solute carrier family 25, member 42	-1,26	2,04	4,01
SLC25A44	solute carrier family 25, member 44	1,43	1,2	2,52
SLC25A5	solute carrier family 25 (mitochondrial carrier; adenine nucleotide translocator), member 5	2,15	2,36	1,53
SLC25A51	solute carrier family 25, member 51	-3,22	-2,97	-2,24
SLC25A6	solute carrier family 25 (mitochondrial carrier; adenine nucleotide translocator), member 6	1,65	1,51	-1,45
SLIRP	SRA stem-loop interacting RNA binding protein	1,63	1,58	1,31
SOD1	superoxide dismutase 1, soluble	1,17	1,23	1,66
SOD2	superoxide dismutase 2, mitochondrial	-1,99	1,04	-2,97
SPG7	spastic paraplegia 7 (pure and complicated autosomal recessive)	-1,88	-1,92	-1,39
SPHK2	sphingosine kinase 2	1,36	1,56	1,36
SPRYD4	SPRY domain containing 4	1,01	1,48	1,78
SSBP1	single-stranded DNA binding protein 1, mitochondrial	1,65	1,33	1,21
SUCLG1	succinate-CoA ligase, alpha subunit	1,7	2,39	1,96
SUCLG2	succinate-CoA ligase, GDP-forming, beta subunit	1,08	1,44	2,04
SUOX	sulfite oxidase	-1,21	1,65	2,48
SURF1	surfeit 1	1,29	2,06	2,96
TACO1	translational activator of mitochondrially encoded cytochrome c oxidase I	1,26	2,1	2,55
TAMM41	TAM41, mitochondrial translocator assembly and maintenance protein, homolog (S. cerevisiae)	1,5	1,15	1,35
TCAIM	T cell activation inhibitor, mitochondrial	1,16	1,44	1,83
TIMM10	translocase of inner mitochondrial membrane 10 homolog (yeast)	1,54	1,51	1,43
TIMM10B	translocase of inner mitochondrial membrane 10 homolog B (yeast)	1,56	1,97	1,73
TIMM17A	translocase of inner mitochondrial membrane 17 homolog A (yeast)	1,66	1,2	1,15
TIMM8A	translocase of inner mitochondrial membrane 8 homolog A (yeast)	1,65	1,69	1,39
TIMM8B	translocase of inner mitochondrial membrane 8 homolog B (yeast)	1,74	1,67	2,08
TK2	thymidine kinase 2, mitochondrial	1,12	1,76	2,74
TMEM11	transmembrane protein 11	1,52	1,38	1,41
TMEM143	transmembrane protein 143	1,21	1,7	1,74
TMEM14C	transmembrane protein 14C	1,64	1,28	1,14
TMEM177	transmembrane protein 177	1,38	1,74	2,02
TMEM186	transmembrane protein 186	1,25	1,77	1,57
TMEM205	transmembrane protein 205	-1,59	1,68	2,2
TOMM20	translocase of outer mitochondrial membrane 20 homolog (yeast)	1,75	1,64	1,04
TOMM22	translocase of outer mitochondrial membrane 22 homolog (yeast)	1,67	1,34	1,11
TOMM7	translocase of outer mitochondrial membrane 7 homolog (yeast)	1,25	1,37	1,56
TRAP1	TNF receptor-associated protein 1	-1,71	-1,29	-1,49
TRIAP1	TP53 regulated inhibitor of apoptosis 1	1,57	1,07	1,01
TRMT1	tRNA methyltransferase 1 homolog (S. cerevisiae)	-1,29	-1,57	-2,18
TRMT10C	tRNA methyltransferase 10 homolog C (S. cerevisiae)	1,85	1,64	1,57
TSFM	Ts translation elongation factor, mitochondrial	1,31	1,31	1,66
TSPO	translocator protein (18kDa)	1,31	1,02	-1,7
TST	thiosulfate sulfurtransferase (rhodanese)	-1,23	2,91	5,67
TTC19	tetratricopeptide repeat domain 19	1,09	1,25	1,94
TXNRD1	thioredoxin reductase 1	1,79	1,01	1,05

UCP3	uncoupling protein 3 (mitochondrial, proton carrier)	3,06	3,48	4,13
UQCC2	ubiquinol-cytochrome c reductase complex assembly factor 2	-1,22	-1,66	-1,43
UQCR10	ubiquinol-cytochrome c reductase, complex III subunit X	1,49	2,21	1,28
UQCR11	ubiquinol-cytochrome c reductase, complex III subunit XI	1,45	1,74	1,76
UQCRB	ubiquinol-cytochrome c reductase binding protein	2,06	2,37	1,85
UQCRC2	ubiquinol-cytochrome c reductase core protein II	1,84	2,46	1,51
UQCRFS1	ubiquinol-cytochrome c reductase, Rieske iron-sulfur polypeptide 1	2,29	2,51	2,23
UQCRH	ubiquinol-cytochrome c reductase hinge protein	1,94	1,98	1,4
UQCRQ	ubiquinol-cytochrome c reductase, complex III subunit VII, 9.5kDa	1,35	1,84	2,2
VARS2	valyl-tRNA synthetase 2, mitochondrial	1,04	1,63	1,6
VDAC2	voltage-dependent anion channel 2	1,59	1,5	1,1
YBEY	ybeY metallopeptidase (putative)	1,04	1,52	1,49

Supplementary Table 4: Clinical and biological features of patients harboring PPARG-positive HCCs

ID NUMBER	Age	Sex	Height	Weight	BMI	Type I Diabetes	Type II Diabetes	AT	Dyslipidemia	Alcohol abuse	Tobacco	Cirrhosis	Hemochro matosis	Child	HBV	HCV	AFP ng/ml
1	88	M	172	90	30	0	1	0	1	1	1	1	0		0	0	7,7
2	62	M	163	90	34	0	0	1	1	1	0	1	0	A	0	0	84,3
3	54	M	170	77	27	0	1	0	0	1	0	1	0	C	1	0	6,0
4	76	M	169	85	30	0	1	0	0	1	0	1	0	A			7,6
5	74	M	172	72	24	0	0	0	0	1	1	1	0	A			5,9
6	66	M	170	93	32	0	0	0	0	1	0	1	0	A	0	0	9,6
7	67	F	167	62	22	NC	NC	NC	NC	0	0	1	0	A			3,6
8	72	M	165	70	26	NC	NC	0	0	0	0	0	0		0	0	7,6
9	78	F	162	70	27	0	0	1	1	1	0	1	0	A	0	0	5,2

ID NUMBER	Lipiodol (neo)	Chemo (neo)	Radiotherapy (neo)	TACE (neo)	Alcoholisation (neo)	RFA (neo)	Tumor size* (cm)	∑ Tumor sizes (cm)	Nber of nodules	Tumor emboli	Iron (Kupffer)	Iron (Hepatocytes)	Margins (cm)	Free margins
1	0	0	0	0	0	0	6	6	1	1	0	1	0,4	1
2	0	0	0	0	0	0	7	7	1	1	1	1	0,3	1
3	0	0	0	0	0	0	3	11	5	0	1	1	transplant	
4	1	0	0	0	0	0	5	5	1	0	0	1	free	1
5	0	0	0	0	0	0	4,5	5,9	3	1	0	1	0,2	1
6	0	0	0	0	0	0	10	11	1	0	0	1	1,2	1
7	0	0	0	0	0	0	4	6,9	4	0	0	0	0,1	1
8	0	0	0	0	0	0	3	3	1	0	0	1	2	1
9	0	0	0	0	0	0	8	8	1	0			0,11	1

Anonymized ID numbers are given. BMI, body mass index; AT, arterial hypertension; HBV, positive serology for hepatitis B virus; HCV, positive serology for hepatitis C virus; AFP, alpha fetoprotein (ng/ml).
Neoadjuvant treatments (neo): lipiodol; chemotherapy (chemo); trans-arterial chemoembolization (TACE); alcoholisation; radiofrequency ablation (RFA);
Tumor size*, size of the largest tumor; ∑, sum of tumor sizes in case of multiple nodules. Empty cells, unavailable data. One case with tumor-free margins had not available quantitative information.
